# Computational ligand design in enantio- and diastereoselective ynamide [5+2] cycloisomerization

**DOI:** 10.1038/ncomms10109

**Published:** 2016-01-05

**Authors:** R. N. Straker, Q. Peng, A. Mekareeya, R. S. Paton, E. A. Anderson

**Affiliations:** 1 Chemistry Research Laboratory, University of Oxford, 12 Mansfield Road, Oxford OX1 3TA, UK

## Abstract

Transition metals can catalyse the stereoselective synthesis of cyclic organic molecules in a highly atom-efficient process called cycloisomerization. Many diastereoselective (substrate stereocontrol), and enantioselective (catalyst stereocontrol) cycloisomerizations have been developed. However, asymmetric cycloisomerizations where a chiral catalyst specifies the stereochemical outcome of the cyclization of a single enantiomer substrate—regardless of its inherent preference—are unknown. Here we show how a combined theoretical and experimental approach enables the design of a highly reactive rhodium catalyst for the stereoselective cycloisomerization of ynamide-vinylcyclopropanes to [5.3.0]-azabicycles. We first establish highly diastereoselective cycloisomerizations using an achiral catalyst, and then explore phosphoramidite-complexed rhodium catalysts in the enantioselective variant, where theoretical investigations uncover an unexpected reaction pathway in which the electronic structure of the phosphoramidite dramatically influences reaction rate and enantioselectivity. A marked enhancement of both is observed using the optimal theory-designed ligand, which enables double stereodifferentiating cycloisomerizations in both matched and mismatched catalyst–substrate settings.

## Abstract (web summary)

Using a chiral catalyst to override the innate stereochemical outcome of a diastereoselective process is a challenging task. Here, the authors use theory and experiment to develop a cycloisomerization where the enantioselectivity is driven by the electronic nature of the ligand regardless of the reaction's inherent diastereoselectivity.

## Graphical abstract

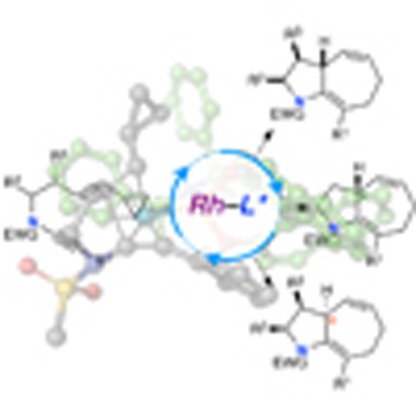

Demand for higher efficiency, economy and selectivity in the synthesis of novel molecular scaffolds drives organic chemistry^1^,^2^. In this context, cycloisomerizations represent ideal methods for the formation of cyclic organic molecules, as they can fulfil all of these criteria. Despite much research into transition metal-catalysed cycloisomerization^3^,^4^, and reports where high enantioselectivity is achieved for the cyclization of prochiral substrates to enantioenriched products^5^,^6^, this important field of synthetic methodology has neglected the development of enantiospecific diastereoselective transformations, where single enantiomer starting materials are subjected to asymmetric cycloisomerization to give specific diastereomer products (that is, double stereodifferentiation, where catalyst and substrate stereocontrol compete)^7^,^8^,^9^,^10^. In an age where absolute control of molecular substitution and stereochemistry is paramount for applications, the realization of such processes would represent a major advance in the sustainable synthesis of precision-manufactured target molecules.

Here we describe rationally designed cycloisomerization catalysts that address these challenges. The reaction selected for this study was the [5+2] cycloisomerization of ynamide-vinylcyclopropanes to [5.3.0] azabicycles (**1**→**2**, **3**, Fig. 1). Although [5+2] processes have a rich history in the field of alkyne-vinylcyclopropanes^11^,^12^,^13^,^14^,^15^,^16^,^17^,^18^,^19^,^20^,^21^,^22^, the development of this process using ynamides—or indeed any asymmetric cycloisomerization of ynamide-tethered enynes—has not been explored either experimentally or theoretically^23^,^24^,^25^,^26^,^27^. More generally, we question whether a catalyst system optimized to achieve an enantioselective cyclization (**1**→**2**, Fig. 1b) can translate to a double stereodifferentiating setting (**1**→**3** or ***epi-*****3**, Fig. 1c), particularly should the catalyst be required to overcome powerful substrate stereocontrol. Intrinsic to these studies is a combined theoretical and experimental approach to optimize catalyst design^28^,^29^,^30^, which in the event reveals an unexpected mechanistic pathway for rhodium-catalysed [5+2] carbocyclizations (Fig. 1a). This work demonstrates the powerful role of density functional level of theory (DFT) computations in understanding asymmetric catalysis, leading to quantitative computational-led design of new, highly selective ligands.

## Results

### Substrate synthesis and reaction optimization

**Figure 2:**
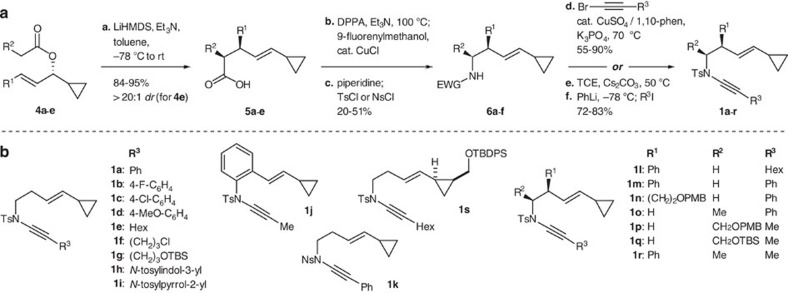
Synthesis of ynamide-vinylcyclopropanes 1. (**a**) The synthetic route employed uses an Ireland-Claisen rearrangement of esters **4a–e** to construct the vinylcyclopropane and install up to two backbone substituents; subsequent Curtius rearrangement/sulfonylation converts the carboxylic acids **5** to sulfonamides **6**; ynamide formation is then achieved using copper-catalysed coupling with a bromoalkyne or, for hindered or aniline-derived ynamides, a two step route via a dichloroenamide. For the preparation of enantioenriched esters **4b**, **4d** and **4e**, and ynamides **1q** and **1s**, see Supplementary Fig. 4. (**b**) Ynamide-vinylcyclopropanes prepared using this strategy.

A selection of ynamide cycloisomerization substrates **1** were readily accessed from allylic esters **4** (Fig. 2) by an Ireland-Claisen/Curtius rearrangement/ynamide formation sequence. Substituents could be introduced at one or both of the carbon atoms on the ene-ynamide backbone, and by incorporating an enzymatic resolution into this synthesis, enantioenriched ynamides could be prepared. Ynamide formation (**6**→**1**) was achieved using copper-catalysed coupling of the sulfonamide with a bromoalkyne^32^, or via formation of an intermediate dichloroenamide^33^. Initial screening of ruthenium^14^ and rhodium^20^,^34^ catalyst systems (Table 1) revealed that only the latter afforded high yields of azabicycle **7a** from ynamide **1a**; using [Rh(cod)naphthalene]SbF_6_ (5 mol%) as the catalyst^35^,^36^, the cycloisomerization could be effected within 3 h at room temperature, giving **7a** in 91% yield (Entry 6).

### Substrate scope

**Figure 3:**
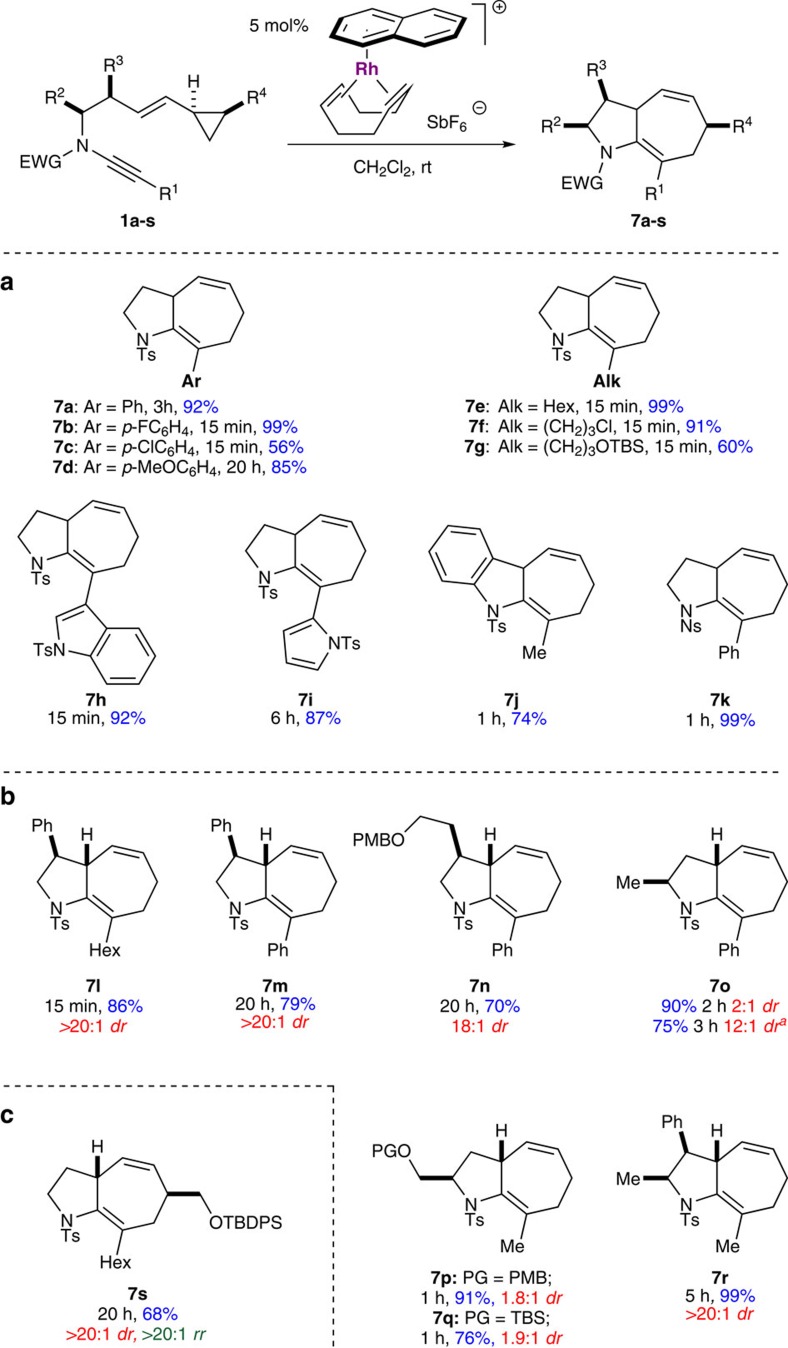
Rhodium-catalysed ynamide [5+2] cycloisomerization. (**a**) Ynamide substituent scope. (**b**) Diastereoselective cycloisomerization proceeds with high levels of substrate stereoinduction. (**c**) High regioselectivity and diastereoselectivity is observed with a substituted cyclopropane. ^*a*^Reaction performed using [Rh(cod)Cl]_2_ (10 mol%), toluene, 110 °C.

A variety of ynamides **1a**–**s** were now examined in the cycloisomerization using these optimized conditions (Fig. 3). Aryl-substituted ynamides **1b**–**d** reacted with high efficiency, and revealed a clear electronic effect on the reaction rate, with electron-deficient ynamides **1b** and **1c** showing reduced reaction times. Alkyl-substituted ynamides **1e**–**g** also displayed enhanced reactivity compared with phenyl ynamide **1a**, affording the corresponding [5.3.0]-azabicycles **7e**–**g** in excellent yields within 15 min at ambient temperature. Similar efficient reactivity was observed for the cyclization of the heteroaryl-substituted ynamides **1h** and **1i** to the indolyl- and pyrrolyl-substituted products **7h** and **7i**. The mild conditions of the reaction are emphasized by the cyclization of the aniline-derived ynamide **1j**, which led to tricycle **7j** in 74% yield—notably, this 1,4-diene did not undergo *in situ* isomerization to the indole.

Of clear interest was the level of substrate stereocontrol that might be achieved using ynamides **1l**–**s**. Excellent levels of substrate stereoinduction were observed, with products **7l**–**s** afforded in high yield and as single diastereomers for substrates featuring an allylic stereocenter; and, for **7s**, as a single regioisomer as well as diastereoisomer^14^. Cyclization of substrates containing homoallylic stereocenters proved less selective; however, good stereoselectivity (*dr*=12:1) could be achieved using [Rh(cod)Cl]_2_. These collected results are significant in the wider context of [5+2] cycloisomerization, where high levels of substrate stereocontrol have previously been generally interpreted to arise from an `inside alkoxy effect' from oxygen-bearing stereocenters allylic to the vinylcyclopropane^37^,^38^. In this work, it is apparent that such stereoelectronic effects are not a prerequisite for high stereoselectivity.

### Enantioselective cycloisomerization

**Figure 4:**
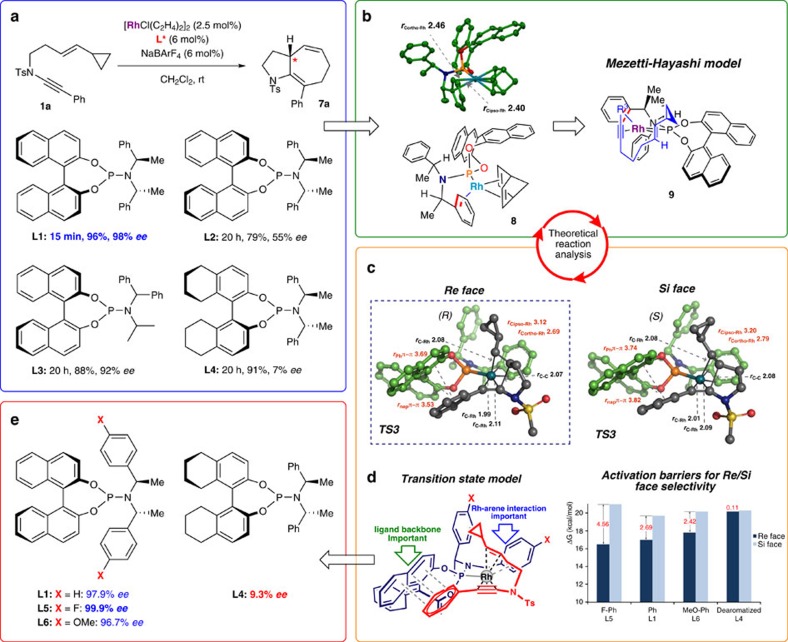
Exploration of asymmetric ynamide [5+2] cycloisomerization inspires a computation-guided ligand design. (**a**) A selection of phosphoramidite ligands screened reveals a strong dependence of enantioselectivity and reaction rate on amine and BINOL components' structure and chirality, with **L1** giving superior results. (**b**) The Shintani–Hayashi model for enantioselectivity is based on a crystal structure of [**L1** Rh(norbornadiene)]^+^. (**c**) Our calculations (Fig. 5) support an alternative mode of binding of the ynamide, and an alternative reaction pathway that initiates with rhodacyclopentene formation. *Re* face binding of the vinylcyclopropane is favoured, with calculated structures, and activation barriers (kcal mol^−1^)/selectivities depicted (distances in Å). (**d**) A transition state model is shown that identifies key ligand design parameters. (**e**) Variation of the electronic character of the η^2^-bound arene of the phosphoramidite leads to predicted enantioselectivies for **L5** (*p*-F) and **L6** (*p*-OMe), and an excellent correlation of experiment and theory for **L1** and **L4**.

In targeting an asymmetric version of the cyclization, we were mindful of the excellent asymmetric [5+2] Rh-catalysed cycloisomerization of alkyne-vinylcyclopropanes described by Shintani *et al*.^39^,^40^, which employed the versatile phosphoramidite ligand **L1** (Fig. 4a)^41^. A preliminary screen of a range of phosphoramidite ligands **L1**–**L4** revealed that **L1** indeed seemed optimal for the enantioselective cycloisomerization of ynamide-vinylcyclopropane **1a**, delivering product (–)-**7a** in excellent yield and enantioselectivity (96%, 98% *ee*) after just 15 min at room temperature (see Supplementary Information for assignment of absolute stereochemistry). However, forays with other substrates suggested that this ligand might not meet our expectations in more challenging settings, and we realized that any further advance would require a combined theoretical and experimental design approach.

To this end, we conducted a computational exploration of the reaction pathway, which began with investigation of the empirical model adopted by Shintani *et al*. to explain the enantioselectivity of vinylcyclopropane-alkyne cycloisomerization. This model (Fig. 4b) is based on an X-ray crystal structure of [Rh(**L1**) norbornadiene]BF_4_ reported by Mezetti (**8**) (ref. 42), which reveals an η^2^-complexation of one of the α-methylbenzylamine arenes to the rhodium cation—the phosphoramidite thus acting as a bidentate ligand. The Shintani–Hayashi model docks the vinylcyclopropane-alkyne onto this ligated rhodium framework as in structure **9**, such that the alkyne coordinates *trans* to the phosphorous atom of the phosphoramidite ligand, and the vinylcyclopropane coordinates *trans* to the η^2^-complexed arene, with an orientation that minimizes steric interactions between the cyclopropane ring and naphthyl group.

### Computational study and ligand optimization

**Figure 5:**
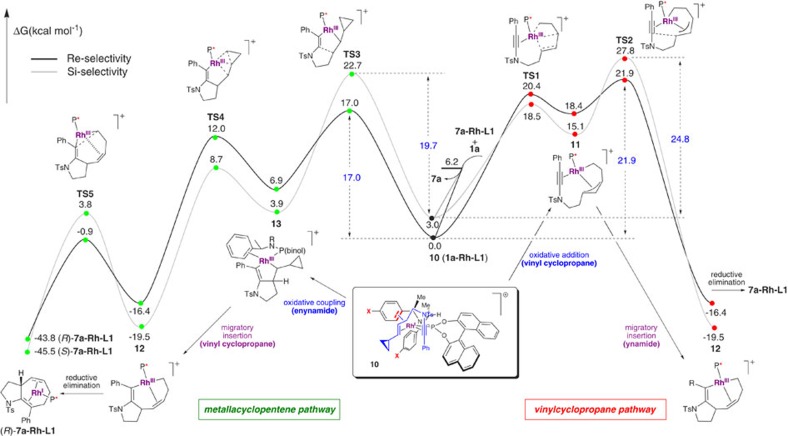
Theoretical reaction analysis. Complex **10** (*Re* face binding) is found to be the lowest energy ground state for ynamide complexation, and transition state for oxidative coupling (metallacyclopentene pathway, see Table 2). The calculated energy profiles of the cycloisomerization for Re-face (black, bold) and Si-face (grey) substrate association via pathways initiating with metallacyclopentene formation (left), or vinylcyclopropane insertion (right) are illustrated; the former is favoured. SMD-ωB97XD/6-311+G(d,p)/Lanl2TZ//ωB97XD/6-31G(d)/Lanl2DZ Gibbs free energies are shown in kcal mol^−1^.

Computation, particularly at the DFT level, has emerged as a powerful tool for assessing the feasibility of mechanistic steps involved in catalysis^43^. Our theoretical work first explored eight possibilities for the binding of ynamide-vinylcyclopropane **1a** to the [Rh(**L1**)] cation (Table 2). In contrast to the Shintani–Hayashi model, this suggested that the lowest energy complex of [Rh(**L1**)**1a**] positions the ynamide proximal to the naphthyl ring and *trans*- to the arene ligand, and the vinylcyclopropane *trans*- to the phosphorous atom (**10**, Fig. 5, P-*trans*-ene/Up). The next lowest energy structure maintains this positional selectivity of substrate binding, but inverts its orientation (that is, P-*trans*-ene/Down). Next, calculations were carried out to explore the two widely accepted mechanisms for [5+2] cycloisomerization, which initiate either with an oxidative addition into the vinylcyclopropane, followed by alkyne insertion into the resultant σ/π-allyl rhodium(III) complex **11** to give eight-membered rhodacycle **12** (vinylcyclopropane pathway), or via oxidative cycloaddition of Rh(I) with the alkyne and alkene to give rhodacyclopentene **13**, followed by ring expansion into the cyclopropane to give the same intermediate **12** (metallacyclopentene pathway). Where investigations by Yu, Houk and Wender on Rh-catalysed [5+2] cycloisomerizations suggest that oxidative addition into the vinylcyclopropane is the first step on the catalytic pathway^17^,^21^, our DFT calculations (Fig. 5) suggest that oxidative coupling of the alkene-ynamide to form metallacyclopentene **13** appears to be the preferred reaction pathway for an ynamide-vinylcyclopropane to give **12**, followed by ring expansion of the cyclopropane. Notably, this mechanism has also been calculated to be the preferred pathway in ruthenium-catalysed [5+2] cycloisomerization^17^. The preference for this pathway over vinylcyclopropane oxidative addition is in the range of 4–12 kcal mol^−1^, depending on the orientation with which **1a** binds to the [Rh(**L1**)] cation; notably, the transition states for both pathways favour *Re*-face binding of the alkene in a P-*trans*-ene/Down orientation. This sequence of steps is favoured in this intramolecular reaction due to additional stabilization of the forming Rh(III) intermediate in the oxidative coupling step by the electron-rich ynamide. The free energy profile for the catalytic cycle is exergonic by more than 40 kcal mol^−1^, and product inhibition is predicted to be minimal, since the reactant preferentially binds to the catalyst by 6.2 kcal mol^−1^; taken together with a turnover and selectivity determining barrier of 17.0 kcal mol^−1^ for the metallacyclopentene pathway, our computations are consistent with the short reaction times observed at room temperature for the conversion of **1a**–**7a** using **L1**.

The lowest energy transition state for the oxidative coupling of this metallacyclopentene pathway (TS3) is illustrated in Fig. 4c. This transition state leads to a calculated enantioselectivity for cyclization with ligand **L1** of 97.9% *ee* (ΔΔG^‡^_Re/Si_=–2.69 kcal mol^−1^), which is in excellent agreement with the experimental value (*R*, 98% *ee*). The calculated enantioselectivity for phosphoramidite **L4** (9.3% *ee*, ΔΔG^‡^_Re/Si_=–0.11 kcal mol^−1^) also correlates well with the poor selectivity we had already observed experimentally with this ligand (7% *ee*), supporting the mechanistic model, and implying that the naphthyl ring plays a crucial role—likely related (for aryl ynamides) to a stabilizing dispersive (π−π) interaction between the ynamide substituent and the naphthyl group in TS3. Although partial saturation of the naphthyl backbone (that is, in **L4**) leads to higher activation barriers, it is notable that the erosion of enantioselectivity for this ligand results from a greater increase (by 2.58 kcal mol^−1^) to the activation barrier for *Re*-face addition compared with *Si*-face addition; this supports the existence, and importance, of stabilizing non-bonding (dispersion) interactions because of the aromatic backbone of **L1** in favouring the major enantiomer. Alkyl substituents are expected to experience similar attractive non-bonding interactions (CH–π); these interactions are further evident from an analysis of the computed non-covalent interaction index (Supplementary Fig. 68).

The oxidative coupling (TS3) is the rate-limiting and enantioselectivity determining step in this rhodacyclopentene mechanism. As observed in the Mezetti norbornene crystal structure (Fig. 4b)^42^, one of the ligand benzylamine groups acts as 2π-electron donor (2.6–2.7 Å) in these transition states. We hypothesized that variation of the electronic character of this η^2^-complexed arene could dramatically influence both the rate, and selectivity, of the reaction. Calculations on the effect of electronically-biasing methoxy and fluoro groups at the *para* position of these arenes suggested that decreasing the electron density on the arene (*p*-F, **L5**, Fig. 4d,e) would lead to an increase in reaction rate (ΔΔ*G*^‡^_**L5**/**L1**_=−0.47 kcal mol^−1^) and enantioselectivity (ΔΔ*G*^‡^_Re/Si_=–4.56 kcal mol^−1^, 99.9% *ee*), while an electron-donating substituent (*p*-MeO, **L6**) would have the opposite effect (ΔΔG^‡^_**L6**/**L1**_=+0.76 kcal mol^−1^, ΔΔ*G*^‡^_Re/Si_=–2.42 kcal mol^−1^, 96.7% *ee*). This computed trend in reactivity and enantioselectivity, namely **L5**>**L1**>**L6**, results from a weakened metal-arene interaction in the TS leading to the major enantiomer, which is compensated for through stronger coordination of the alkynyl and alkenyl groups (see Supplementary Information for further details and discussion). These ligand structural modifications also modulate the Lewis basicity at phosphorus, however preferential stabilization of the major enantiomeric pathway confirms that the predominant effect is not inductive in nature, but rather depends on through-space interactions involving the Rh-coordinating aryl group.

### Substrate scope in the asymmetric cycloisomerization

**Figure 6:**
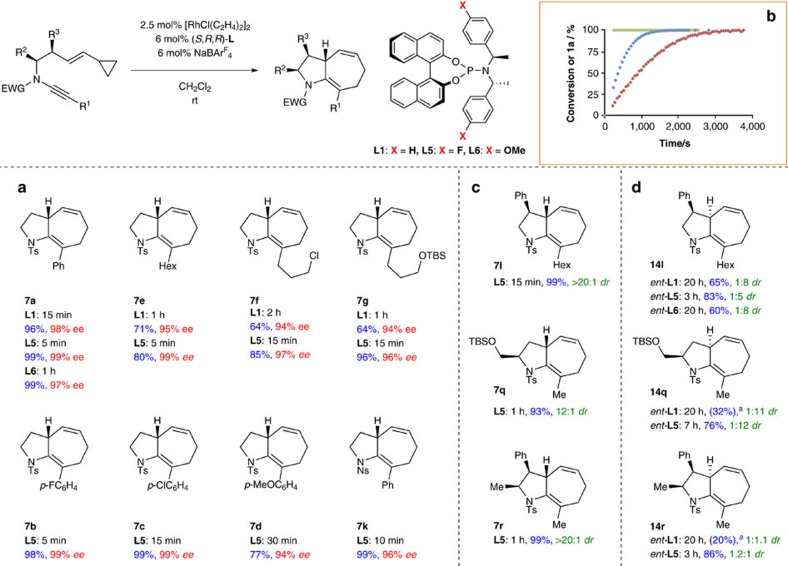
Enantioselective and double stereodifferentiating, ynamide [5+2] cycloisomerizations. (**a**) Enantioselective cyclization is tested against a range of ynamide-vinylcyclopropanes, showing an excellent correlation of computation and experiment for the theory-designed ligands **L5** and **L6**. The synthesis of **7e** was performed on 1 mmol scale (1.25 mol% catalyst). (**b**) ^1^H NMR spectroscopic monitoring of reaction progress emphasizes the rate enhancement between ligands **L6**, **L1** and **L5**, also predicted theoretically. (**c**) Matched double stereodifferentiating cycloisomerizations proceed with high selectivity and rate. (**d**) Mismatched double stereodifferentiating cycloisomerizations proceed successfully under catalyst stereocontrol using the enantiomers of ligands **L1**, **L5** and **L6** (that is, (*R,S,S*)-**L** stereochemistry); the major diastereomer is shown. The ^*a*^ signifies reaction conversion, as judged by ^1^H NMR spectroscopic analysis.

While phosphoramidite **L6** is known, (ref. 44) **L5** is not, but could be readily synthesized using standard methods^45^. Both were evaluated in the enantioselective cyclization alongside **L1** (Fig. 6a). To our delight, experiment correlated well with the predicted outcomes of these cyclizations; the *p*-fluorobenzyl ligand **L5** indeed showed a dramatic enhancement of rate and selectivity in the cyclization of **1a** compared with the parent ligand **L1**, affording **7a** in under 5 min with 99% *ee* (calc. 99.9% *ee*), while the *p*-methoxybenzyl ligand **L6** exhibited a much reduced rate of reaction, and also lower enantioselectivity (1 h, 97% *ee*, calc. 96.7% *ee*). The cyclization of alkyl-substituted ynamides **1e**–**g** to give enantioenriched azabicycles **7e**–**g** also showed a rate and selectivity enhancement between **L1** and **L5**. The potent reactivity of the latter ligand was extended to a variety of aryl-substituted ynamides, where the marked rate difference between electron-poor (**1b**) and electron-rich (**1d**) ynamides supports the hypothesis that the ynamide is intimately involved in the rate-determining step (that is, a metallacyclopentene pathway). In all cases using ligand **L5**, these reactions proceeded in under 30 min with excellent levels of enantioselectivity (94–99% *ee*); furthermore, the synthesis of **7e** could be achieved on a 1 mmol scale in less than 5 min with 1.25 mol% catalyst loading. Monitoring of reaction conversion by ^1^H NMR spectroscopy (in CDCl_3_, Fig. 6b) emphasizes the dramatic rate difference between these three ligands, with the reaction using **L5** complete in under 4 min, compared with the slower cyclizations with **L1** and **L6** (addition of the catalyst solution to the substrate followed by NMR spectroscopic analysis necessitated a four minute delay between reaction initiation and acquisition of the first NMR spectrum. The reaction catalysed by [L5Rh] was complete by this time).

Finally, the performance of these ligands was tested against the challenge of cyclizations that proceed with high levels of substrate stereocontrol, to address the question of how a high enantioselectivity-inducing catalyst would cope with mismatched substrate-catalyst diastereoselective cycloisomerization scenarios. Three substrates were chosen for this challenge: ynamides **1l**, **1q** and **1r**, which gave >20:1, 1.8:1 and >20:1 *dr*, respectively when cyclized using [Rh(cod)naphthalene]SbF_6_ (Fig. 3). As expected, these single enantiomer substrates cyclized rapidly, and with high efficiency and selectivity, with the matched substrate-catalyst combination (that is, (*S,R,R*)-**L5**, Fig. 6c). To our delight, we found that cycloisomerization of substrate **1l** using the enantiomeric catalyst system ((*R,S,S*)-**L1**) successfully overturned this powerful substrate stereoselectivity, giving **14l** in up to 1:8 *dr*. Interestingly, it was ligand **L1**—and not **L5**—which performed optimally in this challenging situation, albeit requiring an extended reaction time. In the case of **1q**, the catalyst (with either **L1** or **L5**) was able to achieve an equivalent (reversed) level of selectivity to that achieved in the matched sense—in this instance, a modest level of inherent substrate stereocontrol being completely overturned. Finally, the most challenging setting of the reinforcing substituents in **1r** proved a hurdle that could also be partly overcome, again demonstrating significant catalyst influence.

These observations may suggest that tighter substrate-Rh complexation in the case of **L5** (a consequence of a slightly weaker ligand-metal interaction observed in our calculations (see Supplementary Information for details), which improves rate and enantioselectivity), enhances unfavourable (that is, mismatched) steric effects in a double stereodifferentiating setting such that it effectively increases the stereocontrolling influence of the substrate relative to that of the ligand. The most reactive/selective catalysts for enantioselective cyclizations could thus suffer from higher than expected transition state energies in diastereoselective cyclizations, where such steric effects are enhanced compared with `less enantioselective' catalysts (looser substrate binding); and that different considerations are therefore needed in the development of double stereodifferentiating reactions, with more ‘promiscuous' catalysts potentially giving superior selectivity.

## Discussion

In summary, the first example of an enantioselective ynamide-tethered cycloisomerization has been achieved, with a series of highly enantio- and diastereoselective cyclizations giving a range of substituted/enantioenriched [5.3.0] azabicycles. Theoretical reaction analysis crucially influenced ligand design, leading to a catalyst system that displayed enhanced rate and enantioselectivity in the cycloisomerization. The demonstration of the first successful examples of enantiospecific diastereoselective transition metal-catalysed cycloisomerizations in a significantly mismatched substrate-catalyst environment illustrates that cycloisomerization can assemble functionalized ring systems with tuneable selectivity. These studies set the stage for the development of further computationally guided catalyst systems.

## Methods

### Racemic [5+2] cycloisomerization

To an oven-dried vial containing the ynamide vinylcyclopropane (1.0 equiv.) under Ar was added a solution of [(C_10_H_8_)Rh(cod)]SbF_6_ (5 mol%) in degassed CH_2_Cl_2_ (10 ml mmol^−1^ of ynamide). The reaction mixture was stirred at room temperature under Ar until consumption of the ynamide was observed by thin layer chromatography (see Fig. 3 for reaction times). The reaction mixture was then concentrated, and the resulting material was purified by flash chromatography (SiO_2_, petroleum ether/ethyl acetate eluent) (Fig. 3).

### Asymmetric [5+2] cycloisomerization

A solution of [RhCl(C_2_H_4_)_2_]_2_ (2.5 mol%), NaBAr^F^_4_ (6 mol%) and phosphoramidite ligand (6 mol%) in degassed CH_2_Cl_2_ (10 ml mmol^−1^ of ynamide) was stirred for 20 min under Ar. The solution was filtered (through a PTFE filter-tipped syringe) into an oven-dried vial containing ynamide vinylcyclopropane (1.0 equiv.) under Ar. The reaction mixture was stirred at room temperature under Ar until consumption of the ynamide was observed by thin layer chromatography (see Fig. 6 for reaction times). The reaction mixture was then concentrated, and the resulting material was purified by flash chromatography (SiO_2_, petroleum ether/ethyl acetate eluent) (Fig. 6).

### Computational methods

Molecular geometries were fully optimized at the DFT theory level in Gaussian 09 (rev. D.01), using the dispersion-corrected ω-B97XD functional^46^ without symmetry constraints. The effective core potentials (ECPs) of Hay and Wadt^47^ with a double-ζ basis set (LanL2DZ) were used for Rh, S and P, and the 6-31G(d) basis set was used for H, C, N and O(BS1). The energies were further estimated using a larger basis set (6-311+G (d, p) basis set for H, C, N, O, S and P) and triple-ζ basis set (LanL2TZ)^48^ for Rh (BS2) by single-point calculations, in implicit solvent CH_2_Cl_2_ treated with the SMD universal solvation models^49^. The structures of the ynamide substrate and a series of phosphoramidite ligands were computed in full, while the toluenesulfonamide *p*-tolyl group was modelled as a methyl group in the interests of computational tractability. See Supplementary Methods for further details.

## Additional information

**How to cite this article:** Straker, R. N. *et al*. Computational ligand design in enantio- and diastereoselective ynamide [5+2] cycloisomerization. *Nat. Commun.* 7:10109 doi: 10.1038/ncomms10109 (2016).

## Supplementary Material

Supplementary Information Supplementary Figures 1-68, Supplementary Tables 1-2, Supplementary Discussion, Supplementary Methods and Supplementary References

Supplementary Data 1 xyz coordinates for computational modelling

## Figures and Tables

**Figure 1:**
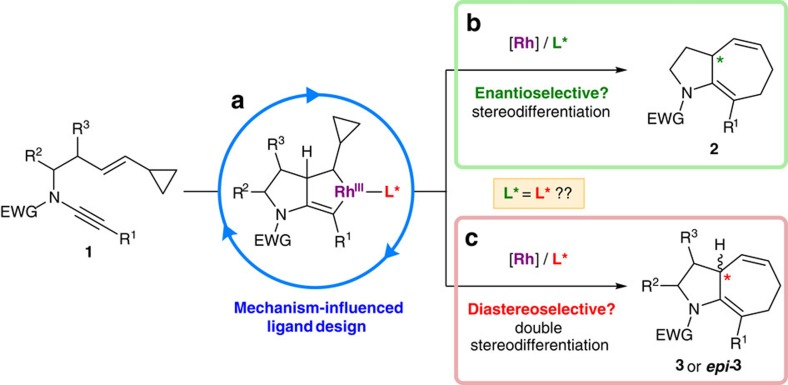
A reaction blueprint for enantioselective and/or double stereodifferentiating diastereoselective ynamide-vinylcyclopropane cycloisomerization. (**a**) We show that mechanistic calculations are crucial in the design of an enantioselective cycloisomerization catalyst. (**b**) Enantioselective higher order ynamide cycloisomerization is realized. (**c**) Despite high levels of substrate stereocontrol, the computationally designed catalyst is able to define product stereochemistry.

**Table 1:** Optimization of the [5+2] cycloisomerization reaction.

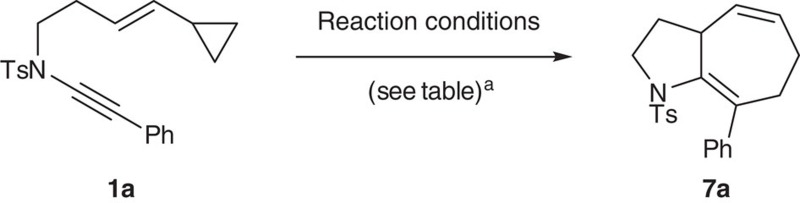					
Entry	Catalyst (mol%)	Solvent	Temp. (°C)	Time (h)	Yield (%)
1	[CpRu(MeCN)_3_]PF_6_ (10)	acetone	50	20	– ^b^
2	[RhCl(cod)]_2_ (10)	toluene	110	20	– ^b^
3	RhCl(PPh_3_)_3_ (10)	toluene	110	1	72
4	[(C_10_H_8_)Rh(cod)]SbF_6_ (10)	1,2-dichloroethane	rt	1.5	92
5	[(C_10_H_8_)Rh(cod)]SbF_6_ (10)	CH_2_Cl_2_	rt	1.5	94
6	[(C_10_H_8_)Rh(cod)]SbF_6_ (5)	CH_2_Cl_2_	rt	3	91

^a^Reactions performed at 0.1 M substrate in the solvent stated. ^b^Reaction gave a complex mixture of products.

**Table 2:** Eight possible orientations of enynamide docking onto the L1-Rh cation are explored.

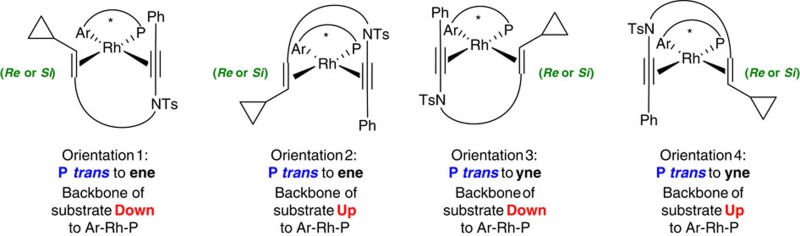				
Orientation	Metallacyclopentene Pathway		Vinylcyclopropane pathway	
	*Re-*selectivity (*R*)	*Si-*selectivity (*S*)	*Re-*selectivity (*R*)	*Si-*selectivity (*S*)
1	18.7	31.3	23.0	22.0
2	**17.0**	19.7	21.9	24.8
3	23.6	23.9	29.3	24.5
4	23.2	30.7	35.3	41.7

Transition state energies (SMD-ωB97XD/6-311+G(d,p)/Lanl2TZ//ωB97XD/6-31G(d)/Lanl2DZ Gibbs free energies, shown in kcal mol^−1^) associated with each mode of binding are tabulated.
