# Supplementary material for: Computational ligand design in enantio- and diastereoselective ynamide [5+2] cycloisomerization
Source: Nat Commun. 2016 Jan 5;7:10109. doi: 10.1038/ncomms10109 (PMC4728367; doi:10.1038/ncomms10109)
Supplement: Supplementary Information — Supplementary Figures 1-68, Supplementary Tables 1-2, Supplementary Discussion, Supplementary Methods and Supplementary References [file ncomms10109-s1.pdf]

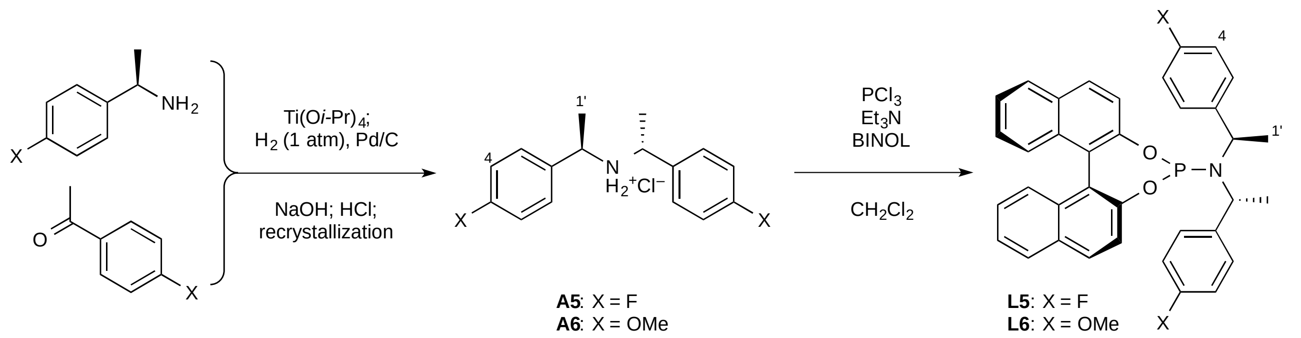

Supplementary Figure 1. Synthesis of phosphoramidites **L5** and **L67**

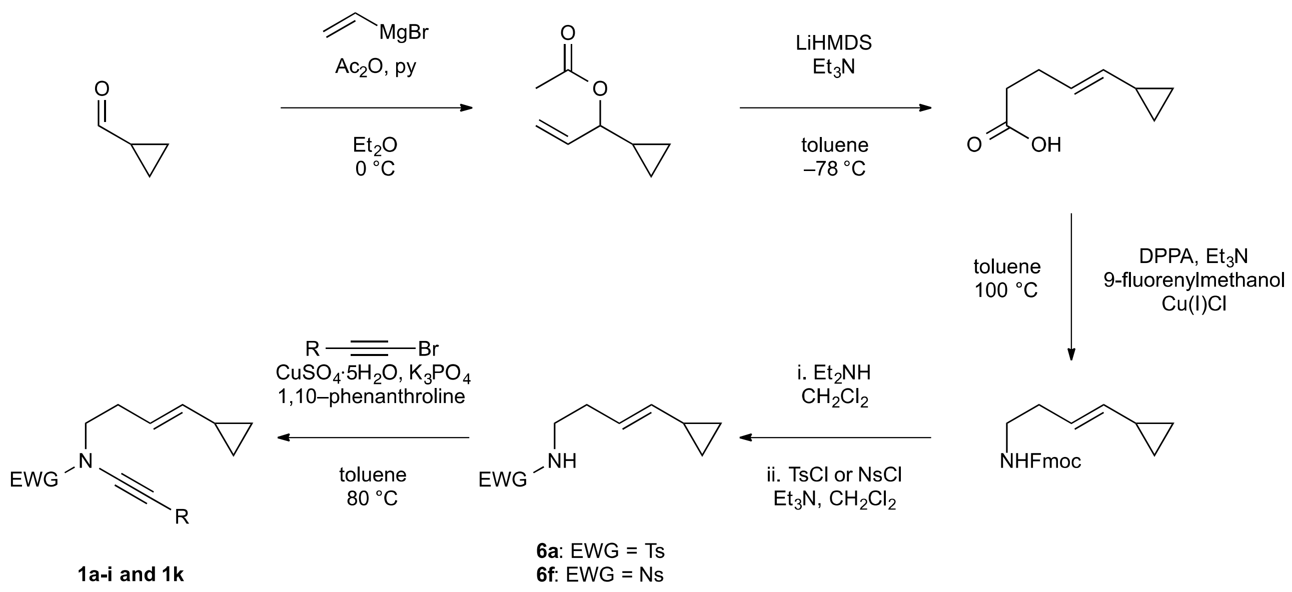

Supplementary Figure 2. Synthesis of ynamide vinylcyclopropanes **1a-i and 1k**

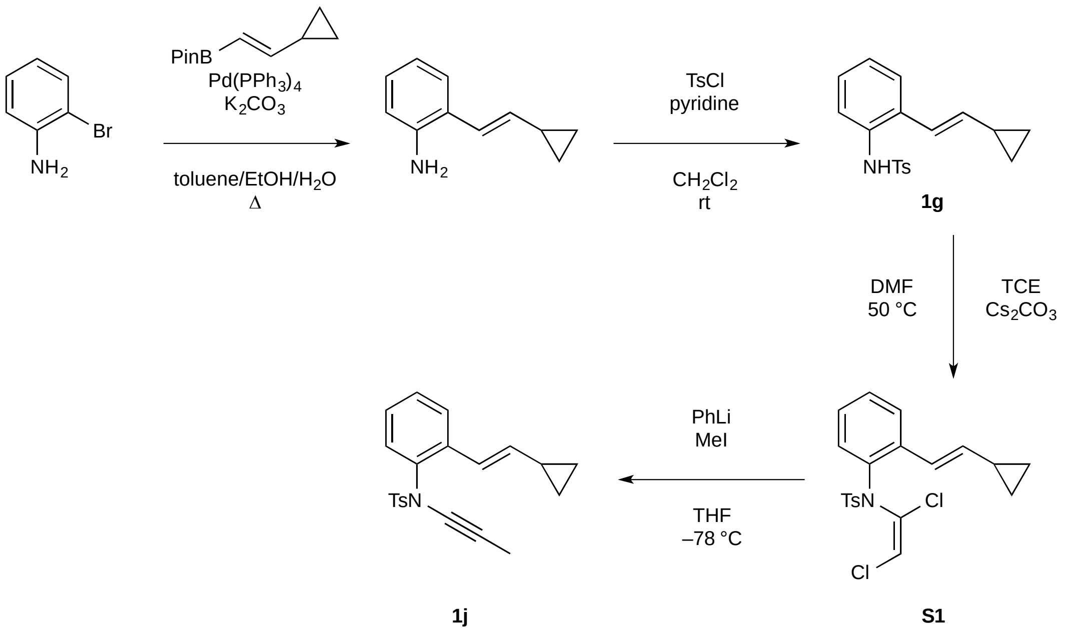

Supplementary Figure 3. Synthesis of aryl-tethered ynamide vinyl-cyclopropane **1j**

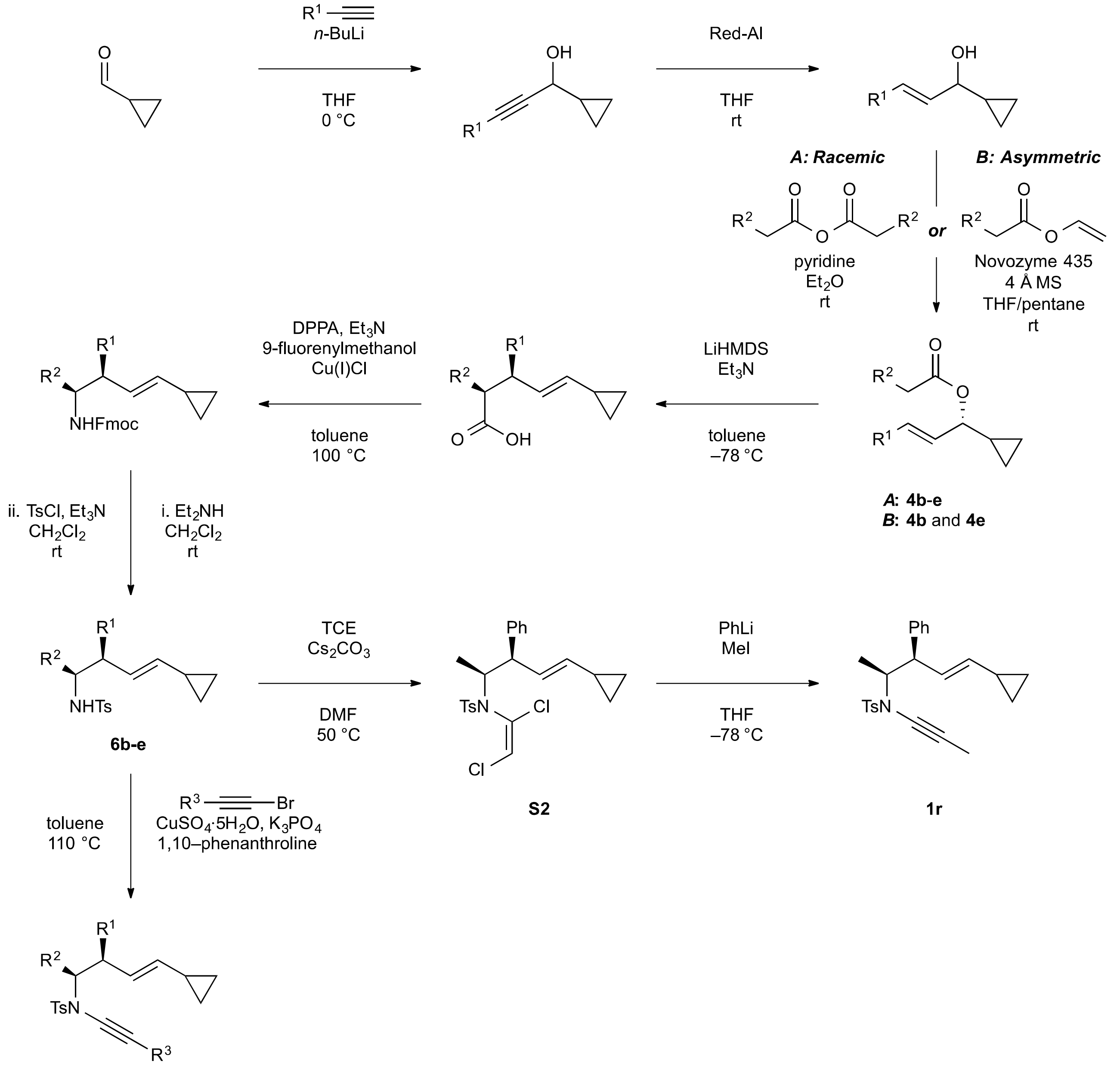

Supplementary Figure 4. Synthesis of substituted-tether ynamide vinyl-cyclopropanes **1l-o and 1r**

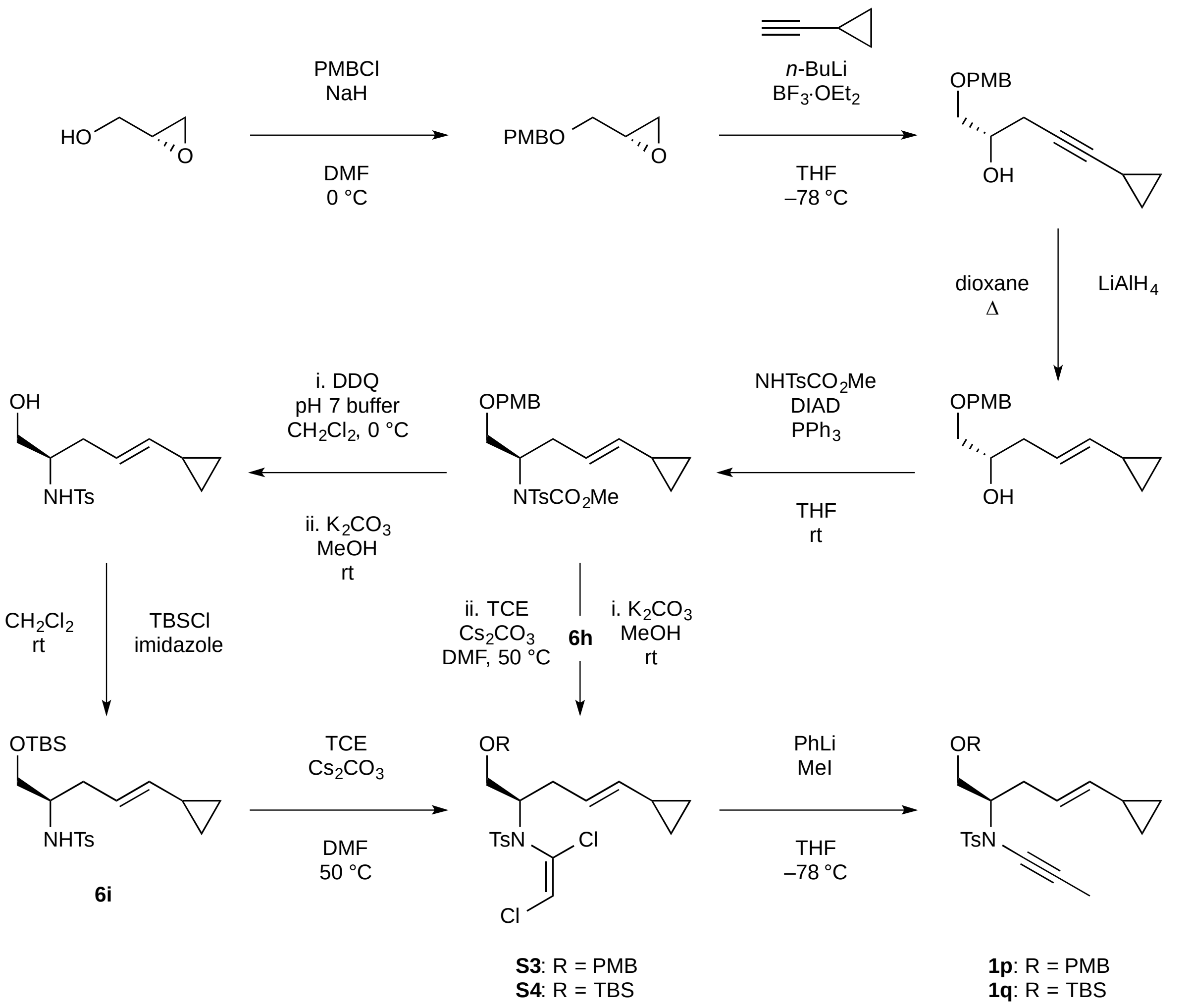

Supplementary Figure 5. Synthesis of substituted-tether ynamide vinylcyclopropanes **1p** and **1q**

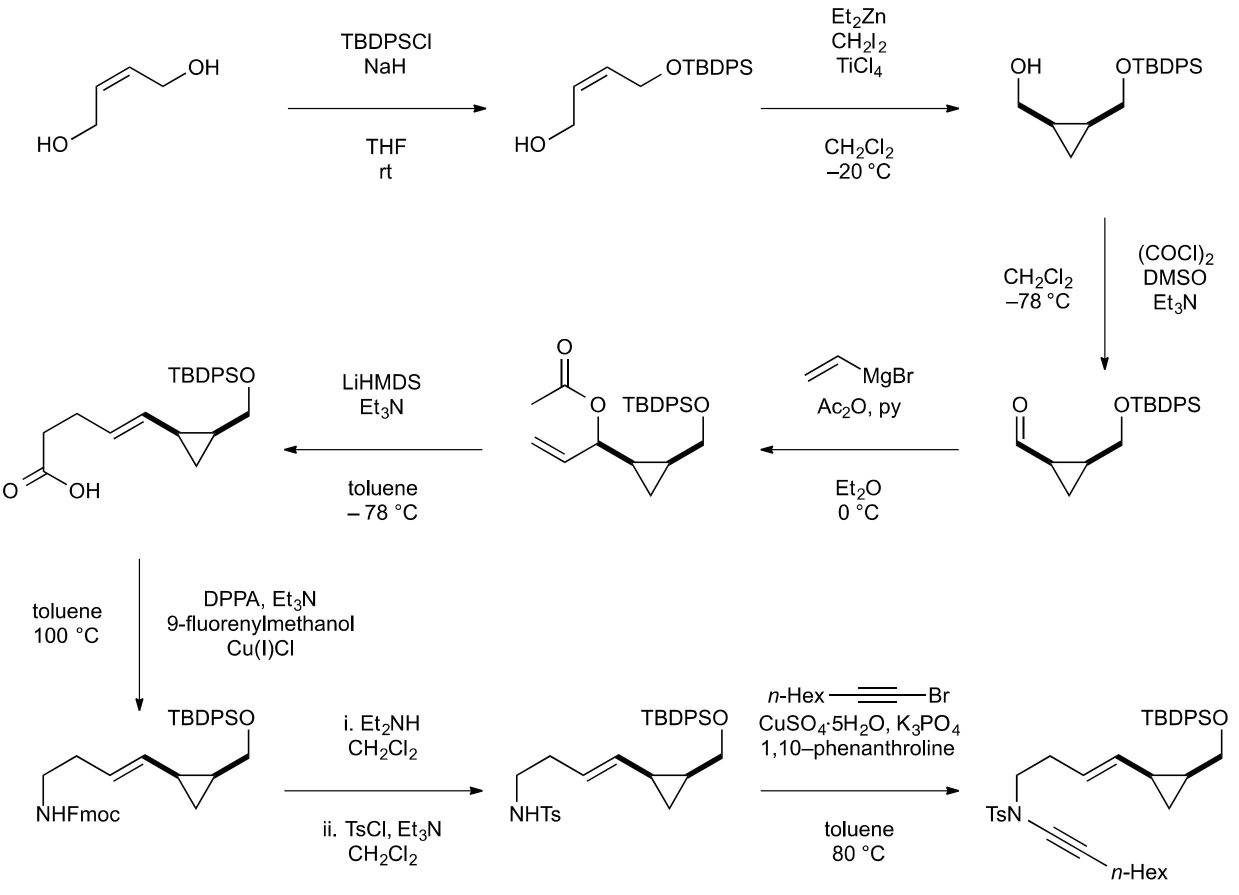

6j **1s**

Supplementary Figure 6. Synthesis of ynamide vinylcyclopropane **1s**

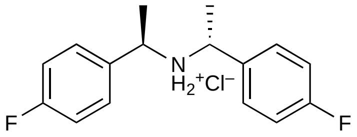

^{1}H NMR (400 MHz, $CDCl_3$)

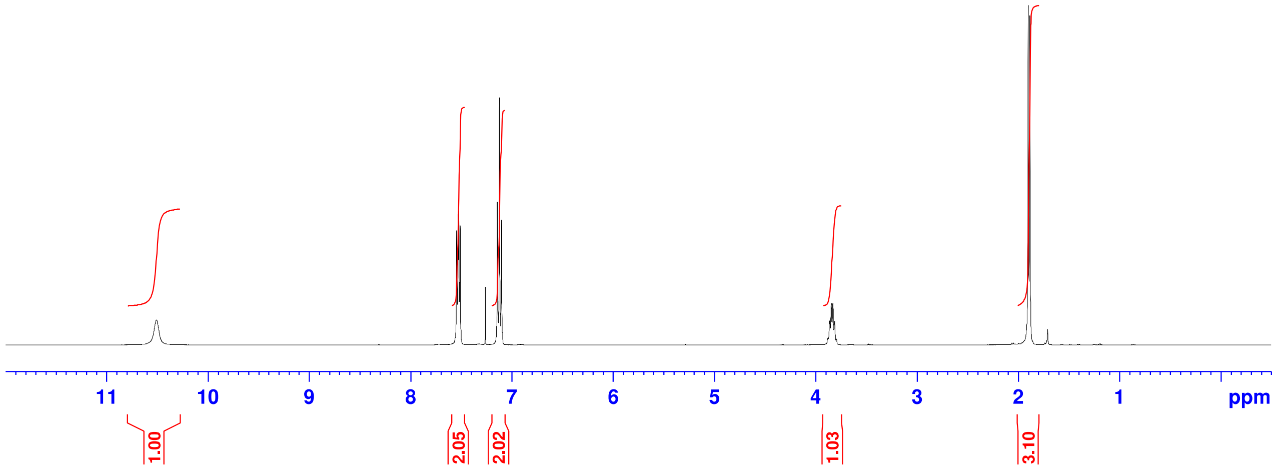

^{13}C NMR (100 MHz, $CDCl_3$)

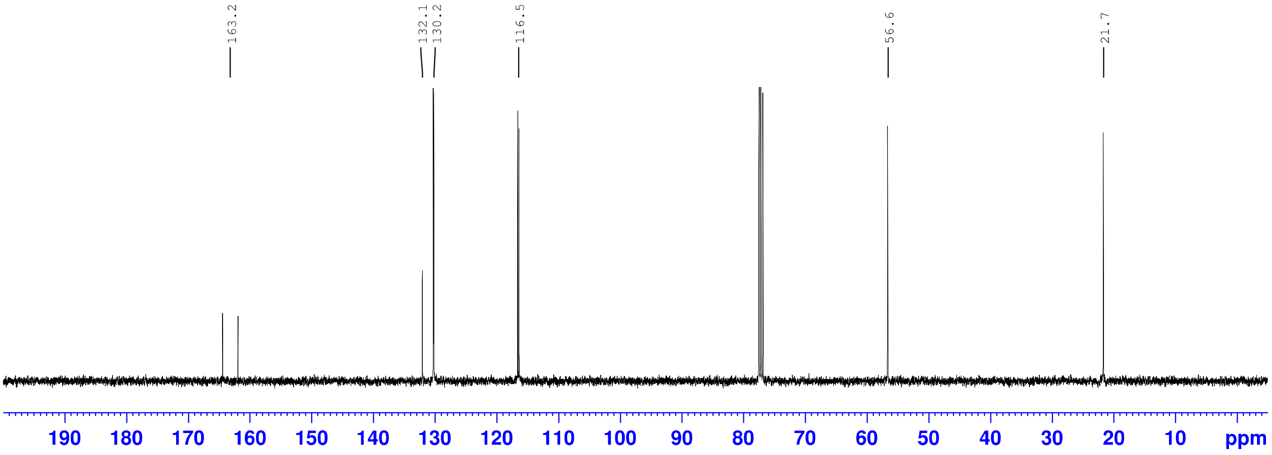

Supplementary Figure 7. ^{1}H and ^{13}C NMR spectra for compound **A5**

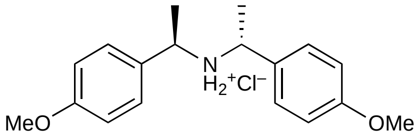

^{1}H NMR (400 MHz, $CDCl_3$)

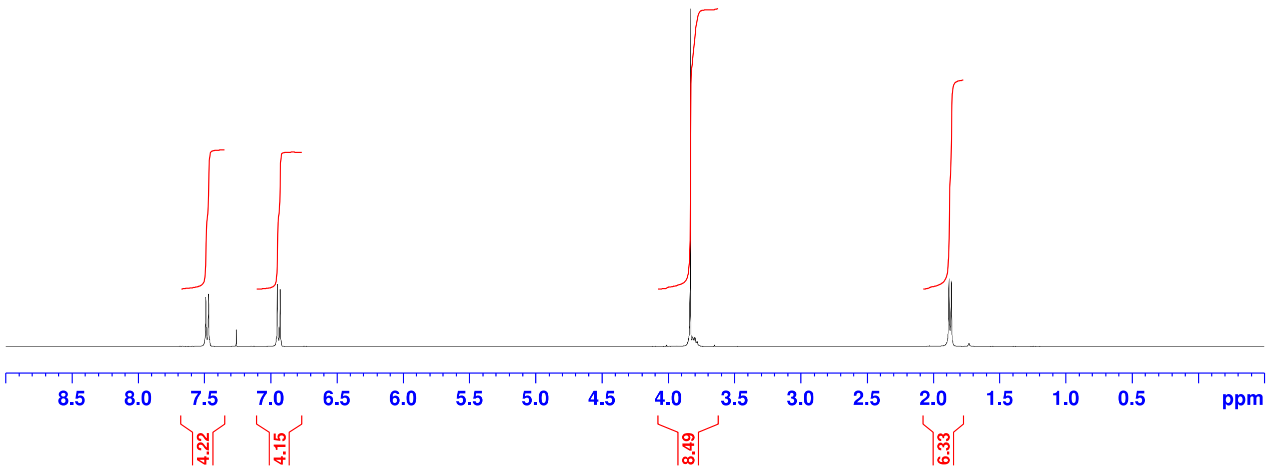

^{13}C NMR (100 MHz, $CDCl_3$)

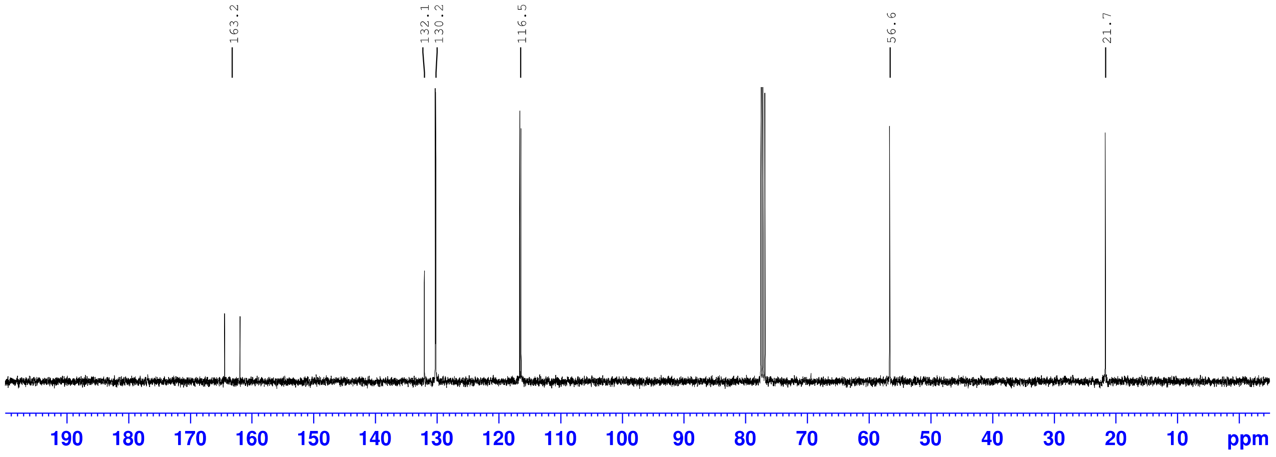

Supplementary Figure 8. ^{1}H and ^{13}C NMR spectra for compound **A6**

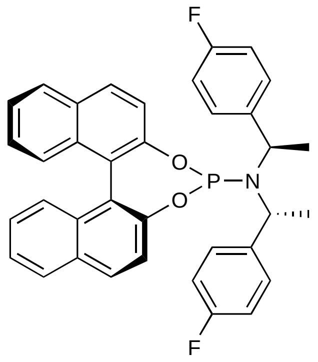

^{1}H NMR (500 MHz, $CDCl_3$)

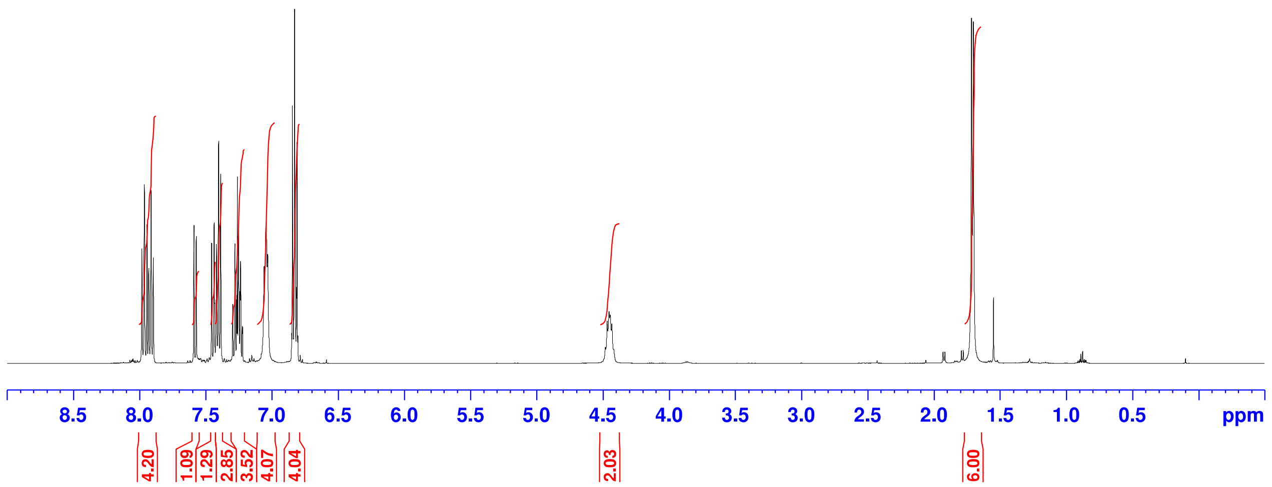

^{13}C NMR (125 MHz, $CDCl_3$)

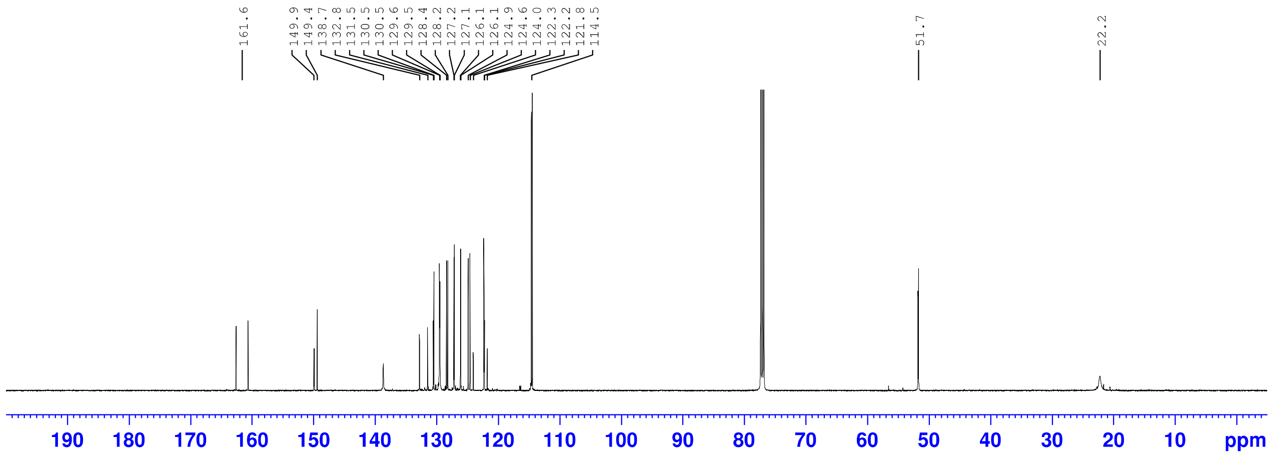

Supplementary Figure 9. ^{1}H and ^{13}C NMR spectra for compound **L5**

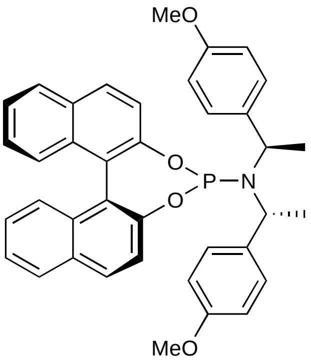

^{1}H NMR (500 MHz, $CDCl_3$)

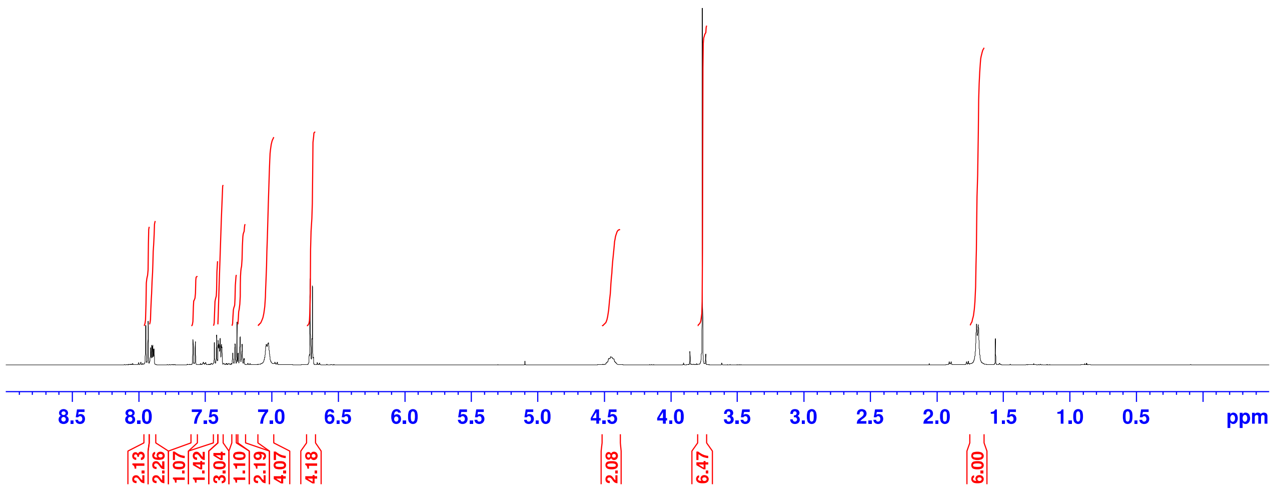

^{13}C NMR (125 MHz, $CDCl_3$)

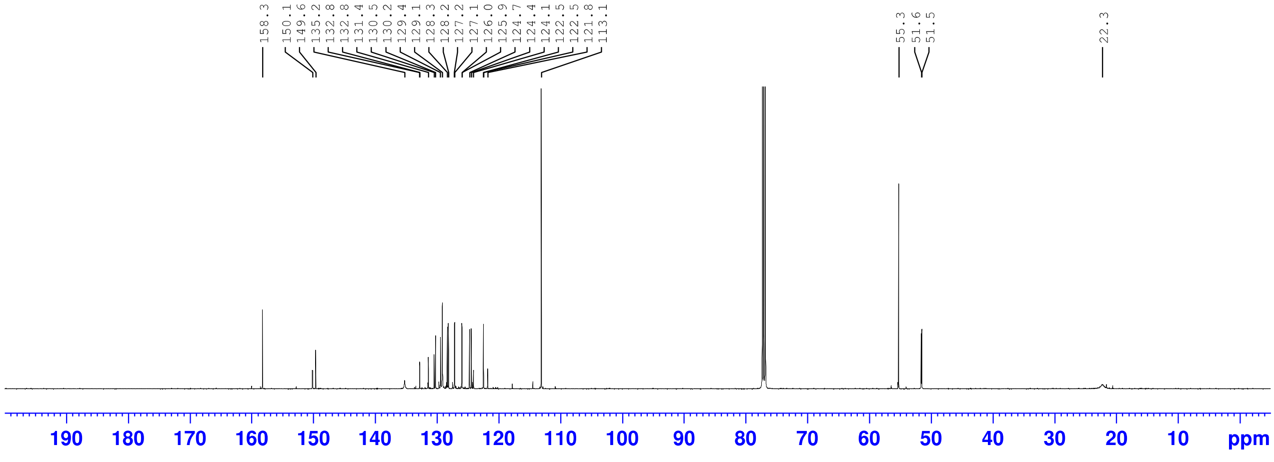

Supplementary Figure 10. ^{1}H and ^{13}C NMR spectra for compound **L6**

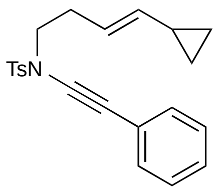

^{1}H NMR (500 MHz, $CDCl_3$)

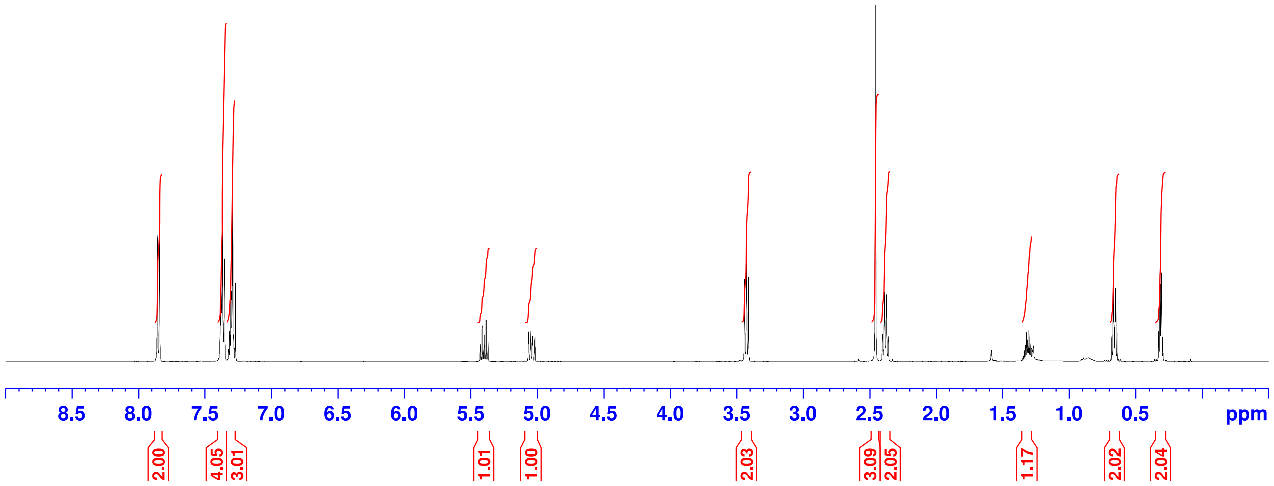

^{13}C NMR (125 MHz, $CDCl_3$)

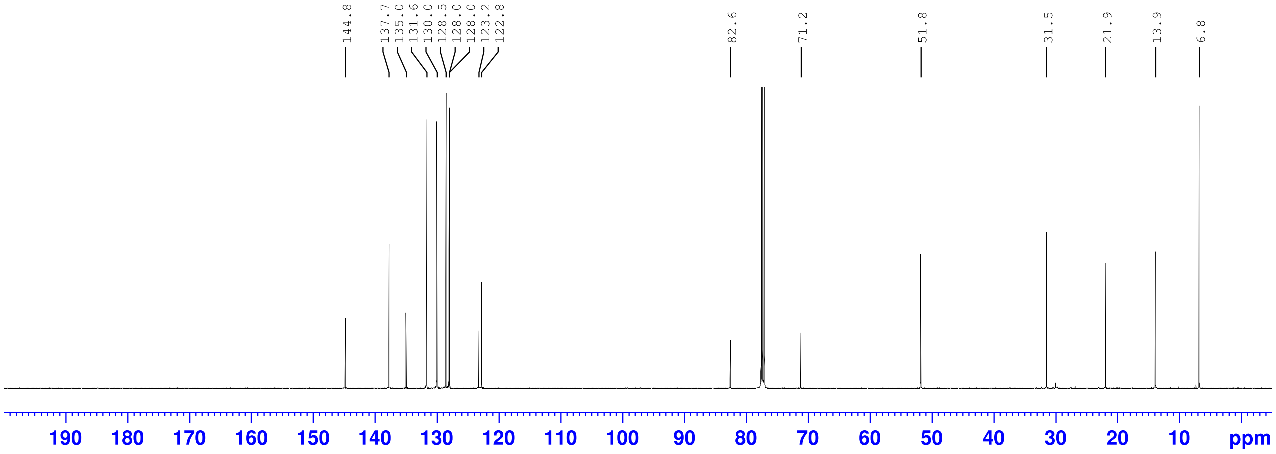

Supplementary Figure 11. ^{1}H and ^{13}C NMR spectra for compound **1a**

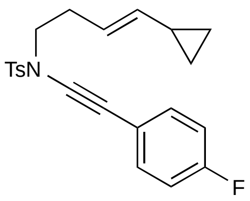

^{1}H NMR (500 MHz, $CDCl_3$)

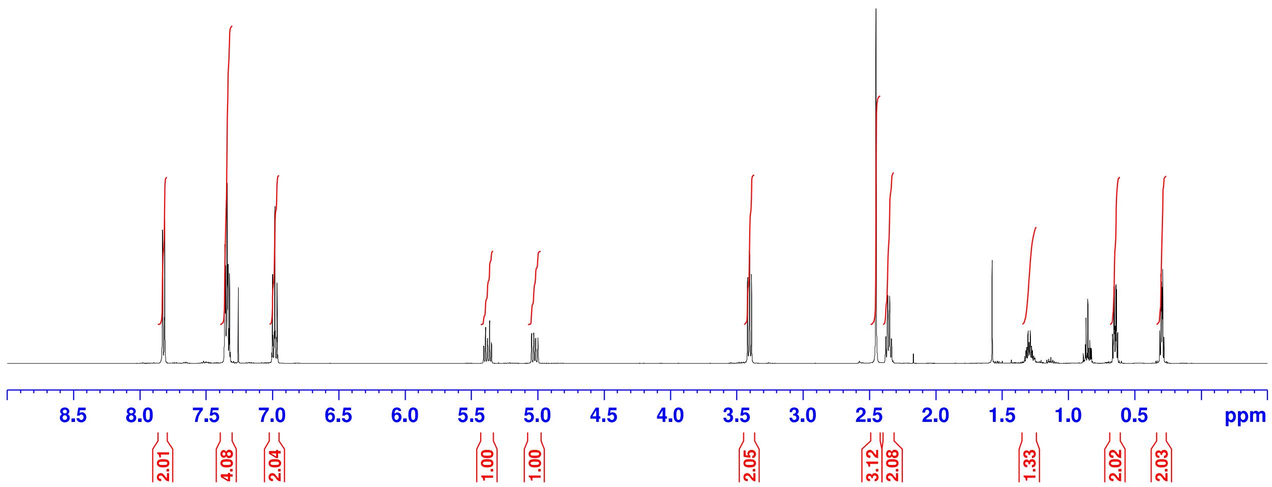

^{13}C NMR (125 MHz, $CDCl_3$)

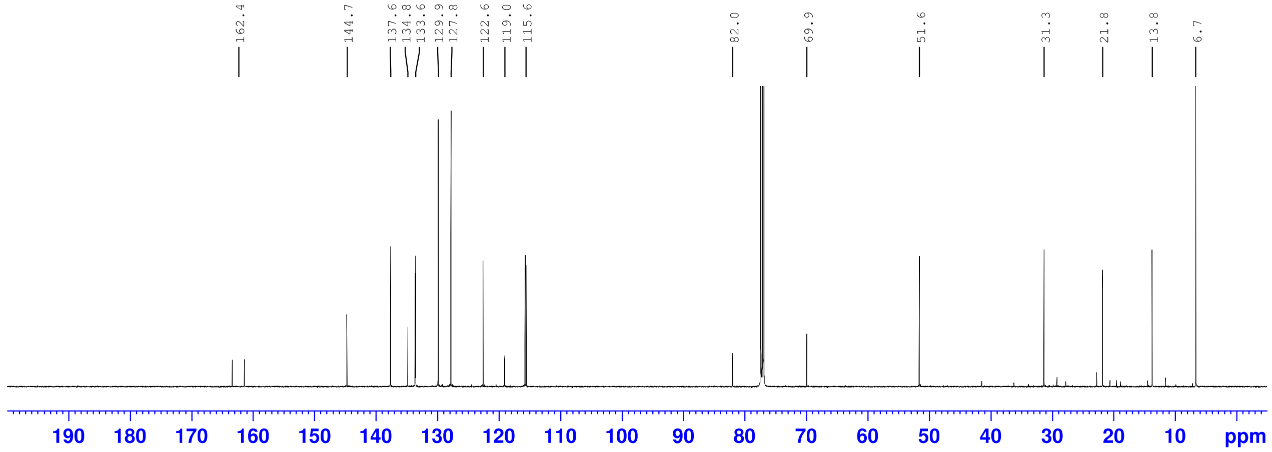

Supplementary Figure 12. ^{1}H and ^{13}C NMR spectra for compound **1b**

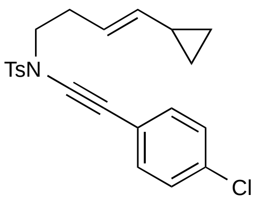

^{1}H NMR (500 MHz, $CDCl_3$)

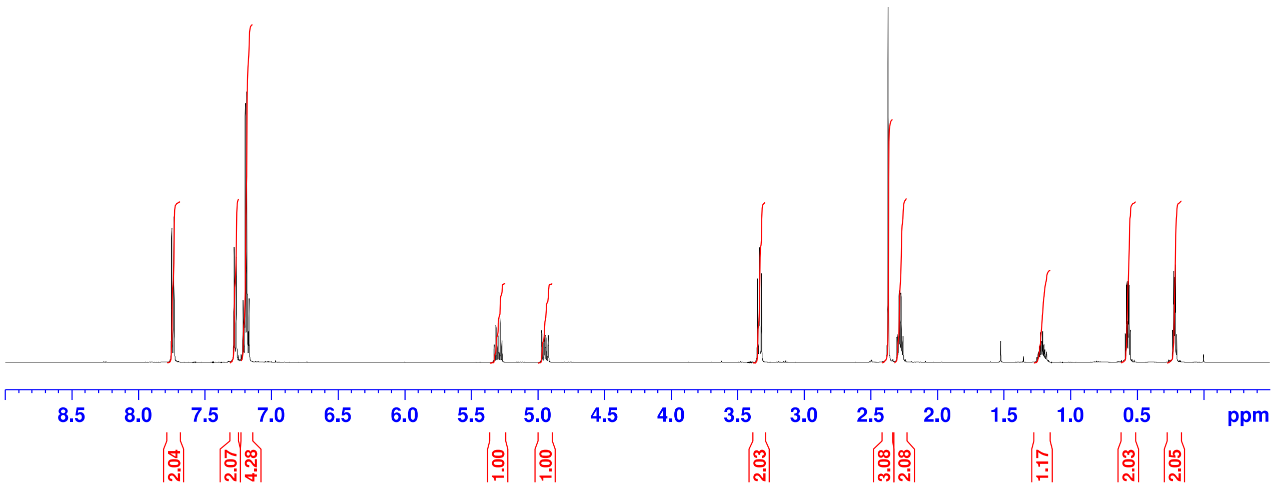

^{13}C NMR (125 MHz, $CDCl_3$)

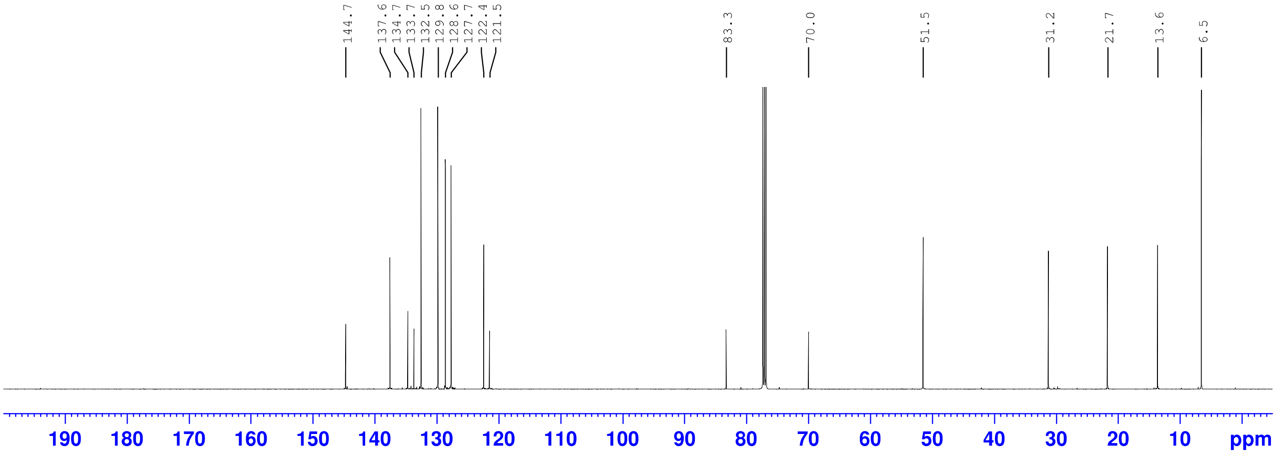

Supplementary Figure 13. ^{1}H and ^{13}C NMR spectra for compound **1c**

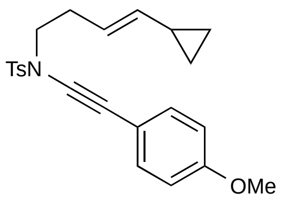

^{1}H NMR (500 MHz, $CDCl_3$)

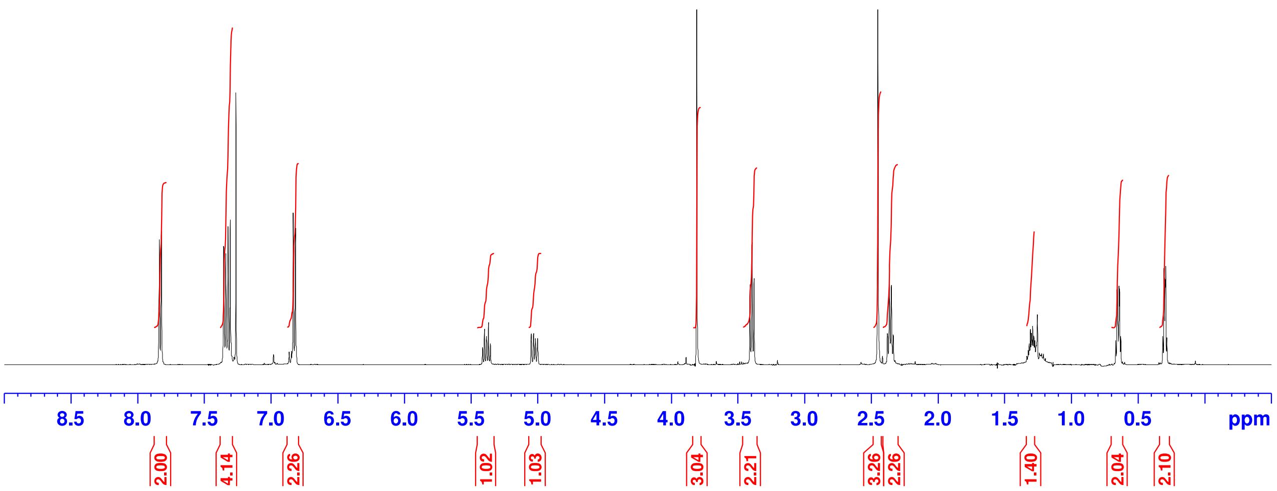

^{13}C NMR (125 MHz, $CDCl_3$)

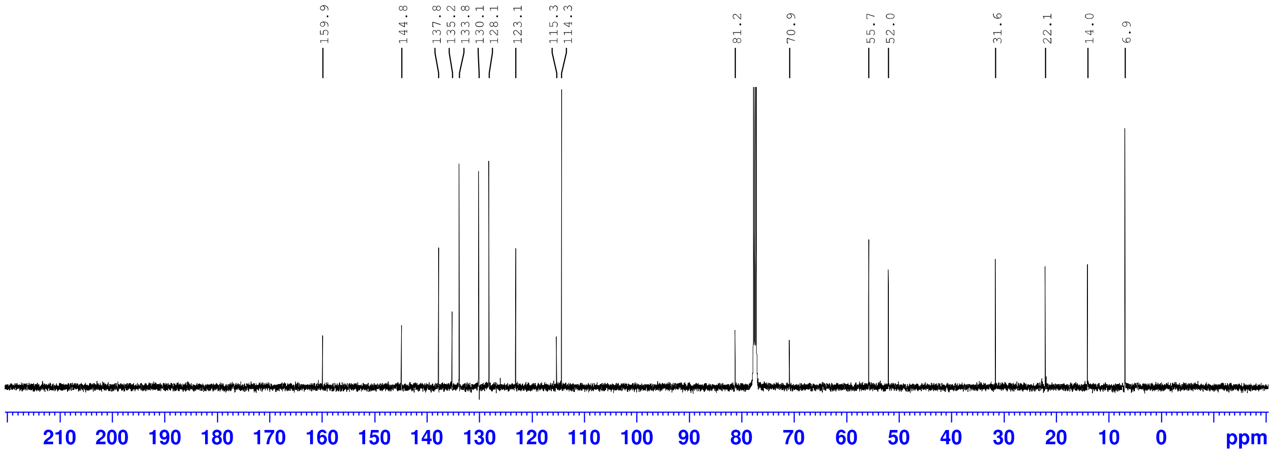

Supplementary Figure 14. ^{1}H and ^{13}C NMR spectra for compound **1d**

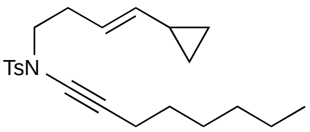

^{1}H NMR (500 MHz, $CDCl_3$)

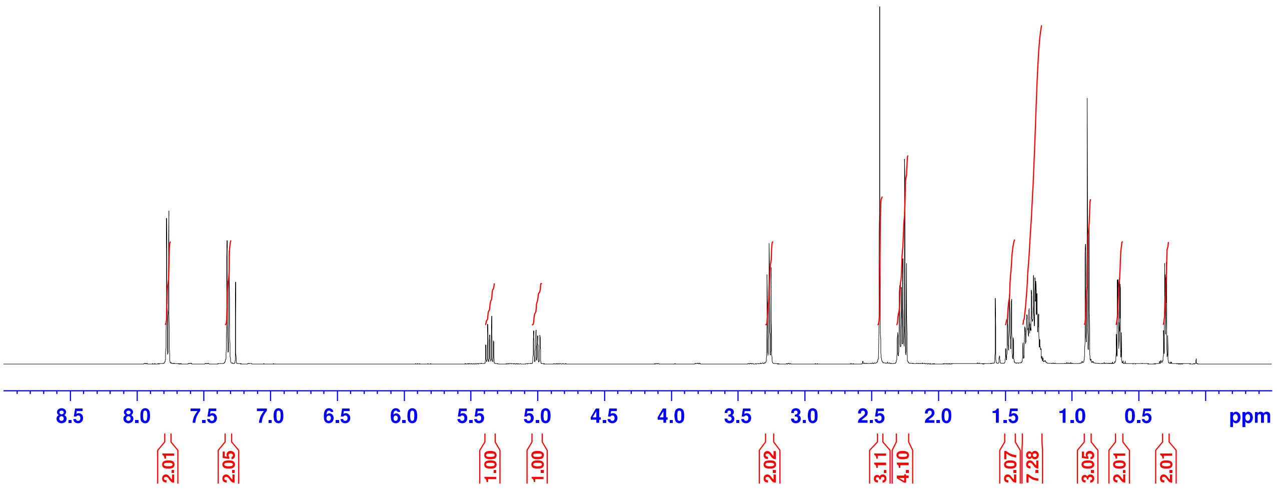

^{13}C NMR (125 MHz, $CDCl_3$)

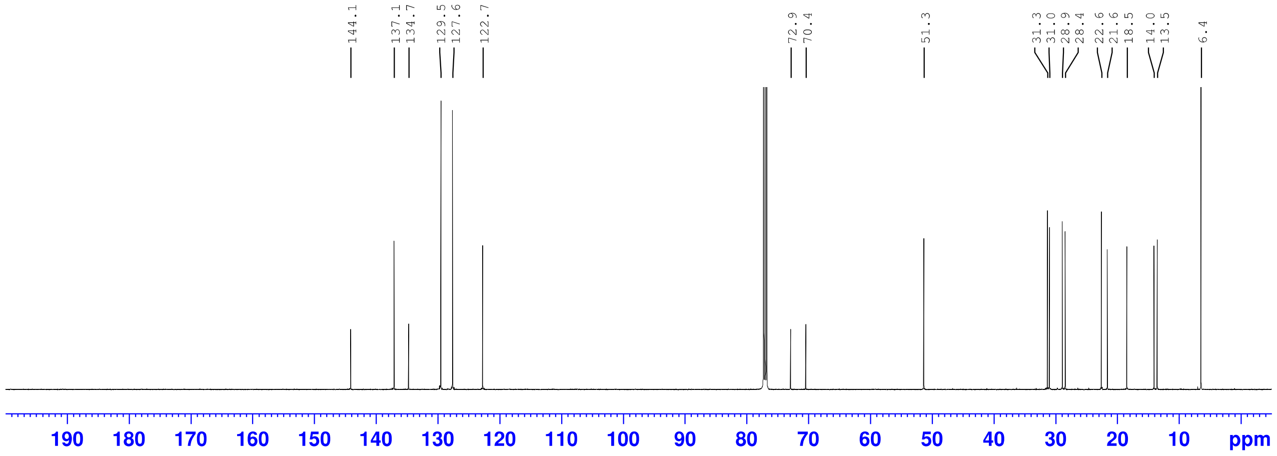

Supplementary Figure 15. ^{1}H and ^{13}C NMR spectra for compound **1e**

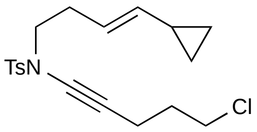

^{1}H NMR (500 MHz, $CDCl_3$)

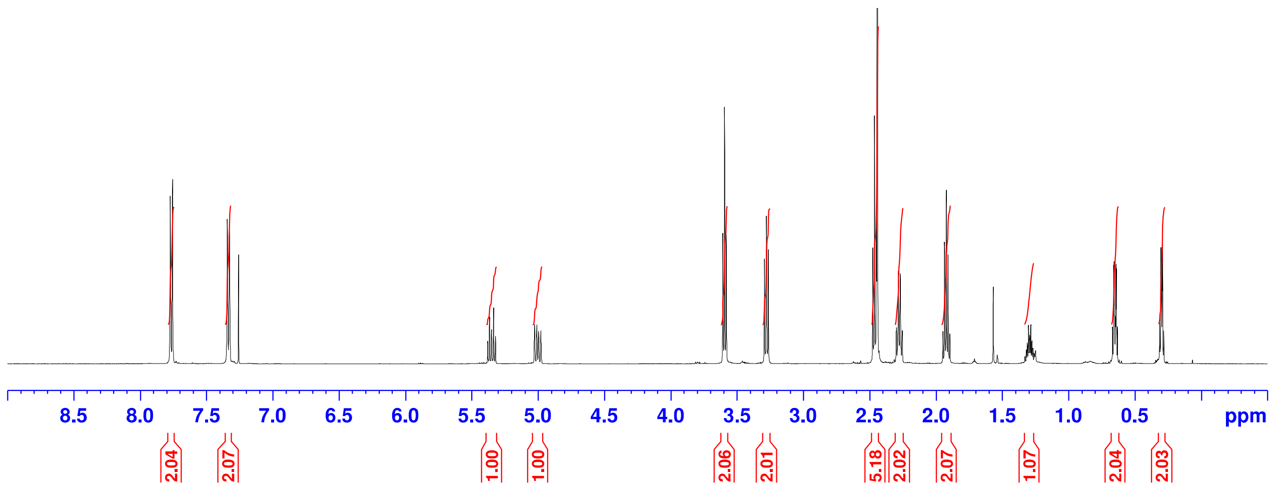

^{13}C NMR (125 MHz, $CDCl_3$)

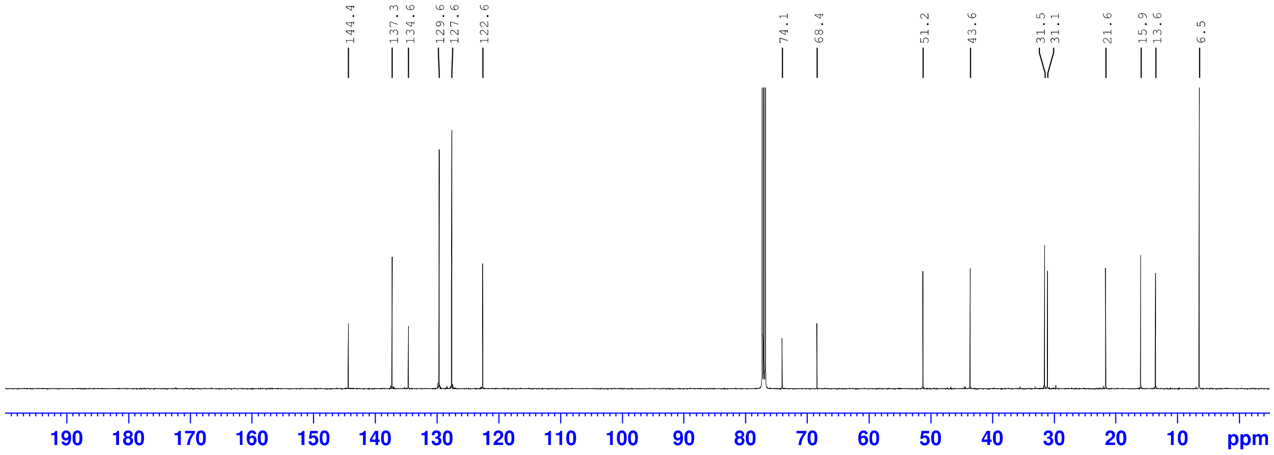

Supplementary Figure 16. ^{1}H and ^{13}C NMR spectra for compound **1f**

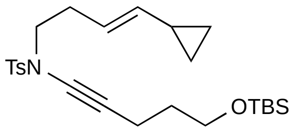

^{1}H NMR (500 MHz, $CDCl_3$)

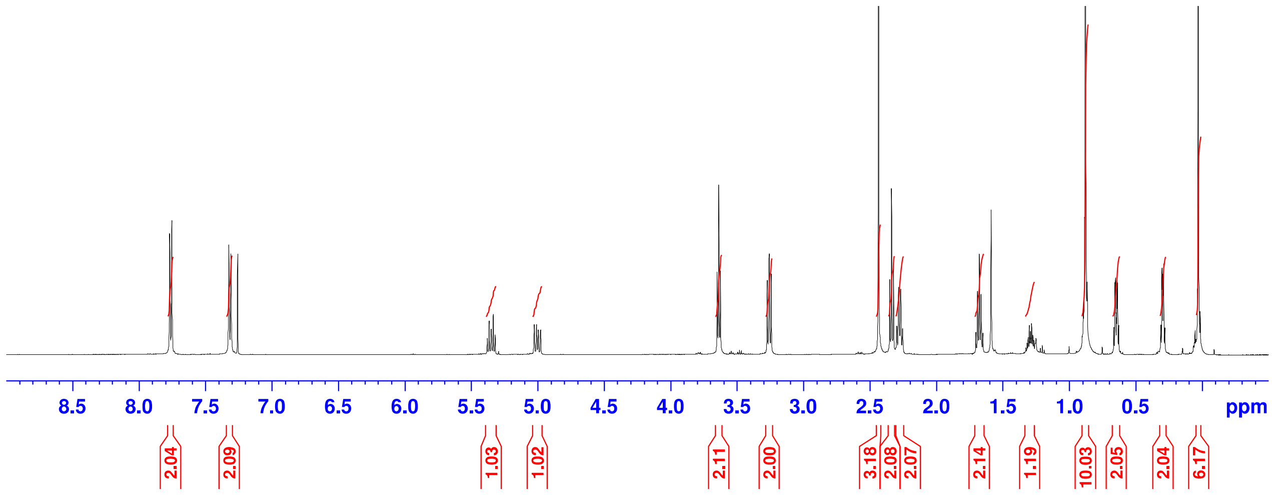

^{13}C NMR (125 MHz, $CDCl_3$)

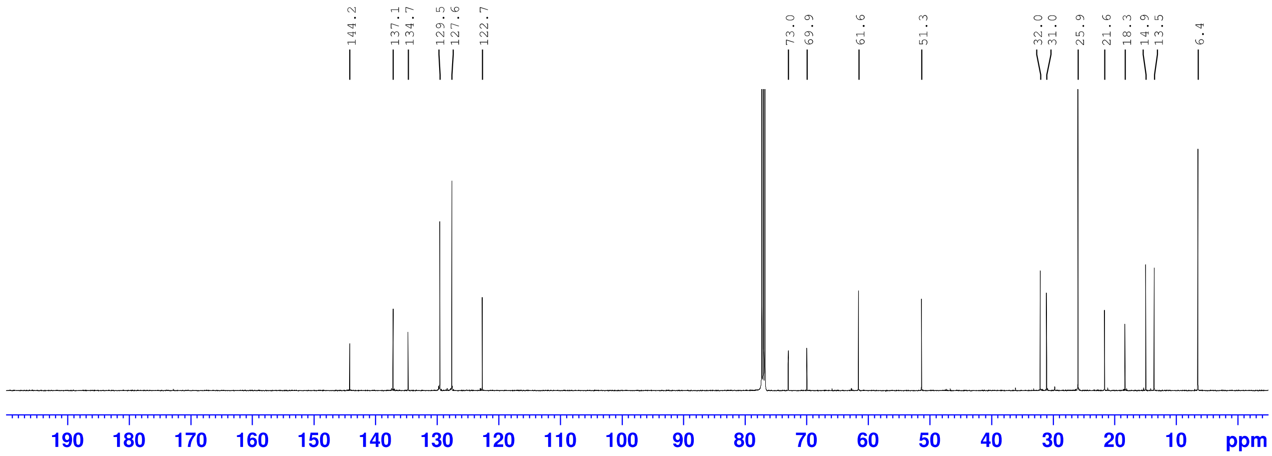

Supplementary Figure 17. ^{1}H and ^{13}C NMR spectra for compound **1g**

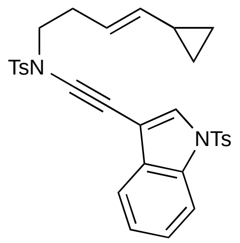

^{1}H NMR (500 MHz, $CDCl_3$)

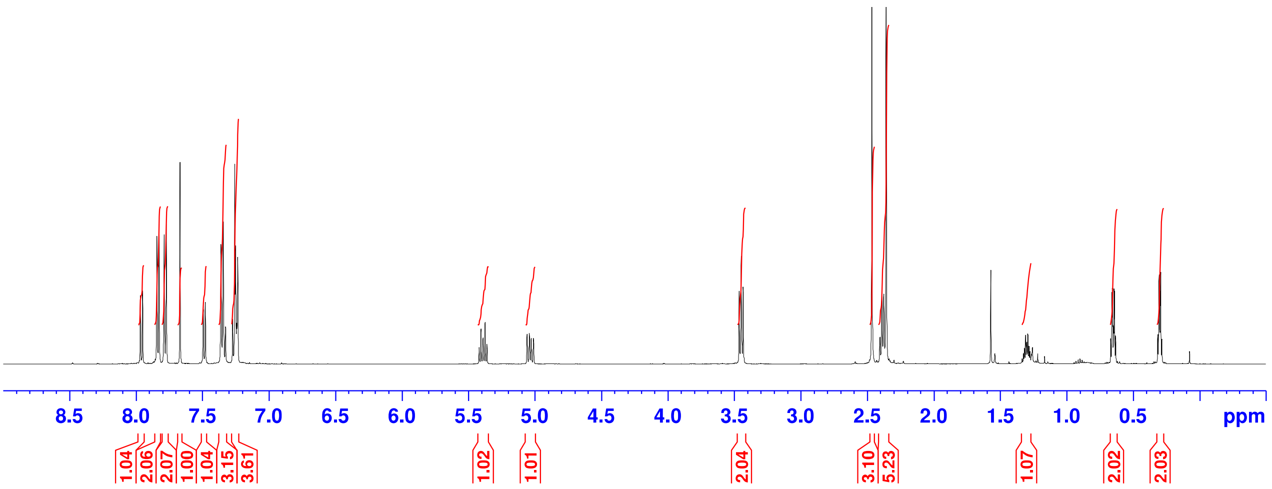

^{13}C NMR (125 MHz, $CDCl_3$)

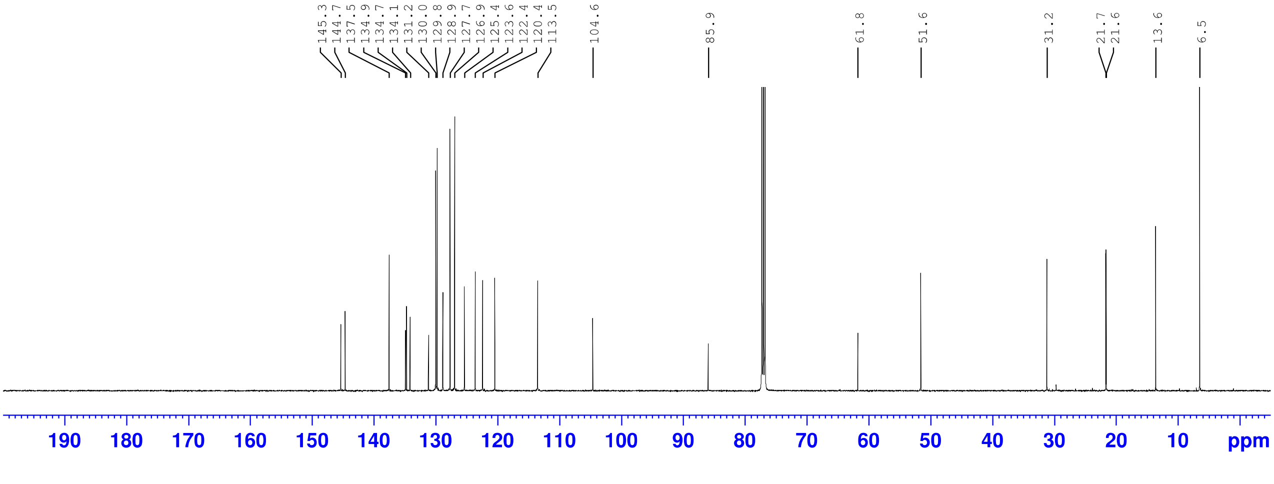

Supplementary Figure 18. ^{1}H and ^{13}C NMR spectra for compound **1h**

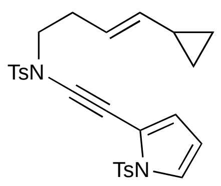

¹H NMR (500 MHz, $CDCl_3$)

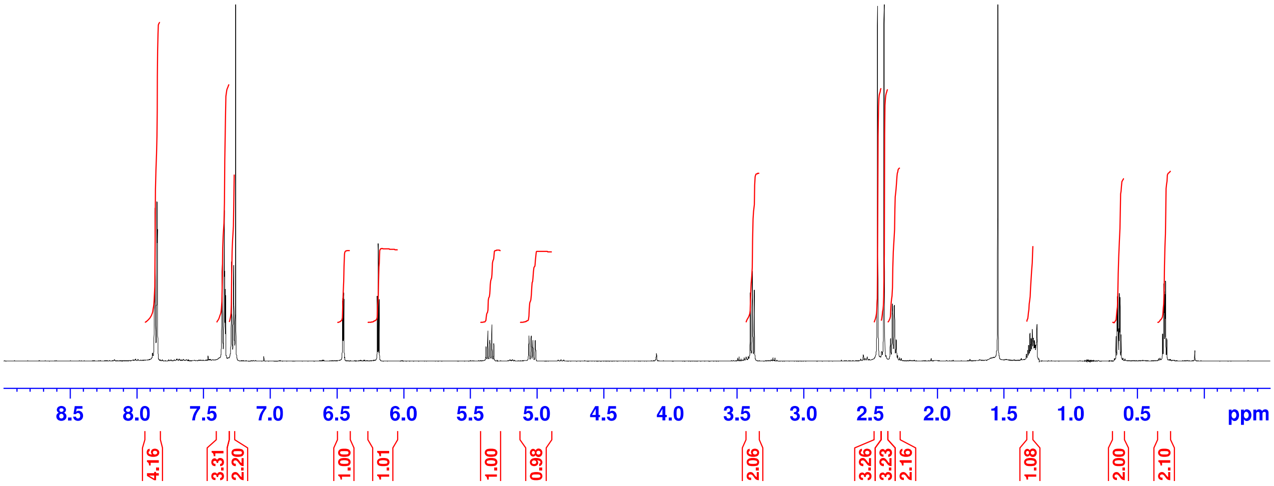

¹³C NMR (125 MHz, $CDCl_3$)

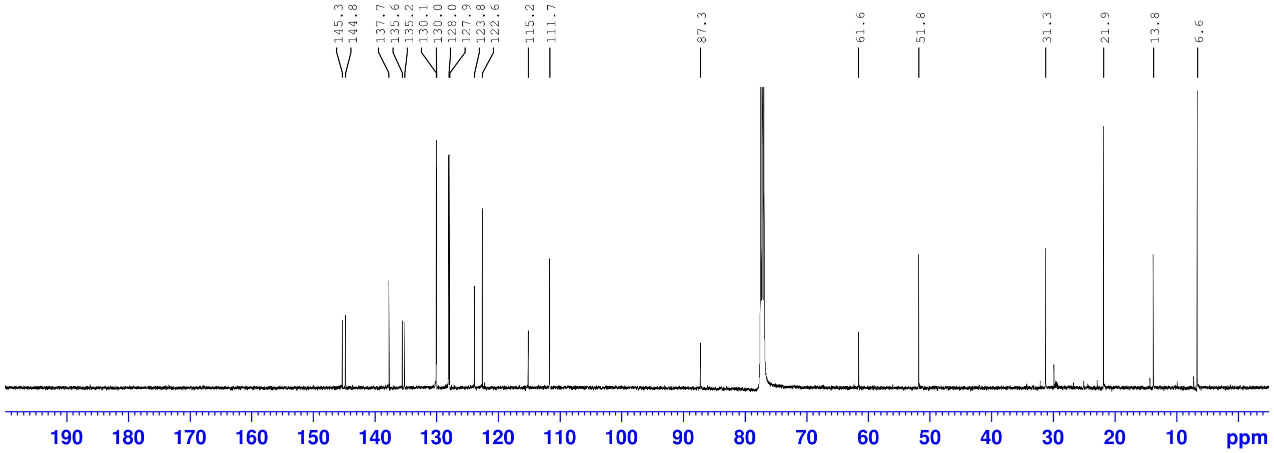

Supplementary Figure 19. ¹H and ¹³C NMR spectra for compound **1i**

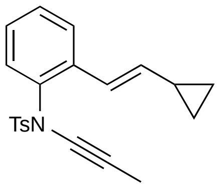

^{1}H NMR (500 MHz, $CDCl_3$)

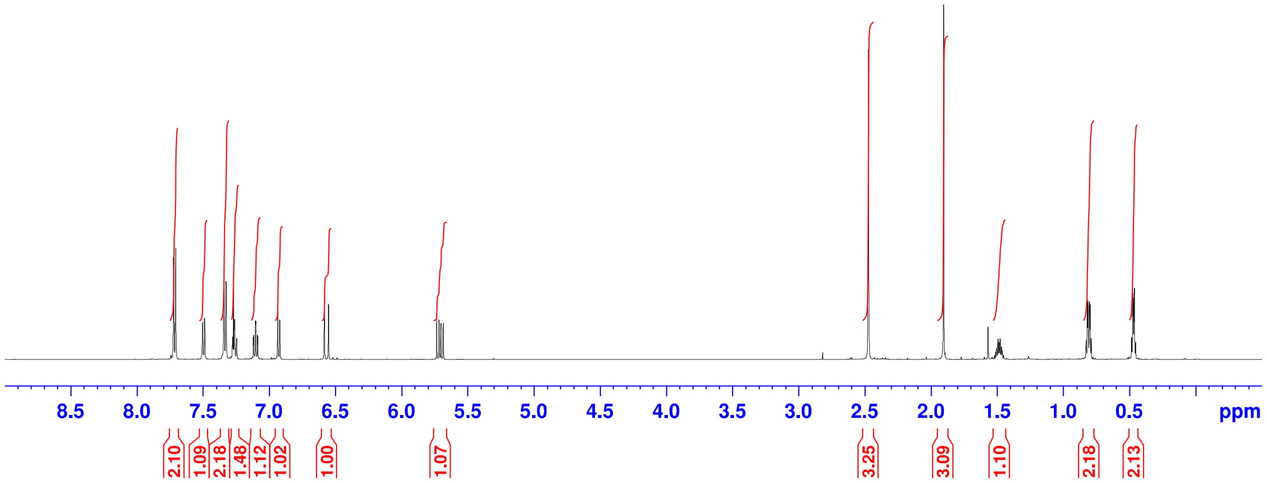

^{13}C NMR (125 MHz, $CDCl_3$)

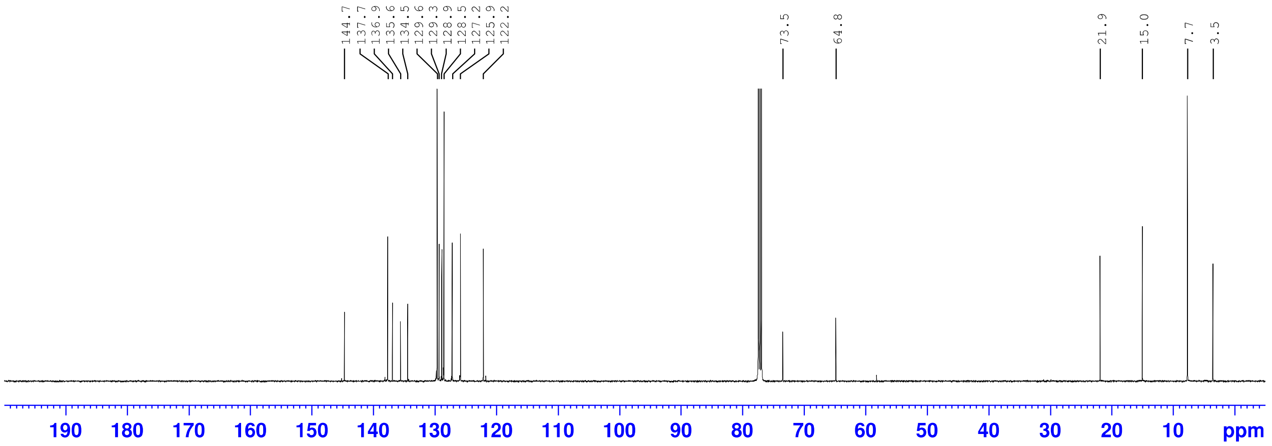

Supplementary Figure 20. ^{1}H and ^{13}C NMR spectra for compound **1j**

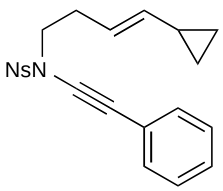

^{1}H NMR (500 MHz, $CDCl_3$)

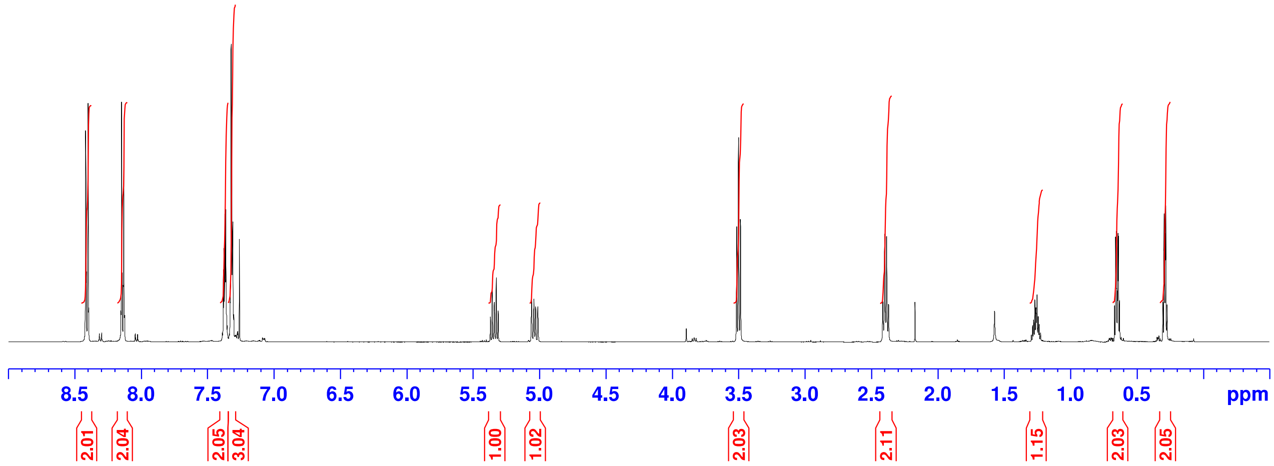

^{13}C NMR (125 MHz, $CDCl_3$)

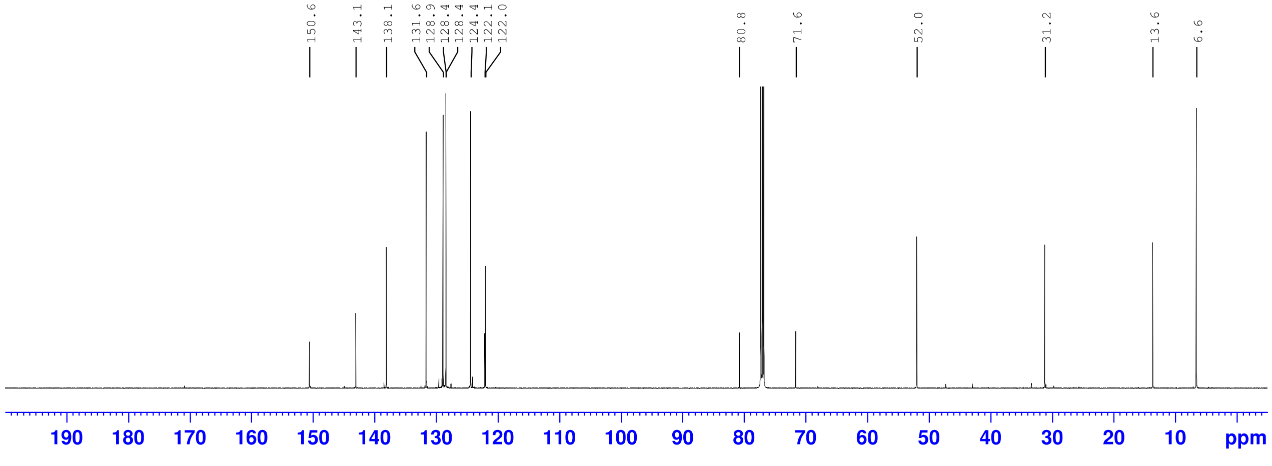

Supplementary Figure 21. ^{1}H and ^{13}C NMR spectra for compound **1k**

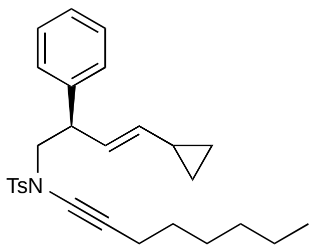

^{1}H NMR (700 MHz, $CDCl_3$)

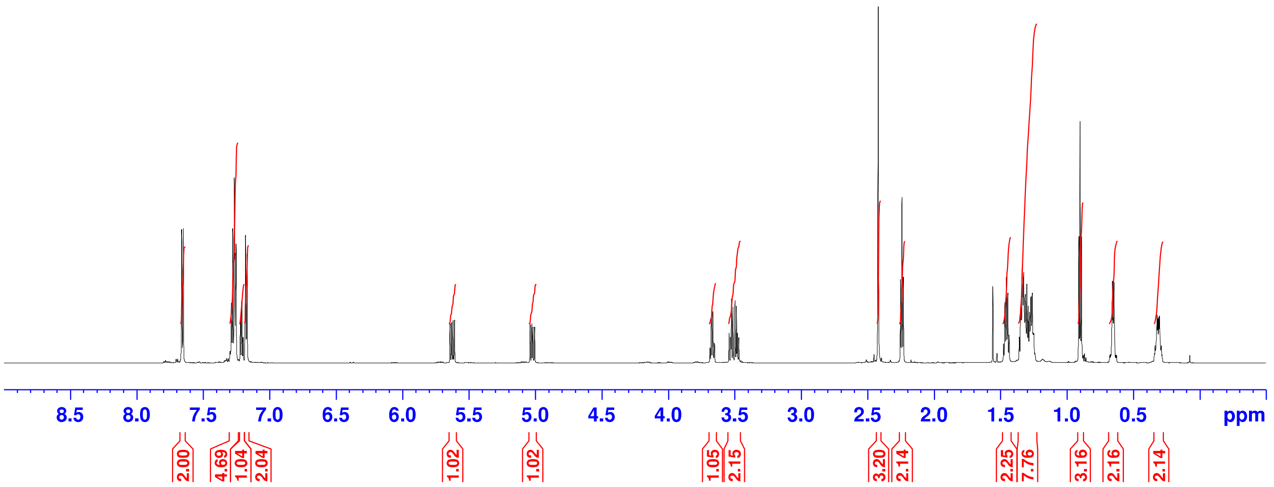

^{13}C NMR (176 MHz, $CDCl_3$)

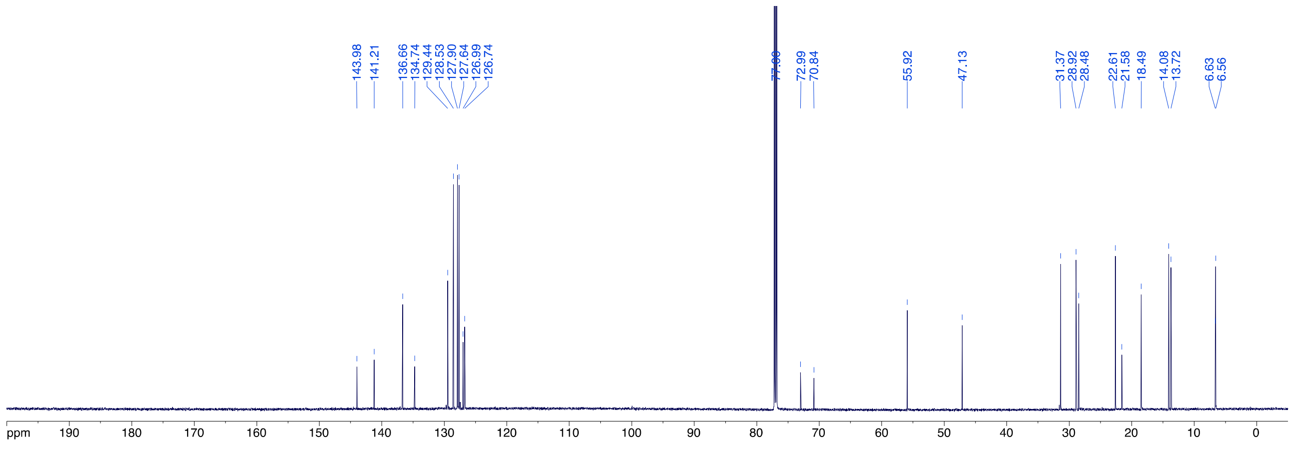

Supplementary Figure 22. ^{1}H and ^{13}C NMR spectra for compound **1l**

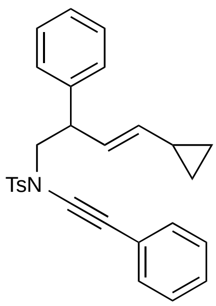

¹H NMR (700 MHz, $CDCl_3$)

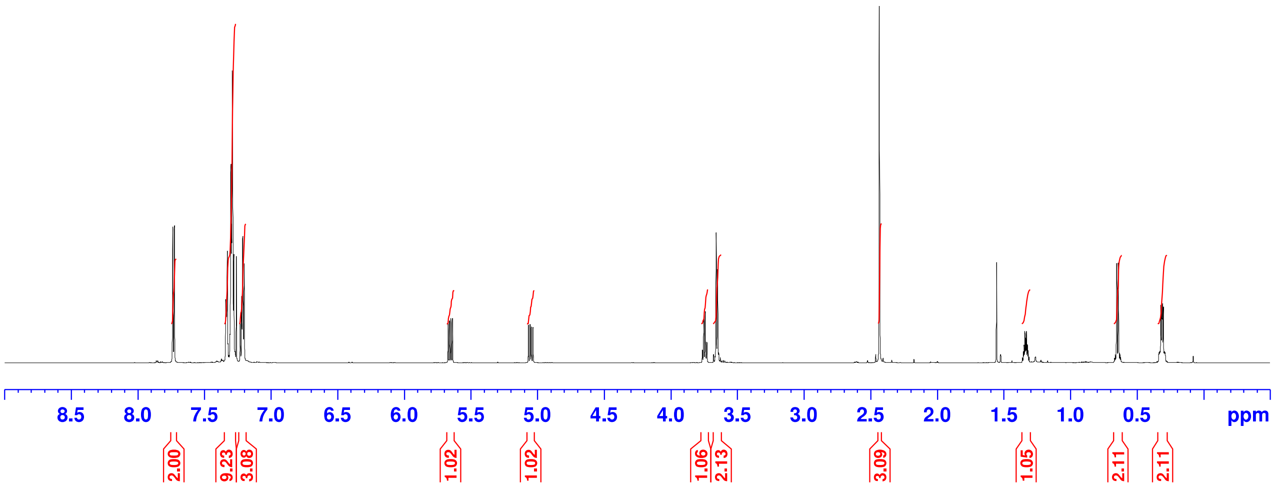

¹³C NMR (176 MHz, $CDCl_3$)

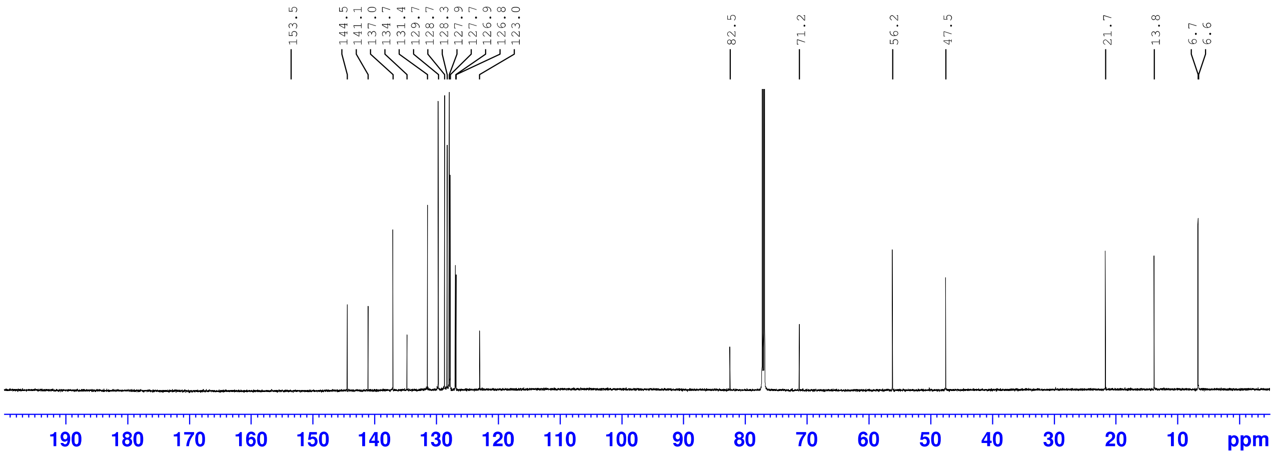

Supplementary Figure 23. ¹H and ¹³C NMR spectra for compound **1m**

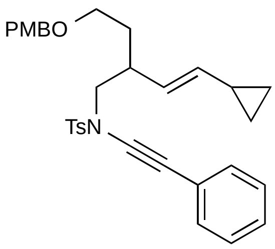

^{1}H NMR (500 MHz, $CDCl_3$)

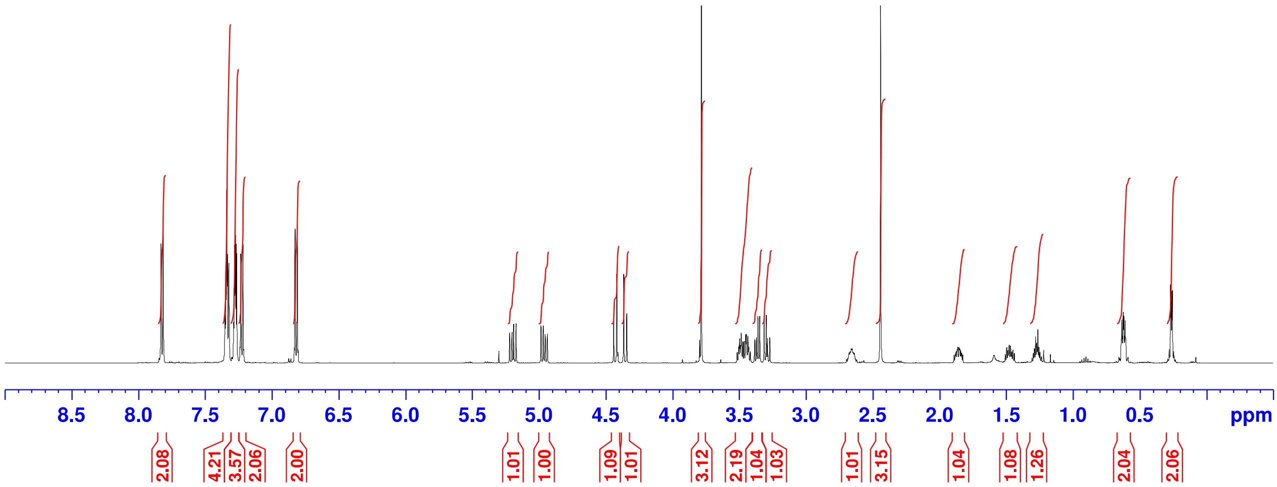

^{13}C NMR (125 MHz, $CDCl_3$)

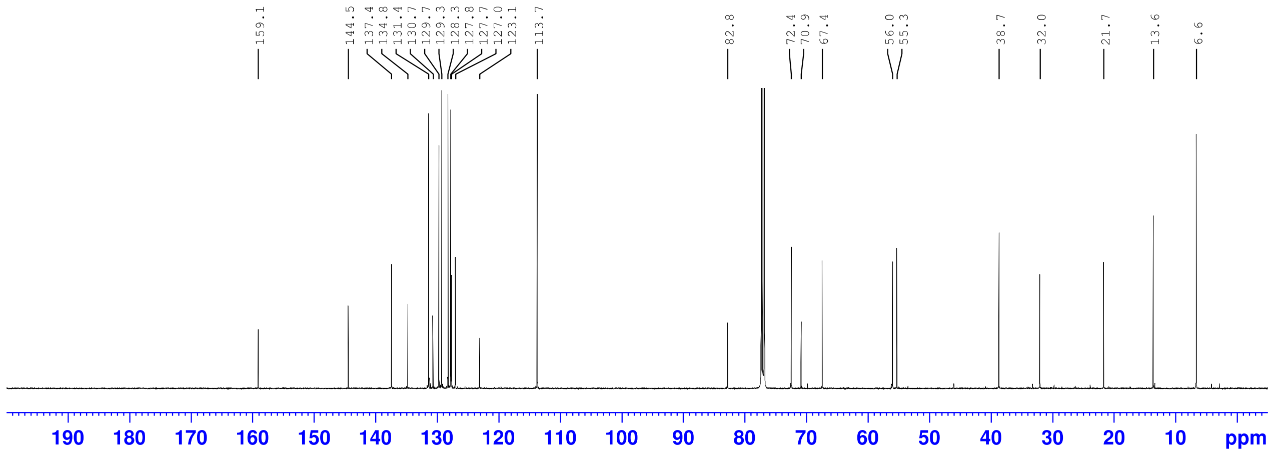

Supplementary Figure 24. ^{1}H and ^{13}C NMR spectra for compound **1n**

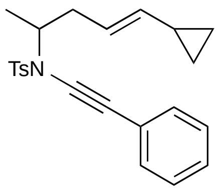

^{1}H NMR (500 MHz, $CDCl_3$)

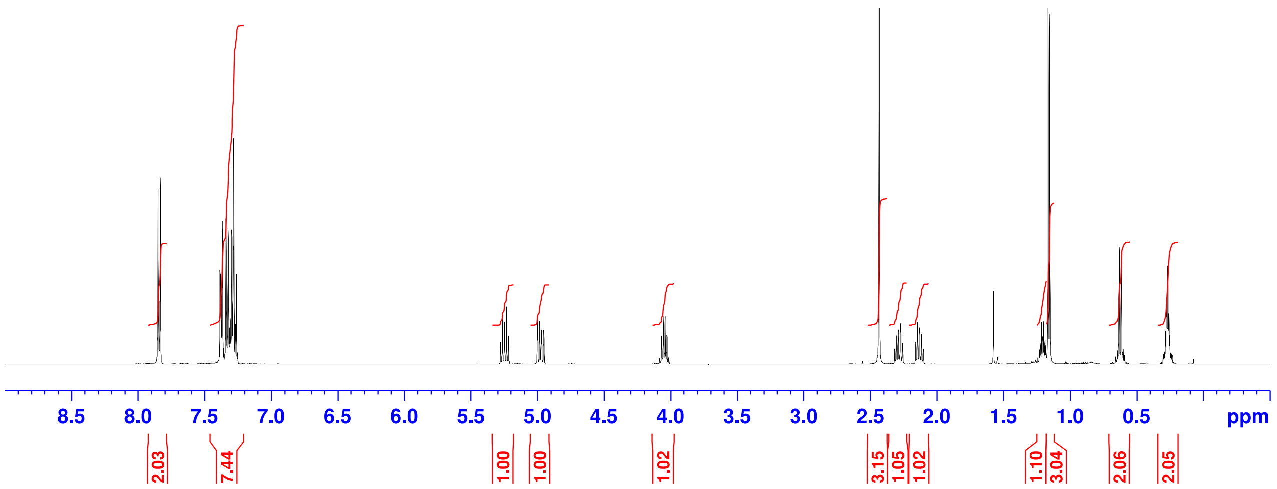

^{13}C NMR (125 MHz, $CDCl_3$)

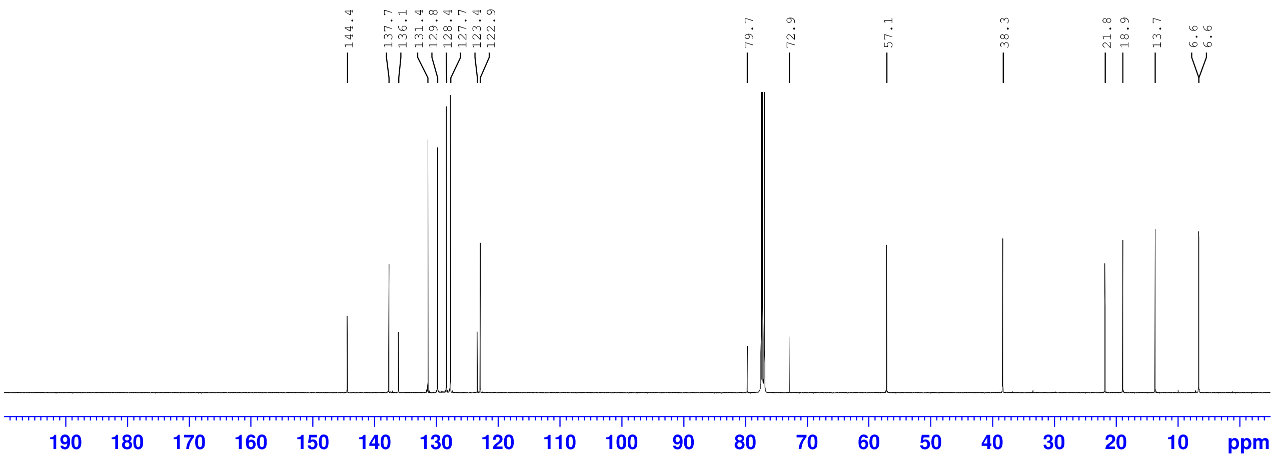

Supplementary Figure 25. ^{1}H and ^{13}C NMR spectra for compound **1o**

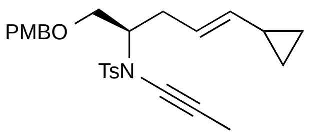

^{1}**H NMR** (500 MHz, $CDCl_3$)

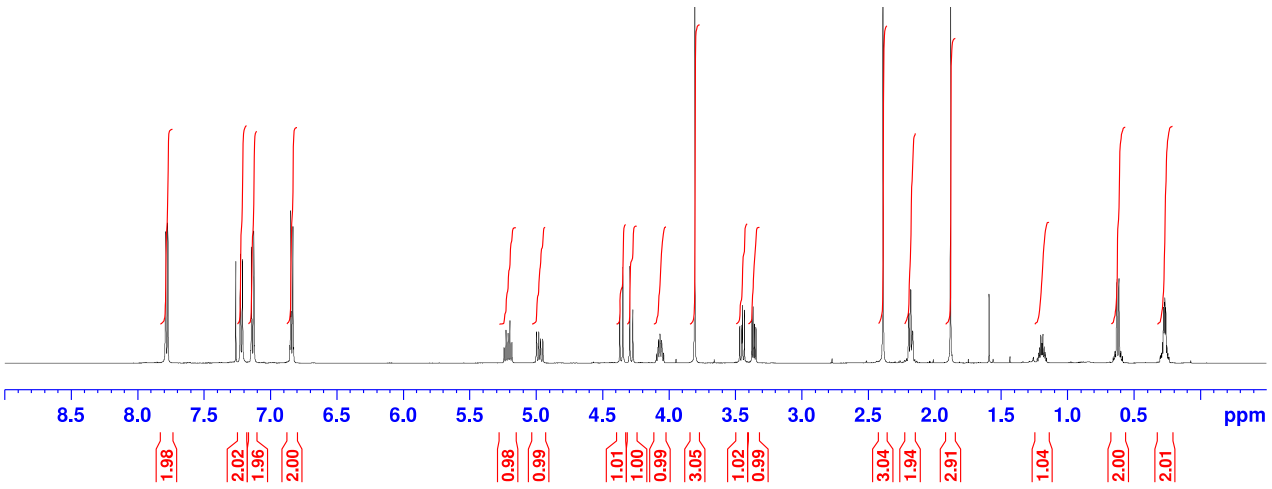

^{13}**C NMR** (125 MHz, $CDCl_3$)

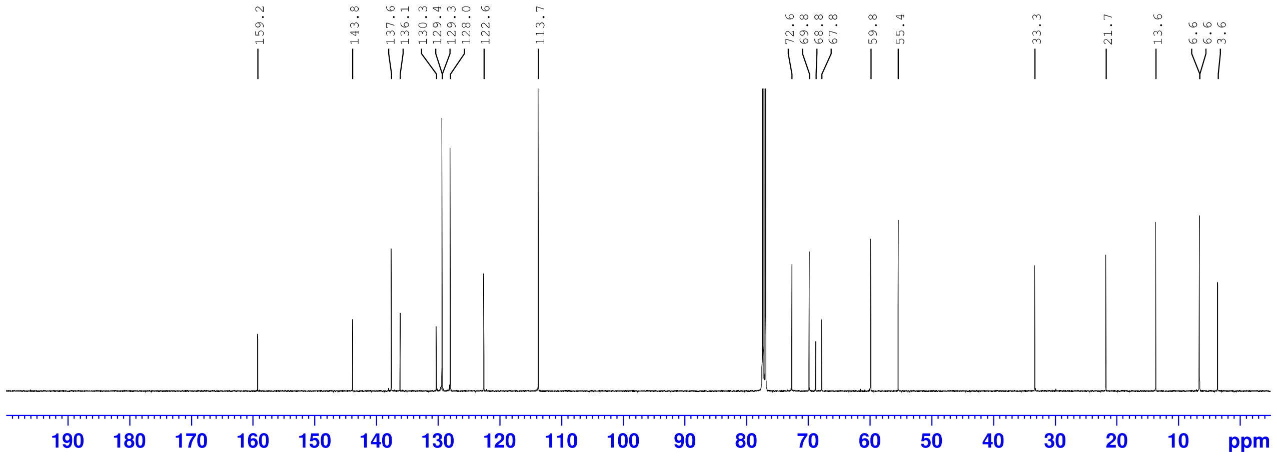

Supplementary Figure 26. ^{1}H and ^{13}C NMR spectra for compound **1p**

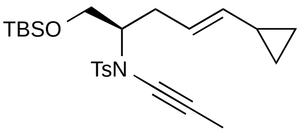

^{1}H NMR (500 MHz, $CDCl_3$)

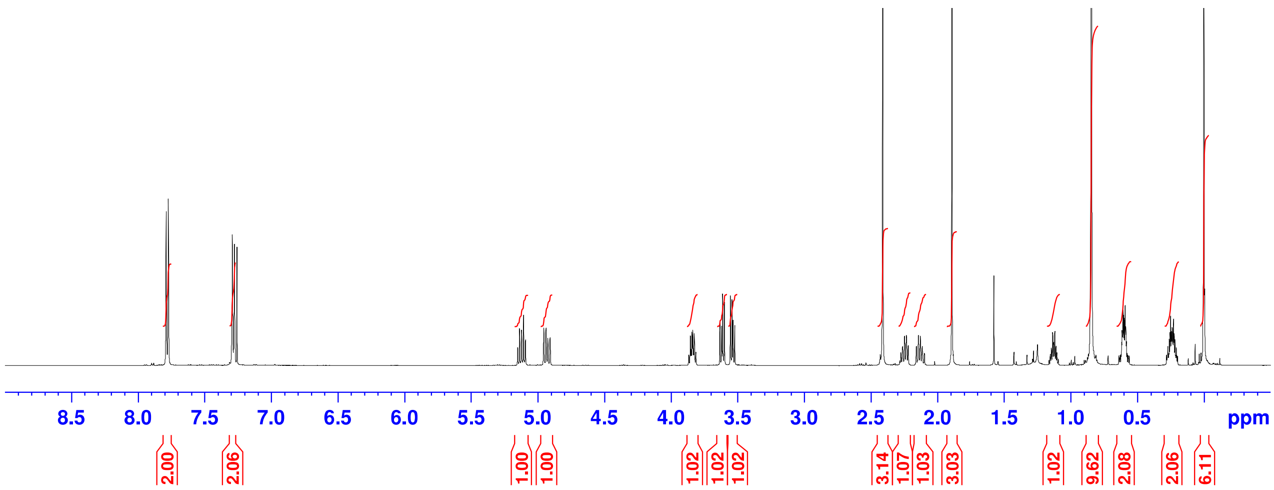

^{13}C NMR (125 MHz, $CDCl_3$)

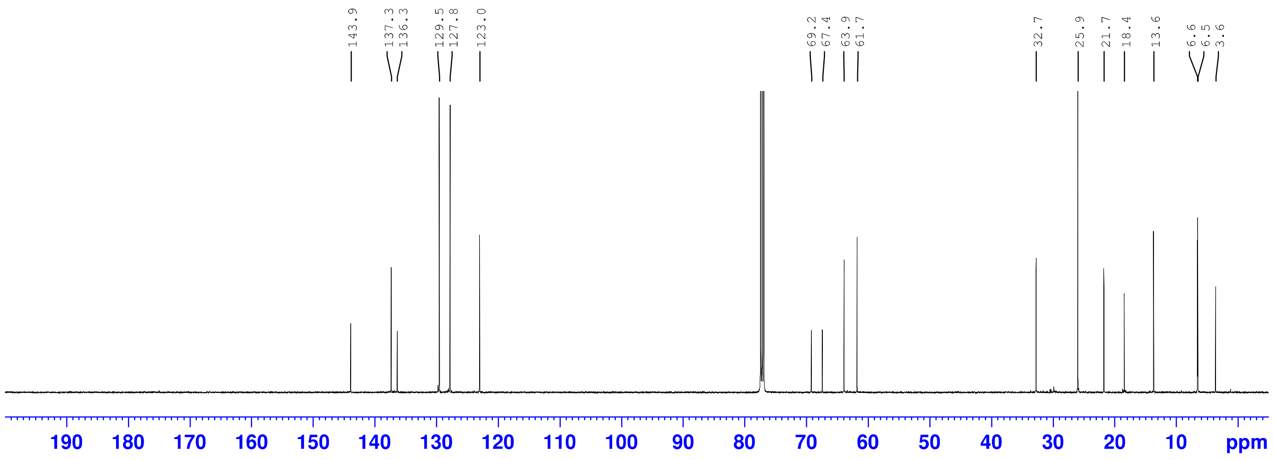

Supplementary Figure 27. ^{1}H and ^{13}C NMR spectra for compound **1q**

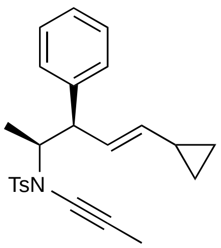

¹H NMR (500 MHz, $CDCl_3$)

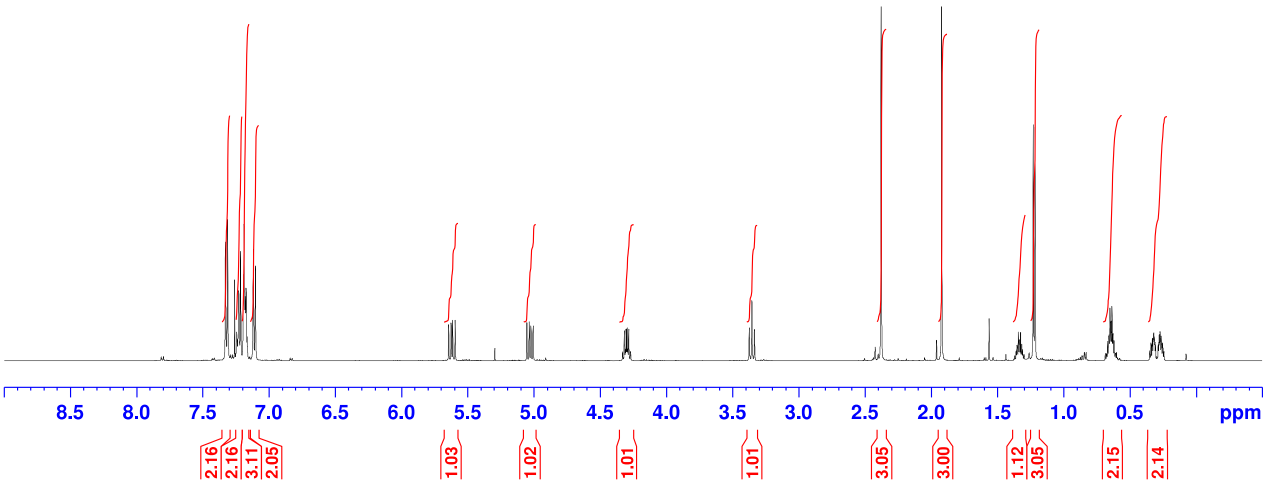

¹³C NMR (125 MHz, $CDCl_3$)

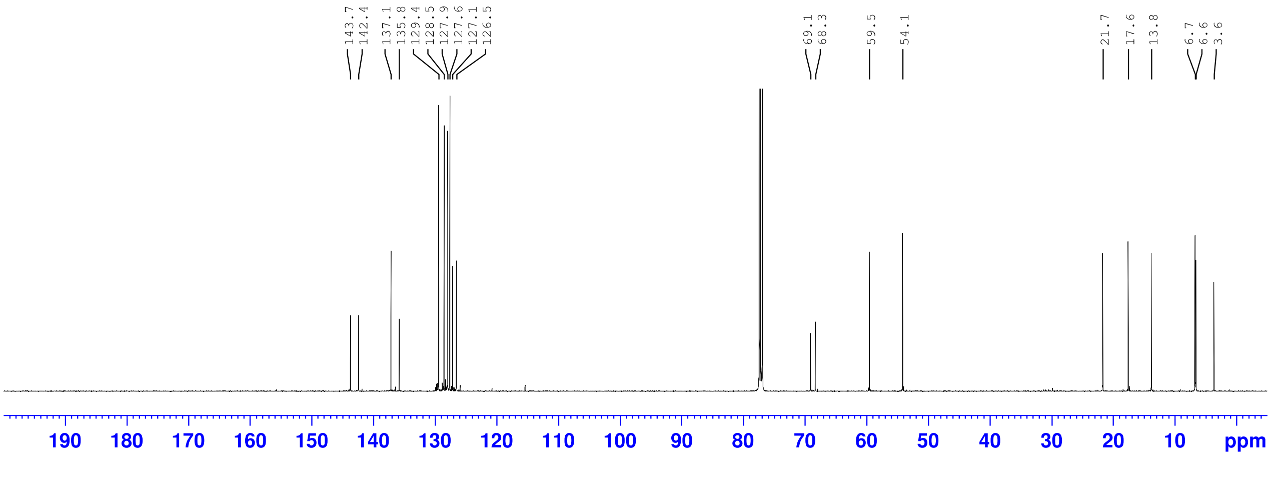

Supplementary Figure 28. ¹H and ¹³C NMR spectra for compound **1r**

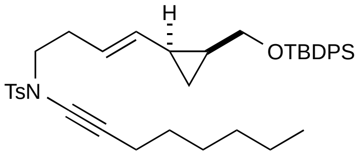

¹H NMR (500 MHz, $CDCl_3$)

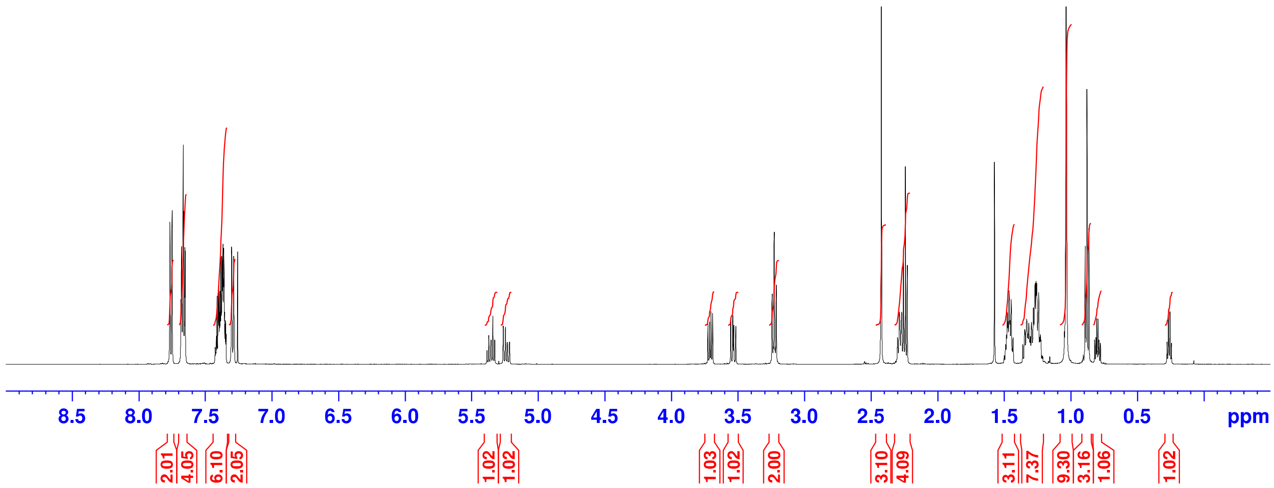

¹³C NMR (125 MHz, $CDCl_3$)

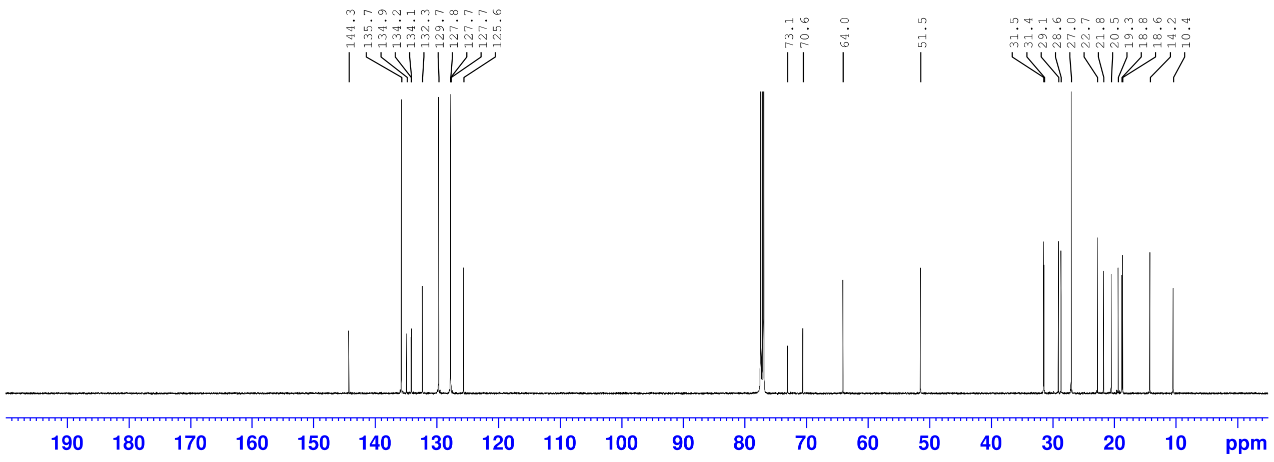

Supplementary Figure 29. ¹H and ¹³C NMR spectra for compound **1s**

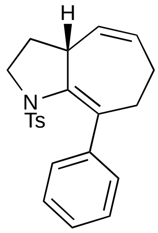

^{1}**H NMR** (500 MHz, C_6D_6)

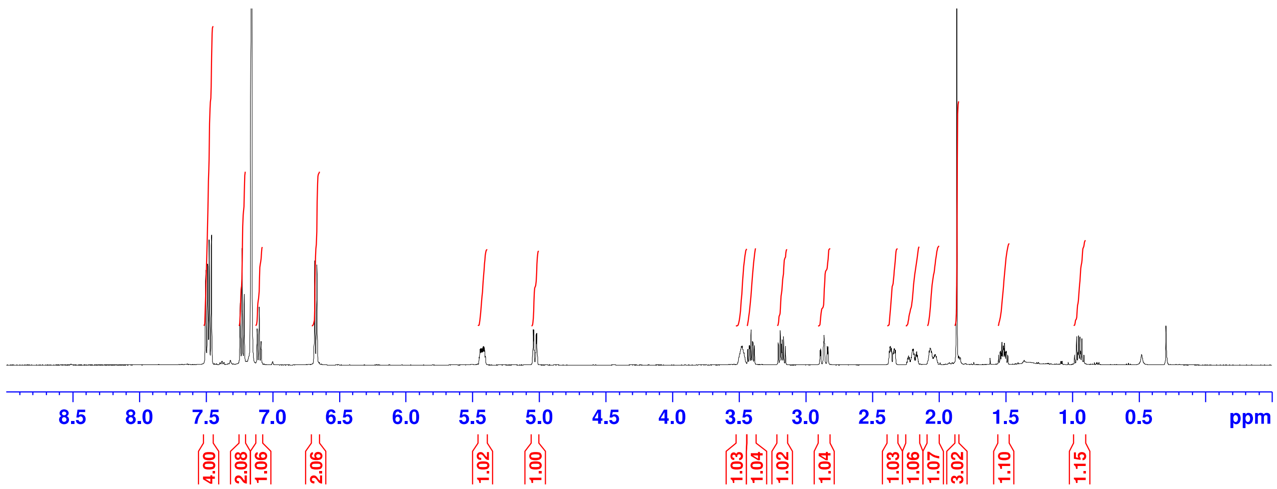

^{13}**C NMR** (125 MHz, C_6D_6)

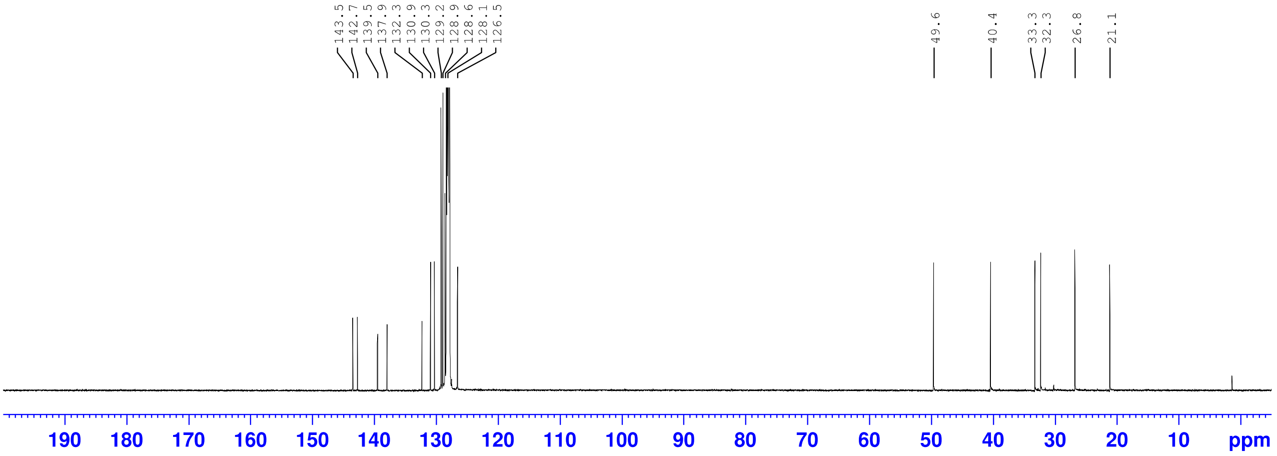

Supplementary Figure 30. ^{1}H and ^{13}C NMR spectra for compound **7a**

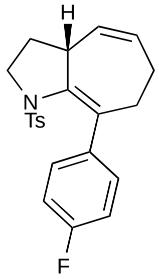

^{1}H NMR (500 MHz, C_6D_6)

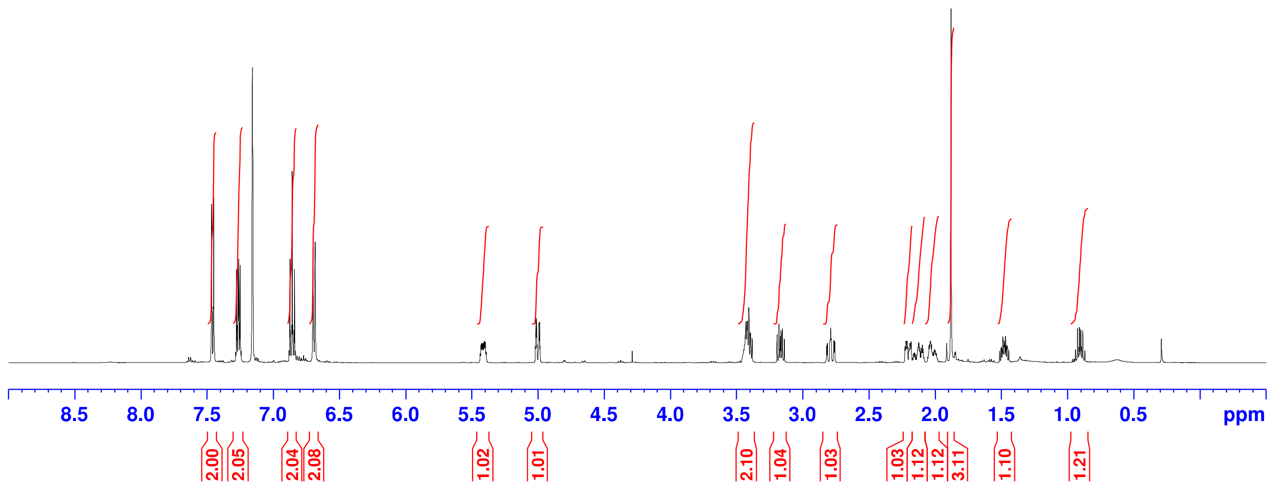

^{13}C NMR (125 MHz, C_6D_6)

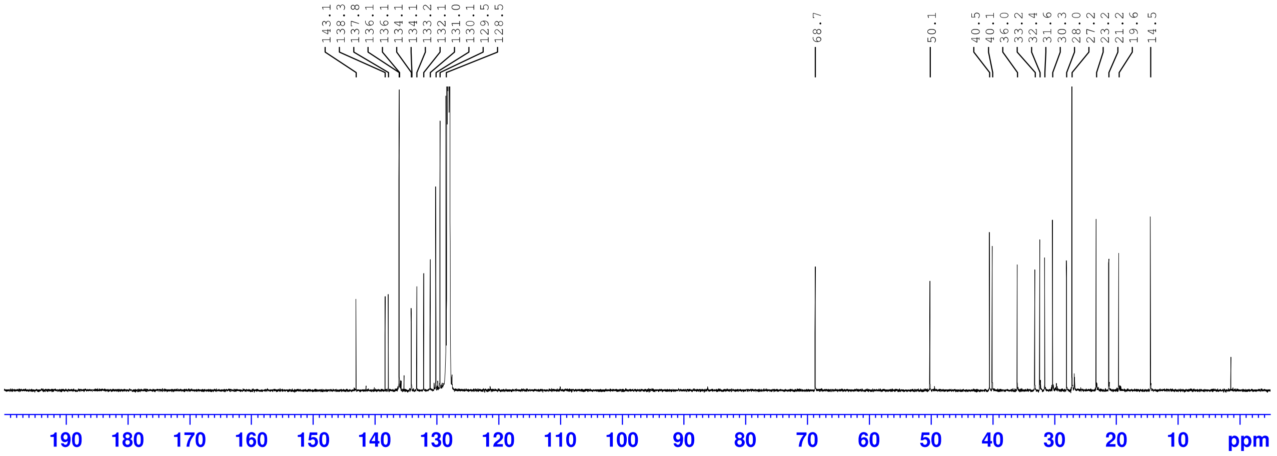

Supplementary Figure 31. ^{1}H and ^{13}C NMR spectra for compound **7b**

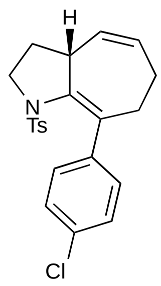

^{1}**H NMR** (500 MHz, C_6D_6)

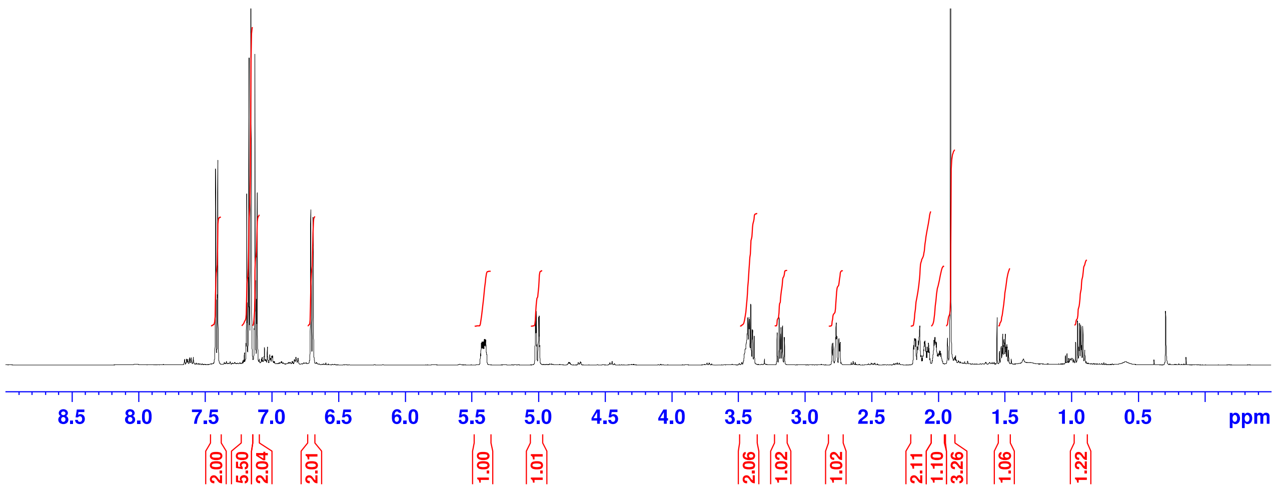

^{13}**C NMR** (125 MHz, C_6D_6)

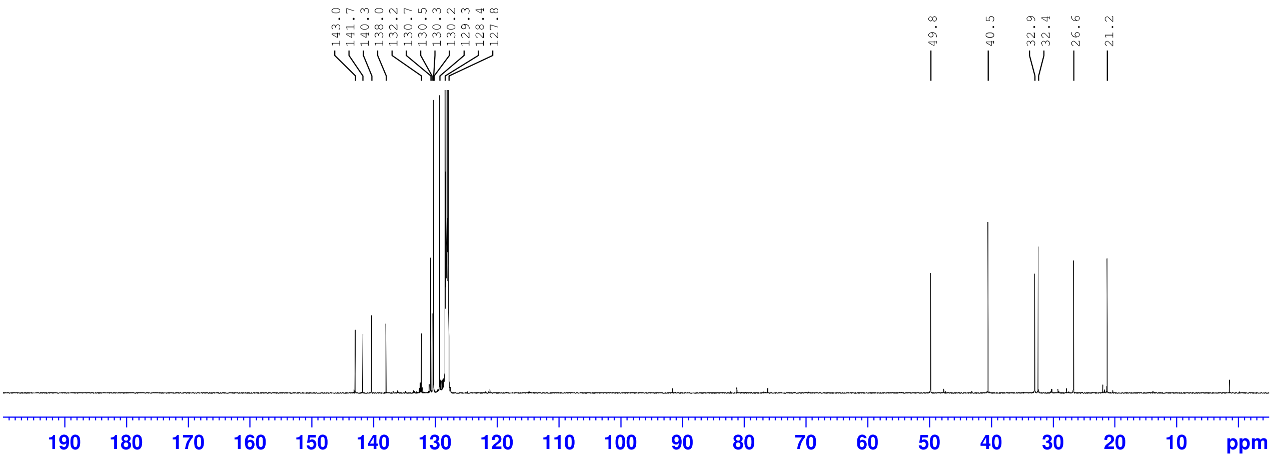

Supplementary Figure 32. ^{1}H and ^{13}C NMR spectra for compound **7c**

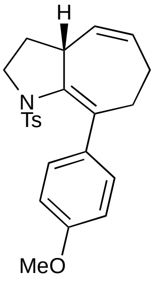

^{1}H NMR (500 MHz, C_6D_6)

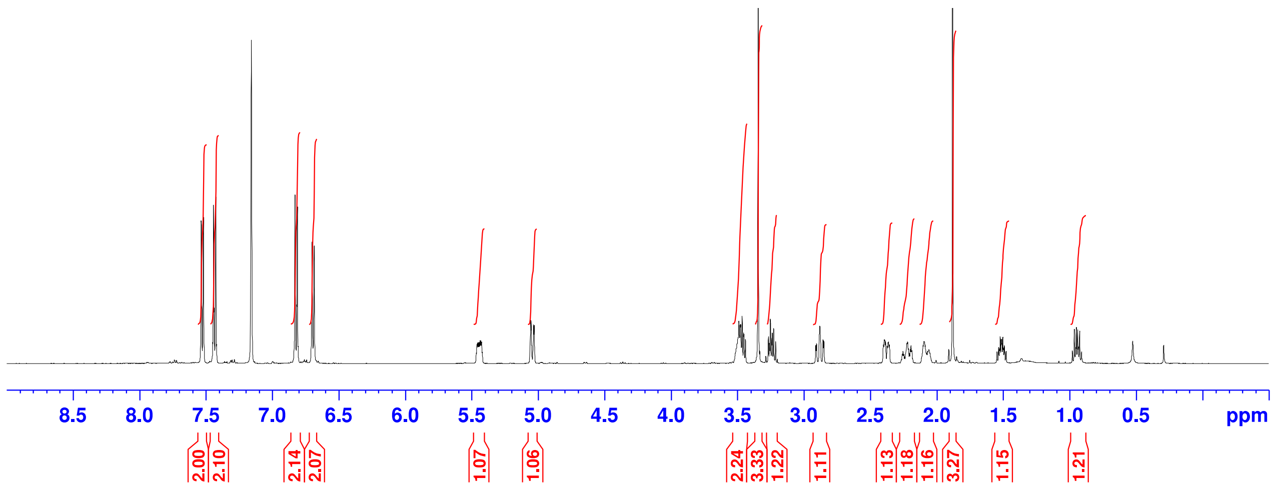

^{13}C NMR (125 MHz, C_6D_6)

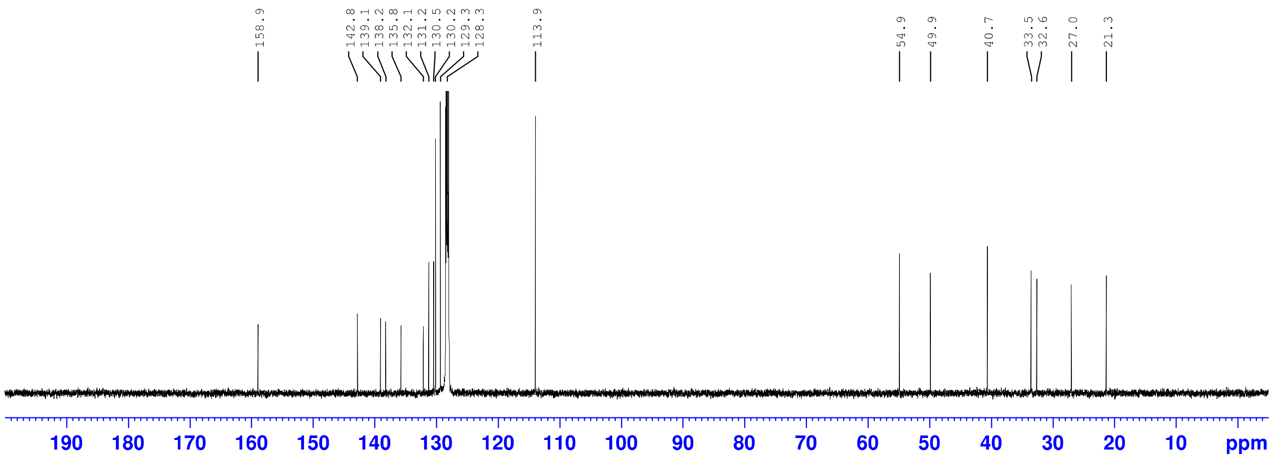

Supplementary Figure 33. ^{1}H and ^{13}C NMR spectra for compound **7d**

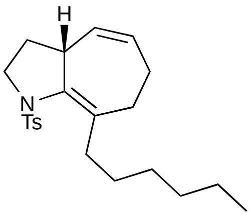

^{1}H NMR (500 MHz, C_6D_6)

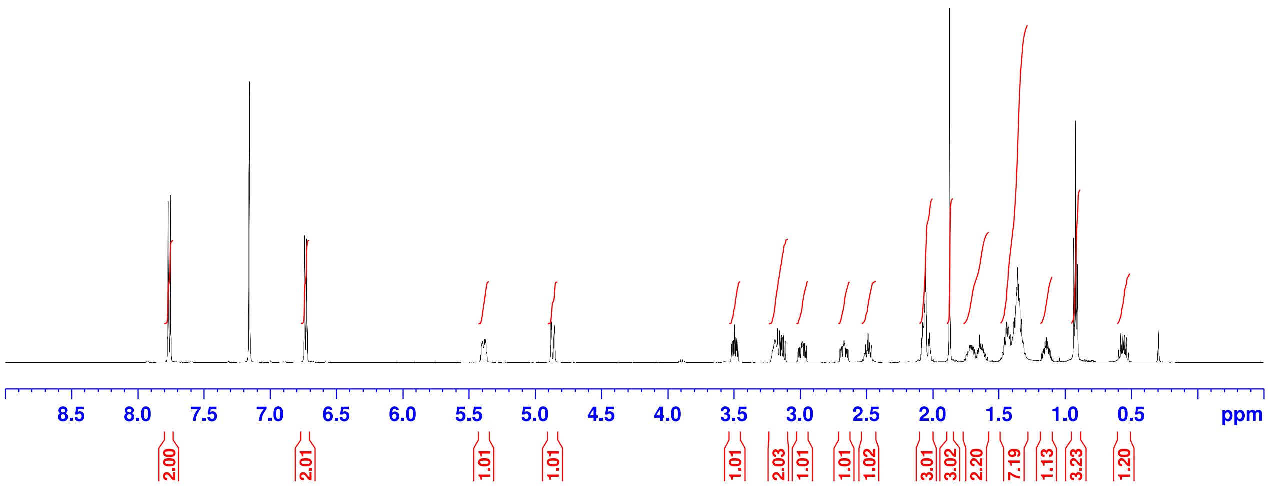

^{13}C NMR (125 MHz, C_6D_6)

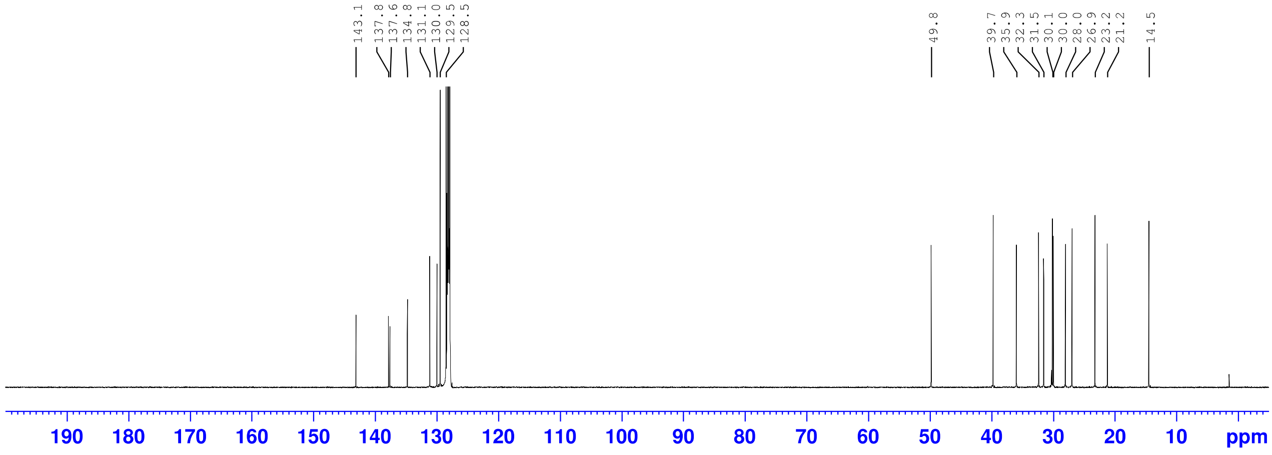

Supplementary Figure 34. ^{1}H and ^{13}C NMR spectra for compound **7e**

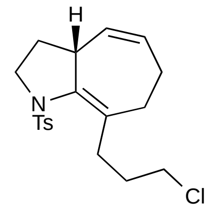

^{1}H NMR (500 MHz, C_6D_6)

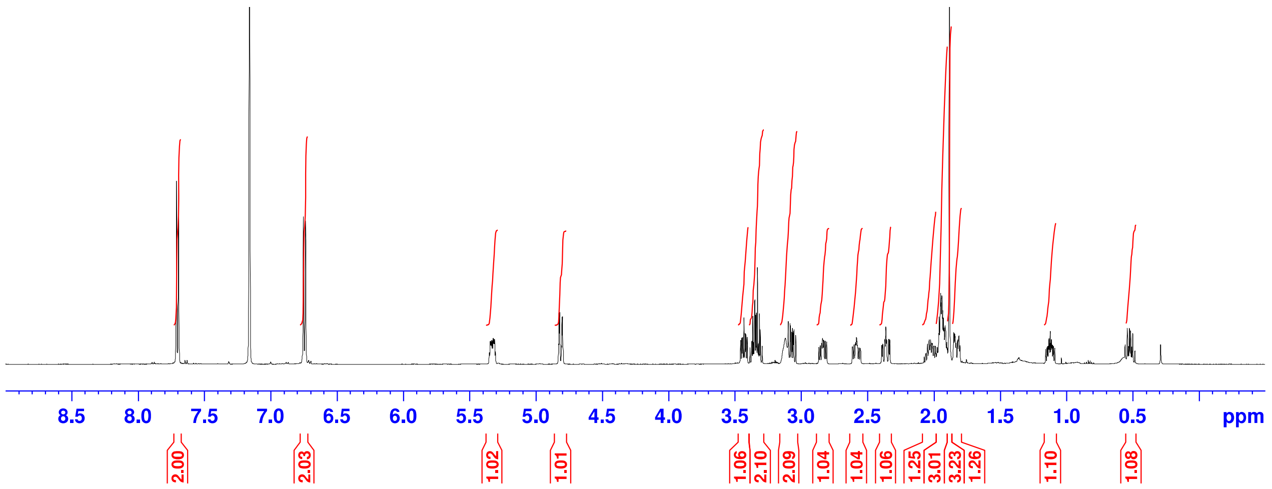

^{13}C NMR (125 MHz, C_6D_6)

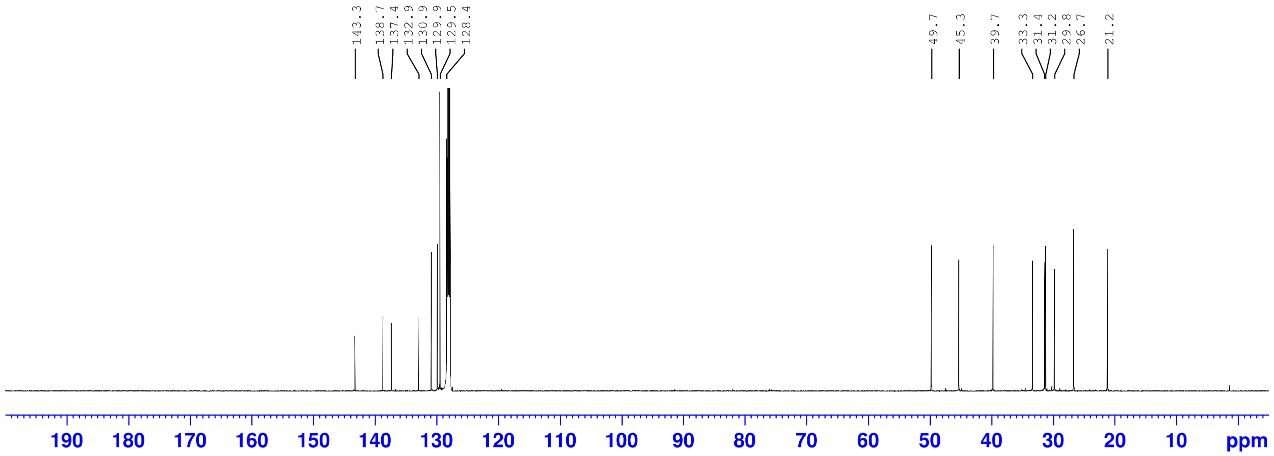

Supplementary Figure 35. ^{1}H and ^{13}C NMR spectra for compound **7f**

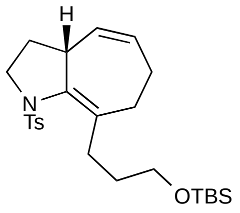

^{1}H NMR (500 MHz, C_6D_6)

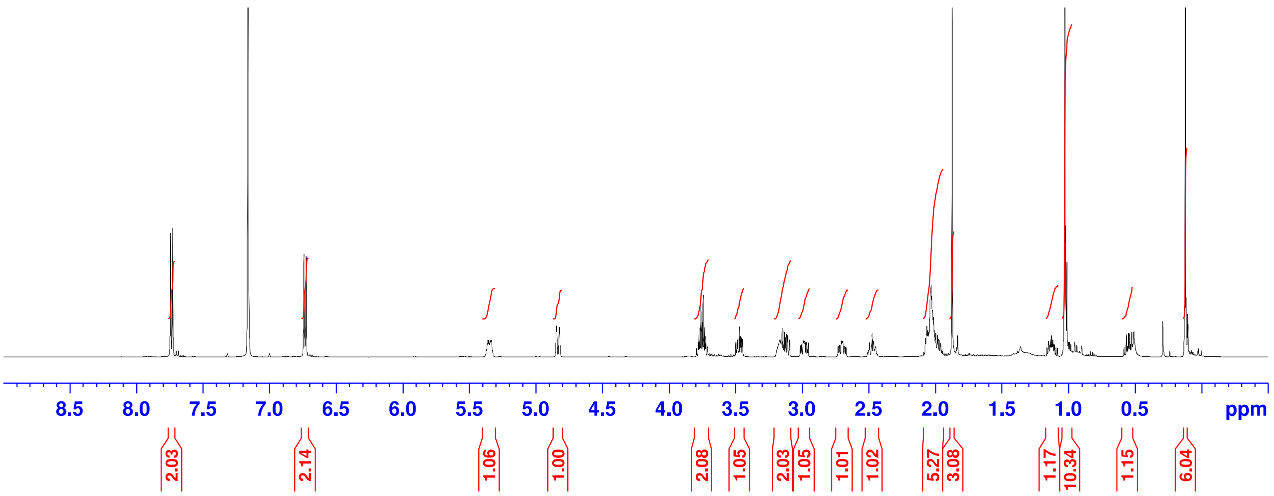

^{13}C NMR (125 MHz, C_6D_6)

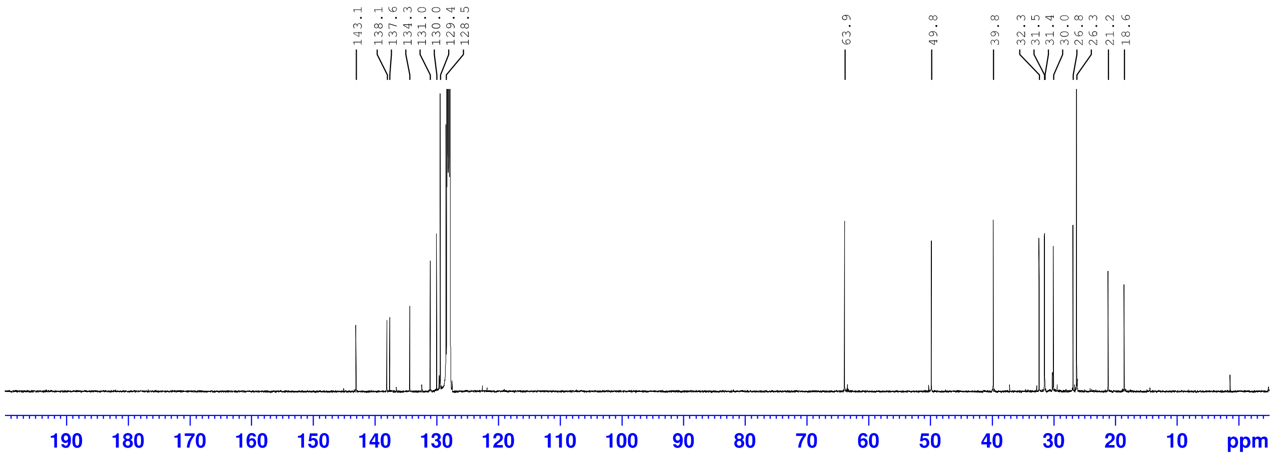

Supplementary Figure 36. ^{1}H and ^{13}C NMR spectra for compound **7g**

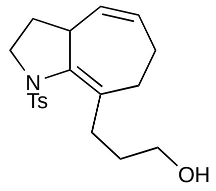

^{1}H NMR (500 MHz, C_6D_6)

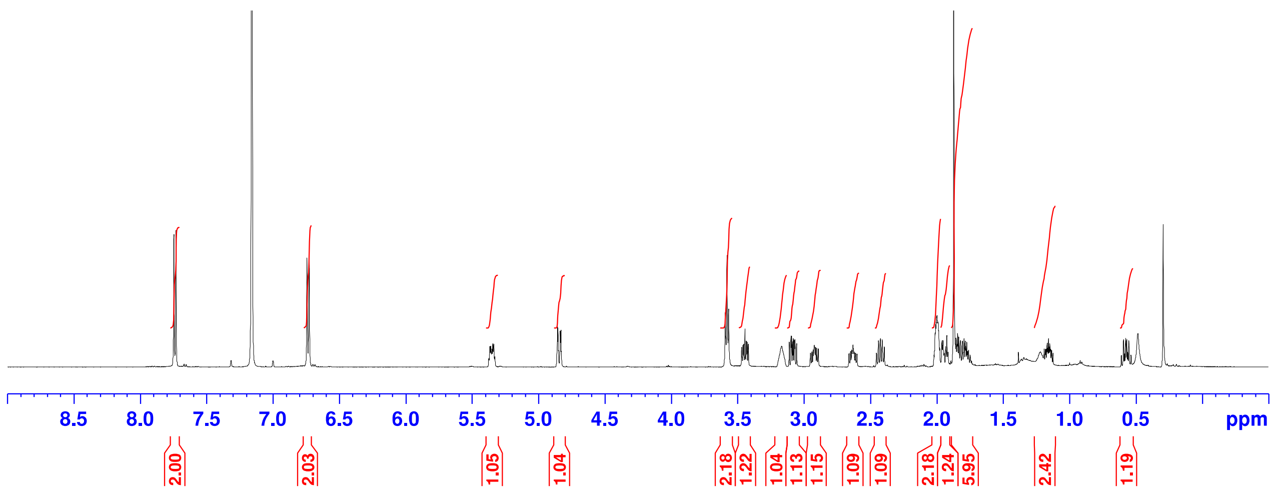

^{13}C NMR (125 MHz, C_6D_6)

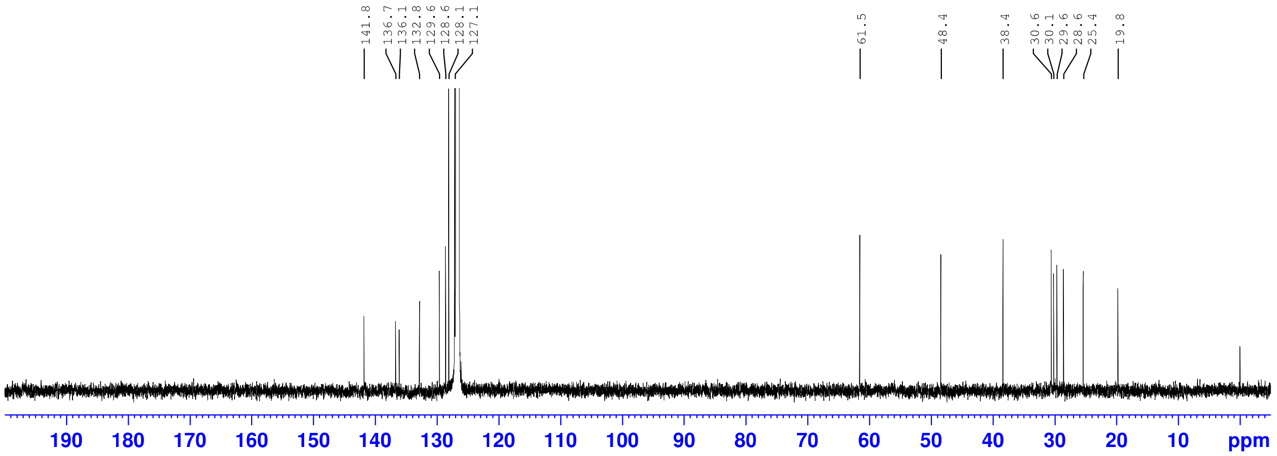

Supplementary Figure 37. ^{1}H and ^{13}C NMR spectra for compound **7g′**

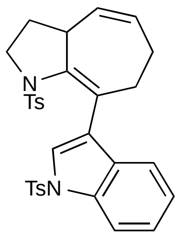

^{1}**H NMR** (500 MHz, C_6D_6)

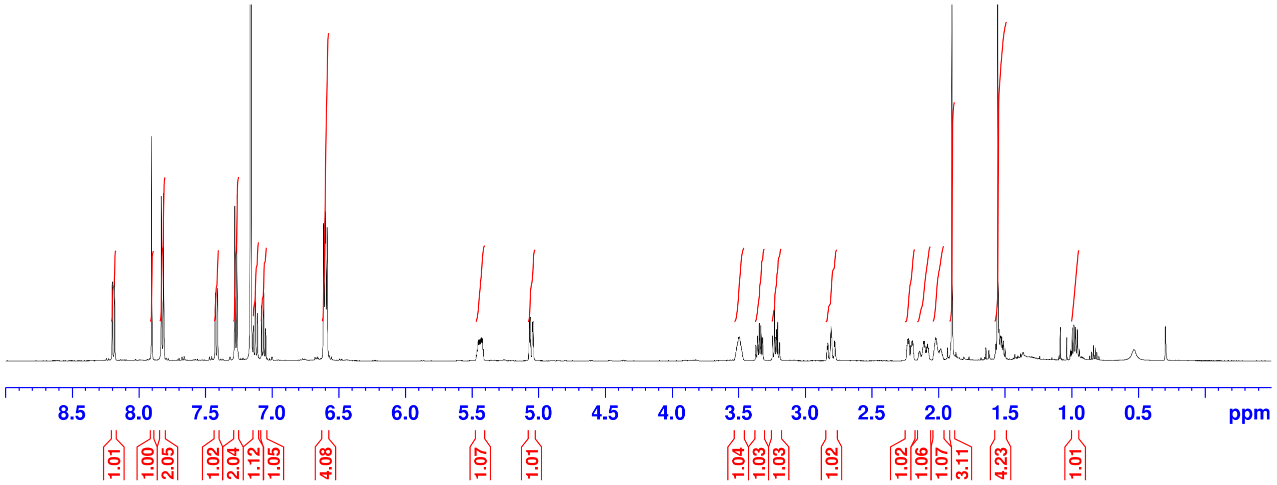

^{13}**C NMR** (125 MHz, C_6D_6)

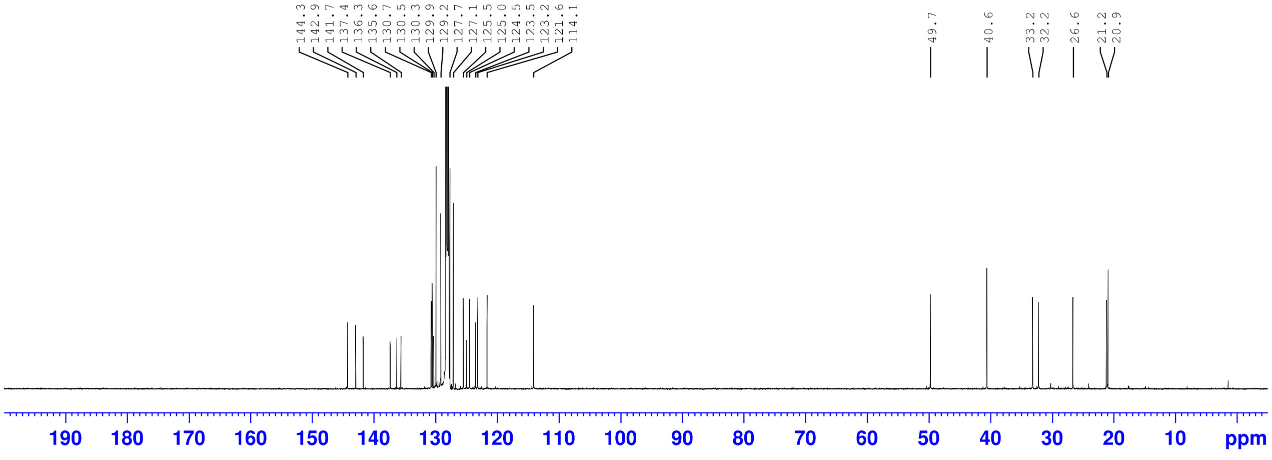

Supplementary Figure 38. ^{1}H and ^{13}C NMR spectra for compound **7h**

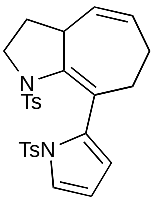

^{1}**H NMR** (500 MHz, C_6D_6)

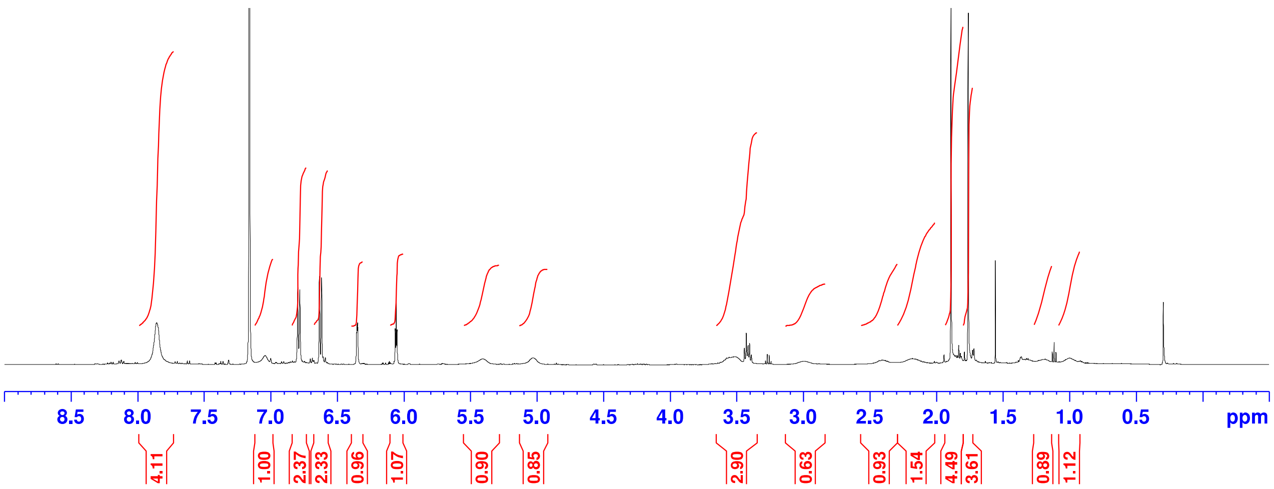

^{13}**C NMR** (125 MHz, C_6D_6)

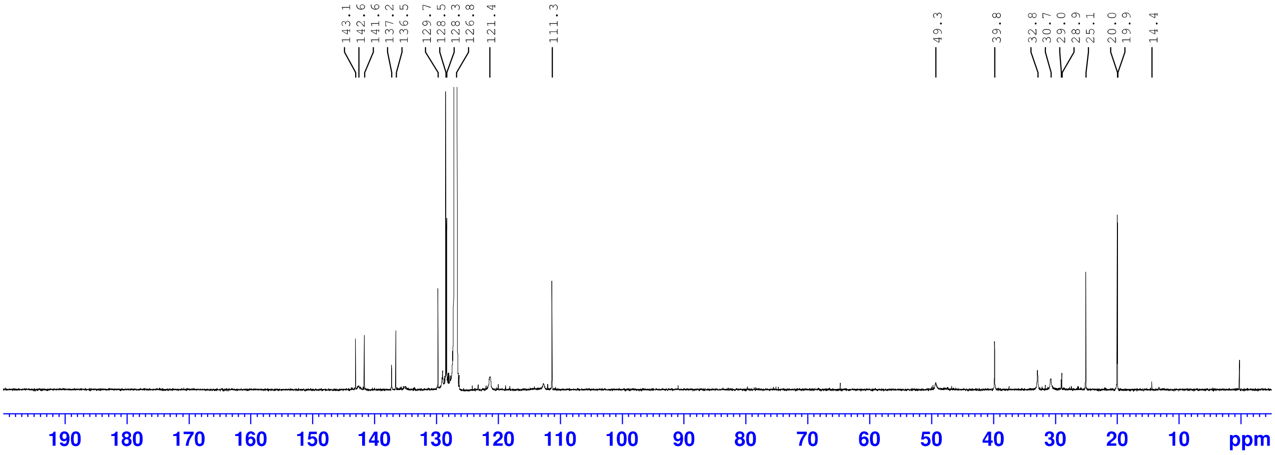

Supplementary Figure 39. ^{1}H and ^{13}C NMR spectra for compound **7i**. Some peaks in the proton and carbon NMR spectra are significantly broadened due to rotamer effects.

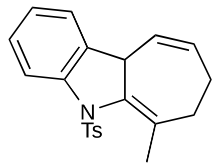

^{1}H NMR (500 MHz, C_6D_6)

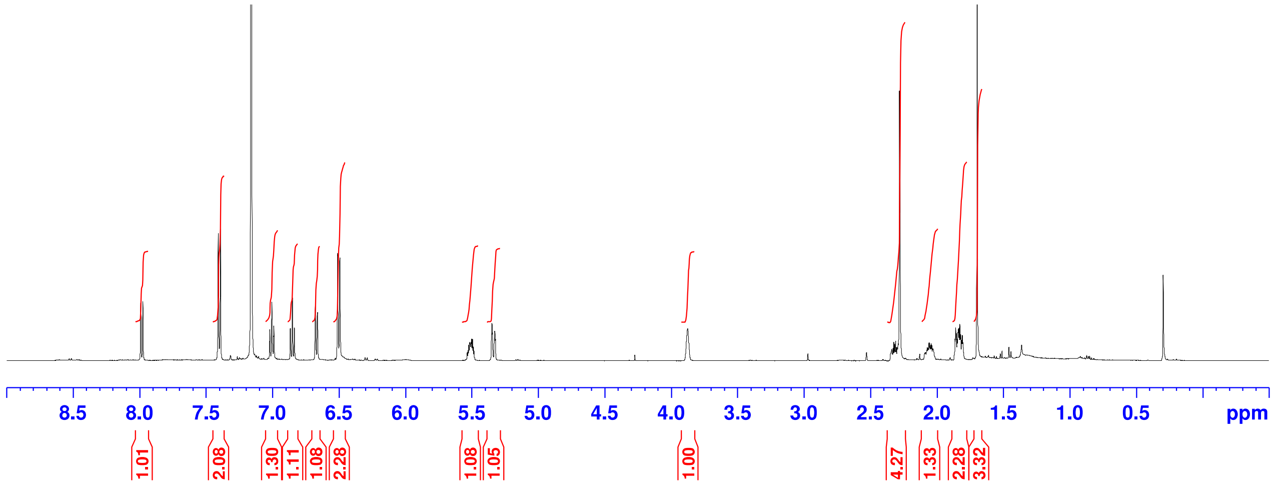

^{13}C NMR (125 MHz, C_6D_6)

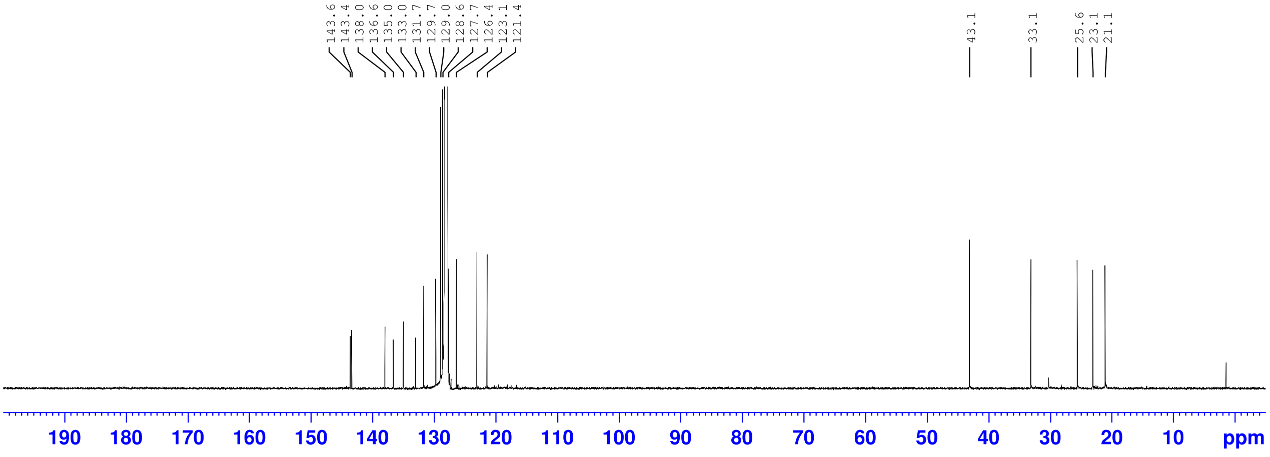

Supplementary Figure 40. ^{1}H and ^{13}C NMR spectra for compound **7j**

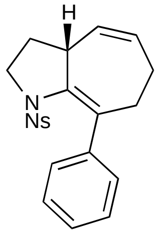

^{1}H NMR (500 MHz, C_6D_6)

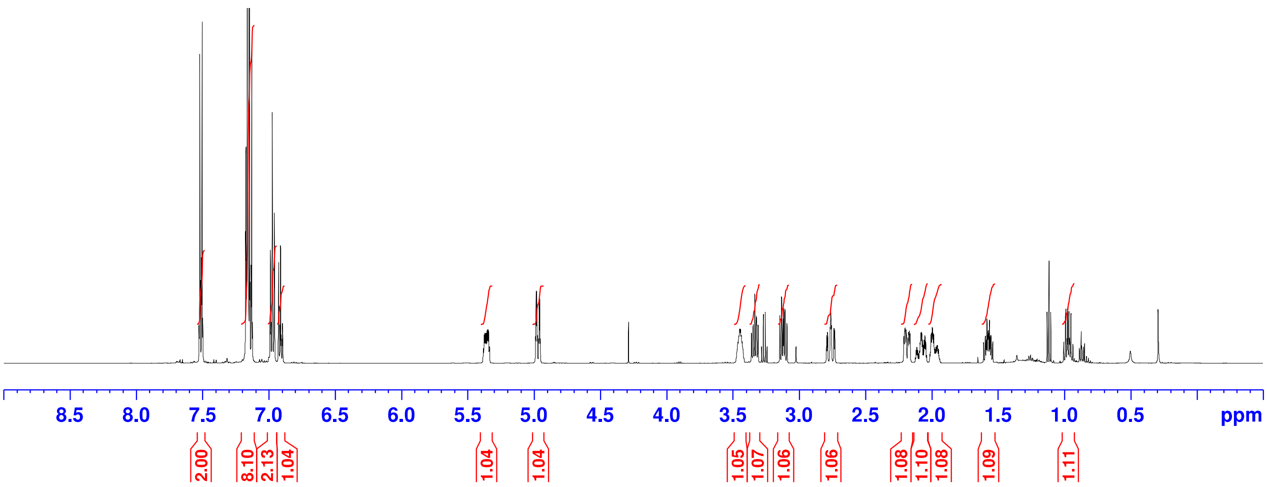

^{13}C NMR (125 MHz, C_6D_6)

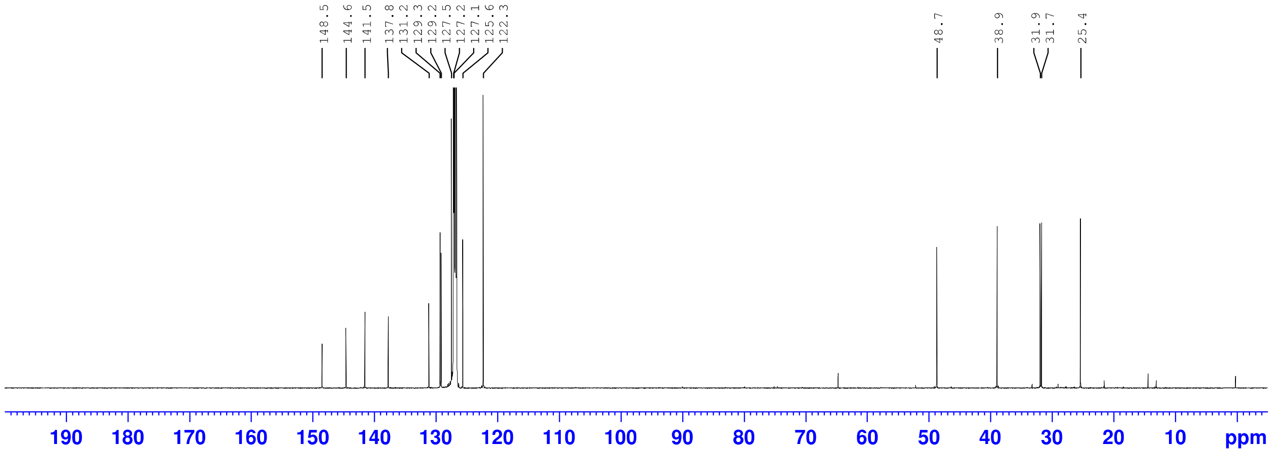

Supplementary Figure 41. ^{1}H and ^{13}C NMR spectra for compound **7k**

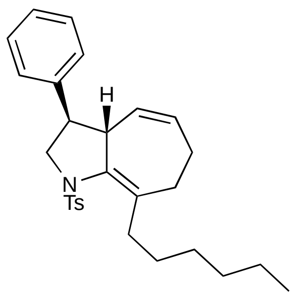

^{1}H NMR (500 MHz, C_6D_6)

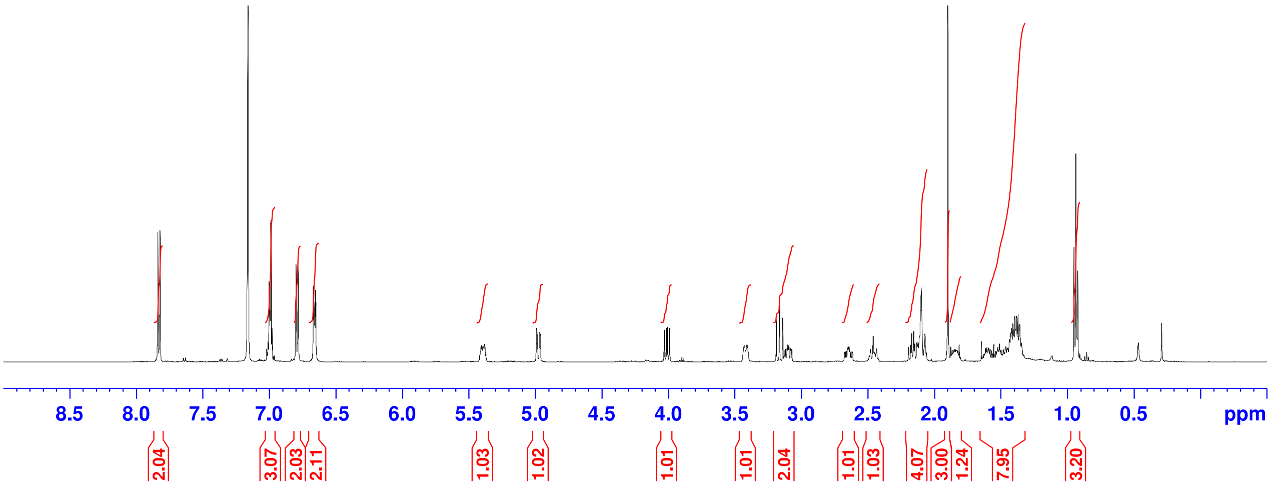

^{13}C NMR (125 MHz, C_6D_6)

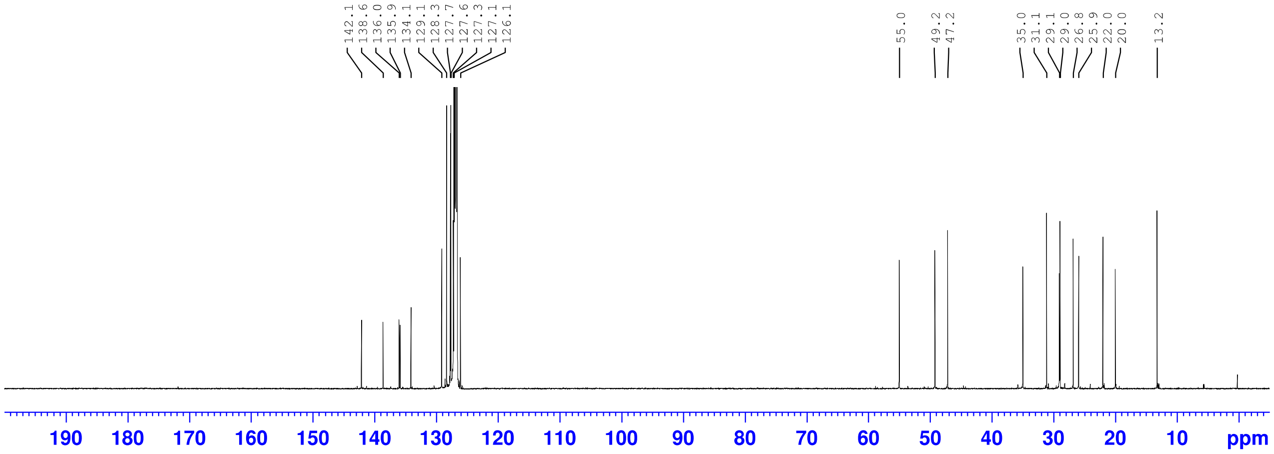

Supplementary Figure 42. ^{1}H and ^{13}C NMR spectra for compound **7l**

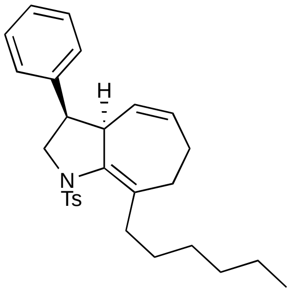

^{1}H NMR (500 MHz, C_6D_6)

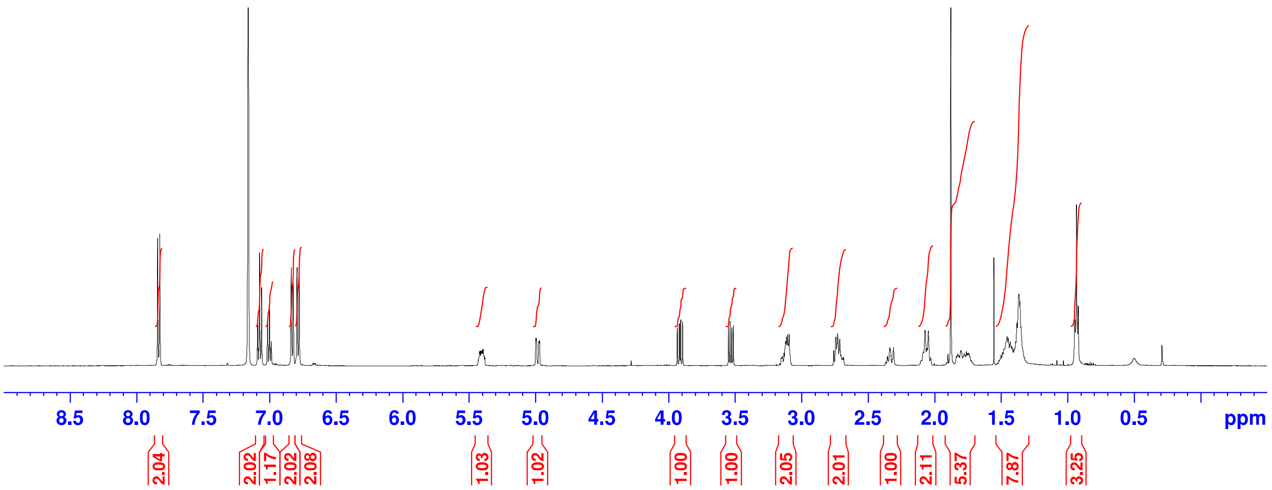

^{13}C NMR (125 MHz, C_6D_6)

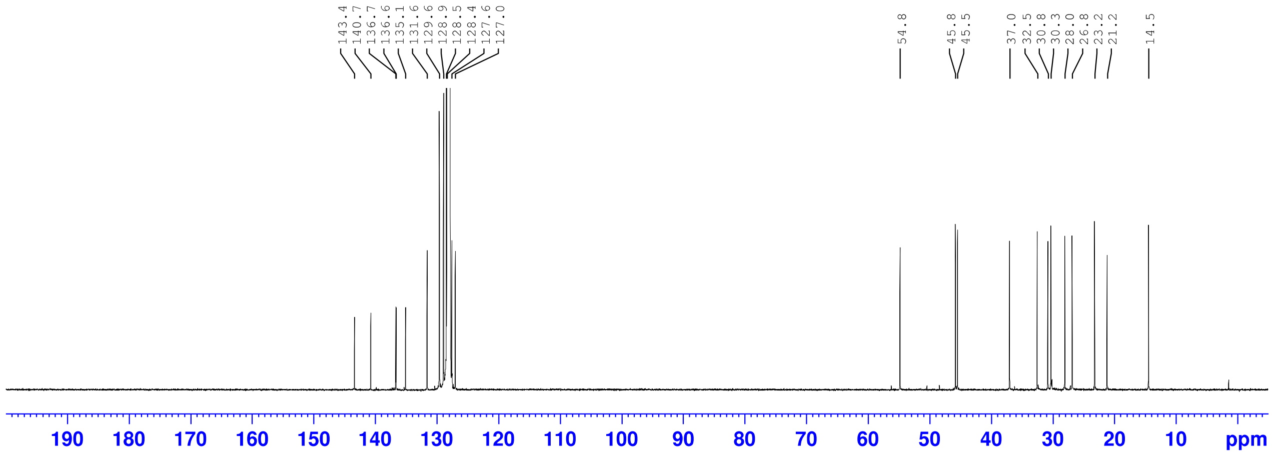

Supplementary Figure 43. ^{1}H and ^{13}C NMR spectra for compound **14l**

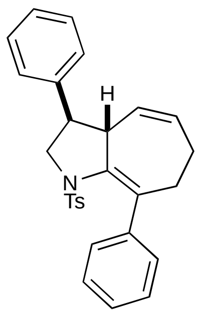

^{1}H NMR (500 MHz, C_6D_6)

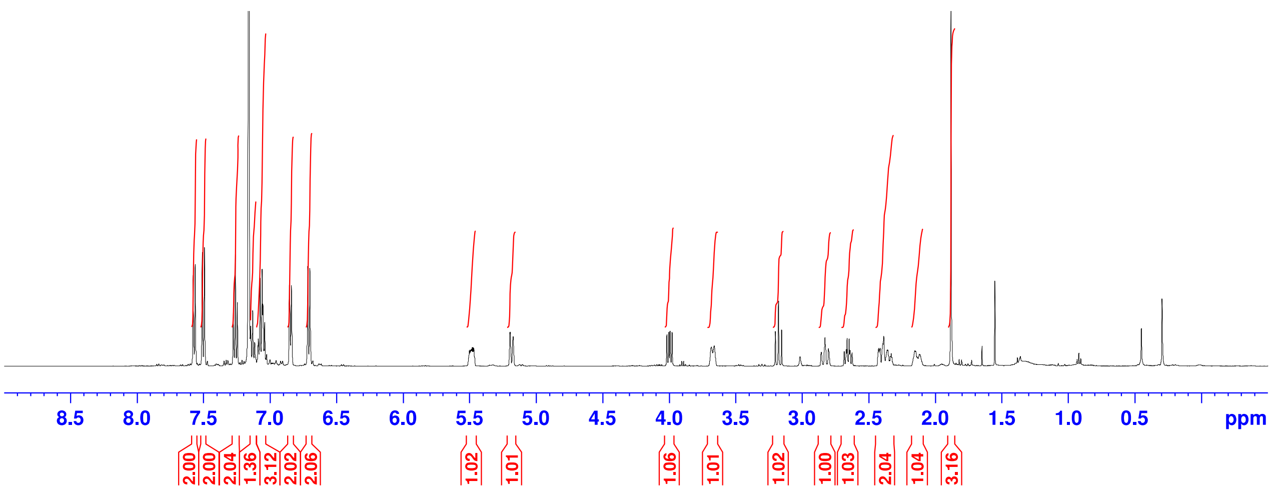

^{13}C NMR (125 MHz, C_6D_6)

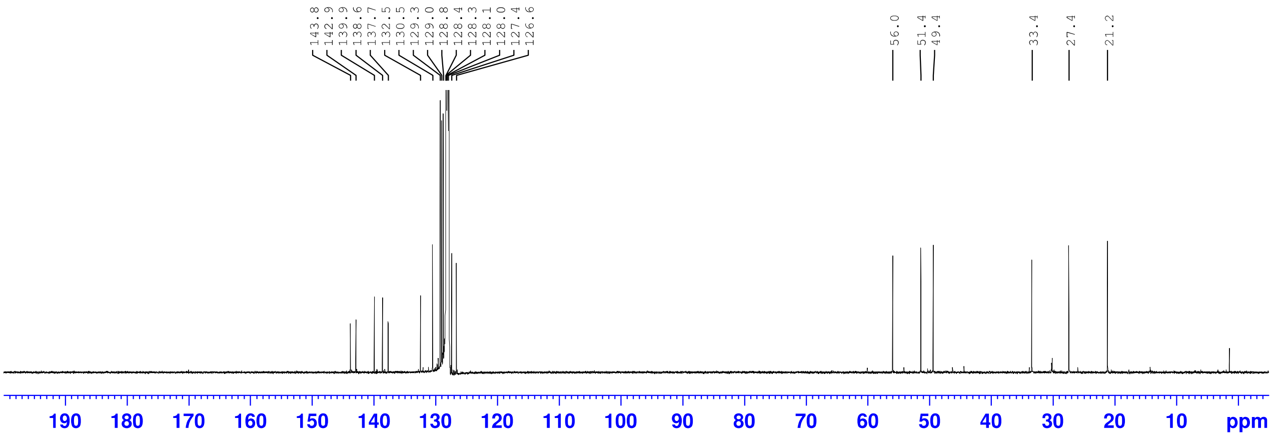

Supplementary Figure 44. ^{1}H and ^{13}C NMR spectra for compound **7m**

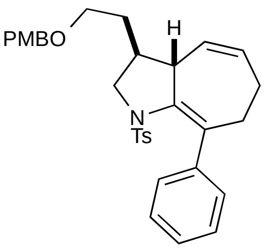

^{1}H NMR (500 MHz, C_6D_6)

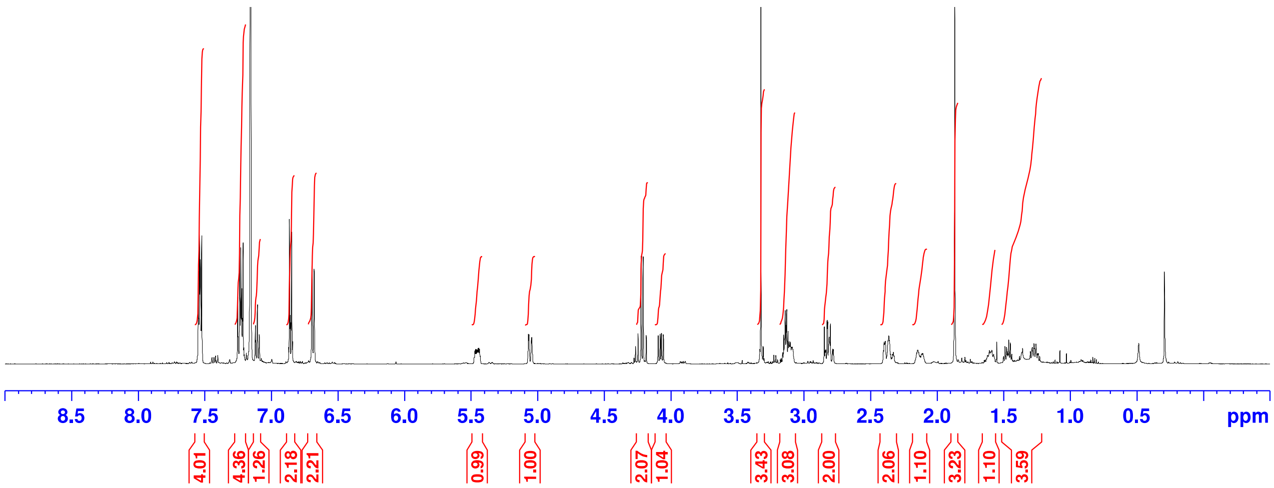

^{13}C NMR (125 MHz, C_6D_6)

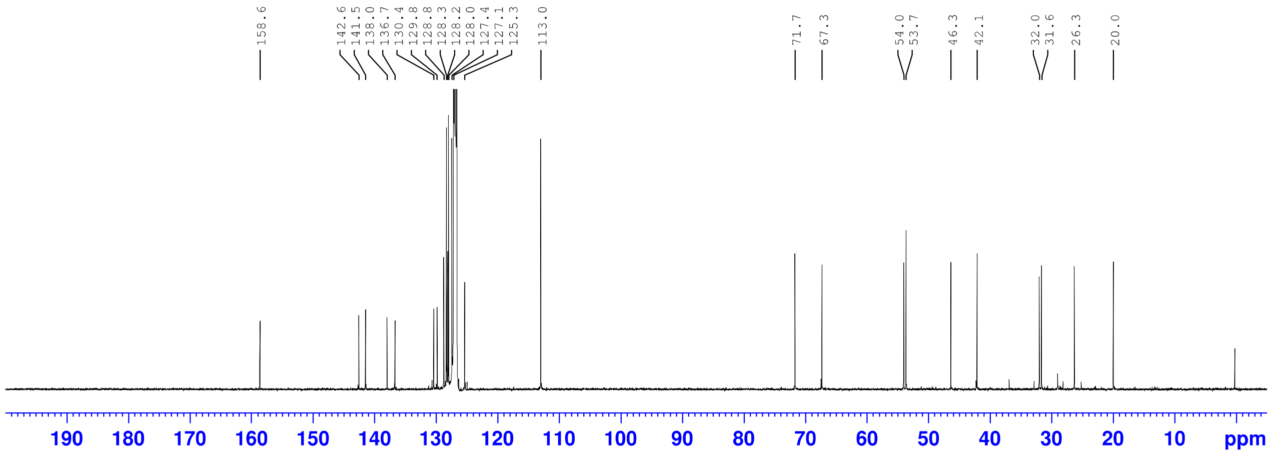

Supplementary Figure 45. ^{1}H and ^{13}C NMR spectra for compound **7n**

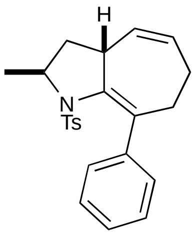

^{1}H NMR (500 MHz, C_6D_6)

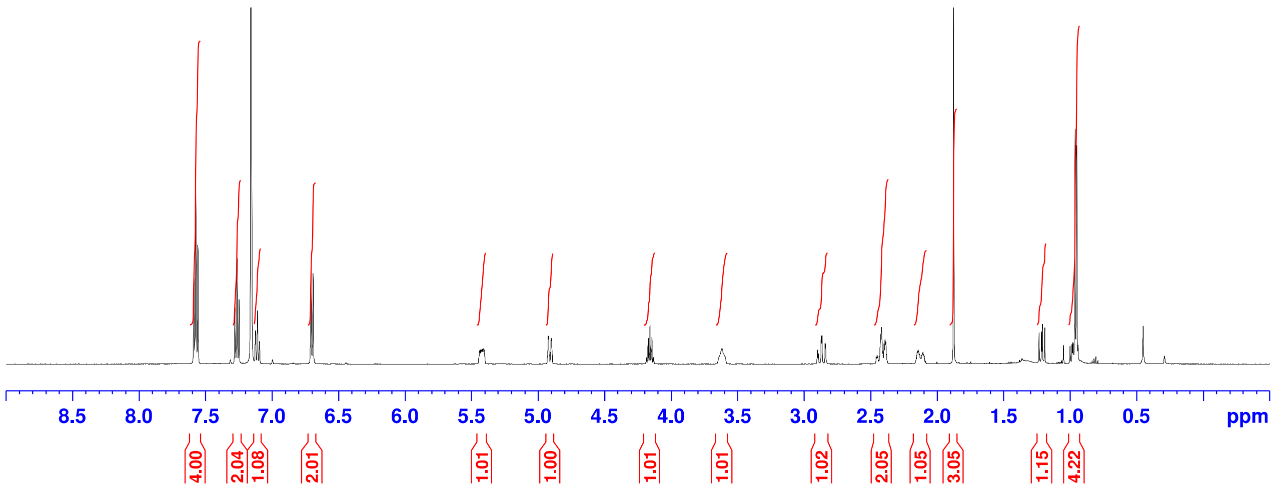

^{13}C NMR (125 MHz, C_6D_6)

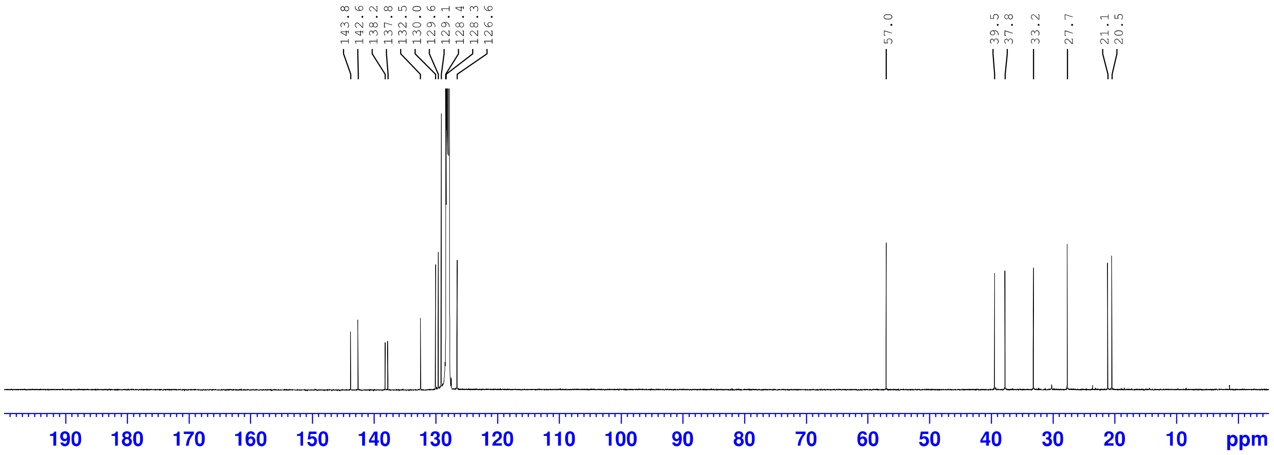

Supplementary Figure 46. ^{1}H and ^{13}C NMR spectra for compound **7o**

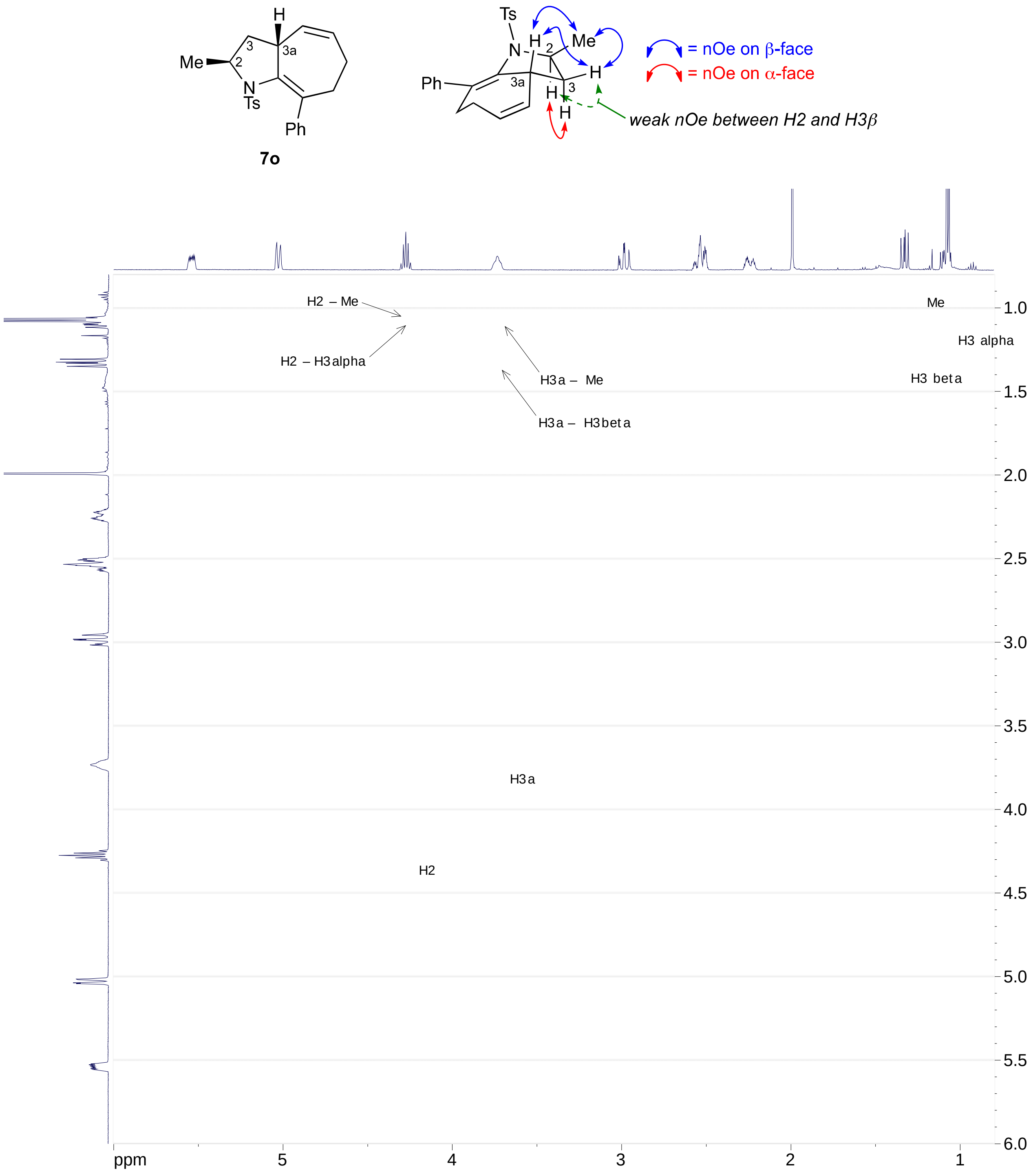

Supplementary Figure 47. NOESY spectrum for compound **7o**

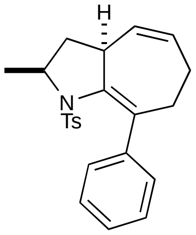

^{1}**H NMR** (500 MHz, C_6D_6)

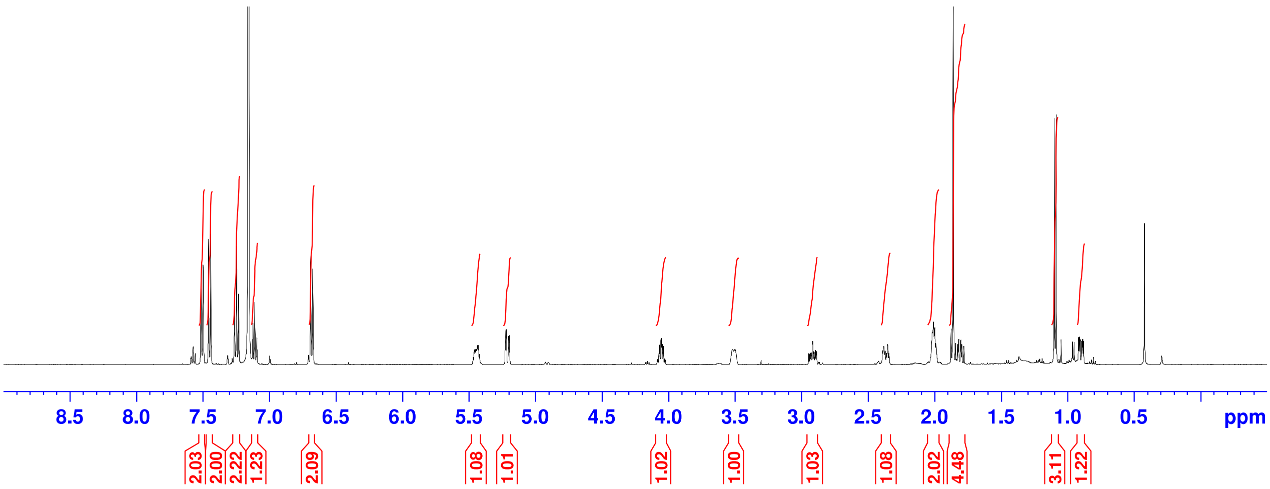

^{13}**C NMR** (125 MHz, C_6D_6)

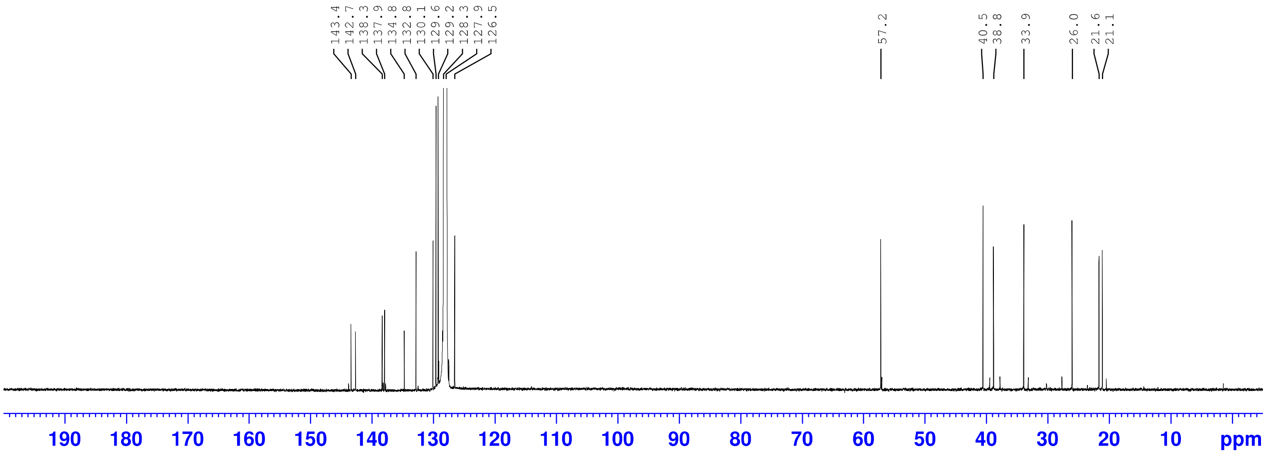

Supplementary Figure 48. ^{1}H and ^{13}C NMR spectra for compound **14o**

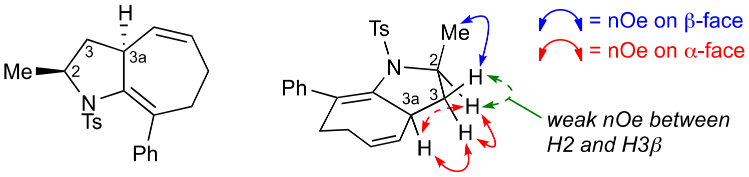

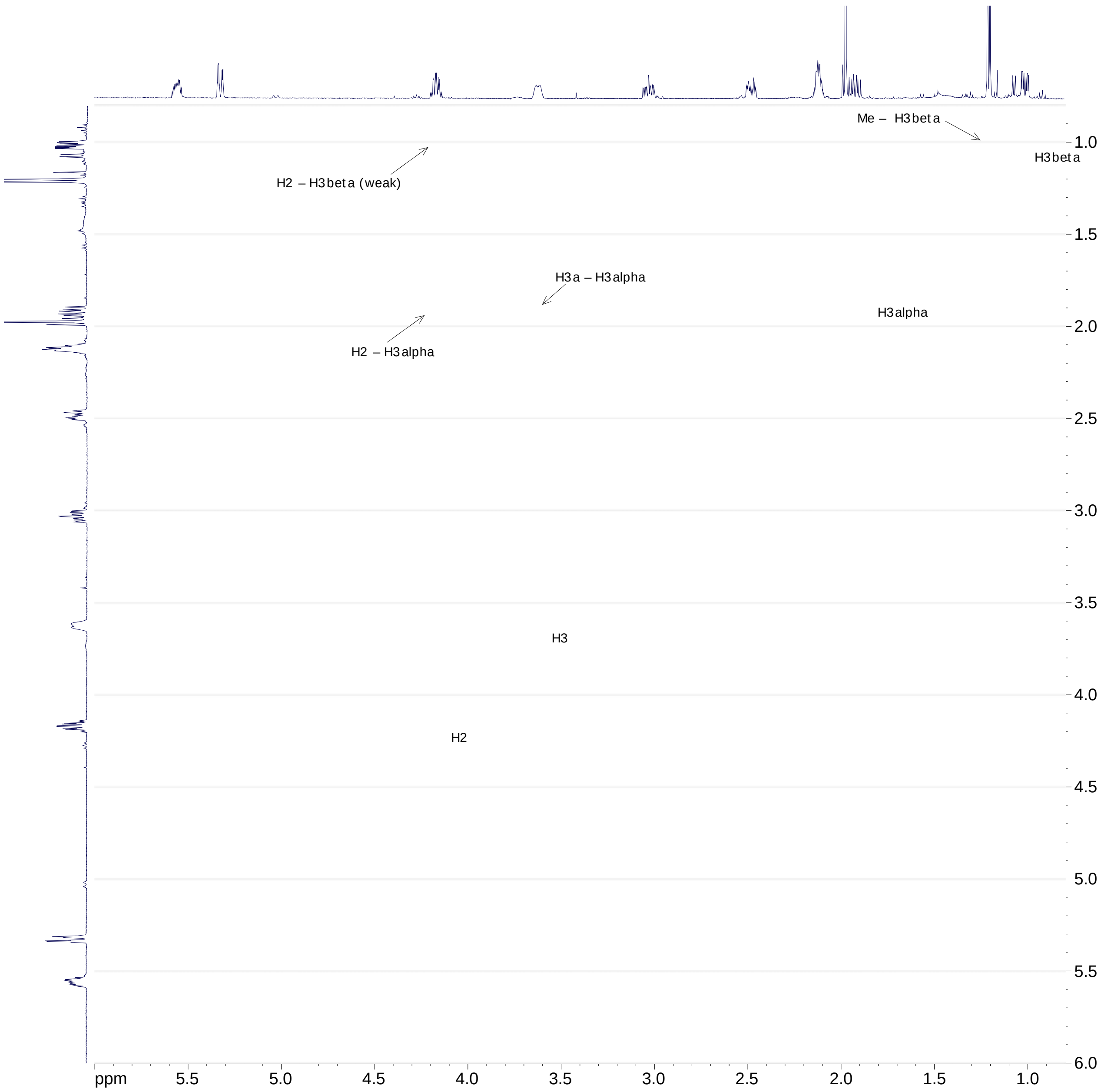

Supplementary Figure 49. NOESY spectrum for compound **14o**

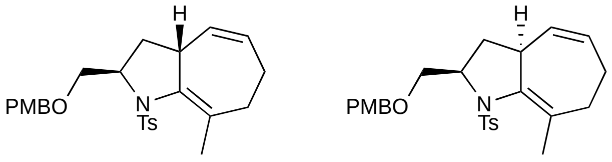

7p **14p**

^{1}H NMR (500 MHz, C_6D_6)

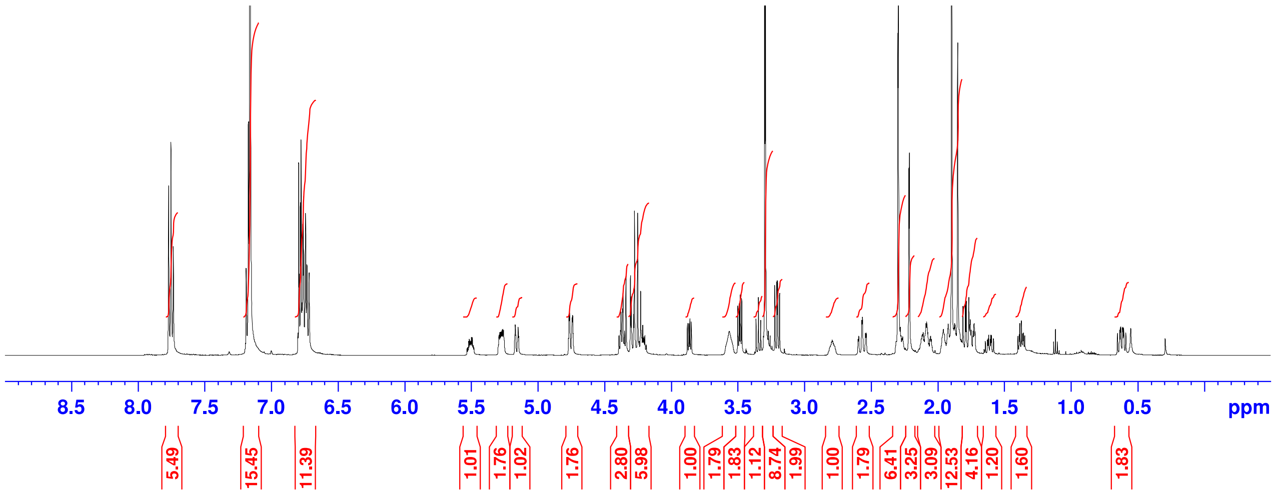

^{13}C NMR (125 MHz, C_6D_6)

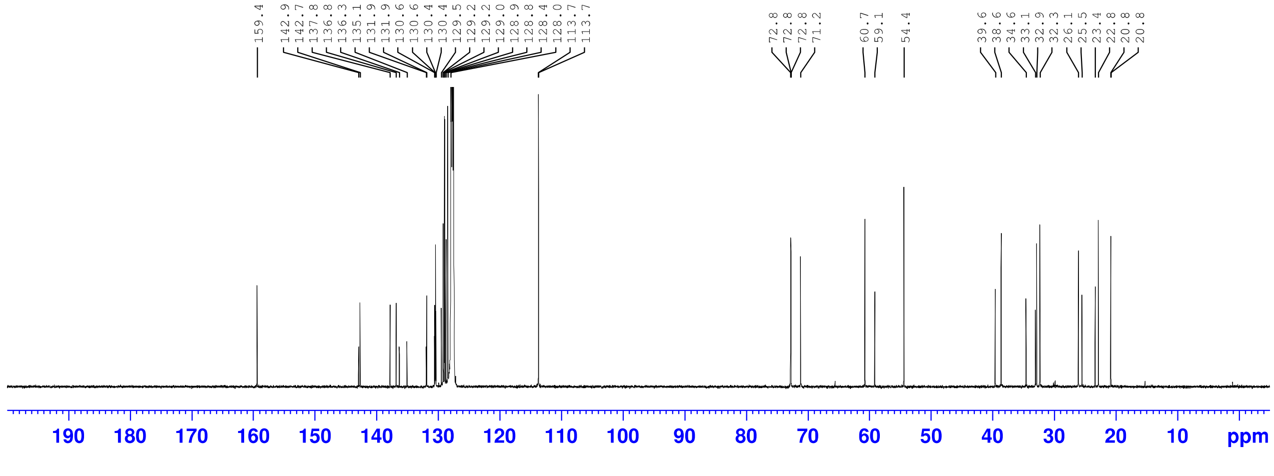

Supplementary Figure 50. ^{1}H and ^{13}C NMR spectra for compound **7p** and **14p**

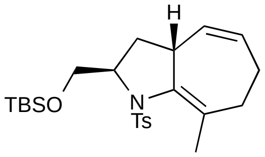

^{1}H NMR (500 MHz, C_6D_6)

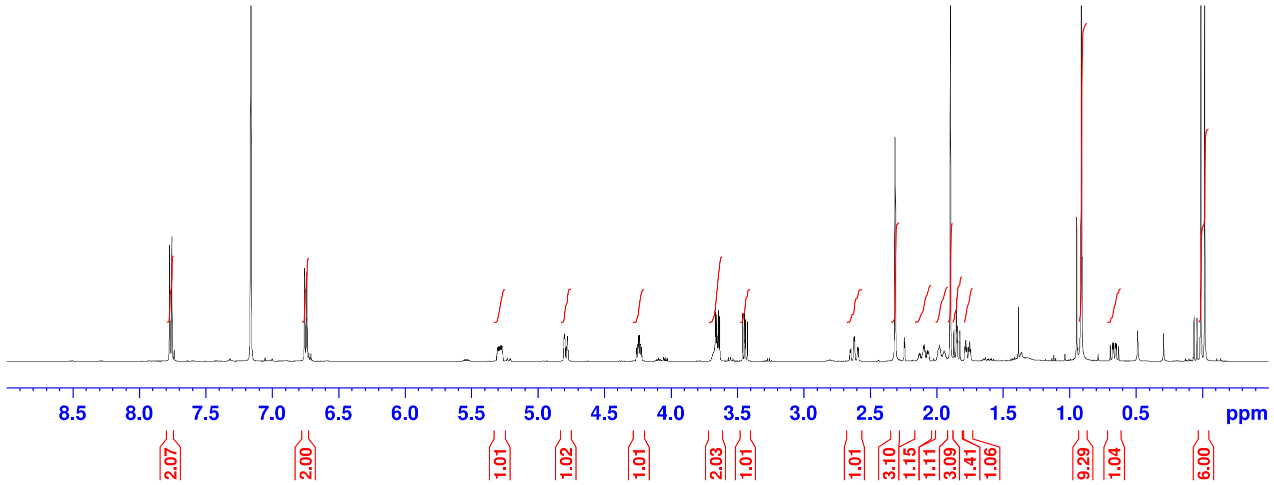

^{13}C NMR (125 MHz, C_6D_6)

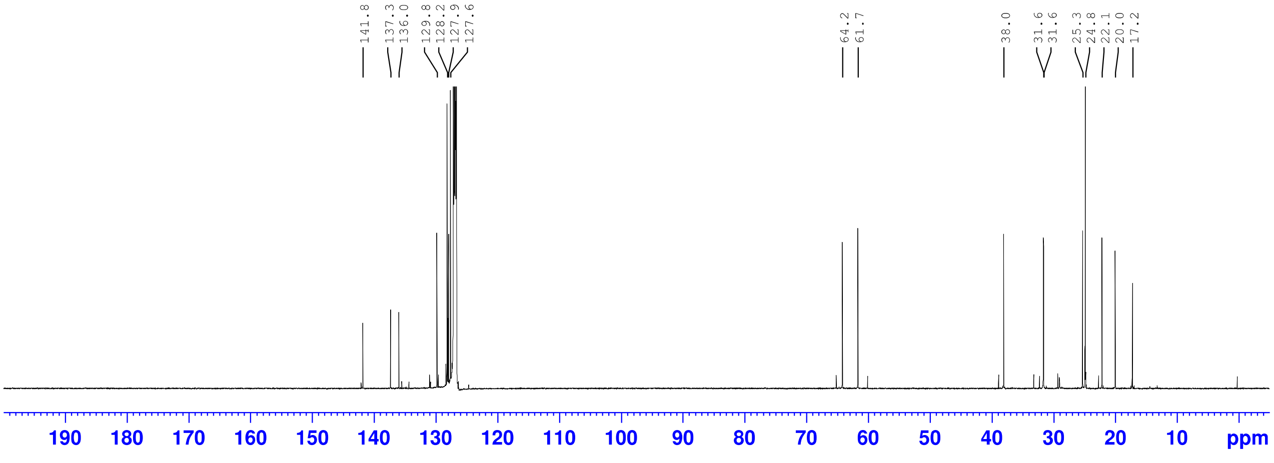

Supplementary Figure 51. ^{1}H and ^{13}C NMR spectra for compound **7q**

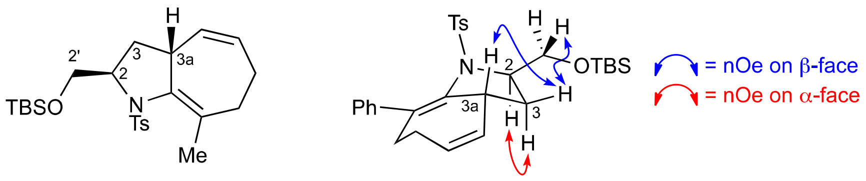

7q

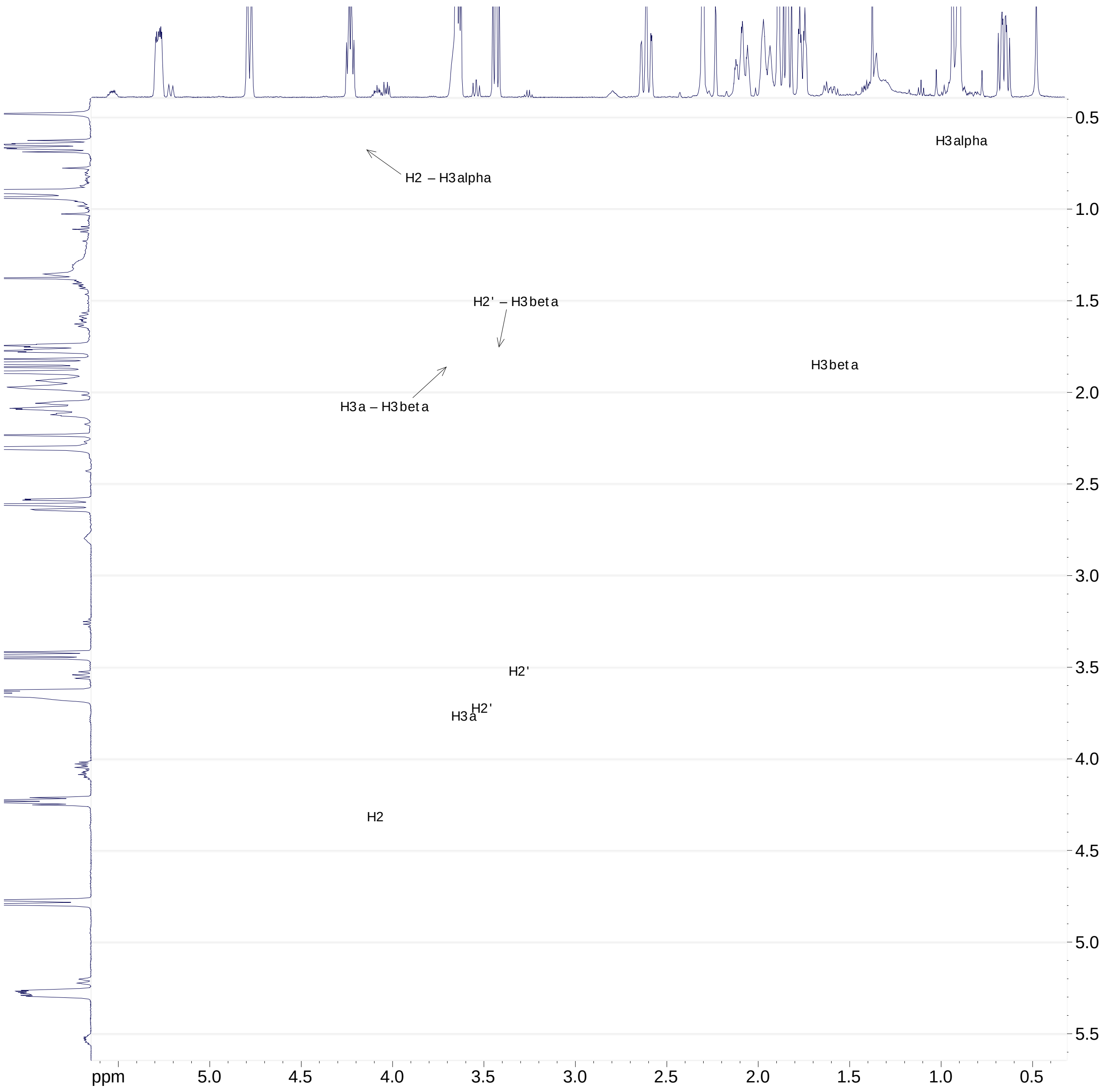

Supplementary Figure 52. NOESY spectrum for compound **7q**

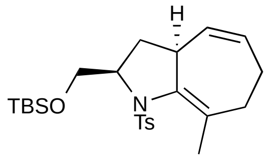

^{1}H NMR (500 MHz, C_6D_6)

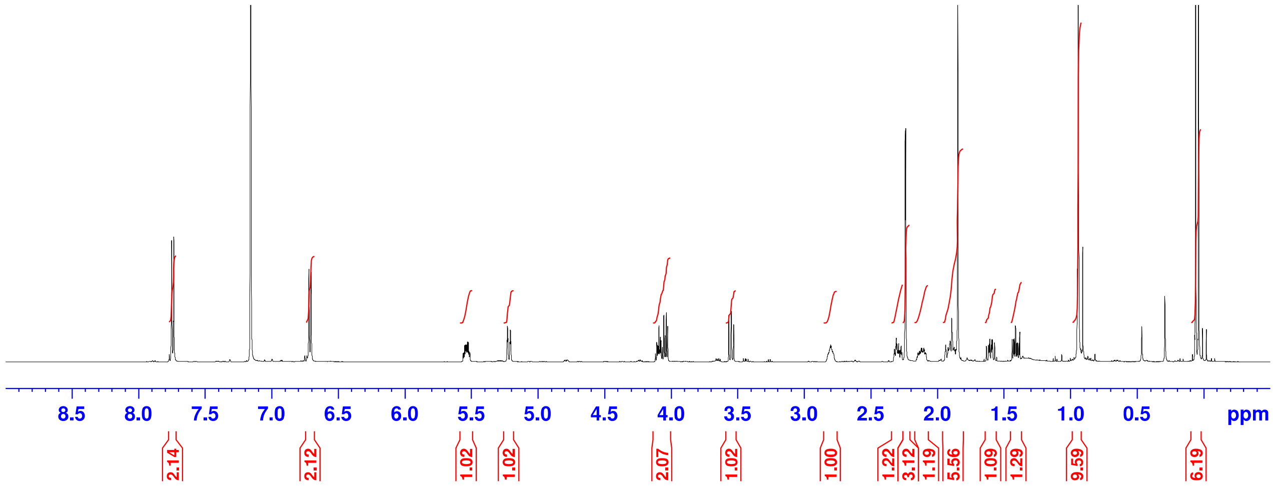

^{13}C NMR (125 MHz, C_6D_6)

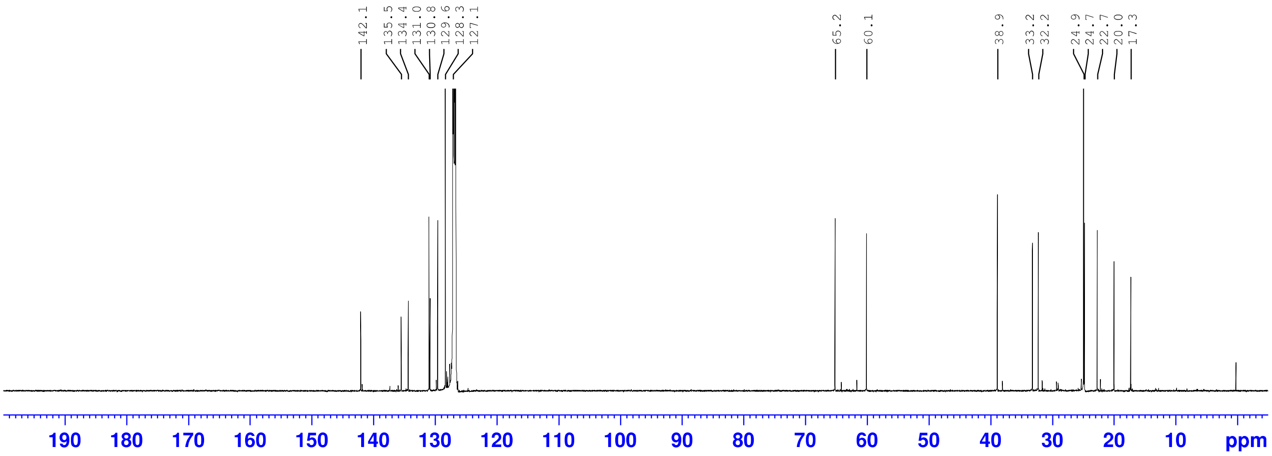

Supplementary Figure 53. ^{1}H and ^{13}C NMR spectra for compound **14q**

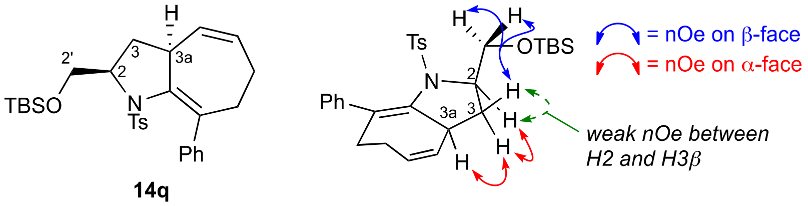

14q

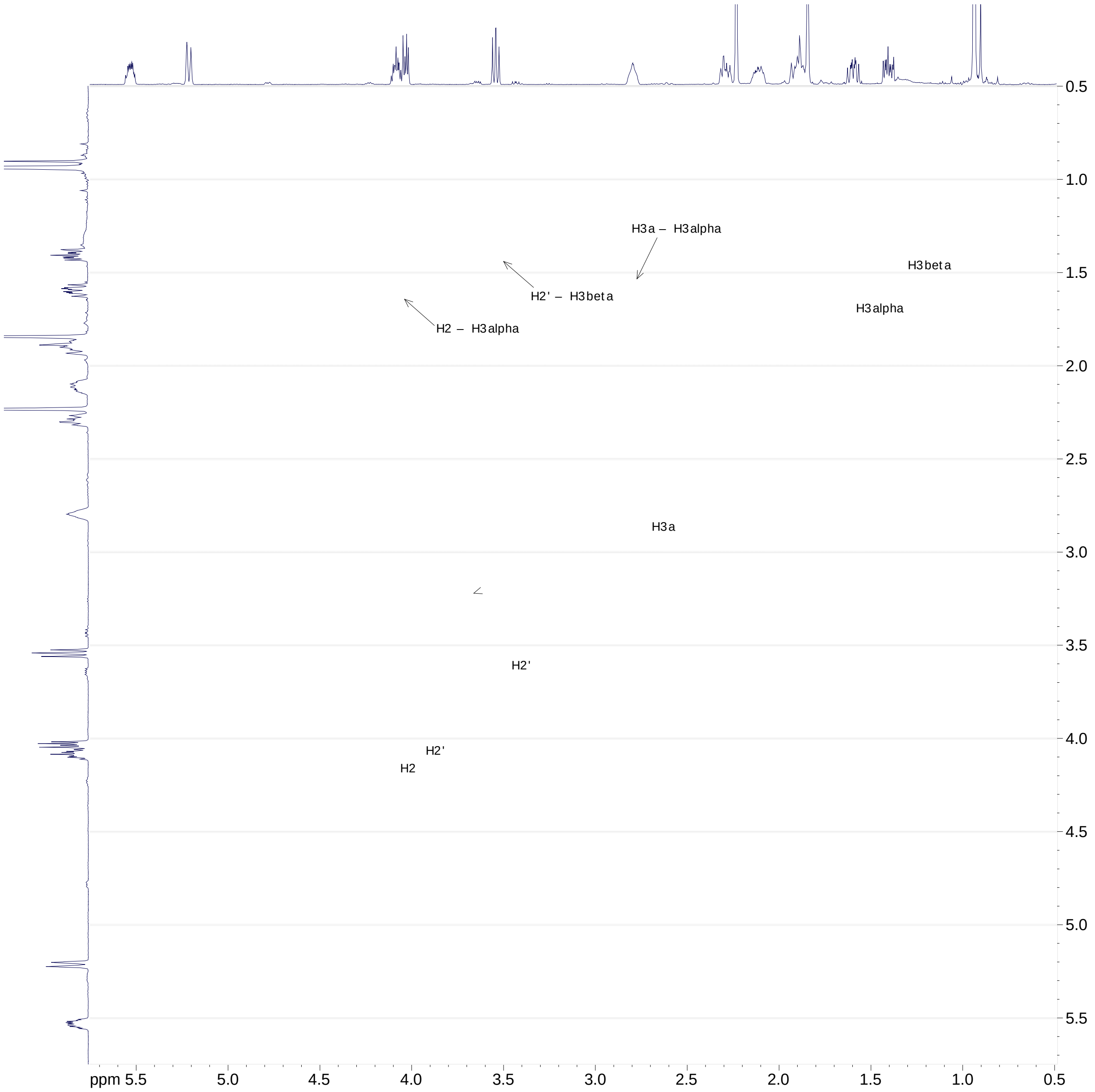

Supplementary Figure 54. NOESY spectrum for compound **14q**

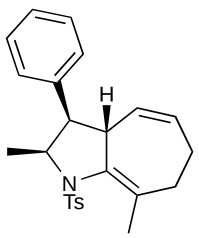

^{1}H NMR (500 MHz, C_6D_6)

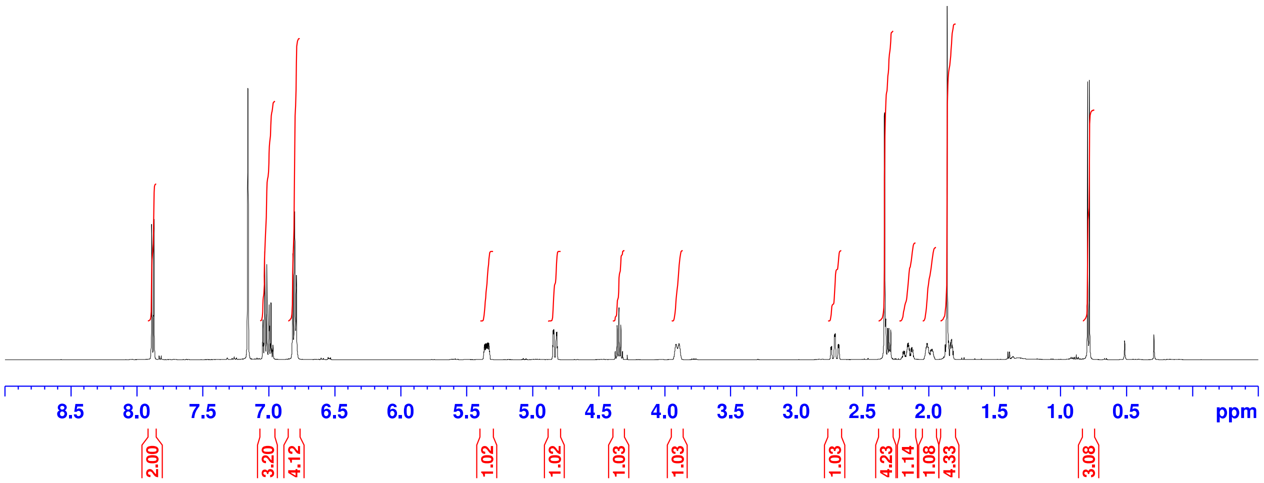

^{13}C NMR (125 MHz, C_6D_6)

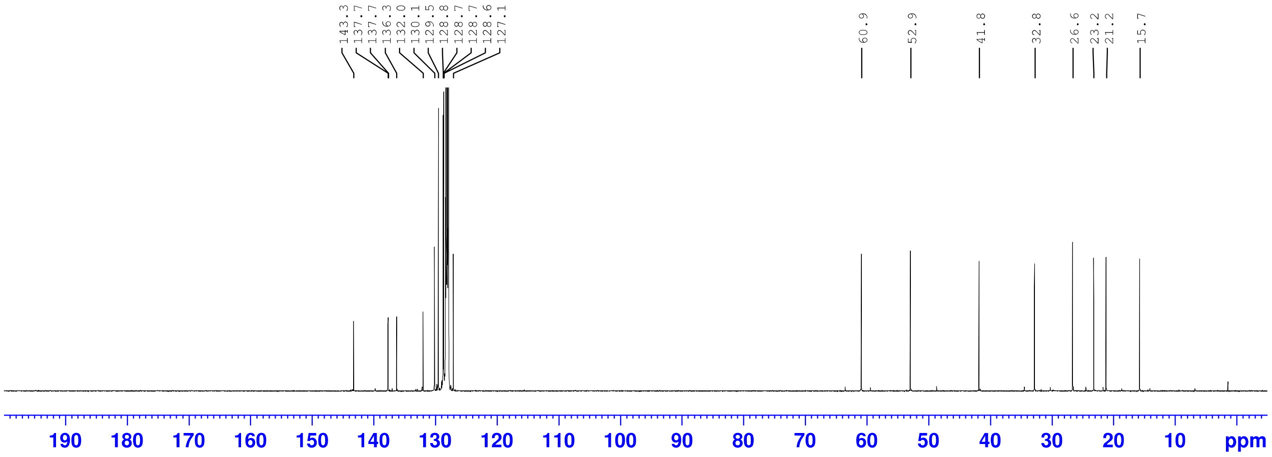

Supplementary Figure 55. ^{1}H and ^{13}C NMR spectra for compound **7r**

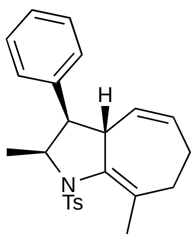

7r

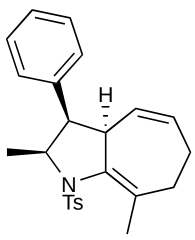

14r

^{1}H NMR (500 MHz, C_6D_6)

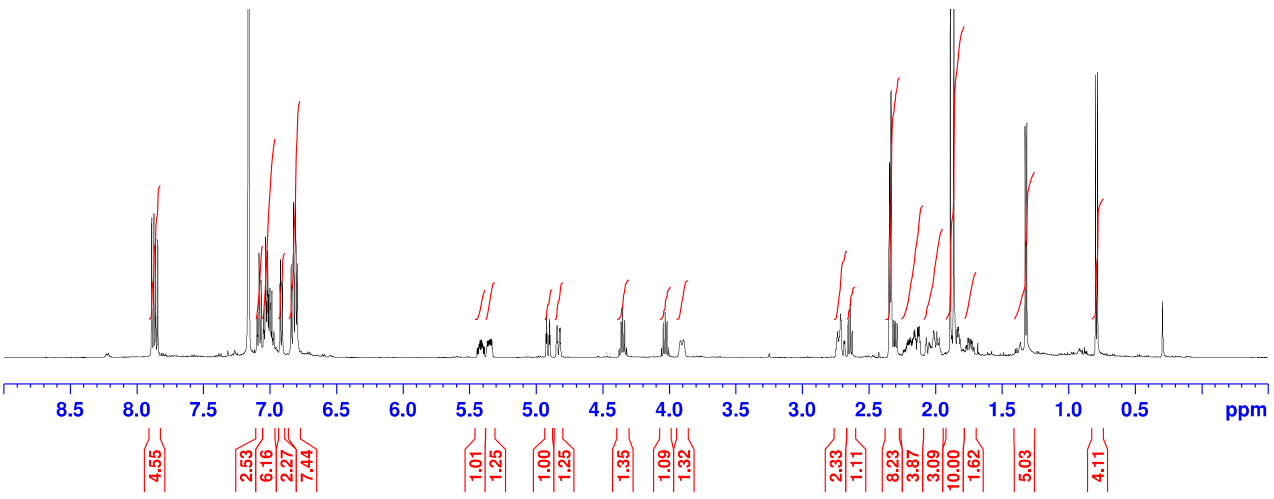

^{13}C NMR (125 MHz, C_6D_6)

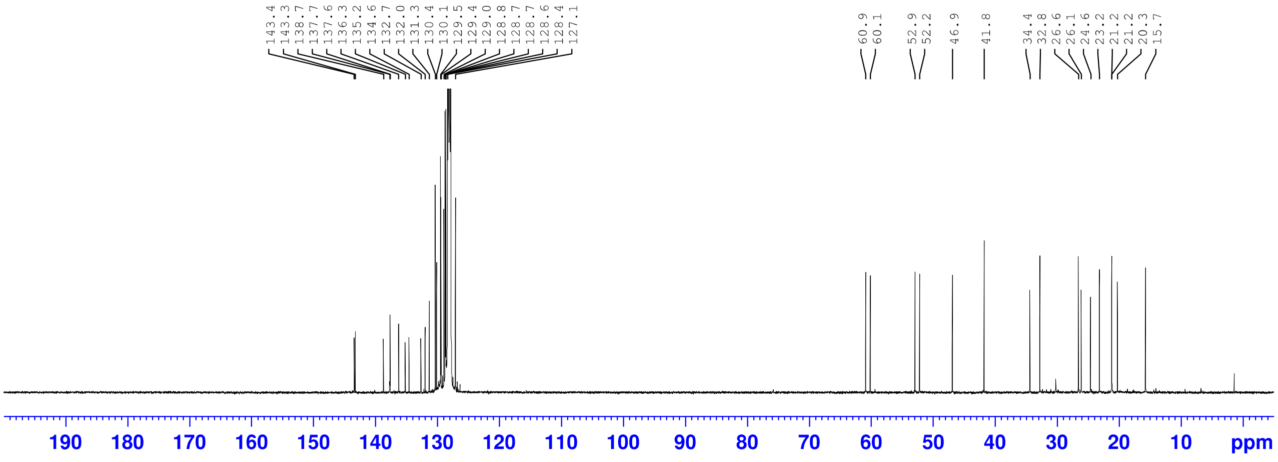

Supplementary Figure 56. ^{1}H and ^{13}C NMR spectra for compound **7r** and **14r**

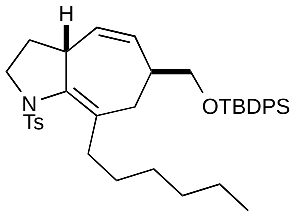

^{1}H NMR (500 MHz, C_6D_6)

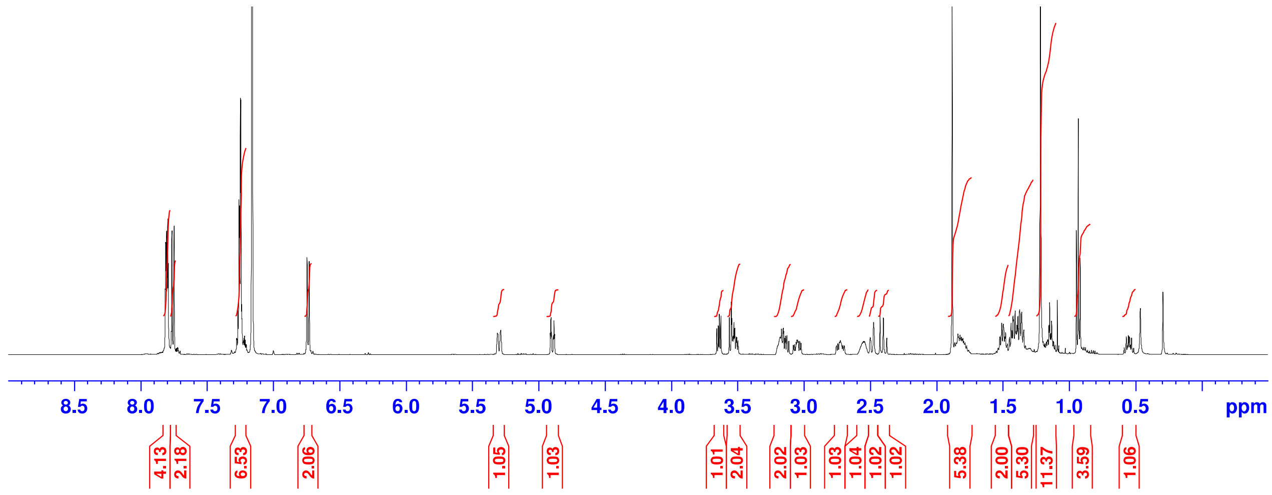

^{13}C NMR (125 MHz, C_6D_6)

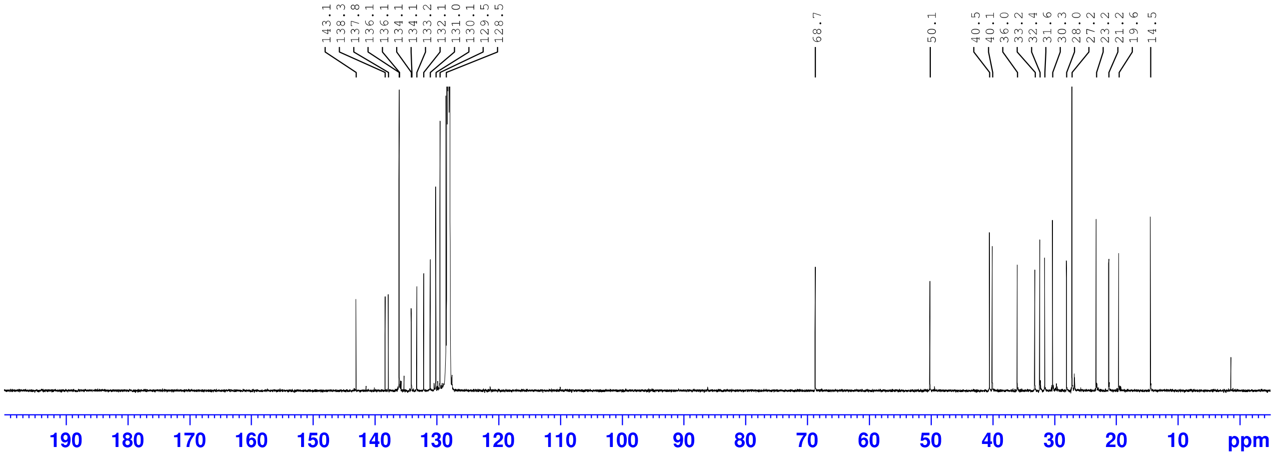

Supplementary Figure 57. ^{1}H and ^{13}C NMR spectra for compound **7s**

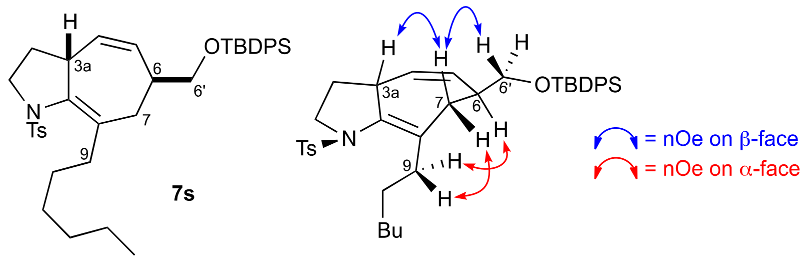

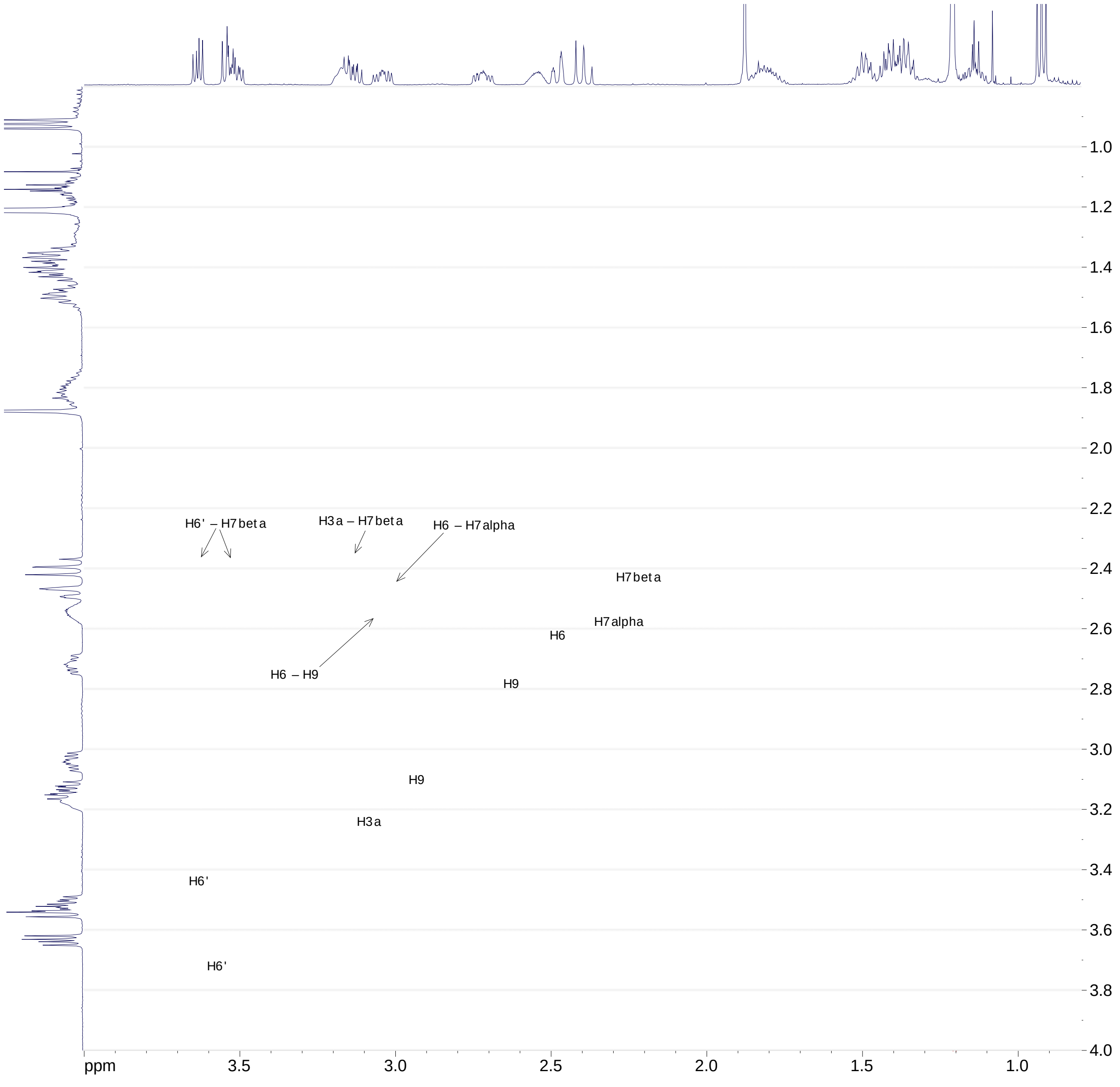

Supplementary Figure 58. NOESY spectrum for compound **7s**

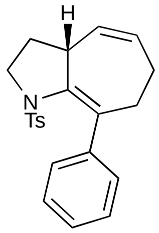

Chiralpak IA (5% IPA in hexane, flow rate = 1.3 mL/min, 254 nm) t_R major – 12.2 min, minor – 13.7 min

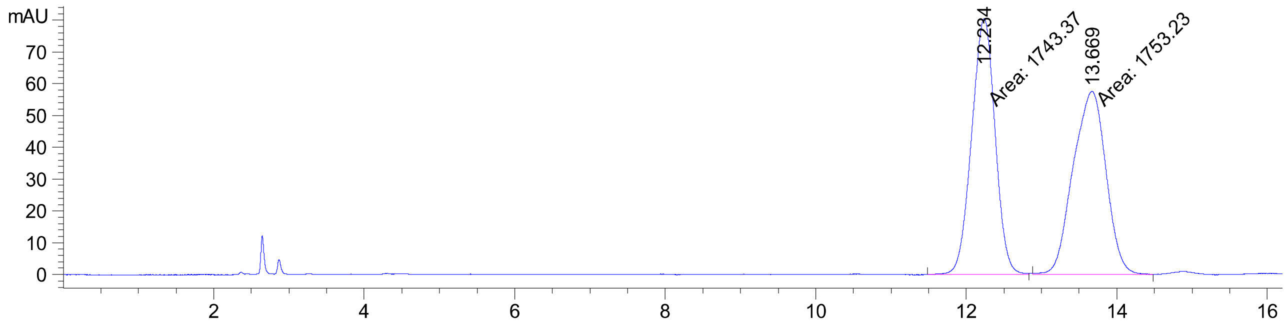

Peak #	RetTime [min]	Type	Width [min]	Area [mAU*s]	Height [mAU]	Area %
1	12.234	MM T	0.3613	1743.37268	80.41655	49.8591
2	13.669	MM T	0.5074	1753.22852	57.58450	50.1409

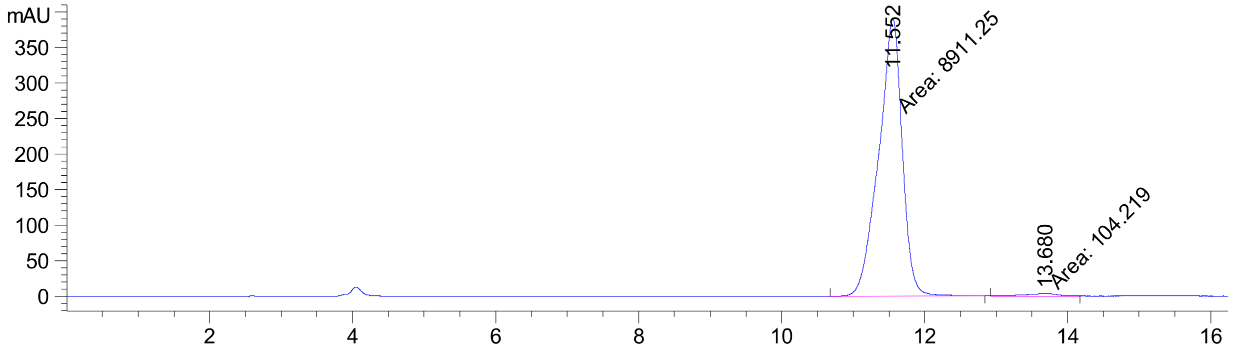

Peak #	RetTime [min]	Type	Width [min]	Area [mAU*s]	Height [mAU]	Area %
1	11.552	MM	0.3800	8911.24609	390.89105	98.8440
2	13.680	MM	0.5213	104.21923	3.33188	1.1560

Supplementary Figure 59. HPLC traces for compound **7a**

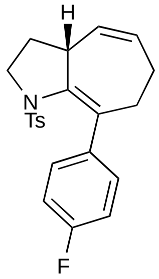

Chiralpak IA (5% IPA in hexane, flow rate = 1.3 mL/min, 254 nm) t_R major – 9.9 min, minor – 10.7 min

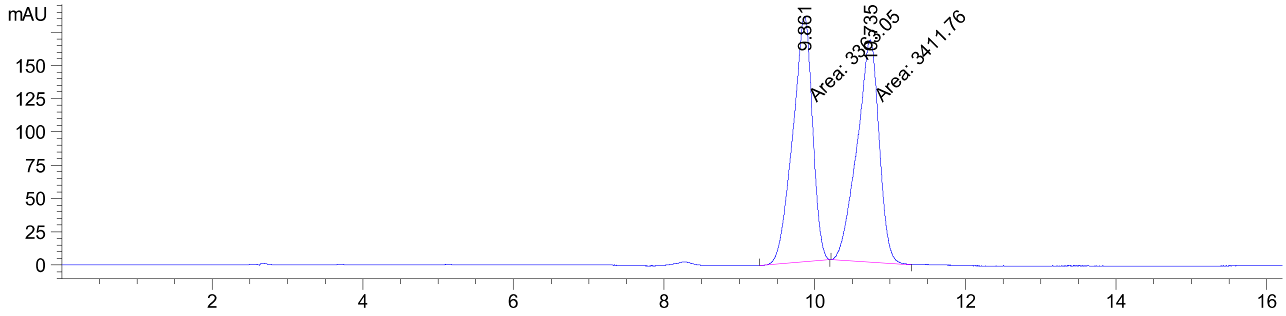

Peak #	RetTime [min]	Type	Width [min]	Area [mAU*s]	Height [mAU]	Area %
1	9.861	MM T	0.4148	3363.05225	184.37761	49.6406
2	10.735	MM T	0.3403	3411.75610	167.10854	50.3594

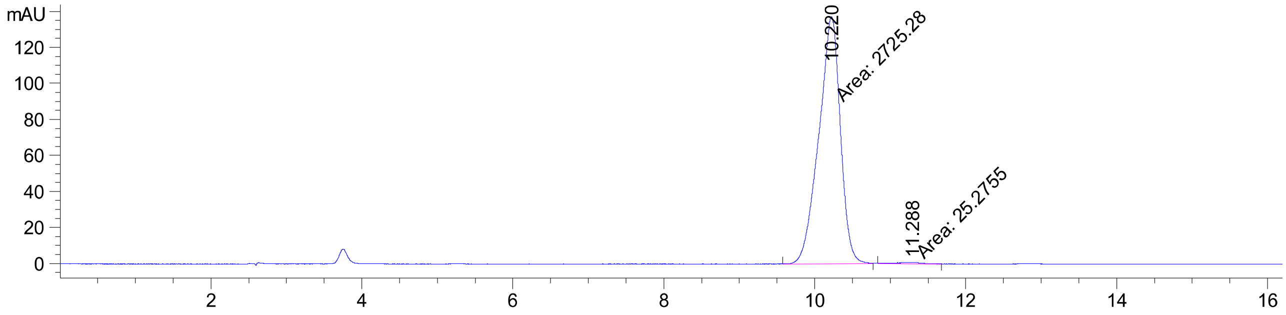

Peak #	RetTime [min]	Type	Width [min]	Area [mAU*s]	Height [mAU]	Area %
1	10.220	MM T	0.4493	2725.27783	137.23038	99.0811
2	11.288	MM T	0.4609	25.27553	9.14000e-1	0.9189

Supplementary Figure 60. HPLC traces for compound **7b**

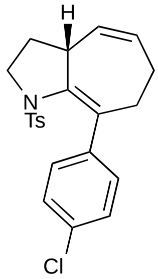

Chiralpak IA (5% IPA in hexane, flow rate = 1.3 mL/min, 254 nm) t_R major – 10.5 min, minor – 12.2 min

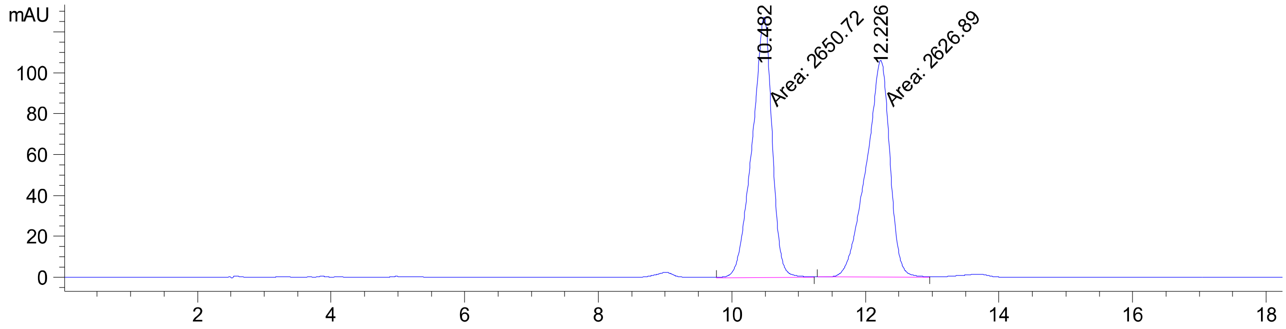

Peak #	RetTime [min]	Type	Width [min]	Area [mAU*s]	Height [mAU]	Area %
1	10.482	MM T	0.3466	2650.72339	127.47778	50.2258
2	12.226	MM T	0.6089	2626.89282	106.02496	49.7742

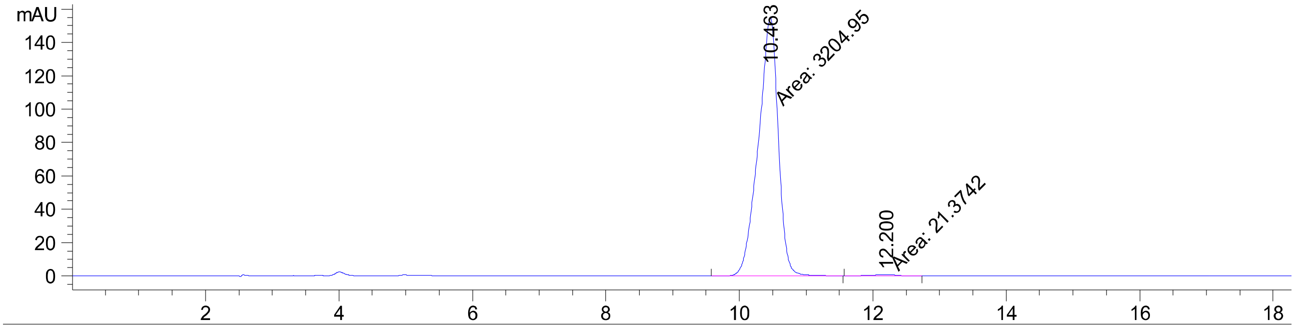

Peak #	RetTime [min]	Type	Width [min]	Area [mAU*s]	Height [mAU]	Area %
1	10.463	MM	0.3441	3204.94580	155.22279	99.3375
2	12.200	MM	0.4114	21.37419	8.65818e-1	0.6625

Supplementary Figure 61. HPLC traces for compound **7c**

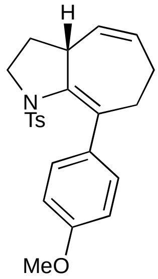

Chiralpak IC (30% IPA in hexane, flow rate = 1.0 mL/min, 254 nm) t_R major – 39.0 min, minor – 51.5 min

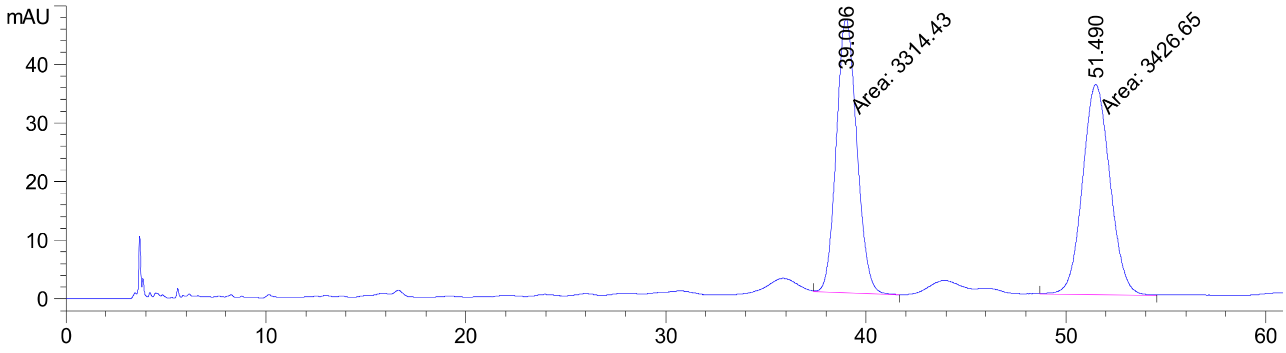

Peak #	RetTime [min]	Type	Width [min]	Area [mAU*s]	Height [mAU]	Area %
1	39.006	MM	1.1849	3314.42700	46.62084	49.1677
2	51.490	MM	1.5943	3426.64502	35.82107	50.8323

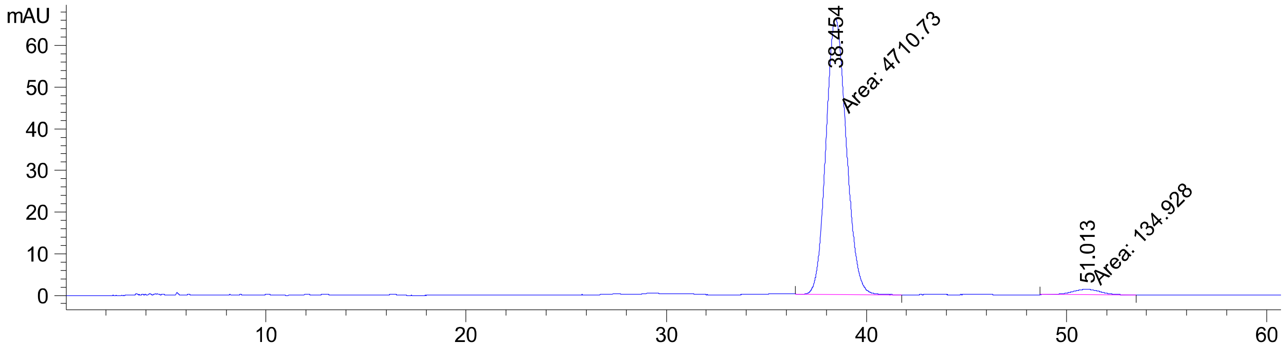

Peak #	RetTime [min]	Type	Width [min]	Area [mAU*s]	Height [mAU]	Area %
1	38.454	MM	1.1827	4710.73096	66.38640	97.2155
2	51.013	MM	1.6743	134.92757	1.34315	2.7845

Supplementary Figure 62. HPLC traces for compound **7d**

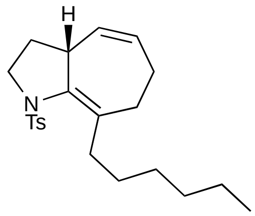

Chiralpak IA (2% IPA in hexane, flow rate = 1.3 mL/min, 254 nm) t_R major – 7.0 min, minor – 7.7 min

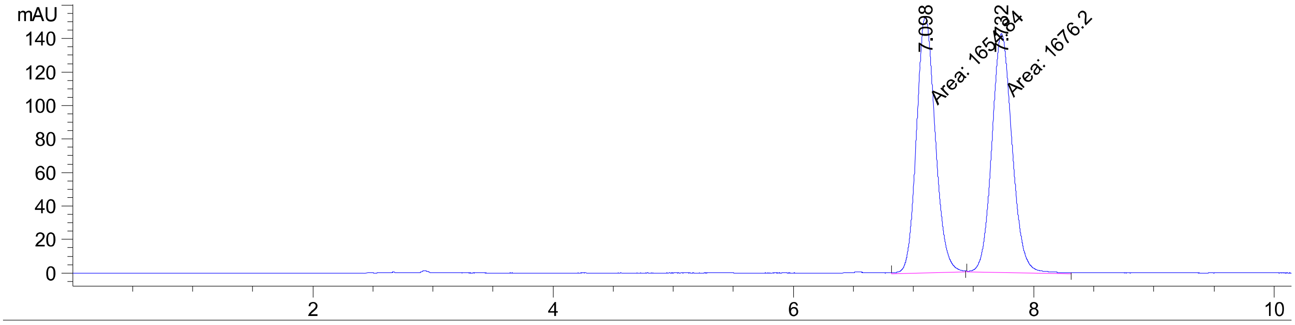

Peak #	RetTime [min]	Type	Width [min]	Area [mAU*s]	Height [mAU]	Area %
1	7.098	MM T	0.1805	1654.84167	152.78372	49.6795
2	7.732	MM T	0.1961	1676.19641	142.46574	50.3205

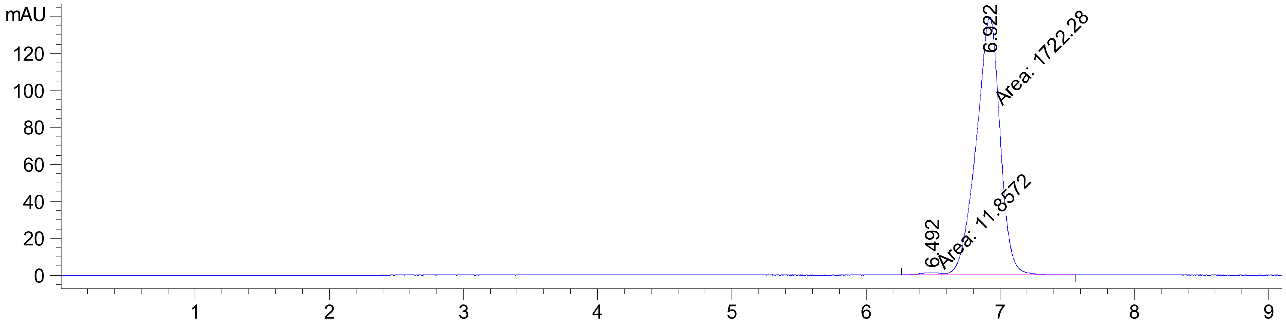

Peak #	RetTime [min]	Type	Width [min]	Area [mAU*s]	Height [mAU]	Area %
1	6.492	MM	0.1677	11.85719	1.17840	0.6838
2	6.922	MM	0.2055	1722.27979	139.65088	99.3162

Supplementary Figure 63. HPLC traces for compound **7e**

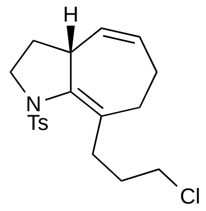

Chiralpak IB (1% IPA in hexane, flow rate = 1.3 mL/min, 254 nm) t_R major – 10.1 min, minor – 10.7 min

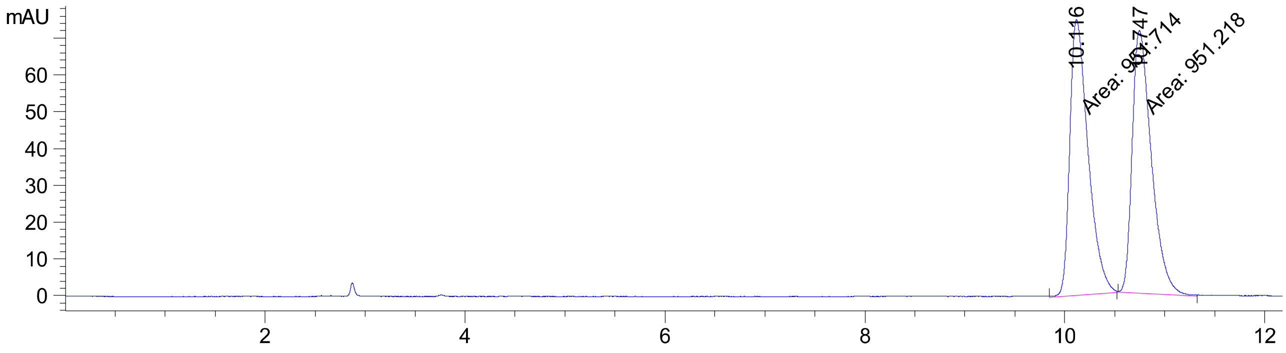

Peak #	RetTime [min]	Type	Width [min]	Area [mAU*s]	Height [mAU]	Area %
1	10.116	MM T	0.2523	951.71436	74.79766	50.0130
2	10.747	MM T	0.2224	951.21790	71.27395	49.9870

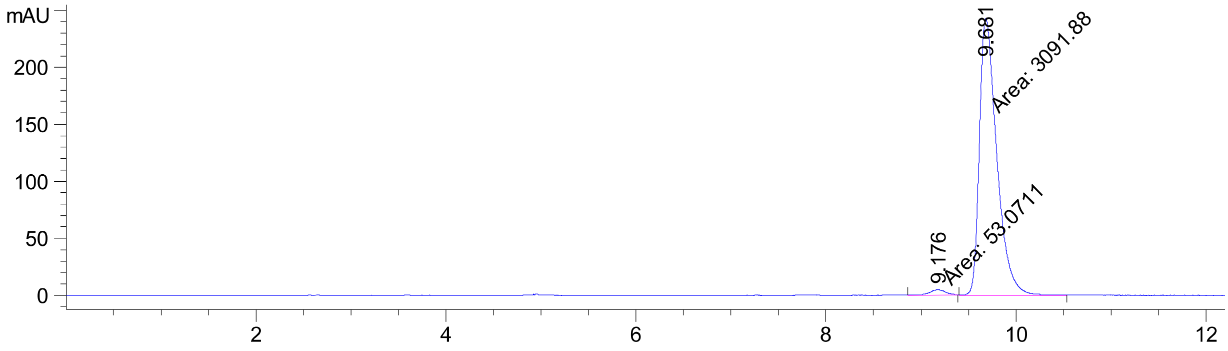

Peak #	RetTime [min]	Type	Width [min]	Area [mAU*s]	Height [mAU]	Area %
1	9.176	MM T	0.2320	53.07109	4.54429	1.6875
2	9.681	MM T	0.2525	3091.88428	243.05989	98.3125

Supplementary Figure 64. HPLC traces for compound **7f**

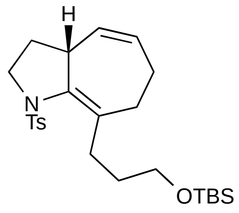

Chiralpak IB (1% IPA in hexane, flow rate = 1.3 mL/min, 254 nm) t_R major – 5.5 min, minor – 6.0 min

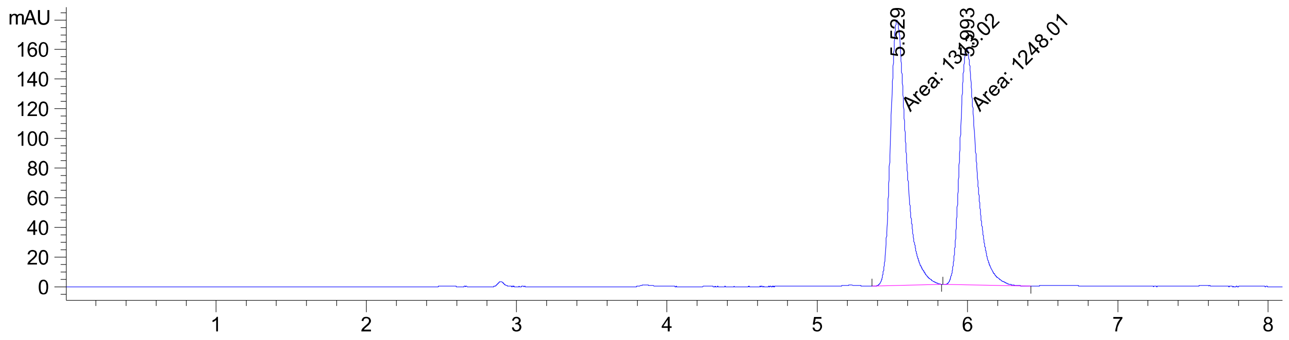

Peak #	RetTime [min]	Type	Width [min]	Area [mAU*s]	Height [mAU]	Area %
1	5.529	MM T	0.1220	1313.02344	179.34894	51.2693
2	5.993	MM T	0.1304	1248.00671	159.56924	48.7307

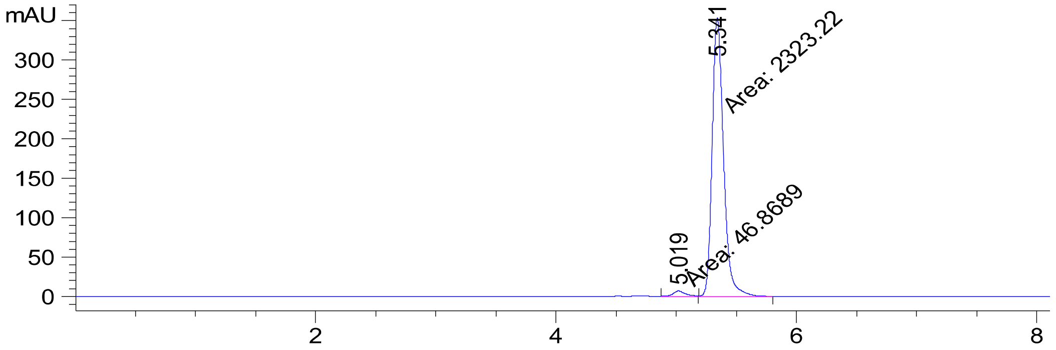

Peak #	RetTime [min]	Type	Width [min]	Area [mAU*s]	Height [mAU]	Area %
1	5.019	MM T	0.1166	46.86889	6.69742	1.9775
2	5.341	MM T	0.1094	2323.22437	353.77325	98.0225

Supplementary Figure 65. HPLC traces for compound **7g**

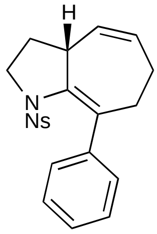

Chiralpak IB (10% IPA in hexane, flow rate = 1.3 mL/min, 254 nm) t_R major – 12.5 min, minor – 14.6 min

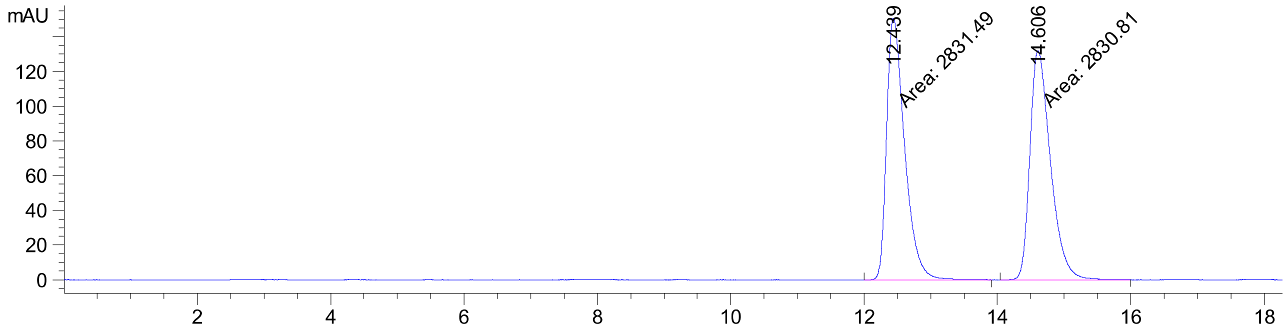

Peak #	RetTime [min]	Type	Width [min]	Area [mAU*s]	Height [mAU]	Area %
1	12.439	MM T	0.3795	2831.48706	150.72394	50.0060
2	14.606	MM T	0.4460	2830.80518	131.89793	49.9940

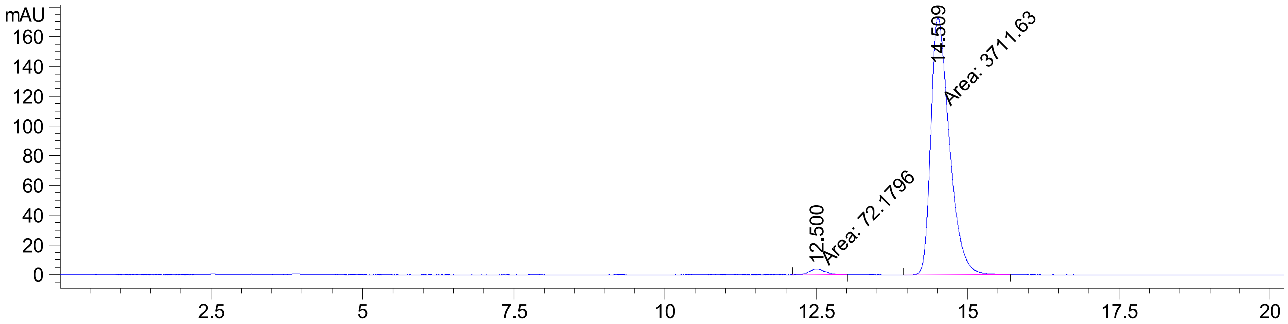

Peak #	RetTime [min]	Type	Width [min]	Area [mAU*s]	Height [mAU]	Area %
1	12.500	MM T	0.3090	72.17957	3.89345	1.9076
2	14.509	MM T	0.4512	3711.63354	173.29655	98.0924

Supplementary Figure 66. HPLC traces for compound **7k**

(a)

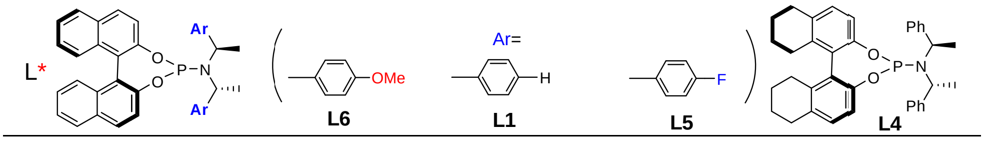

	MeO-Ph		Ph		F-Ph		Dearomatized	
	Re	*Si*	*Re*	*Si*	*Re*	*Si*	*Re*	*Si*
$r_{C3\text{-}Rh}$(Å)	2.67	2.82	2.69	2.79	2.70	2.82	2.68	2.73
$r_{Naph\pi-\pi}$(Å)	3.50	3.57	3.53	3.82	3.49	3.51	——	——
$r_{ph\pi-\pi}$(Å)	3.65	3.67	3.69	3.74	3.70	3.62	3.93	3.69
C_1-C_2-P-C_3(deg)	18.8	21.2	16.0	20.8	16.3	23.8	24.4	24.5

(b)

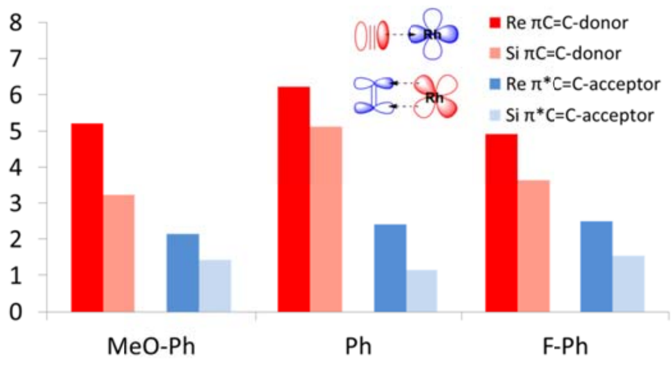

(c)

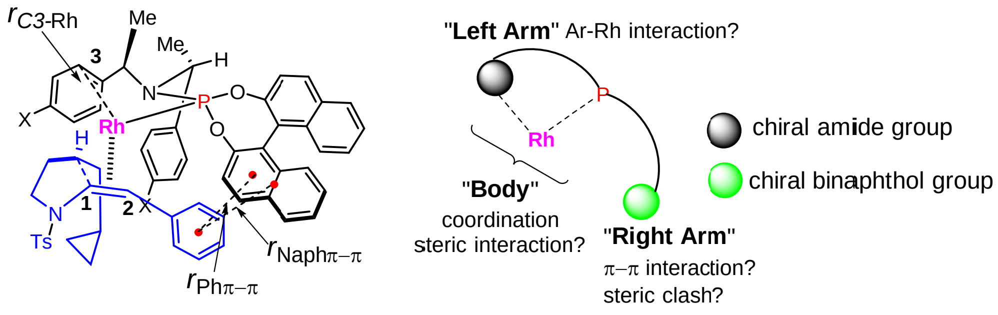

Supplementary Figure 67. Structure details and model of transition states (a) Important structure data in transition state **TS3** from Re and Si face by different ligands; (b) NBO analysis second-order perturbation theory for Rh-arene interaction; (c) Qualitative model for transition state with three functional regions.

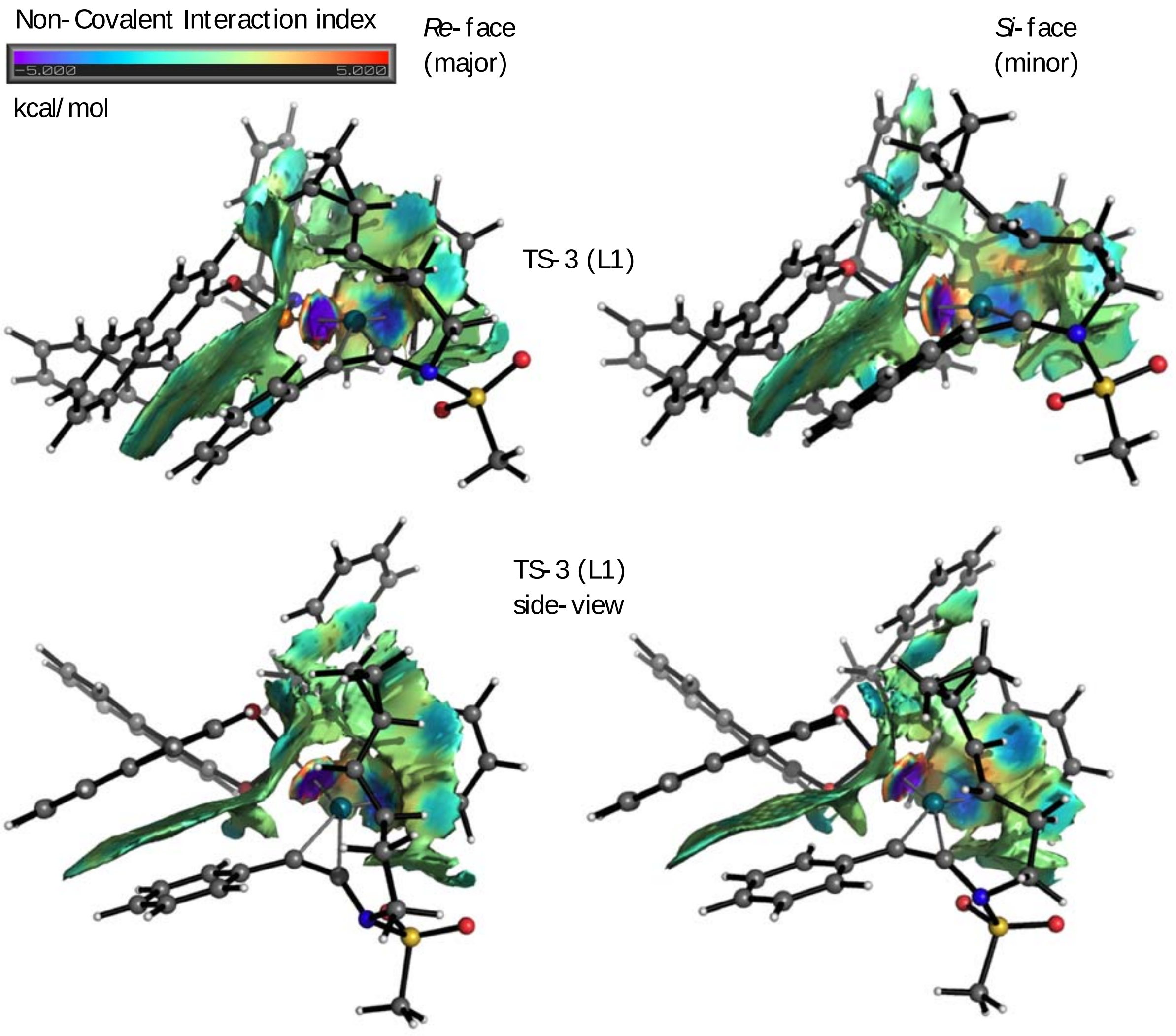

Supplementary Figure 68. Non-Covalent Interaction (NCI) index computed using from promolecular densities showing interactions between ligand and metal:ynamide complex, coloured according to magnitude.

	**TS3** in Metallacyclopentene Pathway		**TS2** in Vinylcyclopropane Pathway	
	Re-selectivity (R)	*Si*-selectivity (S)	*Re*-selectivity (R)	*Si*-selectivity (S)
***P*-*trans*-Ene-Down**	**18.7** 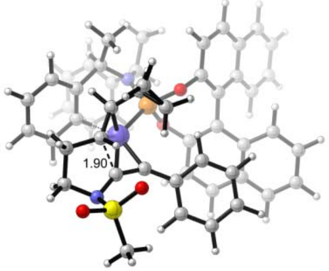	**31.3** 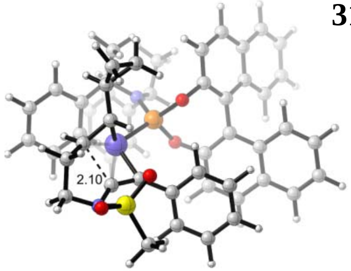	**23.0** 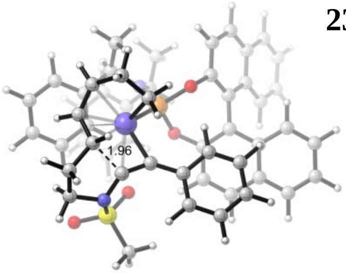	**22.0** 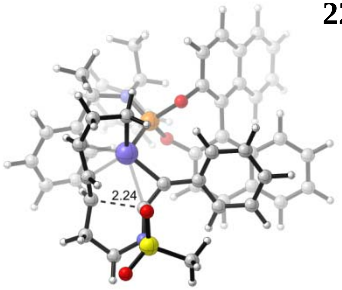
***P*-*trans*-Ene-Up**	**17.0** 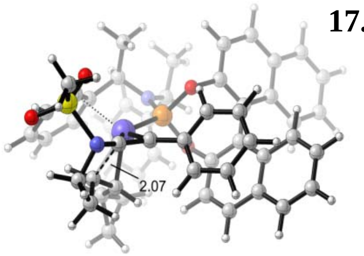	**19.7** 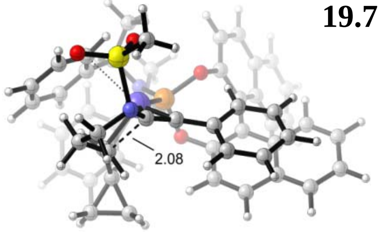	**21.9** 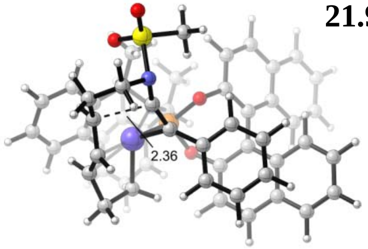	**24.8** 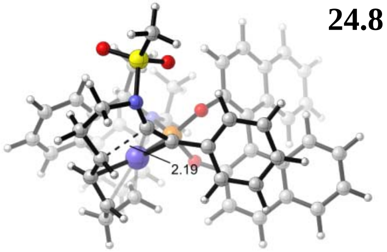
***P*-*trans*-Yne-Down**	**23.6** 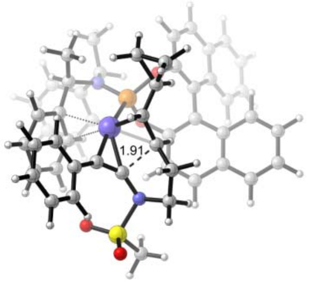	**23.9** 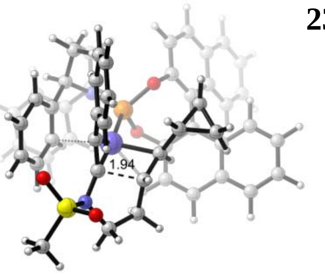	**29.3** 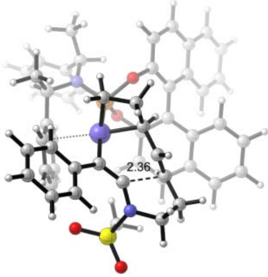	**24.5** 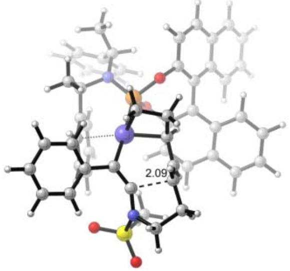

***P-trans*-Yne-Up**	**23.2** 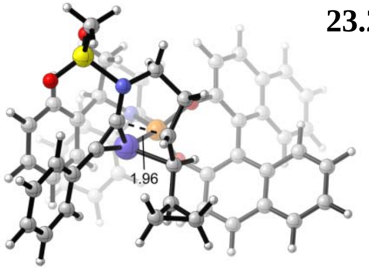	**30.7** 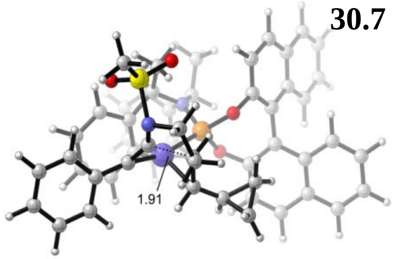	**35.3** 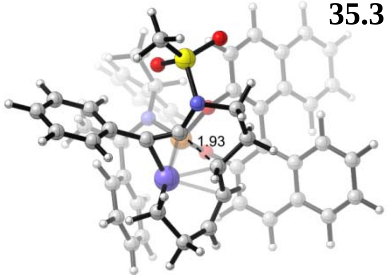	**41.7** 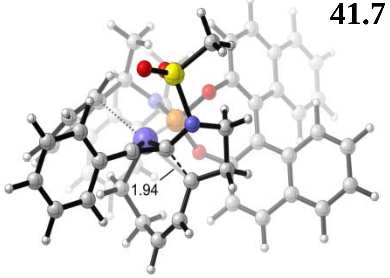

Supplementary Table 1. Structure of transition states TS2 and TS3 (energy shown in activation barrier with kcal mol^{-1} unit).

Re face	Metallacyclopentene pathway	Functional[b]	Vinylcyclopropane pathway	*Re* face
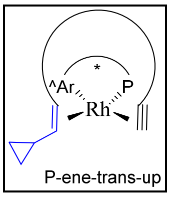	0	ω-B97XD	2.6	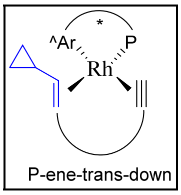
	0	ω-B97XD[c]	2.1	
	0	B3LYP	9.6	
	0	B3LYP-D3	1.3	
	0	M06	4.9	

[a]energy shown in kcal mol^{-1}, [b]Basis set: 6-311+G (d, p)/ Lanl2TZ, [c] Basis set: def2tzvpp

Supplementary Table 2. Relative energy[a] of key transition states **TS3** by different functional levels of calculations.

Supplementary Discussion

I Assignment of Relative Stereochemistry

Stereochemical assignment of compounds **7l-n**, **7o-q**, **7r**, **7s**; and **14o**, **14q**, and **14r**, and discussion of assignment of absolute configuration.

7l-n: Assignment of the relative stereochemistry of **7l** and **7m** is made on the basis of coupling constant analysis between H3 and H3a, which has a value of 10.5-11 Hz. Comparison with **7n** shows equivalent coupling constants between 2xH2 and H3 (11.0 and 7.0 Hz) (H3 and H3a are obscured), supporting an equivalent structural assigment. For the diastereomeric compound **14l**, these coupling constants are 7.5 and 6.0 Hz, indicating a different conformation and therefore configuration (see below).

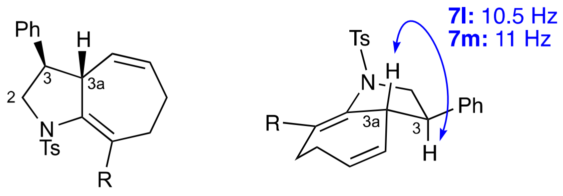

7l: R = Hex
7m: R = Ph

7l: R = Hex
7m: R = Ph

7o: Assignment of stereochemistry for **7o** is made on the basis of nOe enhancements, measured using ^{1}H–^{1}H NOESY experiments. For **7o**, a strong nOe enhancement was observed between the C2 methyl group and H3β, and between the Me group and H3a. A similar strong enhancement was seen between H3a and H3β, thus indicating all these groups to be on the same face of the bicycle. A complementary enhancement was observed between H2 (on the α-face), and H3α. A weak enhancement was observed between H2 and H3β.

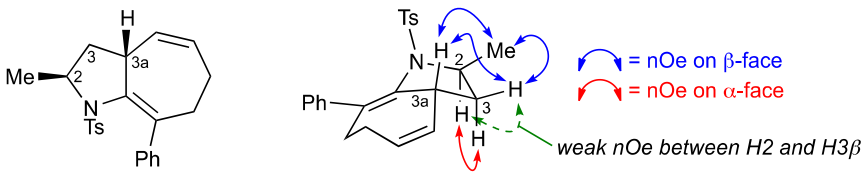

7o

14o: The assignment of stereochemistry in **14o** is clearly achieved through the observation of strong nOe enhancements between the C2 methyl group and H3β (β face), and between H2 and H3α, and H3α and H3a. Weak enhancements are observed between H2 and H3a, and between H3β and H2.

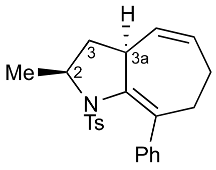

14o

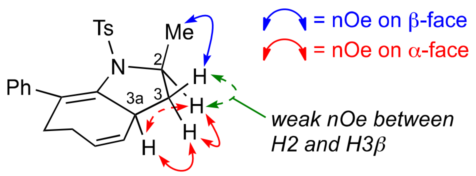

7q: H3a and one of the C2 sidechain protons (H2') are overlapping, rendering an unequivocal stereochemical assignment difficult. However, strong nOe enhancements are again seen between H2 and H3α, the latter of which does *not* show an enhancement with either of the H2' protons, or H3a, implying H2' and H3a to be on the opposite (β-) face. A strong enhancement is also observed between H3β and one of H2', as well as the overlapping H2'/H3a signals. This assignment is supported by equivalent nOe data for the C3a epimer, **14q** (see below).

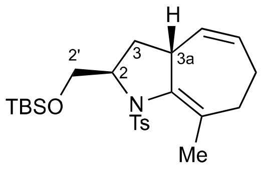

7q

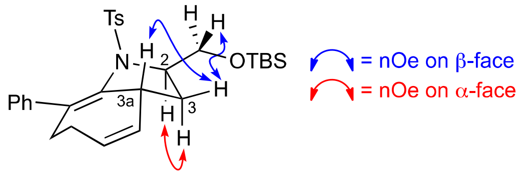

14q: The assignment of stereochemistry in **14q** is clearly achieved through the observation of strong nOe enhancements between the C2 sidechain protons H2', and H3β (β face), and between H2 and H3α, and H3α and H3a. A weak enhancement is observed between H3β and H2.

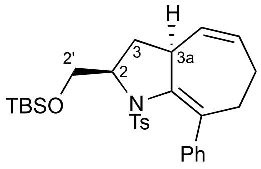

14q

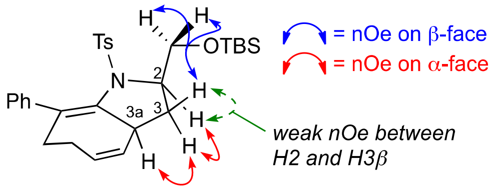

7p: The assignment of stereochemistry of **7p** is made by analogy with the data from **7o** and **7q** (this material being isolated as an inseparable mixture with its C3a epimer, **14p**).

7r: By analogy with compounds **7l-n**, a coupling constant of 12 Hz between H3a and H3 indicates an *anti* relationship, and therefore stereochemistry as depicted below:

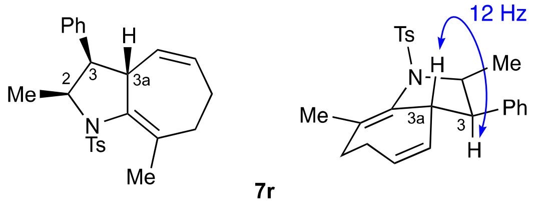

7r

For the diastereomeric compound **14l**, the H3a–H3 coupling constants is 7.5 Hz, indicating a different conformation and configuration.

7s: The regiochemistry of this product was assigned by HMBC correlations, which identified the position of the C6 sidechain (rather than this being at C7) by a clear mutual correlations between H9/C9 and C7/H7. Stereochemical assignment was achieved using a ^{1}H–^{1}H NOESY experiment. A strong enhancement between H3a and H7β, the latter of which also showed a weak enhancement with H6'. On the α-face, H7α showed an enhancement with one of H9, while the other H9 proton showed an enhancement with H6. These enhancements suggest a conformation as depicted below, where a strong puckering of the ring places the C6' sidechain in a *pseudo*-equatorial position, and distorts the positioning of the C9 sidechain to be closer to protons on the α-face of the ring system.

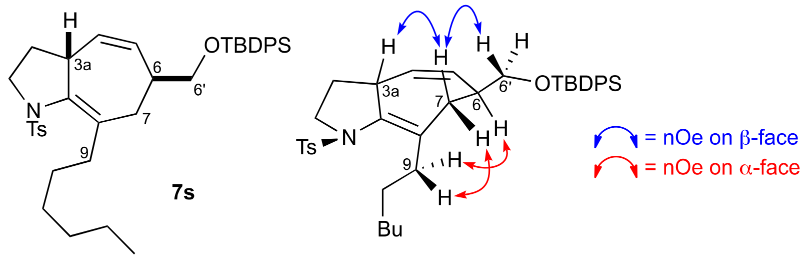

7s

II Assignment of absolute stereochemistry

As the C2 stereogenic centres in **7q** and **14q** are unambiguously defined from their synthesis (from (*R*)-glycidol), the above assignments therefore enable a definitive assignment of the stereochemistry at H3a in each compound, and therefore the sense of catalyst stereoinduction in both the catalyst / substrate matched and mismatched settings. We extend this assignment of (*R*)-configuration at the C3a stereogenic centre to the enantioselective reaction using (*S*,*R*,*R*)-ligands, assuming the same sense of catalyst stereocontrol operates in this enantioselective reaction as in the diastereoselective cases.

Supplementary Methods

I General Experimental Considerations

Nuclear Magnetic Resonance

^{1}H NMR spectra were obtained on a Bruker AVII500 (500 MHz) or AVIII400 (400 MHz) spectrometer and were referenced to residual non-deuterated solvent peaks in $CDCl_3$ (δ = 7.26) or C_6D_6 (δ = 7.16). Chemical shifts (δ_H and δ_C) are reported in parts per million (ppm) with signal splittings recorded as singlet (s), doublet (d), triplet (t), quartet (q), quintet (quin), sextet (sex), septet (sept), octet (oct), nonet (non) and multiplet (m). Coupling constants (*J* values) are measured to the nearest 0.5 Hz and are presented as observed. ^{13}C NMR spectra were obtained on a Bruker AVII500 with cryoprobe (126 MHz) or AVIII400 (101 MHz) spectrometer and were referenced to solvent peaks in $CDCl_3$ (δ = 77.16) and C6D6 (δ = 128.06).

Mass Spectrometry

Low-resolution mass spectra (*m/z*) were recorded on a Waters LCT Premier EX mass spectrometer, using electrospray ionization (ESI). High-resolution mass spectra (HRMS) were recorded by the Departmental Mass Spectrometry Service, University of Oxford on a Bruker MicroTOF (resolution = 5000 FWHM) using electrospray (ESI). The parent ion $[M]^+$, $[M+H]^+$ or $[M+Na]^+$ is calculated to 4 decimal places from the molecular formula, and all values are within a tolerance of 5 ppm.

Infrared Spectroscopy

Absorption spectra were obtained in $CHCl_3$ as solvent on a Bruker Tensor 27 FT-IR spectrometer. The sample was prepared as a thin film on a diamond/ZnSe PIKE Miracle ATR module. Wavelengths of maximum absorbance (ν_{max}) are quoted in wavenubers (cm^{-1}). Only selected, characteristic IR absorption data are provided for each compound.

Polarimetry

Optical rotations were recorded on a Perkin Elmer 241 or 341 polarimeter with a path length of 1 dm (using the sodium D line, 589 nm). $[\alpha]_D$ are reported in units of 10^{-1} deg cm^2 g^{-1}. Concentrations are reported in g/100 mL. Temperatures are reported in °C.

Elemental Analysis

Samples were analyzed by Mr. Stephen Boyer, Science Centre, London Metropolitan University.

Chromatography

Column chromatography refers to normal phase column chromatography and was performed on silica gel (35-70 mm) using head pressure by means of a nitrogen line. Thin-layer chromatography was performed on Merck Kieselgel 60 F_{254} plates with visualization by ultraviolet light (254 nm) and/or heating the plate after staining with vanillin. High performance liquid chromatography (HPLC) was performed on an Agilent 1200 Series running in normal phase under UV detection using a ZORBAX RX-SIL (150 mm x 4.6 mm ID) as the analytic column. Chiral analysis was carried out using a DAICEL CHIRALPAK-IA, IB or IC (250 mm x 4.6 mm ID).

Materials

Unless otherwise stated, all reactions were carried out in oven-dried glassware under an atmosphere of argon. Diethyl ether, dichloromethane, THF and toluene were dried over activated alumina beads before use. All other commercially available reagents and solvents, where appropriate, were dried and purified before use using standard procedures. Petroleum ether refers to the fraction of light petroleum ether boiling at 40-60 °C.

II General Experimental Procedures

General Procedure A: Synthesis of amines

According to the procedure of Alexakis et al.[1] To a solution of amine (1.0 equiv.) in Ti(O*i*-Pr)$_4$ (3.0 equiv.) was added ketone (1.0 equiv.) and the reaction mixture stirred for 1 h. Pd/C (10 wt%) was added and the resulting suspension was stirred under H_2 (1 atm, balloon) for 16 h. To the reaction mixture was added NaOH (3M, aq., 1.5 mL / mmol of amine) and EtOAc (3 mL / mmol of amine), and the solution was stirred for 1 h. The organic layer was separated and a further two rounds of this extraction process were carried out on the resulting (and subsequent) aqueous phase. The combined organic extracts were dried (Na_2SO_4), filtered through a pad of celite, and concentrated *in vacuo*. The residue was taken up in EtOAc (5 mL) and concentrated HCl (1 mL), and the resulting solution was azeotroped with EtOAc until a white solid (the amine hydrochloride salt) was obtained.

General Procedure B: Synthesis of phosphoramidites

According to the procedure of Fletcher et al.[2] To a solution of PCl_3 (1.0 equiv.) in CH_2Cl_2 (10 mL / mmol of amine) at 0 °C was added triethylamine (6.0 equiv.) dropwise. The solution was allowed to reach rt, then the amine (1.0 equiv.) was added and the reaction mixture stirred for 5 h. To the stirred solution was added BINOL (1.0 equiv.) and the subsequent mixture stirred for a further 16 h. The reaction mixture was filtered through a pad of silica (5 mm) and celite (5 mm), washed with CH_2Cl_2 and the solvent removed *in vacuo*.

General Procedure C: Copper(II)-catalyzed ynamide formation

According to the procedure of Hsung et al.[3] To a mixture of sulfonamide (1.0 equiv.), K_3PO_4 (2.0 equiv.), $CuSO_4$•$5H_2O$ (0.4 equiv.) and 1,10-phenanthroline (0.8 equiv.) was added a solution of bromoalkyne (1.5 equiv.) in toluene (3 mL / mmol of sulfonamide). The reaction mixture was stirred at 70 °C for the stated time and then cooled to rt. The mixture was filtered through celite, eluting with Et_2O, and the filtrate was concentrated *in vacuo*.

General Procedure D: Cs_2CO_3 promoted synthesis of dichloroenamides

According to the procedure of Anderson et al.[4] To a suspension of amide (1.0 equiv.) and Cs_2CO_3 (3.0 equiv.) in DMF (0.75 mL / mmol amide) at 50 °C was added trichloroethylene (3.0 equiv.) dropwise over ten min. The resulting mixture was stirred at 50 °C for the stated time. Upon cooling to rt, the mixture was partitioned between EtOAc and water, the organic layer separated and further washed with water (x 3). The organic layer was dried (Na_2SO_4), filtered and concentrated *in vacuo*.

General Procedure E: Synthesis of ynamides using phenyllithium

According to the procedure of Anderson et al.[4] To a solution of 1,2-dichloroenamide (1.0 equiv.) in THF (10 mL / mmol of enamide) at –78 °C was added phenyllithium (2.0 M in dibutyl ether, 2.2 equiv.) dropwise over 10 minutes. The reaction mixture was stirred at –78 °C for 1 h, after which time the electrophile (1.2 equiv.) was added. The solution was allowed to warm to rt and stirred for 1 h. The reaction mixture was quenched with water and the aqueous layer extracted with Et_2O (x 2). The combined organic extracts were dried ($MgSO_4$), filtered and concentrated *in vacuo*.

General Procedure F: Racemic [5+2] cycloisomerization

To an oven-dried vial containing the ynamide vinylcyclopropane (1.0 equiv.) under Ar was added a solution of $[(C_{10}H_8)Rh(cod)]SbF_6$ (5 mol%) in degassed CH_2Cl_2 (10 mL / mmol of ynamide). The reaction mixture was stirred at room temperature under Ar until consumption of the ynamide was observed by TLC (see Figure 3 for reaction times). The reaction mixture was then concentrated, and the resulting crude product was purified by flash chromatography (SiO_2, petroleum ether / ethyl acetate eluent).

General Procedure G: Asymmetric [5+2] cycloisomerization

A solution of $[RhCl(C_2H_4)_2]_2$ (2.5 mol%), $NaBAr^F_4$ (6 mol%) and phosphoramidite ligand (6 mol%) in degassed CH_2Cl_2 (10 mL / mmol of ynamide) was stirred for 20 min under Ar. The solution was filtered (through a PTFE filter-tipped syringe) into an oven-dried vial containing ynamide vinylcyclopropane (1.0 equiv.) under Ar. The reaction mixture was stirred at room temperature under Ar until consumption of the ynamide was observed by TLC (see Figure 6 for reaction times). The reaction mixture was then concentrated, and the resulting material was purified by flash chromatography (SiO_2, petroleum ether / ethyl acetate eluent).

III Characterization of Compounds

Phosphoramidite ligands

(*R*)-bis((*R*)-1-(4-fluorophenyl)ethyl)ammonium chloride, A5

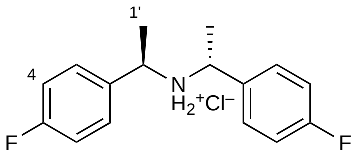

Prepared by General Procedure A using (*R*)-1-(4-fluorophenyl)ethan-1-amine (0.97 mL, 7.19 mmol, 1.0 equiv.) and 1-(4-fluorophenyl)ethan-1-one (0.87 mL, 7.19 mmol, 1.0 equiv.). The crude material was recrystallised from hot EtOAc and methanol to give **A5** as colourless crystals (604 mg, 2.29 mmol, 32%); **m.p.** 185–187 °C; $[\alpha]_D^{25}$ –16.70 (*c* = 1.0, $CHCl_3$); **R_f** 0.31 (petroleum ether / EtOAc (8:2)); **IR** (thin film, ν_{max} / cm^{-1}) 3044, 2755, 2494, 1584, 1514, 1229, 1165; **^{1}H NMR** (400 MHz, $CDCl_3$) δ_H 10.51 (2H, s, NH_2), 7.53 (4H, dd, J_{HH} = 8.5 Hz and J_{HF} = 5.0 Hz, H3), 7.13 (4H, app. t, *J* = 8.5 Hz, H4), 3.88-3.80 (2H, m, H1), 1.89 (6H, s, H1'); **^{13}C NMR** (100 MHz, $CDCl_3$) δ_C 163.2 (d, J_{CF} = 250.5 Hz), 132.1 (d, J_{CF} = 3.5 Hz), 130.2 (d, J_{CF} = 8.5 Hz), 116.5 (d, J_{CF} = 22.0 Hz), 56.5, 21.7; **^{19}F NMR** (377 MHz, $CDCl_3$) δ_F –111.7; **HRMS** (ESI+) calc. for $C_{16}H_{18}NF_2$ $[M+H]^+$ 262.1402, found 262.1401.

(*R*)-bis((*R*)-1-(4-methoxyphenyl)ethyl)ammonium chloride, A6

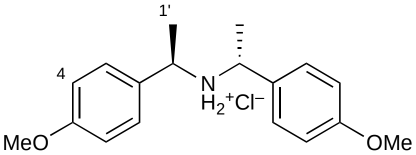

Prepared by General Procedure A using (*R*)-1-(4-methoxyphenyl)ethan-1-amine (0.98 mL, 6.61 mmol, 1.0 equiv.) and 1-(4-methoxyphenyl)ethan-1-one (0.99 g, 6.61 mmol, 1.0 equiv.). The crude material was recrystallised from hot EtOAc and methanol to give **A6** as colourless crystals (395 mg, 1.38 mmol, 21%); **m.p.** 209–211 °C; $[\alpha]_D^{25}$ –119.50 (c = 1.0, $CHCl_3$); **R_f** 0.22 (petroleum ether / EtOAc (2:1)); **IR** (thin film, ν_{max} / cm^{-1}) 2954, 2704, 2493, 1612, 1581, 1516, 1250, 1182; **^{1}H NMR** (400 MHz, $CDCl_3$) δ_H 10.28 (2H, s, NH_2), 7.48 (4H, d, *J* = 8.5 Hz, H3), 6.94 (4H, d, *J* = 8.5 Hz, H4), 3.85-378 (2H, m, H1), 3.83 (6H, s, OCH_3), 1.87 (6H, d, *J* = 7.0 Hz, H1'); **^{13}C NMR** (100 MHz, $CDCl_3$) δ_C 160.1, 129.8, 128.5, 114.6, 56.6, 55.5, 21.7; **HRMS** (ESI+) calc. for $C_{18}H_{24}O_2N$ $[M+H]^+$ 286.1802, found 286.1802.

(11b*S*)-*N*,*N*-bis((*R*)-1-(4-fluorophenyl)ethyl)dinaphtho[2,1-*d*:1',2'-*f*][1,3,2]dioxaphosphepin-4-amine, L5

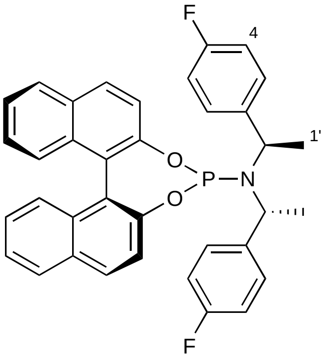

Prepared by General Procedure B using amine **A5** (243 mg, 0.87 mmol, 1.0 equiv.). The crude material was purified by column chromatography (petroleum ether / CH_2Cl_2 (8:2)) to give **L5** as a colourless crystalline solid (380 mg, 0.66 mmol, 76%); $[\alpha]_D^{25}$ –468.00 (*c* = 1.0, $CHCl_3$); **R*f*** 0.19 (petroleum ether / CH_2Cl_2 (8:2)); **IR** (thin film, ν_{max} / cm^{-1}) 3060, 2973, 1603, 1509, 1230; **^{1}H NMR** (500 MHz, $CDCl_3$) δ_H 7.99-7.90 (4H, m, BINOL*H*), 7.58 (1H, d, *J* = 9.0 Hz, BINOL*H*), 7.45 (1H, d, *J* = 9.0 Hz, BINOL*H*), 7.42-7.39 (3H, m, BINOL*H*), 7.30-7.22 (3H, m, BINOL*H*), 7.06-7.03 (4H, m, H3), 6.83 (4H, dd, J_{HH} = 8.5 Hz and J_{HF} = 8.5 Hz, H4), 4.48-4.42 (2H, m, H1), 1.71 (6H, d, *J* = 7.0 Hz, H1'); **^{13}C NMR** (125 MHz, $CDCl_3$) δ_C 161.6 (2C, d, J_{CF} = 245.5 Hz), 149.9 (d, J_{CP} = 7.5 Hz), 149.4, 138.7 (2C), 132.8 (2C), 131.5, 130.5, 130.5, 129.6, 129.5 (4C, d, J_{CF} = 8.0 Hz), 128.4, 128.2, 127.2, 127.1, 126.1, 126.1, 124.9, 124.6, 124.1 (d, J_{CP} = 5.5 Hz), 122.3, 122.2, 121.8 (d, J_{CP} = 2.5 Hz), 114.5 (4C, d, J_{CF} = 21.0 Hz), 51.7 (2C, d, J_{CP} = 12.0 Hz), 22.2 (2C); **^{19}F NMR** (377 MHz, $CDCl_3$) δ_F –116.1; **^{31}P NMR** (202 MHz, $CDCl_3$) δ_P 145.5; **HRMS** (ESI+) calc. for $C_{36}H_{29}O_2NF_2P$ $[M+H]^+$ 576.1899, found 576.1897.

11b*S*)-*N*,*N*-bis((*R*)-1-(4-methoxyphenyl)ethyl)dinaphtho[2,1-*d*:1',2'-*f*][1,3,2]dioxaphosphepin-4-amine, L6

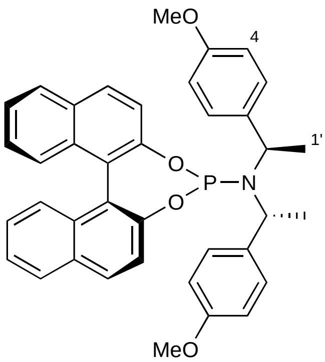

Prepared by General Procedure B using amine **A6** (268 mg, 0.83 mmol, 1.0 equiv.). The crude material was purified by column chromatography (petroleum ether / CH_2Cl_2 (3:1)) to give **L6** as a colourless crystalline solid (316 mg, 0.53 mmol, 63%); $[\alpha]_D^{25}$ –492.40 (*c* = 1.0, $CHCl_3$); **R*f*** 0.36

(petroleum ether / CH_2Cl_2 (1:1)); **IR** (thin film, ν_{max} / cm^{-1}) 3060, 2969, 2835, 1611, 1590, 1511, 1249, 1232; **^{1}H NMR** (500 MHz, $CDCl_3$) δ_H 7.94 (2H, d, *J* = 9.0 Hz, BINOL*H*), 7.91-7.88 (2H, m, BINOL*H*), 7.58 (1H, d, *J* = 9.0 Hz, BINOL*H*), 7.42 (1H, d, *J* = 8.5 Hz, BINOL*H*), 7.40-7.37 (3H, m, BINOL*H*), 7.28 (1H, d, *J* = 8.5 Hz, BINOL*H*), 7.26-7.21 (2H, m, BINOL*H*), 7.04-7.02 (4H, m, H3), 6.70 (4H, d, *J* = 9.0 Hz, H4), 4.48-4.41 (2H, m, H1), 3.76 (6H, s, OCH_3), 1.70 (6H, d, *J* = 6.5 Hz, H1'); **^{13}C NMR** (125 MHz, $CDCl_3$) δ_C 158.3 (2C), 150.2 (d, J_{CP} = 7.0 Hz), 149.6, 135.2 (2C), 132.8 (2C), 131.4, 130.5, 130.2, 129.4, 129.1 (4C), 128.3, 128.2, 127.2, 127.1, 126.0, 125.9, 124.7, 124.4, 124.0 (d, J_{CP} = 5.5 Hz), 122.5, 122.5, 121.8 (d, J_{CP} = 2.5 Hz), 113.1 (4C), 55.3 (2C), 51.5 (2C, d, J_{CP} = 12.5 Hz), 22.3 (2C); **^{31}P NMR** (202 MHz, $CDCl_3$) δ_P 147.4; **HRMS** (ESI+) calc. for $C_{38}H_{35}O_4NP$ $[M+H]^+$ 600.2298, found 600.2298.

Ynamide vinylcyclopropanes

(*E*)-*N*-(4-cyclopropylbut-3-en-1-yl)-4-methyl-*N*-(phenylethynyl)benzenesulfonamide, 1a

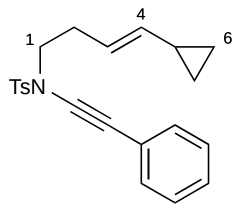

Prepared by General Procedure C using sulfonamide **6a** (200 mg, 0.75 mmol, 1.0 equiv.) and (bromoethynyl)benzene (205 mg, 1.13 mmol, 1.5 equiv.). The resulting crude material was purified by column chromatography (petroleum ether / EtOAc (95:5)) to give **1a** as a colourless oil (250 mg, 0.68 mmol, 91%); **R_f** 0.40 (petroleum ether / EtOAc (9:1)); **IR** (thin film, ν_{max} / cm^{-1}) 3004, 2925, 2234, 1666, 1364, 1167, 1091; **^{1}H NMR** (500 MHz, $CDCl_3$) δ_H 7.85 (2H, d, *J* = 8.0 Hz, Ts*H*), 7.39-7.28 (7H, m, Ts*H* and Ph*H*), 5.40 (1H, dt, *J* = 15.0 and 7.0 Hz, H3), 5.05 (1H, dd, *J* = 15.0 and 8.5 Hz, H4), 3.43 (2H, t, *J* = 7.5 Hz, H1), 2.46 (3H, s, TsCH_3), 2.38 (2H, app. q, *J* = 7.5 Hz, H2), 1.35-1.28 (1H, m, H5), 0.68-0.64 (2H, m, H6), 0.33-0.30 (2H, m, H6); **^{13}C NMR** (125 MHz, $CDCl_3$) δ_C 144.8, 137.7, 135.0, 131.6, 130.0, 128.5, 128.0, 128.0, 123.2, 122.8, 82.6, 71.2, 51.8, 31.5, 21.9, 13.9, 6.8; **HRMS** (ESI+) calc. for $C_{22}H_{23}NNaO_2S$ $[M+Na]^+$ 388.1342, found 388.1327.

(*E*)-*N*-(4-cyclopropylbut-3-en-1-yl)-*N*-((4-fluorophenyl)ethynyl)-4-methylbenzenesulfonamide, 1b

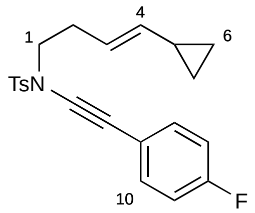

Prepared by General Procedure C using sulfonamide **6a** (175 mg, 0.66 mmol, 1.0 equiv.) and 1-(bromoethynyl)-4-fluorobenzene (263 mg, 1.32 mmol, 2.0 equiv.). The resulting crude material was purified by flash column chromatography (petroleum ether / Et_2O (9:1)) to give **1b** as a yellow oil (180 mg, 0.47 mmol, 71%): $\mathbf{R_f}$ 0.24 (petroleum ether / Et_2O (9:1)); **IR** (thin film, ν_{max}/ cm^{-1}) 3075, 3004, 2924, 2273,1599, 1510, 1354, 1225, 1161, 1089; ^{1}**H NMR** (400 MHz, $CDCl_3$) δ_H 7.84-7.81 (2H, m, Ts*H*), 7.36-7.31 (4H, m, Ts*H* and H10), 7.01-6.95 (2H, m, H11), 5.38 (1H, dt, *J* = 15.5 and 7.0 Hz, H3), 5.02 (1H, ddt, *J* = 15.5, 8.5 and 1.0 Hz, H4), 3.40 (2H, t, *J* = 7.5 Hz, H1), 2.45 (3H, s, TsCH_3), 2.36 (2H, app. q, *J* = 7.0, H2), 1.33-1.25 (1H, m, H5), 0.67-0.61 (2H, m, H6), 0.31-0.28 (2H, m, H6); ^{13}**C NMR** (100 MHz, $CDCl_3$) δ_C 162.3 (d, *J* = 249.0 Hz), 144.7, 137.6, 134.8, 133.6 (d, *J* = 234.0 Hz), 129.9, 127.8, 122.6, 119.0 (d, *J* = 4.0 Hz), 115.6 (d, *J* = 22.0 Hz), 82.0, 69.9, 51.6, 31.3, 21.8, 13.8, 6.7; ^{19}**F NMR** (376 MHz, $CDCl_3$) δ_F −115.6; **HRMS** (ESI+) calc. for $C_{22}H_{22}FNNaO_2S$ $[M+Na]^+$ 406.1253, found 406.1256.

(*E*)-*N*-((4-chlorophenyl)ethynyl)-*N*-(4-cyclopropylbut-3-en-1-yl)-4-methylbenzenesulfonamide, 1c

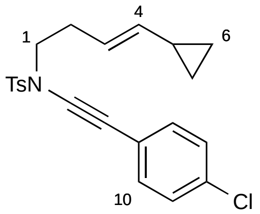

Prepared by General Procedure C using sulfonamide **6a** (130 mg, 0.49 mmol, 1.0 equiv.) and 1-(bromoethynyl)-4-chlorobenzene (210 mg, 0.98 mmol, 2.0 equiv.). The resulting crude material was purified by flash column chromatography (petroleum ether / Et_2O (9:1)) to give **1c** as a yellow oil (153 mg, 0.38 mmol, 79%): $\mathbf{R_f}$ 0.28 (petroleum ether / Et_2O (9:1)); **IR** (thin film, ν_{max}/ cm^{-1}) 3004, 2928, 2236, 1596, 1366, 1168, 1090, 1014; ^{1}**H NMR** (400 MHz, $CDCl_3$) δ_H 7.82 (2H, d, *J* = 8.5 Hz, Ts*H*), 7.35 (2H, d, *J* = 8.5 Hz, Ts*H*), 7.29-7.24 (4H, m, H10 and H11), 5.38 (1H, dt, *J* = 15.0 and 7.0 Hz, H3), 5.03 (1H, dd, *J* = 15.0 and 8.5 Hz, H4), 3.41 (2H, t, *J* = 7.5 Hz, H1), 2.45 (3H, s, TsCH_3), 2.36 (2H, td, *J* = 7.5 and 7.0 Hz, H2), 1.34-1.26 (1H, m, H5), 0.67-0.63 (2H, m, H6), 0.32-0.28 (2H, m, H6); ^{13}**C NMR** (100 MHz, $CDCl_3$) δ_C 144.7, 137.5, 134.7, 133.7, 132.5, 129.8, 128.6,

127.7, 122.4, 121.5, 83.3, 70.0, 51.5, 31.2, 21.7, 13.6, 6.5; **HRMS** (ESI+) calc. for $C_{22}H_{22}ClNNaO_2S$ $[M+Na]^+$ 422.0957, found 442.0960.

(*E*)-*N*-(4-cyclopropylbut-3-en-1-yl)-*N*-((4-methoxyphenyl)ethynyl)-4-methylbenzenesulfonamide, 1d

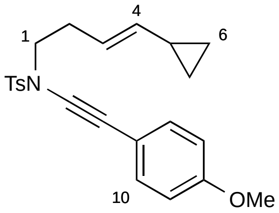

Prepared by General Procedure C using sulfonamide **6a** (175 mg, 0.66 mmol, 1.0 equiv.) and 1-(bromoethynyl)-4-methoxybenzene (278 mg, 1.32 mmol, 2.0 equiv.). The resulting crude material was purified by flash column chromatography (petroleum ether / Et_2O (9:1)) to give **1d** as a yellow oil (170 mg, 0.43 mmol, 65%): **R*f*** 0.14 (petroleum ether / Et_2O (9:1)); **IR** (thin film, ν_{max}/ cm^{-1}) 3003, 2934, 2237, 1605, 1568, 1512, 1457, 1363, 1288, 1248, 1167, 1091, 1030; **^{1}H NMR** (500 MHz, $CDCl_3$) δ_H 7.83 (2H, d, *J* = 8.0 Hz, Ts*H*), 7.35 (2H, d, *J* = 8.0 Hz, Ts*H*), 7.31 (2H, d, *J* = 8.5 Hz, H10), 6.82 (2H, d, *J* = 8.5 Hz, H11), 5.39 (1H, dt, *J* = 15.5 and 7.0 Hz, H3), 5.03 (1H, dd, *J* = 15.5 and 8.5 Hz, H4), 3.81 (3H, s, OC*H*$_3$), 3.40 (2H, t, *J* = 7.5 Hz, H1), 2.45 (3H, s, TsC*H*$_3$), 2.36 (2H, td, *J* = 7.5 and 7.0 Hz, H2), 1.33-1.27 (1H, m, H5), 0.67-0.63 (2H, m, H6), 0.31-0.28 (2H, m, H6); **^{13}C NMR** (100 MHz, $CDCl_3$) δ_C 159.9, 144.8, 137.8, 135.2, 133.8, 130.1, 128.1, 123.1, 115.3, 114.3, 81.2, 70.9, 55.7, 52.0, 31.6, 22.1, 14.0, 6.9; **HRMS** (ESI+) calc. for $C_{23}H_{25}NNaO_3S$ $[M+Na]^+$ 418.1453, found 418.1458.

(*E*)-*N*-(4-cyclopropylbut-3-en-1-yl)-4-methyl-*N*-(oct-1-yn-1-yl)benzenesulfonamide, 1e

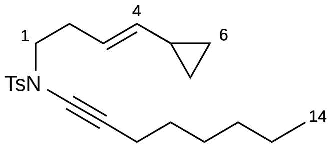

Prepared by General Procedure C using sulfonamide **6a** (100 mg, 0.38 mmol, 1.0 equiv.) and 1-bromooct-1-yne (107 mg, 0.57 mmol, 1.5 equiv.). The crude material was purified by column chromatography (petroleum ether / EtOAc (95:5)) to give **1e** as a colourless oil (87 mg, 0.23 mmol, 62%); **R*f*** 0.55 (petroleum ether / EtOAc (9:1)); **IR** (thin film, ν_{max} / cm^{-1}) 2929, 2858, 2252, 1456, 1364, 1167, 1092; **^{1}H NMR** (500 MHz, $CDCl_3$) δ_H 7.77 (2H, d, *J* = 8.0 Hz, Ts*H*), 7.32 (2H, d, *J* = 8.0 Hz, Ts*H*), 5.36 (1H, dt, *J* = 15.5 and 7.0 Hz, H3), 5.00 (1H, dd, *J* = 15.5 and 8.0 Hz, H4), 3.27 (2H, t, *J* = 7.5 Hz, H1), 2.44 (3H, s, TsC*H*$_3$), 2.30-2.24 (4H, m, H2 and 9), 1.49-1.44 (2H, m, H10), 1.36-1.23 (7H, m, H5, 11, 12 and 13), 0.88 (3H, t, *J* = 7.0 Hz, H14), 0.67-0.63 (2H, m, H6), 0.31-

0.28 (2H, m, H6); **^{13}C NMR** (125 MHz, $CDCl_3$) δ_C 144.1, 137.1, 134.7, 129.5, 127.6, 122.7, 72.9, 70.4, 51.3, 31.3, 31.0, 28.9, 28.5, 22.6, 21.6, 18.5, 14.1, 13.6, 6.4; **HRMS** (ESI+) calc. for $C_{22}H_{31}NNaO_2S$ $[M+Na]^+$ 396.1968, found 396.1958.

(*E*)-*N*-(5-chloropent-1-yn-1-yl)-*N*-(4-cyclopropylbut-3-en-1-yl)-4-methylbenzenesulfonamide, 1f

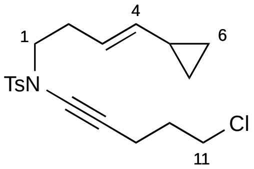

Prepared by General Procedure C using sulfonamide **6a** (100 mg, 0.38 mmol, 1.0 equiv.) and 1-bromo-5-chloropent-1-yne (103 mg, 0.57 mmol, 1.5 equiv.). The crude material was purified by column chromatography (petroleum ether / EtOAc (95:5)) to give **1f** as a colourless oil (112 mg, 0.31 mmol, 81%); **R_f** 0.40 (petroleum ether / EtOAc (9:1)); **IR** (thin film, ν_{max} / cm^{-1}) 3081, 3003, 2926, 2253, 1597, 1363, 1166, 1120; **^{1}H NMR** (500 MHz, $CDCl_3$) δ_H 7.77 (2H, d, *J* = 8.5 Hz, Ts*H*), 7.34 (2H, d, *J* = 8.5 Hz, Ts*H*), 5.35 (1H, dt, *J* = 15.5 and 7.5 Hz, H3), 5.00 (1H, dd, *J* = 15.5 and 8.5 Hz, H4), 3.60 (2H, t, *J* = 6.5 Hz, H11), 3.28 (2H, t, *J* = 7.5 Hz, H1), 2.46 (2 H, t, *J* = 6.5 Hz, H9), 2.44 (3H, s, $TsCH_3$), 2.28 (2H, q, *J* = 7.5 Hz, H2), 1.92 (2H, quin, *J* = 6.5 Hz, H10), 1.33-1.26 (1H, m, H5), 0.67-0.63 (2H, m, H6), 0.31-0.28 (2H, m, H6); **^{13}C NMR** (125 MHz, $CDCl_3$) δ_C 144.4, 137.3, 134.6, 129.6, 127.6, 122.6, 74.1, 68.4, 51.3, 43.6, 31.5, 31.0, 21.6, 15.9, 13.6, 6.5; **HRMS** (ESI+) calc. for $C_{19}H_{24}ClNNaO_2S$ $[M+Na]^+$ 388.1108, found 388.1093.

(*E*)-*N*-(5-((*tert*-butyldimethylsilyl)oxy)pent-1-yn-1-yl)-*N*-(4-cyclopropylbut-3-en-1-yl)-4-methylbenzenesulfonamide, 1g

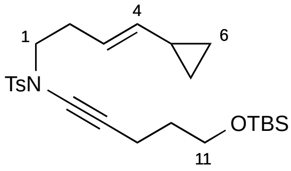

Prepared by General Procedure C using sulfonamide **6a** (100 mg, 0.38 mmol, 1.0 equiv.) and ((5-bromopent-4-yn-1-yl)oxy)(*tert*-butyl)dimethylsilane (157 mg, 0.57 mmol, 1.5 equiv.). The crude material was purified by column chromatography (petroleum ether / EtOAc (95:5)) to give **1g** as a colourless oil (96 mg, 0.21 mmol, 55%); **R_f** 0.58 (petroleum ether / EtOAc (9:1)); **IR** (thin film, ν_{max} / cm^{-1}) 2953, 2923, 2857, 2254, 1363, 1254, 1168, 1097; **^{1}H NMR** (500 MHz, $CDCl_3$) δ_H 7.76 (2H, d, *J* = 8.0 Hz, Ts*H*), 7.32 (2H, d, *J* = 8.0 Hz, Ts*H*), 5.35 (1H, dt, *J* = 15.5 and 7.5 Hz, H3), 5.00 (1H, dd, *J* = 15.5 and 8.5 Hz, H4), 3.64 (2H, t, *J* = 6.5 Hz, H11), 3.26 (2H, t, *J* = 7.5 Hz, H1), 2.44 (3H, s, $TsCH_3$), 2.34 (2H, t, *J* = 6.5 Hz, H9), 2.28 (2H, q, *J* = 7.5 Hz, H2), 1.68 (2H, quin, *J* = 6.5

Hz, H10), 1.33-1.26 (1H, m, H5), 0.88 (9H, s, SiC(C*H*3)3), 0.67-0.63 (2H, m, H6), 0.32-0.28 (2H, m, H6), 0.03 (6H, s, Si(C*H*3)2); **^{13}C NMR** (125 MHz, $CDCl_3$) δ_C 144.1, 137.1, 134.8, 129.5, 127.6, 122.7, 73.0, 70.0, 61.6, 51.4, 32.1, 31.0, 25.9, 21.7, 18.4, 14.9, 13.6, 6.5, –5.4; **HRMS** (ESI+) calc. for $C_{25}H_{39}NNaO_3SSi$ $[M+Na]^+$ 484.2312, found 484.2299.

(*E*)-*N*-(4-cyclopropylbut-3-en-1-yl)-4-methyl-*N*-((1-tosyl-1*H*-indol-3-yl)ethynyl)benzenesulfonamide, 1h

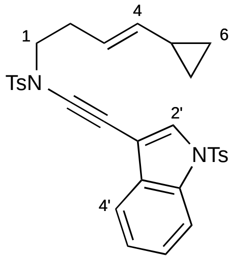

Prepared by General Procedure C using sulfonamide **6a** (50 mg, 0.19 mmol, 1.0 equiv.) and 3-(bromoethynyl)-1-tosyl-1*H*-indole (85 mg, 0.23 mmol, 1.2 equiv.). The crude material was purified by column chromatography (petroleum ether / EtOAc (9:1)) to give **1h** as an orange oil (64 mg, 0.11 mmol, 61%); **R*f*** 0.16 (petroleum ether / EtOAc (9:1)); **IR** (thin film, ν_{max} / cm^{-1}) 3004, 2925, 2241, 1447, 1369, 1169, 1130, 1091; **^{1}H NMR** (500 MHz, $CDCl_3$) δ_H 7.96 (1H, d, *J* = 8.5 Hz, H4′), 7.84 (2H, d, *J* = 8.0 Hz, Ts*H*), 7.78 (2H, d, *J* = 8.5 Hz, Ts*H*), 7.67 (1H, s, H2′), 7.49 (1H, d, *J* = 8.0 Hz, H7′), 7.36-7.33 (3H, m, H6′ and Ts*H*), 7.28-7.24 (3H, m, H5′ and Ts*H*), 5.39 (1H, dt, *J* = 15.5 and 7.0 Hz, H3), 5.03 (1H, dd, *J* = 15.5 and 8.5 Hz, H4), 3.45 (2H, t, *J* = 7.5 Hz, H1), 2.47 (3H, s, TsC*H*3), 2.40-2.36 (5H, m, H2 and TsC*H*3), 1.33-1.26 (1H, m, H5), 0.67-0.63 (2H, m, H6), 0.31-0.28 (2H, m, H6); **^{13}C NMR** (125 MHz, $CDCl_3$) δ_C 145.4, 144.7, 137.6, 134.9, 134.7, 134.2, 131.2, 130.0, 129.8, 128.9, 127.7, 127.0, 125.4, 123.6, 122.4, 120.5, 113.5, 104.6, 86.0, 61.8, 51.6, 31.2, 21.7, 21.6, 13.6, 6.5; **HRMS** (ESI+) calc. for $C_{31}H_{30}N_2NaO_4S_2$ $[M+Na]^+$ 581.1539, found 581.1532.

(*E*)-*N*-(4-cyclopropylbut-3-en-1-yl)-4-methyl-*N*-((1-tosyl-1*H*-pyrrol-2-yl)ethynyl)benzenesulfonamide, 1i

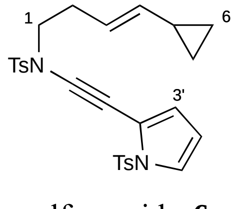

Prepared by General Procedure C using sulfonamide **6a** (93 mg, 0.35 mmol, 1.0 equiv.) and 2-(bromoethynyl)-1-tosyl-1*H*-pyrrole (228 mg, 0.70 mmol, 2.0 equiv.). The crude material was purified by column chromatography (petroleum ether / Et_2O (9:1)) to give **1i** as a colourless oil (114 mg, 0.23 mmol, 65%): **R*f*** 0.34 (petroleum ether / Et_2O (5:2)); **IR** (thin film, ν_{max}/ cm^{-1}) 2999, 2916,

2338, 2227, 1661, 1437, 1047, 1363, 1313, 1172, 1019; **^{1}H NMR** (500 MHz, $CDCl_3$) δ_H 7.86-7.84 (4H, m, Ts*H*), 7.36-7.33 (3H, m, Ts*H* and H5′), 7.29-7.27 (2H, d, *J* = 8.0 Hz, Ts*H*), 6.45 (1H, dd, *J* = 3.5 and 1.5 Hz, H3′), 6.19 (1H, t, *J* = 3.5 Hz, H4′), 5.35 (1H, dt, *J* = 15.5 and 7.0 Hz, H3), 5.04 (1H, dd, *J* = 15.5 and 8.5 Hz, H4), 3.38 (2H, t, *J* = 7.5 Hz, H1), 2.45 (3H, s, TsCH_3), 2.40 (3H, s, TsCH_3), 2.33 (2H, td, *J* = 7.5 and 7.0 Hz, H2), 1.33-1.27 (1H, m, H5), 0.66-0.62 (2H, m, H6), 0.30-0.28 (2H, m, H6); **^{13}C NMR** (125 MHz, $CDCl_3$) δ_C 145.3, 144.8, 137.7, 135.6, 135.1, 130.1, 130.0, 128.0, 127.9, 123.8, 122.6 (2C), 115.1, 111.7, 87.1, 61.6, 51.8, 31.2, 21.8 (2C), 13.8, 6.7; **HRMS** (ESI+) calc. for $C_{27}H_{28}N_2NaO_4S_2$ $[M+Na]^+$ 531.1383, found 531.1382

(*E*)-*N*-(4-cyclopropylbut-3-en-1-yl)-4-nitro-*N*-(phenylethynyl)benzenesulfonamide, 1k

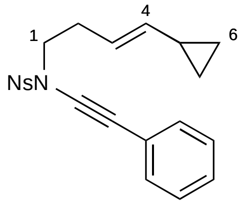

Prepared by General Procedure C using sulfonamide **6f** (100 mg, 0.34 mmol, 1.0 equiv.) and 1-bromo-2-phenylacetylene (92 mg, 0.51 mmol, 1.5 equiv.). The crude material was purified by column chromatography (petroleum ether / EtOAc (9:1)) to give **1k** as a colourless oil (116 mg, 0.29 mmol, 87%); **R*f*** 0.36 (petroleum ether / EtOAc (9:1)); **IR** (thin film, ν_{max} / cm^{-1}) 3105, 3004, 2238, 1530, 1371, 1348, 1172, 1070; **^{1}H NMR** (500 MHz, $CDCl_3$) δ_H 8.41 (2H, d, *J* = 9.0 Hz, Ns*H*), 8.14 (2H, d, *J* = 9.0 Hz, Ns*H*), 7.38-7.36 (2H, m, Ph*H*), 7.32-7.30 (3H, m, Ph*H*), 5.34 (1H, dt, *J* = 15.0 and 7.0 Hz, H3), 5.03 (1H, dd, *J* = 15.0 and 8.5 Hz, H4), 3.50 (2H, t, *J* = 7.5 Hz, H1), 2.39 (2H, td, *J* = 7.5 and 7.0 Hz, H2), 1.29-1.23 (1H, m, H5), 0.67-0.63 (2H, m, H6), 0.30-0.27 (2H, m, H6); **^{13}C NMR** (125 MHz, $CDCl_3$) δ_C 150.6, 143.1, 138.1, 131.6, 128.9, 128.5, 128.4, 124.4, 122.1, 122.0, 80.8, 71.6, 52.0, 31.2, 13.7, 6.6; **HRMS** (ESI+) calc. for $C_{21}H_{20}O_4N_2NaS$ $[M+Na]^+$ 419.1036, found 419.1030.

***N*-(2-((*E*)-2-cyclopropylvinyl)phenyl)-*N*-((*E*)-1,2-dichlorovinyl)-4-methylbenzenesulfonamide, S1**

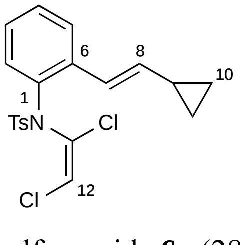

Prepared by General Procedure D using sulfonamide **6g** (280 mg, 0.89 mmol, 1.0 equiv.). The crude material was purified by column chromatography (petroleum ether / EtOAc (98:2)) to give **S1** as a

colourless oil (308 mg, 0.76 mmol, 84%); **R$_f$** 0.49 (petroleum ether / EtOAc (9:1)); **IR** (thin film, ν_{max} / cm^{-1}) 3084, 3006, 2923, 2852, 1645, 1597, 1402, 1168; **^{1}H NMR** (400 MHz, $CDCl_3$) δ_H 7.66 (2H, d, *J* = 8.5 Hz, Ts*H*), 7.49 (1H, d, *J* = 8.0 Hz, H5), 7.29 (1H, dd, *J* = 8.0 and 7.5 Hz, H4), 7.26 (2H, d, *J* = 8.5 Hz, Ts*H*), 7.23 (1H, d, *J* = 8.0 Hz, H2), 7.10 (1H, dd, *J* = 8.0 and 7.5 Hz, H3), 6.93 (1H, d, *J* = 16.0 Hz, H7), 6.43 (1H, s, H12), 5.62 (1H, dd, *J* = 16.0 and 9.0 Hz, H8), 2.44 (3H, s, TsC*H*$_3$), 1.52-1.43 (1H, m, H9), 0.81-0.76 (2H, m, H10), 0.47-0.43 (2H, m, H10); **^{13}C NMR** (100 MHz, $CDCl_3$) δ_C 144.8, 138.9, 137.4, 135.7, 133.6, 131.9, 130.9, 129.8, 129.6, 129.1, 126.7, 126.1, 123.6, 119.2, 21.9, 14.9, 7.5; **HRMS** (ESI+) calc. for $C_{20}H_{19}O_2N_{35}Cl_2NaS$ [M+Na]$^+$ 430.0406, found 430.0399.

(*E*)-*N*-(2-(2-cyclopropylvinyl)phenyl)-4-methyl-*N*-(prop-1-yn-1-yl)benzenesulfonamide, 1j

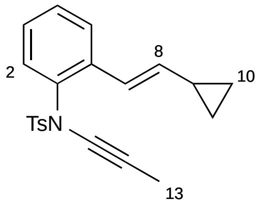

Prepared by General Procedure E using dichloroenamide **S1** (250 mg, 0.61 mmol, 1.0 equiv.). The crude material was purified by column chromatography (petroleum ether/EtOAc (95:5)) to give **1j** as a colourless oil (216 mg, 0.61 mmol, >99%); **R$_f$** 0.36 (petroleum ether / EtOAc (9:1)); **IR** (thin film, ν_{max} / cm^{-1}) 3004, 2919, 2257, 1645, 1597, 1483, 1449, 1366, 1171; **^{1}H NMR** (400 MHz, $CDCl_3$) δ_H 7.71 (2H, d, *J* = 8.5 Hz, Ts*H*), 7.50 (1H, d, *J* = 8.0 Hz, H5), 7.33 (2H, d, *J* = 8.5 Hz, Ts*H*), 7.26 (1H, dd, *J* = 8.0 and 7.5 Hz, H4), 7.10 (1H, dd, *J* = 8.0 and 7.5 Hz, H3), 6.93 (1H, d, *J* = 8.0 Hz, H2), 6.57 (1H, d, *J* = 16.0 Hz, H7), 5.71 (1H, dd, *J* = 16.0 and 9.0 Hz, H8), 2.47 (3H, s, H13), 1.91 (3H, s, TsC*H*$_3$), 1.52-1.45 (1H, m, H9), 0.83-0.79 (2H, m, H10), 0.49-0.46 (2H, m, H10); **^{13}C NMR** (100 MHz, $CDCl_3$) δ_C 144.7, 137.7, 136.9, 35.6, 134.5, 129.6, 129.3, 128.9, 128.5, 127.2, 125.9, 122.2, 73.5, 64.8, 21.9, 15.0, 7.7, 3.5; **HRMS** (ESI+) calc. for $C_{21}H_{21}O_2NNaS$ [M+Na]$^+$ 374.1185, found 374.1179.

(*R*, *E*)-*N*-(4-cyclopropyl-2-phenylbut-3-en-1-yl)-4-methyl-*N*-(oct-1-yn-1-yl)benzenesulfonamide, 1l

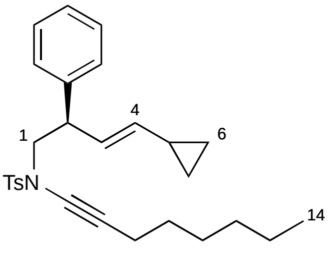

Prepared by General Procedure C using (*R*,*E*)-*N*-(4-cyclopropyl-2-phenylbut-3-en-1-yl)-4-

methylbenzenesulfonamide **6b** (100 mg, 0.29 mmol, 1.0 equiv.) and 1-bromooct-1-yne (90 mg, 0.44 mmol, 1.5 equiv.). The crude material was purified by column chromatography (petroleum ether / EtOAc (9:1)) to give **1l** as a colourless oil (103 mg, 0.22 mmol, 76%); $[\boldsymbol{\alpha}]_{\boldsymbol{D}}^{\mathbf{25}}$ −19.5 (*c* = 1.0, $CHCl_3$); **R*f*** 0.46 (petroleum ether / EtOAc (9:1)); **IR** (thin film, ν_{max} / cm^{-1}) 3064, 3028, 2955, 2858, 2253, 1598, 1365, 1168, 1092; **^{1}H NMR** (700 MHz, $CDCl_3$) δ_H 7.66 (2H, d, *J* = 8.5 Hz, Ts*H*), 7.29-7.25 (4H, m, Ph*H*), 7.21 (1H, t, *J* = 7.0 Hz, Ph*H*), 7.18 (2H, d, *J* = 8.5 Hz, Ts*H*), 5.62 (1H, dd, *J* = 15.5 and 8.0 Hz, H3), 5.02 (1H, dd, *J* = 15.5 and 9.0 Hz, H4), 3.67 (1H, q, *J* = 8.0 Hz, H2), 3.53 (1H, dd, *J* = 13.0 and 8.5 Hz, H1), 3.48 (1H, dd, *J* = 13.0 and 7.5 Hz, H1), 2.42 (3H, s, TsC*H*3), 2.24 (2H, t, *J* = 7.0 Hz, H9), 1.48-1.43 (2H, m, H10), 1.36-1.24 (7H, m, H5 and H11-13), 0.90 (3H, t, *J* = 7.5 Hz, H14), 0.68-0.63 (2H, m, H6), 0.34-0.28 (2H, m, H6); **^{13}C NMR** (176 MHz, $CDCl_3$) δ_C 144.0, 141.3, 136.7, 134.8, 129.5, 128.6, 128.0, 127.7, 127.0, 126.8, 73.0, 70.9, 55.9, 47.1, 31.4, 29.0, 28.5, 22.6, 21.6, 18.5, 14.1, 13.8, 6.7, 6.6; **HRMS** (ESI+) calc. for $C_{28}H_{35}NNaO_2S$ $[M+Na]^+$ 472.2281, found 472.2281.

(*E*)-*N*-(4-cyclopropyl-2-phenylbut-3-en-1-yl)-4-methyl-*N*-(phenylethynyl)benzenesulfonamide, 1m

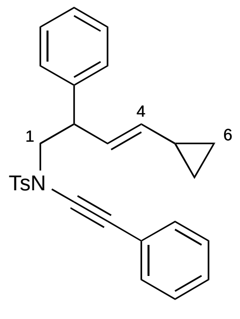

Prepared by General Procedure C using sulfonamide **rac-6b** (100 mg, 0.29 mmol, 1.0 equiv.) and 1-bromo-2-phenylacetylene (80 mg, 0.44 mmol, 1.5 equiv.). The crude material was purified by column chromatography (petroleum ether / EtOAc (9:1)) to give **1m** as a colourless oil (113 mg, 0.26 mmol, 88%); **R*f*** 0.31 (petroleum ether / EtOAc (9:1)); **IR** (thin film, ν_{max} / cm^{-1}) 3062, 3028, 2926, 2235, 1598, 1443, 1307, 1168, 1020; **^{1}H NMR** (700 MHz, $CDCl_3$) δ_H 7.73 (2H, d, *J* = 8.5 Hz, Ts*H*), 7.34-7.28 (9H, m, Ph*H*), 7.23-7.20 (3H, m, Ph*H* and Ts*H*), 5.65 (1H, dd, *J* = 15.5 and 8.0 Hz, H3), 5.05 (1H, dd, *J* = 15.5 and 9.0 Hz, H4), 3.75 (1H, q, *J* = 8.0 Hz, H2), 3.68-3.64 (2H, m, H1), 2.43 (3H, s, TsC*H*3), 1.36-1.31 (1H, m, H5), 0.67-0.62 (2H, m, H6), 0.34-0.29 (2H, m, H6); **^{13}C NMR** (176 MHz, $CDCl_3$) δ_C 144.5, 141.1, 137.0, 134.7, 131.4, 129.7, 128.7, 128.3, 127.9, 127.8, 127.7, 126.9, 126.8, 123.0, 82.5, 71.2, 56.2, 47.5, 21.7, 13.8, 6.7, 6.6; **HRMS** (ESI+) calc. for $C_{28}H_{27}NNaO_2S$ $[M+Na]^+$ 464.1655, found 464.1663.

(*E*)-*N*-(4-cyclopropyl-2-(2-((4-methoxybenzyl)oxy)ethyl)but-3-en-1-yl)-4-methyl-*N*-(phenylethynyl)benzenesulfonamide, 1n

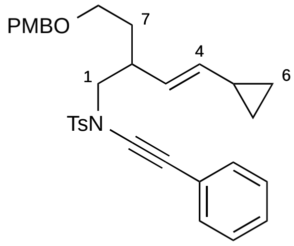

Prepared by General Procedure C using (*E*)-*N*-(4-cyclopropyl-2-(2-((4-methoxybenzyl)oxy)ethyl)but-3-en-1-yl)-4-methylbenzenesulfonamide **6c** (100 mg, 0.23 mmol, 1.0 equiv.) and 1-bromo-2-phenylacetylene (63 mg, 0.35 mmol, 1.5 equiv.). The crude material was purified by column chromatography (petroleum ether / EtOAc (9:1)) to give **1n** as a colourless oil (102 mg, 0.19 mmol, 83%); **R*f*** 0.54 (petroleum ether / EtOAc (4:1)); **IR** (thin film, ν_{max} / cm^{-1}) 3002, 2930, 2860, 2235, 1612, 1513, 1364, 1247, 1168, 1090; **^{1}H NMR** (500 MHz, $CDCl_3$) δ_H 7.82 (2H, d, *J* = 8.0 Hz, Ts*H*), 7.35-7.32 (4H, m, Ts*H* and Ph*H*), 7.29-7.26 (3H, m, Ph*H*), 7.23 (2H, d, *J* = 8.5 Hz, PMB*H*), 6.82 (2H, d, *J* = 8.5 Hz, PMB*H*), 5.20 (1H, dd, *J* = 15.5 and 9.0 Hz, H3), 4.96 (1H, dd, *J* = 15.5 and 8.5 Hz, H4), 4.43 (1H, d, *J* = 11.5 Hz, PMBCH_2), 4.35 (1H, d, *J* = 11.5 Hz, PMBCH_2), 3.78 (3H, s, PMBCH_3), 3.52-3.42 (2H, m, H8), 3.37 (1H, dd, *J* = 12.5 and 7.0 Hz, H1), 3.29 (1H, dd, *J* = 12.5 and 8.0 Hz, H1), 2.70-2.62 (1H, m, H2), 2.44 (3H, s, TsCH_3), 1.89-1.83 (1H, m, H7), 1.51-1.44 (1H, m, H7), 1.31-1.24 (1H, m, H5), 0.66-0.59 (2H, m, H6), 0.29-0.24 (2H, m, H6); **^{13}C NMR** (125 MHz, $CDCl_3$) δ_C 159.1, 144.5, 137.4, 134.8, 131.4, 130.7, 129.7, 129.3, 128.3, 127.8, 127.7, 127.0, 123.1, 113.7, 82.8, 72.4, 70.9, 67.4, 56.0, 55.3, 38.7, 32.0, 21.7, 13.6, 6.6; **HRMS** (ESI+) calc. for $C_{32}H_{36}O_4NS$ $[M+H]^+$ 530.2360, found 530.2350.

(*E*)-*N*-(5-cyclopropylpent-4-en-2-yl)-4-methyl-*N*-(phenylethynyl)benzenesulfonamide, 1o

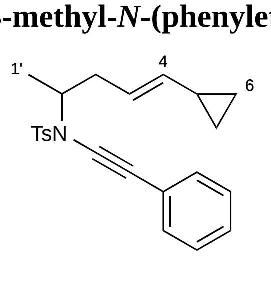

Prepared by General Procedure C using (*E*)-*N*-(5-cyclopropylpent-4-en-2-yl)-4-methylbenzenesulfonamide **6d** (100 mg, 0.36 mmol, 1.0 equiv.) and 1-bromo-2-phenylacetylene (130 mg, 0.72 mmol, 2.0 equiv.), with the reaction mixture stirred at 115 °C for 16 h. The crude material was purified by column chromatography (petroleum ether / EtOAc (95:5)) to give **1o** as a colourless oil (113 mg, 0.30 mmol, 83%); **R*f*** 0.72 (petroleum ether / EtOAc (9:1)); **IR** (thin film, ν_{max} / cm^{-1}) 3081, 3003, 2981, 2929, 2233, 1598, 1363, 1169, 1091, 972; **^{1}H NMR** (500 MHz, $CDCl_3$) δ_H 7.84 (2H, d, *J* = 8.0 Hz, Ts*H*), 7.39-7.25 (7H, m, Ph*H* and Ts*H*), 5.25 (1H, dt, *J* = 15.0

and 7.0 Hz, H3), 4.98 (1H, dd, *J* = 15.0 and 8.5 Hz, H4), 4.05 (1H, sext, *J* = 7.0 Hz, H1), 2.44 (3H, s, TsCH_3), 2.29 (1H, dt, *J* = 14.0 and 7.0 Hz, H2), 2.13 (1H, dt, *J* = 14.0 and 7.0 Hz, H2), 1.24-1.18 (1H, m, H5), 1.16 (3H, d, *J* = 7.0 Hz, H1'), 0.66-0.60 (2H, m, H6), 0.30-0.23 (2H, m, H6); **^{13}C NMR** (500 MHz, $CDCl_3$) δ_C 144.4, 137.7, 136.1, 131.4, 129.8, 128.4, 127.7, 127.7, 123.4, 122.9, 79.7, 72.9, 57.1, 38.3, 21.8, 18.9, 13.7, 6.6, 6.6; **HRMS** (ESI+) calc. for $C_{23}H_{25}NNaO_2S$ $[M+Na]^+$ 402.1498, found 402.1500.

N-((2*S*,3*R*,*E*)-5-cyclopropyl-3-phenylpent-4-en-2-yl)-*N*-((*E*)-1,2-dichlorovinyl)-4-methylbenzenesulfonamide, S2

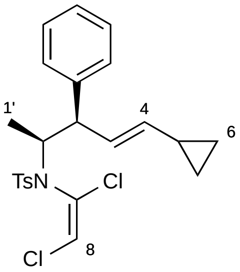

Prepared by General Procedure D using *N*-((2*S*,3*R*,*E*)-5-cyclopropyl-3-phenylpent-4-en-2-yl)-4-methylbenzenesulfonamide **6e** (200 mg, 0.56 mmol, 1.0 equiv.). The crude material was purified by column chromatography (petroleum ether/EtOAc (98:2)) to give **S2** as a colourless oil (192 mg, 0.43 mmol, 76%); $[\alpha]_D^{25}$ –63.0 (*c* = 1.0, $CHCl_3$); $\mathbf{R_f}$ 0.45 (petroleum ether / EtOAc (9:1)); **IR** (thin film, ν_{max} / cm^{-1}) 3084, 3002, 1598, 1494, 1451, 1356, 1165, 1090, 1020; **^{1}H NMR** (400 MHz, $CDCl_3$)* δ_H 7.85-7.75 (2H, m, Ts*H*), 7.31-7.10 (7H, m, Ph*H* and Ts*H*), 6.54-6.42 (1H, m, H8), 5.63 (1H, dd, *J* = 15.0 and 10.0 Hz, H3), 5.13-5.01 (0.5H, m, H4), 4.81-4.67 (0.5H, m, H4), 4.33-4.26 (1H, m, H1), 3.85-3.70 (1H, m, H2), 2.43 (3H, s, TsCH_3), 1.38-1.13 (4H, m, H1′ and H5), 0.68-0.57 (2H, m, H6), 0.34-0.19 (2H, m H6); **^{13}C NMR** (125 MHz, $CDCl_3$)* δ_C 144.5, 144.3, 143.0, 137.9, 137.4, 136.6, 129.7, 128.7, 128.6, 128.2, 126.5, 125.0, 124.7, 123.2, 122.9, 63.7, 60.8, 53.4, 51.9, 21.8, 15.9, 15.5, 13.9, 6.6, 6.5; **HRMS** (ESI+) calc. for $C_{23}H_{25}O_2NCl_2NaS$ $[M+Na]^+$ 472.0875, found 472.0874. *Note that the spectra are highly rotameric.

N-((2S,3R,E)-5-cyclopropyl-3-phenylpent-4-en-2-yl)-4-methyl-N-(prop-1-yn-1-yl)benzenesulfonamide, 1r

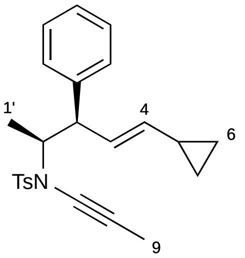

Prepared by General Procedure E using dichloroenamide **S2** (50 mg, 0.11 mmol, 1.0 equiv.) and iodomethane (8 µL, 0.24 mmol, 1.2 equiv.). The crude material was purified by column chromatography (petroleum ether / EtOAc (95:5)) to give **1r** as a colourless oil (42 mg, 0.11 mmol, 95%); $[\alpha]_D^{25}$ +22.50 (c = 1.0, $CHCl_3$); **R$_f$** 0.36 (petroleum ether / EtOAc (9:1)); **IR** (thin film, ν_{max} / cm^{-1}) 3003, 2920, 2249, 1663, 1495, 1356, 1166, 1090; **^{1}H NMR** (500 MHz, $CDCl_3$) δ_H 7.32 (2H, d, J = 8.5 Hz, TsH), 7.24-7.21 (2H, m, PhH), 7.19-7.17 (3H, m, PhH), 7.11 (2H, d, J = 8.5 Hz, TsH), 5.62 (1H, dd, J = 15.0 and 9.5 Hz, H3), 5.03 (1H, dd, J = 15.0 and 8.5 Hz, H4), 4.30 (1H, dq, J = 10.0 and 6.5 Hz, H1), 3.35 (1H, dd, J = 10.0 and 9.5 Hz, H2), 2.38 (3H, s, H9), 1.92 (3H, s, TsCH_3), 1.37-1.30 (1H, m, H5), 1.22 (3H, d, J = 6.5 Hz, H1′), 0.69-0.60 (2H, m, H6), 0.35-0.24 (2H, m, H6); **^{13}C NMR** (125 MHz, $CDCl_3$) δ_C 143.7, 142.4, 137.1, 135.8, 129.4, 128.5, 127.9, 127.6, 127.1, 126.5, 69.1, 68.3, 59.5, 54.2, 21.7, 17.6, 13.8, 6.7, 6.6, 3.6; **HRMS** (ESI+) calc. for $C_{24}H_{27}O_2NNaS$ $[M+Na]^+$ 416.1655, found 416.1652.

N-((R,E)-5-cyclopropyl-1-((4-methoxybenzyl)oxy)pent-4-en-2-yl)-N-((E)-1,2-dichlorovinyl)-4-methylbenzenesulfonamide, S3

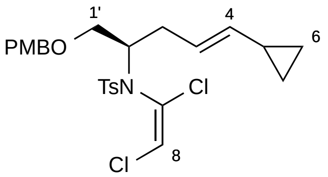

Prepared by General Procedure D using (R,E)-N-(5-cyclopropyl-1-((4-methoxybenzyl)oxy)pent-4-en-2-yl)-4-methylbenzenesulfonamide **6h** (200 mg, 0.48 mmol, 1.0 equiv.). The crude material was purified by column chromatography (petroleum ether / EtOAc (95:5)) to give **S3** as a colourless oil (176 mg, 0.34 mmol, 72%); $[\alpha]_D^{25}$ +14.4 (c = 1.0, $CHCl_3$); **R$_f$** 0.27 (petroleum ether / EtOAc (9:1)); **IR** (thin film, ν_{max} / cm^{-1}) 3082, 3004, 2934, 2863, 1664, 1514, 1357, 1248, 1165, 1091; **^{1}H NMR** (400 MHz, $CDCl_3$)* δ_H 7.87-7.80 (2H, m, TsH), 7.28-7.09 (4H, m, TsH and PMBH), 6.87-6.82 (2H, m, PMBH), 6.61-6.58 (1H, m, H8), 5.42-5.35 (0.5H, m, H3), 5.11-4.97 (1H, m, H3 and H4), 4.86-4.80 (0.5H, m, H4), 4.42-4.32 (1H, m, PMBCH_2), 4.26-4.16 (1H, m, PMBCH_2), 4.02-3.91 (1H, m, H1), 3.81 (3H, s, PMBCH_3), 3.65-3.55 (1H, m, H1′), 3.46-3.44 (1H, m, H1′), 2.49-2.31 (4.5H, m,

TsCH_3 and H2), 2.18-2.11 (0.5H, m, H2), 1.29-1.09 (1H, m, H5), 0.65-0.61 (2H, m, H6), 0.28-0.21 (2H, m, H6); ^{13}**C NMR** (100 MHz, $CDCl_3$)* δ_C 159.1, 144.3, 137.4, 137.3, 136.6, 129.6, 129.4, 129.3, 129.2, 128.6, 128.5, 123.3, 123.1, 113.7, 72.8, 72.6, 70.5, 69.3, 61.9, 55.2, 34.5, 32.9, 21.7, 13.5, 6.5; **HRMS** (ESI+) calc. for $C_{25}H_{29}O_4NCl_2NaS$ $[M+Na]^+$ 532.1087, found 532.1086. *Note that the spectra are highly rotameric.

(*R*,*E*)-*N*-(5-cyclopropyl-1-((4-methoxybenzyl)oxy)pent-4-en-2-yl)-4-methyl-*N*-(prop-1-yn-1-yl)benzenesulfonamide, 1p

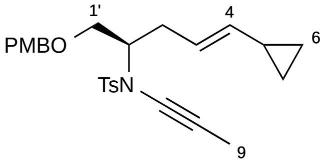

Prepared by General Procedure E using dichloroenamide **S3** (120 mg, 0.24 mmol, 1.0 equiv.) and iodomethane (18 µL, 0.28 mmol, 1.2 equiv.). The crude material was purified by column chromatography (petroleum ether/EtOAc (9:1)) to give **1p** as a colourless oil (93 mg, 0.21 mmol, 86%); $[\alpha]_D^{25}$ +18.7 (*c* = 1.0, $CHCl_3$); **R*f*** 0.19 (petroleum ether / EtOAc (9:1)); **IR** (thin film, ν_{max} / cm^{-1}) 3003, 2918, 2256, 1613, 1513, 1357, 1248, 1166, 1092; ^{1}**H NMR** (500 MHz, $CDCl_3$) δ_H 7.78 (2H, d, *J* = 8.5 Hz, Ts*H*), 7.22 (2H, d, *J* = 8.5 Hz, Ts*H*), 7.13 (2H, d, *J* = 8.5 Hz, PMB*H*), 6.84 (2H, d, *J* = 8.5 Hz, PMB*H*), 5.21 (1H, dt, *J* = 15.0 and 7.0 Hz, H3), 4.97 (1H, dd, *J* = 15.0 and 8.5 Hz, H4), 4.36 (1H, d, *J* = 11.5 Hz, PMBCH_2), 4.28 (1H, d, *J* = 11.5 Hz, PMBCH_2), 4.10-4.04 (1H, m, H1), 3.81 (3H, s, PMBCH_3), 3.45 (1H, dd, *J* = 10.0 and 8.5 Hz, H1′), 3.36 (1H, dd, *J* = 10.0 and 5.0 Hz, H1′), 2.39 (3H, s, TsCH_3), 2.19-2.16 (2H, m, H2), 1.88 (3H, s, H9), 1.23-1.16 (1H, m, H5), 0.66-0.59 (2H, m, H6), 0.30-0.23 (2H, m, H6); ^{13}**C NMR** (125 MHz, $CDCl_3$) δ_C 159.2, 143.8, 137.6, 136.2, 130.3, 129.4, 129.3, 128.1, 122.6, 113.7, 72.6, 69.9, 68.8, 67.8, 59.8, 55.4, 33.3, 21.8, 13.7, 6.6, 6.6, 3.7; **HRMS** (ESI+) calc. for $C_{26}H_{31}O_4NNaS$ $[M+Na]^+$ 476.1866, found 476.1858.

***N*-((*R*,*E*)-1-((*tert*-butyldimethylsilyl)oxy)-5-cyclopropylpent-4-en-2-yl)-*N*-((*E*)-1,2-dichlorovinyl)-4-methylbenzenesulfonamide, S4**

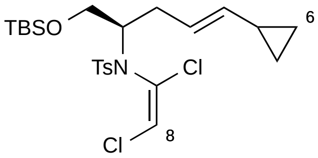

Prepared by General Procedure D using (*R*,*E*)-*N*-(1-((*tert*-butyldimethylsilyl)oxy)-5-cyclopropylpent-4-en-2-yl)-4-methylbenzenesulfonamide **6i** (62 mg, 0.15 mmol, 1.0 equiv.). The crude material was purified by column chromatography (petroleum ether / EtOAc (98:2)) to give **S4** as a colourless oil (69 mg, 0.14 mmol, 91%); $[\alpha]_D^{25}$ +25.0 (*c* = 0.2, $CHCl_3$); **R*f*** 0.47 (petroleum

ether / Et_2O (9:1)); **IR** (thin film, ν_{max} / cm^{-1}) 3084, 2954, 2928, 2885, 2856, 1598, 1495, 1359, 1254, 1166; **^{1}H NMR** (400 MHz, $CDCl_3$) δ_H 7.86 (2H, d, *J* = 8.0 Hz, Ts*H*), 7.31 (2H, d, *J* = 8.0 Hz, Ts*H*), 6.57-6.52 (1H, m, H8), 5.45-5.34 (0.5H, m, H3), 5.11-4.96 (0.5H and 0.5H, m, H3 and H4), 4.86-4.80 (0.5H, m, H4), 3.85-3.73 (2.5H, m, H1 and H1′), 3.67-3.52 (0.5H, m, H1′), 2.59-2.30 (4.5H, m, H2 and TsCH_3), 2.06-1.96 (0.5H, m, H2), 1.32-1.10 (1H, m, H5), 0.89-0.80 (9H, m, SiC(CH_3)$_3$), 0.66-0.59 (2H, m, H6), 0.29-0.20 (2H, m, H6), 0.05–0.10 (6H, m, Si(CH_3)$_2$); **^{13}C NMR** (100 MHz, $CDCl_3$) δ_C 144.4, 137.3, 136.8, 129.7, 128.6, 123.7, 123.0, 123.0, 63.9, 63.8, 32.1, 25.9, 21.8, 18.3, 13.7, 6.5, 6.5, –5.4, –5.6; **HRMS** (ESI+) calc. for $C_{23}H_{35}O_3NCl_2NaSSi$ $[M+Na]^+$ 526.1376, found 526.1379.

(*R*,*E*)-*N*-(1-((*tert*-butyldimethylsilyl)oxy)-5-cyclopropylpent-4-en-2-yl)-4-methyl-*N*-(prop-1-yn-1-yl)benzenesulfonamide, 1q

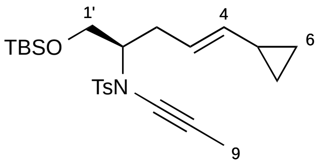

Prepared by General Procedure E using dichloroenamide **S4** (69 mg, 0.14 mmol, 1.0 equiv.) and iodomethane (10 µL, 0.16 mmol, 1.2 equiv.). The crude material was purified by column chromatography (petroleum ether / Et_2O (98:1)) to give **1q** as a colourless oil (51 mg, 0.11 mmol, 83%); $[\alpha]_D^{25}$ +7.5 (*c* = 0.4, $CHCl_3$); **R_f** 0.42 (petroleum ether / Et_2O (9:1)); **IR** (thin film, ν_{max} / cm^{-1}) 2954, 2928, 2856, 2256, 1598, 1471, 1436, 1253, 1168, 1120, 1093; **^{1}H NMR** (500 MHz, $CDCl_3$) δ_H 7.78 (2H, d, *J* = 8.5 Hz, Ts*H*), 7.29 (2H, d, *J* = 8.5 Hz, Ts*H*), 5.12 (1H, dt, *J* = 15.0 and 7.0 Hz, H3), 4.93 (1H, d, *J* = 15.0 and 8.5 Hz, H4), 3.84 (1H, dq, *J* = 9.0 and 6.0 Hz, H1), 3.62 (1H, dd, *J* = 10.5 and 6.0 Hz, H1′), 3.54 (1H, dd, *J* = 10.5 and 6.0 Hz, H1′), 2.41 (3H, s, TsCH_3), 2.29-2.22 (1H, m, H2), 2.16-2.10 (1H, m, H2), 1.90 (3H, s, H9), 1.13 (1H, m, H5), 0.85 (9H, s, SiC(CH_3)$_3$), 0.64-0.56 (2H, m, H6), 0.28-0.20 (2H, m, H6), 0.00 (6H, s, Si(CH_3)$_2$); **^{13}C NMR** (125 MHz, $CDCl_3$) δ_C 143.9, 137.3, 136.4, 129.6, 127.8, 123.0, 69.2, 67.4, 63.9, 61.8, 32.7, 25.9, 21.8, 18.4, 13.7, 6.6, 6.6, 3.6, –5.4, –5.4; **HRMS** (ESI+) calc. for $C_{24}H_{37}O_3NNaSSi$ $[M+Na]^+$ 470.2156, found 470.2153.

N-((E)-4-((1S*,2S*)-2-(((tert-butyldiphenylsilyl)oxy)methyl)cyclopropyl)but-3-en-1-yl)-4-methyl-N-(oct-1-yn-1-yl)benzenesulfonamide, 1s

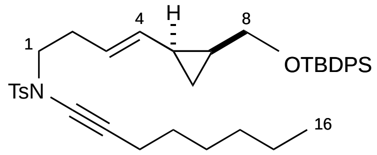

Prepared by General Procedure C using *N*-((*E*)-4-((1*S**,2*S**)-2-(((*tert*-butyldiphenylsilyl)oxy)methyl)cyclopropyl)but-3-en-1-yl)-4-methylbenzenesulfonamide **6j** (100 mg, 0.19 mmol, 1.0 equiv.) and 1-bromooct-1-yne (71 mg, 0.37 mmol, 2.0 equiv.). The crude material was purified by column chromatography (petroleum ether / EtOAc (9:1)) to give **1s** as a colourless oil (63 mg, 0.10 mmol, 53%); $\mathbf{R_f}$ 0.55 (petroleum ether / EtOAc (9:1)); **IR** (thin film, ν_{max} / cm^{-1}) 3070, 2955, 2857, 2253, 1494, 1404, 1185, 1090; **^{1}H NMR** (500 MHz, $CDCl_3$) δ_H 7.76 (2H, d, *J* = 8.5 Hz, Ts*H*), 7.69-7.65 (4H, m, Ph*H*), 7.43-7.35 (6H, m, Ph*H*), 7.30 (2H, d, *J* = 8.5 Hz, Ts*H*), 5.36 (1H, dt, *J* = 15.5 and 7.0 Hz, H3), 5.24 (1H, dd, *J* = 15.5 and 8.0 Hz, H4), 3.71 (1H, dd, *J* = 11.0 and 6.0 Hz, H8), 3.54 (1H, dd, *J* = 11.0 and 8.0 Hz, H8), 3.23 (2H, t, *J* = 8.0 Hz, H1), 2.42 (3H, s, TsCH_3), 2.30-2.27 (2H, m, H2), 2.25 (2H, t, *J* = 7.0 Hz, H11), 1.50-1.44 (3H, m, H5 and H12), 1.36-1.23 (7H, m, H7 and H13-15), 1.04 (9H, s, SiC(CH_3)$_3$), 0.88 (3H, t, *J* = 7.5 Hz, H16), 0.82-0.78 (1H, m, H6), 0.28-0.25 (1H, m, H6); **^{13}C NMR** (125 MHz, $CDCl_3$) δ_C 144.3, 135.7, 135.7, 134.9, 134.2, 134.1, 132.3, 129.7, 129.7, 127.8, 127.7, 127.7, 125.6, 73.1, 70.6, 64.0, 51.5, 31.5, 31.4, 29.1, 28.6, 27.0, 22.7, 21.7, 20.5, 19.3, 18.8, 18.6, 14.2, 10.4; **HRMS** (ESI+) calc. for $C_{39}H_{51}O_3NNaSSi$ [M+Na]$^+$ 664.3251, found 664.3234.

[5+2] Cycloisomerization products

(R)-8-phenyl-1-tosyl-1,2,3,3a,6,7-hexahydrocyclohepta[b]pyrrole, 7a

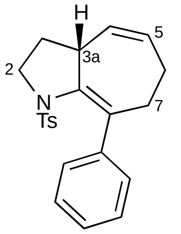

Method A: Prepared by General Procedure F using ynamide **1a** (20 mg, 55 μmol, 1.0 equiv.), with a reaction time of 3 h. The crude material was purified by column chromatography (petroleum ether / EtOAc (95:5)) to give **rac-7a** as a colourless oil (18 mg, 50 μmol, 91%).

Method B: Prepared by General Procedure G using ynamide **1a** (20 mg, 55 μmol, 1.0 equiv.) and (*S*,*R*,*R*)-**L5**, with a reaction time of 5 min. The crude material was purified by column

chromatography (petroleum ether / EtOAc (95:5)) to give **(*R*)-7a** as a colourless oil (15 mg, 41 μmol, 75%).

$[\alpha]_D^{25}$ –93.5 (*c* = 1.0, $CHCl_3$); 99% *ee* (CHIRALPAK IA, 5% IPA / hexane, 1.3 mL/min, t_R major – 11.86 min, minor – 12.71 min); $\mathbf{R_f}$ 0.30 (petroleum ether / EtOAc (9:1)); **IR** (thin film, ν_{max} / cm^{-1}) 2954, 2891, 1598, 1357, 1163, 1091; **^{1}H NMR** (500 MHz, C_6D_6) δ_H 7.50 (2H, d, *J* = 7.5 Hz, Ph*H*), 7.47 (2H, d, *J* = 8.0 Hz, Ts*H*), 7.23 (2H, t, *J* = 7.5 Hz, Ph*H*), 7.10 (1H, t, *J* = 7.5 Hz, Ph*H*), 6.68 (2H, d, *J* = 8.0 Hz, Ts*H*), 5.45-5.40 (1H, m, H5), 5.03 (1H, d, *J* = 11.0 Hz, H4), 3.51-3.45 (1H, m, H3a), 3.41 (1H, ddd, *J* = 12.5, 7.0 and 5.5 Hz, H2), 3.18 (1H, dt, *J* = 12.5 and 7.5 Hz, H2), 2.86 (1H, m, H7), 2.35 (1H, m, H7), 2.24-2.19 (1H, m, H6), 2.08-2.01 (1H, m, H6), 1.87 (3H, s, TsCH_3), 1.55-1.48 (1H, m, H3), 0.98-0.91 (1H, m, H3); **^{13}C NMR** (125 MHz, C_6D_6) δ_C 143.5, 142.7, 139.5, 137.9, 132.3, 130.9, 130.3, 129.2, 128.9, 128.6, 128.1, 126.5, 49.6, 40.4, 33.3, 32.3, 26.8, 21.1; **HRMS** (ESI+) calc. for $C_{22}H_{24}NO_2S$ $[M+H]^+$ 366.1522, found 366.1507; **EA** calc. for $C_{22}H_{23}NO_2S$: C, 72.3; H, 6.3; N, 3.8. Found: C, 72.4; H, 6.5; N, 3.8.

(*R*)-8-(4-fluorophenyl)-1-tosyl-1,2,3,3a,6,7-hexahydrocyclohepta[*b*]pyrrole, 7b

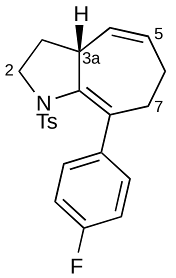

Method A: Prepared by General Procedure F using ynamide **1b** (20 mg, 52 μmol, 1.0 equiv.), with a reaction time of 15 min. The crude material was purified by column chromatography (petroleum ether / EtOAc (9:1)) to give **rac-7b** as a colourless oil (20 mg, 52 μmol, >99%).

Method B: Prepared by General Procedure G using ynamide **1b** (20 mg, 52 μmol, 1.0 equiv.) and (*S*,*R*,*R*)-**L5**, with a reaction time of 5 min. The crude material was purified by column chromatography (petroleum ether / EtOAc (9:1)) to give **(*R*)-7b** as a colourless oil (20 mg, 51 μmol, 98%).

$[\alpha]_D^{25}$ –44.3 (*c* = 1.0, $CHCl_3$); 99% *ee* (CHIRALPAK IA, 5% IPA / hexane, 1.3 mL/min, t_R major – 10.22 min, minor – 11.29 min); $\mathbf{R_f}$ 0.26 (petroleum ether / EtOAc (9:1)); **IR** (thin film, ν_{max} / cm^{-1}) 3065, 2923, 1599, 1508, 1352, 1227, 1162; **^{1}H NMR** (500 MHz, C_6D_6) δ_H 7.46 (2H, d, *J* = 8.5 Hz, Ts*H*), 7.27 (2H, dd, J_{HH} = 9.0 Hz and J_{HF} = 5.5 Hz, H10), 6.86 (2H, dd, J_{HH} = 9.0 Hz and J_{HF} = 9.0 Hz, H11), 6.69 (2H, d, *J* = 8.5 Hz, Ts*H*), 5.44-5.39 (1H, m, H5), 5.00 (1H, d, *J* = 11.5 Hz, H4), 3.46-3.38 (2H, m, H2 and H3a), 3.17 (1H, ddd, *J* = 12.5, 8.0 and 7.0 Hz, H2), 2.82-2.76 (1H, m, H7), 2.23-2.18 (1H, m, H7), 2.17-2.09 (1H, m, H6), 2.06-1.98 (1H, m, H6), 1.88 (3H, s, TsCH_3),

1.51-1.45 (1H, m, H3), 0.95-0.87 (1H, m, H3); **^{13}C NMR** (125 MHz, C_6D_6) δ_C 162.0 (d, J_{CF} = 245.0 Hz), 143.0, 139.8, 139.3 (d, J_{CF} = 3.5 Hz), 137.9, 131.0, 130.8, 130.5 (d, J_{CF} = 8.0 Hz), 130.2, 129.3, 128.0, 115.1 (d, J_{CF} = 21.5 Hz,), 49.7, 40.4, 33.2, 32.3, 26.7, 21.1; **^{19}F NMR** (470 MHz, C_6D_6) δ_F –116.0; **HRMS** (ESI+) calc. for $C_{22}H_{22}O_2NFNaS$ $[M+Na]^+$ 406.1248, found 406.1242.

(*R*)-8-(4-chlorophenyl)-1-tosyl-1,2,3,3a,6,7-hexahydrocyclohepta[*b*]pyrrole, 7c

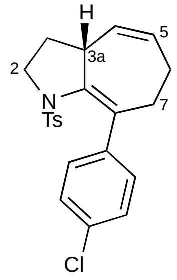

Method A: Prepared by General Procedure F using ynamide **1c** (20 mg, 50 μmol, 1.0 equiv.), with a reaction time of 15 min. The crude material was purified by column chromatography (petroleum ether / EtOAc (95:5)) to give **rac-7c** as a colourless oil (11 mg, 28 μmol, 56%).

Method B: Prepared by General Procedure G using ynamide **1c** (20 mg, 50 μmol, 1.0 equiv.) and (*S*,*R*,*R*)-**L5**, with a reaction time of 15 min. The crude material was purified by column chromatography (petroleum ether / EtOAc (95:5)) to give **(*R*)-7c** as a colourless oil (20 mg, 50 μmol, 99%).

$[\boldsymbol{\alpha}]_{\boldsymbol{D}}^{\mathbf{25}}$ –24.3 (c = 1.0, $CHCl_3$); 99% *ee* (CHIRALPAK IA, 5% IPA / hexane, 1.3 mL/min, t_R major – 10.46 min, minor – 12.20 min); **R_f** 0.23 (petroleum ether / EtOAc (9:1)); **IR** (thin film, ν_{max} / cm^{-1}) 3011, 2954, 2889, 1597, 1490, 1162, 1091; **^{1}H NMR** (500 MHz, C_6D_6) δ_H 7.42 (2H, d, J = 8.0 Hz, Ts*H*), 7.18 (2H, d, J = 8.5 Hz, H11), 7.12 (2H, d, J = 8.5 Hz, H10), 6.70 (1H, d, J = 8.0 Hz, Ts*H*), 5.44-5.39 (1H, m, H5), 5.01 (1H, d, J = 11.0 Hz, H4), 3.46-3.38 (2H, m, H3a and H2), 3.18 (1H, ddd, J = 12.5, 7.5 and 7.0 Hz, H2), 2.80-2.74 (1H, m, H7), 2.18-2.13 (1H, m, H7), 2.11-2.06 (1H, m, H6), 2.04-1.97 (1H, m, H6), 1.91 (3H, s, TsCH_3), 1.54-1.47 (1H, m, H3), 0.97-0.90 (1H, m, H3); **^{13}C NMR** (125 MHz, C_6D_6) δ_C 143.0, 141.7, 140.3, 138.0, 132.2, 130.7, 130.5, 130.3, 130.2, 129.3, 128.4, 127.8, 49.8, 40.5, 32.9, 32.4, 26.6, 21.2; **HRMS** (ESI+) calc. for $C_{22}H_{23}O_2NClS$ $[M+H]^+$ 400.1133, found 400.1135.

(*R*)-8-(4-methoxyphenyl)-1-tosyl-1,2,3,3a,6,7-hexahydrocyclohepta[*b*]pyrrole, 7d

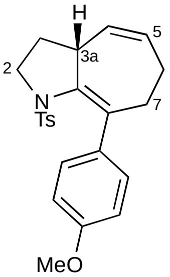

Method A: Prepared by General Procedure F using ynamide **1d** (20 mg, 51 μmol, 1.0 equiv.), with a reaction time of 20 h. The crude material was purified by column chromatography (petroleum ether / EtOAc (9:1)) to give **rac-7d** as a colourless oil (17 mg, 43 μmol, 85%).

Method B: Prepared by General Procedure G using ynamide **1d** (20 mg, 51 μmol, 1.0 equiv.) and (*S*,*R*,*R*)-**L5**, with a reaction time of 10 min. The crude material was purified by column chromatography (petroleum ether / EtOAc (9:1)) to give **(*R*)-7d** as a colourless oil (15 mg, 38 μmol, 75%).

$[\alpha]_D^{25}$ –79.3 (*c* = 1.0, $CHCl_3$); 94% *ee* (CHIRALPAK IC, 30% IPA / hexane, 1.0 mL/min, t_R major – 38.45 min, minor – 51.01 min); **R_f** 0.17 (petroleum ether / EtOAc (9:1)); **IR** (thin film, ν_{max} / cm^{-1}) 3010, 2953, 2835, 1607, 1510, 1353, 1246, 1161; **^{1}H NMR** (500 MHz, C_6D_6) δ_H 7.53 (2H, d, *J* = 8.0 Hz, H11), 7.44 (2H, d, *J* = 8.5 Hz, Ts*H*), 6.82 (2H, d, *J* = 8.5 Hz, Ts*H*), 6.69 (2H, d, *J* = 8.0 Hz, H10), 5.47-5.42 (1H, m, H5), 5.04 (1H, d, *J* = 11.5 Hz, H4), 3.52-3.44 (2H, m, H2 and H3a), 3.34 (3H, s, OCH_3), 3.29-3.20 (1H, m, H2), 2.91-2.85 (1H, m, H7), 2.40-2.35 (1H, m, H7), 2.27-2.18 (1H, m, H6), 2.11-2.05 (1H, m, H6), 1.88 (3H, s, TsCH_3), 1.55-1.48 (1H, m, H3), 0.98-0.91 (1H, m, H3); **^{13}C NMR** (125 MHz, C_6D_6) δ_C 158.9, 142.8, 139.1, 138.2, 135.8, 132.1, 131.2, 130.5, 130.2, 129.3, 128.3, 114.0, 54.9, 49.9, 40.7, 33.5, 32.6, 27.0, 21.3; **HRMS** (ESI+) calc. for $C_{23}H_{25}O_3NNaS$ $[M+Na]^+$ 418.1447, found 418.1442.

(*R*)-8-hexyl-1-tosyl-1,2,3,3a,6,7-hexahydrocyclohepta[*b*]pyrrole, 7e

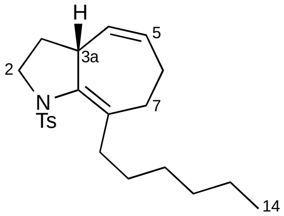

Method A: Prepared by General Procedure F using ynamide **1e** (20 mg, 54 μmol, 1.0 equiv.), with a reaction time of 15 min. The crude material was purified by column chromatography (petroleum ether / EtOAc (98:2)) to give **rac-7e** as a colourless oil (20 mg, 54 μmol, 99%).

Method B: Prepared by General Procedure G using ynamide **1e** (20 mg, 54 μmol, 1.0 equiv.) and (*S*,*R*,*R*)-**L5**, with a reaction time of 5 min. The crude material was purified by column

chromatography (petroleum ether / EtOAc (98:2)) to give ***R*)-7e** as a colourless oil (16 mg, 43 μmol, 80%).

Method C (1 mmol scale reaction): To a stirred solution of ynamide **1e** (374 mg, 1 mmol, 1.0 equiv.) in CH_2Cl_2 (5 mL) was added a solution of $[RhCl(C_2H_4)_2]_2$ (5 mg, 13 μmol, 0.0125 equiv.), $NaBAr^F_4$ (22 mg, 25 μmol, 0.025 equiv.) and phosphoramide (*S*,*R*,*R*)-**L5** (14 mg, 25 μmol, 0.025 equiv.) in CH_2Cl_2 (5 mL). The reaction mixture was stirred for 5 min, after which time water (20 mL) was added. The aqueous layer was extracted with CH_2Cl_2 (2 x 20 mL) and the combined organic extracts dried over Na_2SO_4, filtered and concentrated *in vacuo*. The crude material was purified by column chromatography (petroleum ether / EtOAc (98:2)) to give **(*R*)-7e** as a colourless oil (300 mg, 0.80 mmol, 80% yield).

$[\alpha]_D^{25}$ –24.0 (*c* = 1.0, $CHCl_3$); 99% *ee* (CHIRALPAK IA, 2% IPA / hexane, 1.3 mL/min, t_R minor – 6.89 min, major – 7.42 min); $\mathbf{R_f}$ 0.40 (petroleum ether / EtOAc (9:1)); **IR** (thin film, ν_{max} / cm^{-1}) 2954, 2927, 2856, 1719, 1456, 1350, 1161, 1089; **^{1}H NMR** (500 MHz, C_6D_6) δ_H 7.76 (2H, d, *J* = 8.0 Hz, Ts*H*), 6.74 (2H, d, *J* = 8.0 Hz, Ts*H*), 5.41-5.37 (1H, m, H5), 4.87 (1H, d, *J* = 11.0 Hz, H4), 3.49 (1H, ddd, *J* = 12.5, 7.5 and 4.5 Hz, H2), 3.19 (1H, m, H3a), 3.14 (1H, ddd, *J* = 12.5, 8.5 and 7.0 Hz, H2), 3.01-2.95 (1H, m, H9), 2.70 (1H, m, H9), 2.52-2.45 (1H, m, H7), 2.09-2.01 (3H, m, H7 and 6), 1.87 (3H, s, TsCH_3), 1.76-1.59 (2H, m, H10), 1.48-1.30 (6H, m, H11, 12 and 13), 1.17-1.11 (1H, m, H3), 0.92 (3H, t, *J* = 7.0 Hz, H14), 0.59-0.52 (1H, m, H3); **^{13}C NMR** (125 MHz, C_6D_6) δ_C 143.1, 137.8, 137.6, 134.8, 131.1, 130.0, 129.5, 128.5, 49.8, 39.7, 35.9, 32.4, 31.5, 30.1, 30.0, 28.0, 27.0, 23.2, 21.2, 14.5; **HRMS** (ESI+) calc. for $C_{22}H_{31}NNaO_2S$ $[M+Na]^+$ 396.1968, found 396.1956.

(*R*)-8-(3-chloropropyl)-1-tosyl-1,2,3,3a,6,7-hexahydrocyclohepta[*b*]pyrrole, 7f

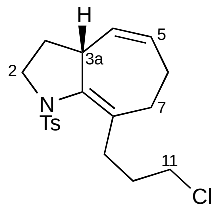

Method A: Prepared by General Procedure F using ynamide **1f** (20 mg, 55 μmol, 1.0 equiv.), with a reaction time of 15 min. The crude material was purified by column chromatography (petroleum ether / EtOAc (95:5)) to give **rac-7f** as a colourless oil (19 mg, 51 μmol, 93%).

Method B: Prepared by General Procedure G using ynamide **1f** (20 mg, 55 μmol, 1.0 equiv.) and (*S*,*R*,*R*)-**L5**, with a reaction time of 15 min. The crude material was purified by column chromatography (petroleum ether / EtOAc (95:5)) to give **(*R*)-7f** as a colourless oil (17 mg, 47 μmol, 85%).

$[\alpha]_D^{25}$ –30.3 (c = 1.0, $CHCl_3$); 97% *ee* (CHIRALPAK IB, 1% IPA / hexane, 1.3 mL/min, t_R minor – 10.51 min, major – 11.03 min); R_f 0.27 (petroleum ether / EtOAc (9:1)); **IR** (thin film, ν_{max} / cm^{-1}) 2956, 2893, 1652, 1445, 1349, 1163, 1090; **^{1}H NMR** (500 MHz, C_6D_6) δ_H 7.70 (2H, d, J = 8.0 Hz, Ts*H*), 6.75 (2H, d, J = 8.0 Hz, Ts*H*), 5.35-5.31 (1H, m, H5), 4.81 (1H, dq, J = 11.0 and 2.0 Hz, H4), 3.43 (1H, ddd, J = 13.0, 7.5 and 4.5 Hz, H2), 3.39-3.29 (2H, m, H11), 3.15-3.09 (1H, m, H3a), 3.07 (1H, ddd, J = 13.0, 8.5 and 7.0 Hz, H2), 2.87-2.81 (1H, m, H9), 2.61-2.55 (1H, m, H9), 2.39-2.33 (1H, m, H7), 2.07 (4H, m, H6 and 10), 1.88 (3H, s, TsCH_3), 1.85-1.80 (1H, m, H7), 1.16 (1H, m, H3), 0.56-0.48 (1H, m, H3); **^{13}C NMR** (125 MHz, C_6D_6) δ_C 143.3, 138.7, 137.4, 132.9, 130.9, 129.9, 129.5, 128.4, 49.7, 45.3, 39.8, 33.3, 31.4, 31.2, 29.8, 26.7, 21.2; **HRMS** (ESI+) calc. for $C_{19}H_{24}ClNNaO_2S$ $[M+Na]^+$ 388.1108, found 388.1093.

(*R*)-8-(3-((*tert*-butyldimethylsilyl)oxy)propyl)-1-tosyl-1,2,3,3a,6,7-hexahydrocyclohepta[*b*]pyrrole, 7g and 3-(1-tosyl-1,2,3,3a,6,7-hexahydrocyclohepta[b]pyrrol-8-yl)propan-1-ol, 7g′

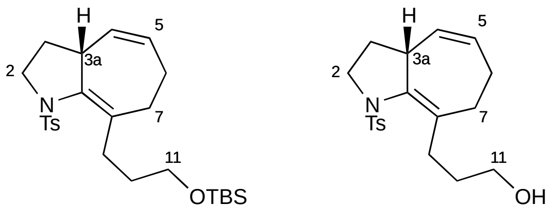

7g **7g'**

Method A: Prepared by General Procedure F using ynamide **1g** (20 mg, 43 µmol, 1.0 equiv.), with a reaction time of 15 min. The crude material was purified by column chromatography (petroleum ether / EtOAc (95:5)) to give **rac-7g** (12 mg, 26 µmol, 60%) and **rac-7g′** (5 mg, 14 µmol, 35%).

Method B: Prepared by General Procedure G using ynamide **1g** (20 mg, 43 µmol, 1.0 equiv.) and (*S*,*R*,*R*)-**L5**, with a reaction time of 15 min. The crude material was purified by column chromatography (petroleum ether / EtOAc (95:5)) to give **(*R*)-7g** as a colourless oil (19 mg, 41 µmol, 96%).

For ***(R)*-7g**: $[\alpha]_D^{25}$ –24.1 (c = 1.0, $CHCl_3$); 96% *ee* (CHIRALPAK IB, 1% IPA / hexane, 1.3 mL/min, t_R minor – 5.65 min, major – 6.08 min); R_f 0.40 (petroleum ether / EtOAc (9:1)); **IR** (thin film, ν_{max} / cm^{-1}) 2953, 2929, 2893, 2856, 1353, 1254, 1164, 1092; **^{1}H NMR** (500 MHz, C_6D_6) δ_H 7.73 (2H, d, J = 8.5 Hz, Ts*H*), 6.73 (2H, d, J = 8.5 Hz, Ts*H*), 5.37-5.33 (1H, m, H5), 4.85 (1H, dq, J = 11.0 and 2.0 Hz, H4), 3.79-3.71 (2H, m, H11), 3.47 (1H, ddd, J = 13.0, 7.5 and 4.5 Hz, H2), 3.20-3.14 (1H, m, H3a), 3.12 (1H, ddd, J = 13.0, 8.5 and 7.0 Hz, H2), 3.02-2.96 (1H, m, H9), 2.73-2.67 (1H, m, H9), 2.51-2.44 (1H, m, H7), 2.09-1.94 (5H, m, H6, 7 and 10), 1.88 (3H, s, TsCH_3), 1.16 (1H, m, H3), 1.03 (9H, s, SiC(CH_3)$_3$), 0.58-0.52 (1H, m, H3), 0.12 (6H, s, Si(CH_3)$_2$); **^{13}C NMR** (125 MHz, C_6D_6) δ_C 143.1, 138.1, 137.6, 134.3, 131.0, 130.0, 129.4, 128.5, 63.9, 49.8, 39.8, 32.3, 31.5, 31.4,

30.0, 26.8, 26.3, 21.2, 18.6, −5.0, −5.1; **HRMS** (ESI+) calc. for $C_{25}H_{39}NNaO_3SSi$ $[M+Na]^+$ 484.2312, found 484.2299.

For **7g′**: **R*f*** 0.20 (petroleum ether / EtOAc (8:2)); **IR** (thin film, ν_{max} / cm^{-1}) 2961, 2925, 1597, 1452, 1348, 1161, 1089, 1057; **^{1}H NMR** (500 MHz, C_6D_6) δ_H 7.74 (2H, d, *J* = 8.0 Hz, Ts*H*), 6.74 (2H, d, *J* = 8.0 Hz, Ts*H*), 5.37-5.33 (1H, m, H5), 4.84 (1H, d, *J* = 11.0 Hz, H4), 3.58 (2H, t, *J* = 6.5 Hz, H11), 3.47-3.42 (1H, m, H2), 3.20-3.14 (1H, m, H3a), 3.11-3.05 (1H, m, H2), 2.95-2.89 (1H, m, H9), 2.66-2.60 (1H, m, H9), 2.45-2.40 (1H, m, H7), 2.02-1.98 (2H, m, H6), 1.96-1.92 (1H, m, H7), 1.87 (3H, s, TsCH_3), 1.85-1.75 (2H, m, H10), 1.22 (1H, s, O*H*), 1.19-1.13 (1H, m, H3), 0.61-0.54 (1H, m, H3); **^{13}C NMR** (125 MHz, C_6D_6) δ_C 141.8, 136.7, 136.1, 132.8, 129.6, 128.6, 128.1, 127.1, 61.5, 48.4, 38.4, 30.6, 30.1, 29.6, 28.6, 25.4, 19.8; **HRMS** (ESI+) calc. for $C_{19}H_{25}NNaO_3S$ $[M+Na]^+$ 370.1447, found 370.1435.

1-tosyl-8-(1-tosyl-1*H*-indol-3-yl)-1,2,3,3a,6,7-hexahydrocyclohepta[*b*]pyrrole, 7h

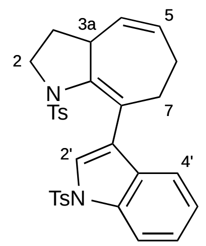

Prepared by General Procedure F using ynamide **1h** (20 mg, 36 μmol, 1.0 equiv.), with a reaction time of 15 min. The crude material was purified by column chromatography (petroleum ether / EtOAc (9:1)) to give **7h** as a colourless oil (19 mg, 33 μmol, 93%); **R*f*** 0.12 (petroleum ether / EtOAc (9:1)); **IR** (thin film, ν_{max} / cm^{-1}) 3011, 2952, 2889, 2280, 1597, 1447, 1358, 1167; **^{1}H NMR** (500 MHz, C_6D_6) δ_H 8.19 (1H, d, *J* = 8.0 Hz, H4′), 7.90 (1H, s, H2′), 7.82 (2H, d, *J* = 8.5 Hz, Ts*H*), 7.42 (1H, d, *J* = 8.0 Hz, H7′), 7.27 (2H, d, *J* = 8.5 Hz, Ts*H*), 7.13 (1H, dd, *J* = 8.0 and 7.5 Hz, H6′), 7.06 (1H, dd, *J* = 8.0 and 7.5 Hz, H5′), 6.61-6.59 (4H, m, Ts*H*), 5.47-5.42 (1H, m, H5), 5.06 (1H, dq, *J* = 11.5 and 2.0 Hz, H4), 3.53-3.47 (1H, m, H3a), 3.37-3.32 (1H, m, H2), 3.24-3.19 (1H, m, H2), 2.83-2.77 (1H, m, H7), 2.24-2.19 (1H, m, H7), 2.15-2.07 (1H, m, H6), 2.04-1.96 (1H, m, H6), 1.90 (3H, s, TsCH_3), 1.57-1.50 (4H, m, H3 and TsCH_3), 1.01-0.94 (1H, m, H3); **^{13}C NMR** (125 MHz, C_6D_6) δ_C 144.3, 142.9, 141.7, 137.4, 136.3, 135.6, 130.7, 130.5, 130.3, 129.9, 129.2, 127.7, 127.1, 125.5, 125.0, 124.5, 123.5, 123.2, 121.6, 114.1, 49.7, 40.6, 33.2, 32.2, 26.6, 21.2, 20.9; **HRMS** (ESI+) calc. for $C_{31}H_{30}N_2NaO_4S_2$ $[M+Na]^+$ 581.1539, found 581.1519.

1-tosyl-8-(1-tosyl-1*H*-pyrrol-2-yl)-1,2,3,3a,6,7-hexahydrocyclohepta[*b*]pyrrole, 7i

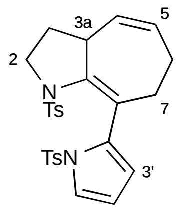

Prepared by General Procedure F using ynamide **1i** (20 mg, 39 μmol, 1.0 equiv.), with a reaction time of 6 h. The crude material was purified by column chromatography (petroleum ether / Et_2O (5:2)) to give **7i** as a colourless oil (17 mg, 34 μmol, 86%); **R*f*** 0.28 (petroleum ether / Et_2O) (5:2)); **IR** (thin film, ν_{max} / cm^{-1}) 2955, 2919, 2893, 1597, 1357, 1332, 1166, 1150, 1091; **^{1}H NMR** (500 MHz, C_6D_6) δ_H 7.95-7.75 (4H, m, Ts*H*), 7.10-6.99 (1H, m, H5′), 6.79 (2H, d, *J* = 8.0 Hz, Ts*H*), 6.63 (2H, d, *J* = 8.0 Hz, Ts*H*), 6.36-6.33 (1H, m, H3′), 6.06 (1H, t, *J* = 3.5 Hz, H4′), 5.53-5.34 (1H, m, H5), 5.11-4.96 (1H, m, H4), 3.62-3.38 (3H, m, H2 and H3a), 3.09-2.93 (1H, m, H7), 2.50-2.34 (1H, m, H7), 2.26-2.08 (1H, m, H6), 1.93-1.82 (4H, m, H6 and TsCH_3), 1.76 (3H, s, TsCH_3), 1.26-1.14 (1H, m, H3), 1.08-0.94 (1H, m, H3); **^{13}C NMR** (125 MHz, C_6D_6) δ_C 143.1, 142.6, 141.6, 137.2, 136.6, 129.8, 129.0, 128.5, 128.3, 126.8, 121.4, 112.7, 111.4, 49.3, 39.8, 32.9, 30.7, 29.0, 25.1, 19.7, 19.9; **HRMS** (ESI+) calc. for $C_{27}H_{28}O_4N_2NaS_2$ $[M+Na]^+$ 531.1383, found 531.1382.

6-methyl-5-tosyl-5,7,8,10a-tetrahydrocyclohepta[*b*]indole, 7j

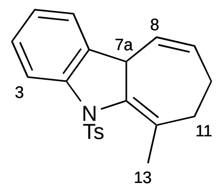

Prepared by General Procedure F using ynamide **1j** (20 mg, 57 μmol, 1.0 equiv.), with a reaction time of 1 h. The crude material was purified by column chromatography (petroleum ether / EtOAc (95:5)) to give **7j** as a colourless oil (15 mg, 42 μmol, 74%); **R*f*** 0.34 (petroleum ether / EtOAc (9:1)); **IR** (thin film, ν_{max} / cm^{-1}) 3032, 2910, 1656, 1493, 1358, 1170; **^{1}H NMR** (500 MHz, C_6D_6) δ_H 7.98 (1H, d, *J* = 8.0 Hz, H6), 7.40 (2H, d, *J* = 8.5 Hz, Ts*H*), 7.00 (1H, t, *J* = 8.0 and 7.5 Hz, H5), 6.85 (1H, t, *J* = 7.5 Hz, H4), 6.67 (1H, d, *J* = 7.5 Hz, H3), 6.50 (2H, d, *J* = 8.5 Hz, Ts*H*), 5.53-5.48 (1H, m, H9), 5.34 (1H, d, *J* = 11.0 Hz, H8), 3.90-3.85 (1H, m, H7a), 2.35-2.28 (4H, m, H11 and 13), 2.09-2.02 (1H, m, H10), 1.87-1.80 (2H, m, H10 and 11), 1.70 (3H, s, TsCH_3); **^{13}C NMR** (125 MHz, C_6D_6) δ_C 143.6, 143.4, 138.0, 136.6, 135.0, 133.0, 131.7, 129.7, 129.0, 128.6, 127.7, 126.4, 123.1, 121.4, 43.1, 33.1, 25.6, 23.1, 21.1; **HRMS** (ESI+) calc. for $C_{21}H_{22}O_2NS$ $[M+H]^+$ 352.1366, found 352.1360.

(*R*)-1-((4-nitrophenyl)sulfonyl)-8-phenyl-1,2,3,3a,6,7-hexahydrocyclohepta[b]pyrrole, 7k

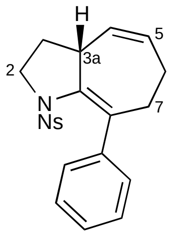

Method A: Prepared by General Procedure F using ynamide **1k** (20 mg, 54 µmol, 1.0 equiv.), with a reaction time of 1 h. The crude material was purified by column chromatography (petroleum ether / EtOAc (95:5)) to give **rac-7k** as a yellow oil (20 mg, 54 µmol, >99%).

Method B: Prepared by General Procedure G using ynamide **1k** (20 mg, 54 µmol, 1.0 equiv.) and (*S*,*R*,*R*)-**L5**, with a reaction time of 10 min. The crude material was purified by column chromatography (petroleum ether / EtOAc (95:5)) to give **(*R*)-7k** as a yellow oil (20 mg, 54 µmol, >99%).

$[\alpha]_D^{25}$ –149.0 (*c* = 1.0, $CHCl_3$); 96% *ee* (CHIRALPAK IB, 10% IPA / hexane, 1.3 mL/min, t_R minor – 12.5 min, major – 14.5 min); $\mathbf{R_f}$ (petroleum ether / EtOAc (9:1)); **IR** (thin film, ν_{max} / cm^{-1}) 3104, 2953, 1605, 1523, 1349, 1309, 1169, 1107; **^{1}H NMR** (500 MHz, C_6D_6) δ_H 7.51 (2H, d, *J* = 9.0 Hz, Ns*H*), 7.18-7.12 (4H, m, Ns*H* and Ph*H*), 6.99-6.96 (2H, m, Ph*H*), 6.93-6.89 (1H, m, Ph*H*), 5.38-5.34 (1H, m, H5), 4.97 (1H, d, *J* = 11.0 Hz, H4), 3.47-3.41 (1H, m, H3a), 3.34 (1H, ddd, *J* = 12.0, 7.0 and 5.5, H2), 3.12 (1H, ddd, *J* = 12.0, 7.5 and 7.0, H2), 2.79-2.73 (1H, m, H7), 2.21-2.16 (1H, m, H7), 2.12-2.03 (1H, m, H6), 2.02-1.94 (1H, m, H6), 1.61-1.54 (1H, m, H3), 1.00-0.94 (1H, m, H3); **^{13}C NMR** (125 MHz, C_6D_6) δ_C 148.5, 144.6, 141.5, 137.8, 131.2, 129.3, 129.2, 127.5, 127.2, 127.1, 125.6, 122.3, 48.7, 38.9, 31.9, 31.7, 25.4
; **HRMS** (ESI+) calc. for $C_{21}H_{20}O_4N_2NaS$ $[M+Na]^+$ 419.1036, found 419.1030.

(3*R*,3a*S*)-8-hexyl-3-phenyl-1-tosyl-1,2,3,3a,6,7-hexahydrocyclohepta[*b*]pyrrole, 7l

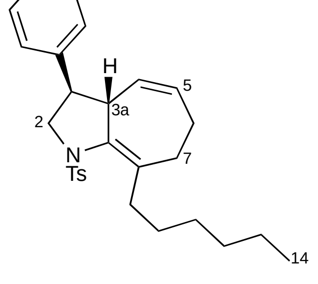

Method A: Prepared by General Procedure F using ynamide **1l** (20 mg, 44 µmol, 1.0 equiv.), with a reaction time of 16 h. The crude material was purified by column chromatography (petroleum ether / EtOAc (9:1)) to give **7l** as a colourless oil (17 mg, 38 µmol, 86%)

Method B: Prepared by General Procedure G using ynamide **1l** (20 mg, 44 µmol, 1.0 equiv.) and (*S*,*R*,*R*)-**L5**, with a reaction time of 15 min. The crude material was purified by column

chromatography (petroleum ether / EtOAc (9:1)) to give **7l** as a colourless oil (20 mg, 44 μmol, >99%)

$[\alpha]_D^{25}$ –44.9 (*c* = 1.0, $CHCl_3$); $\mathbf{R_f}$ 0.40 (petroleum ether / EtOAc (9:1)); **IR** (thin film, ν_{max} / cm^{-1}) 3028, 2954, 2925, 2855, 1598, 1496, 1353, 1163; **^{1}H NMR** (500 MHz, C_6D_6) δ_H 7.83 (2H, d, *J* = 8.0 Hz, Ts*H*), 7.02-6.98 (3H, m, Ph*H* and Ts*H*), 6.79 (2H, d, *J* = 8.0 Hz, Ts*H*), 6.67-6.65 (2H, m, Ph*H*), 5.42-5.37 (1H, m, H5), 4.98 (1H, d, *J* = 11.0 Hz, H4), 4.01 (1H, dd, *J* = 12.5 and 7.5 Hz, H2), 3.42 (1H, br d, *J* = 10.5 Hz, H3a), 3.17 (1H, t, *J* = 12.5 Hz, H2), 3.13-3.07 (1H, m, H7), 2.68-2.62 (1H, m, H7), 2.49-2.43 (1H, m, H9), 2.20-2.06 (4H, m, H3, 9 and 10), 1.90 (3H, s, TsCH_3), 1.89-1.81 (1H, m, H6), 1.63-1.33 (7H, m, H6 and H11-13), 0.94 (3H, s, H14); **^{13}C NMR** (125 MHz, C_6D_6) δ_C 142.13, 138.6, 136.0, 135.9, 134.1, 129.1, 128.3, 127.7, 127.6, 127.3, 127.1, 126.1, 55.0, 49.2, 47.2, 35.0, 31.1, 29.1, 29.0, 26.8, 25.9, 22.0, 20.0, 13.2; **HRMS** (ESI+) calc. for $C_{28}H_{35}NNaO_2S$ $[M+Na]^+$ 472.2281, found 472.2284.

(3*R*,3a*R*)-8-hexyl-3-phenyl-1-tosyl-1,2,3,3a,6,7-hexahydrocyclohepta[*b*]pyrrole, 14l

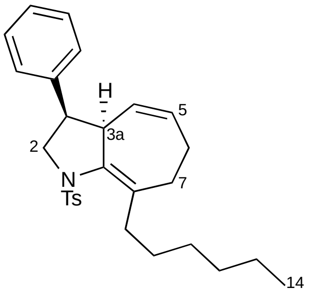

Prepared by General Procedure G using ynamide **1l** (20 mg, 44 μmol, 1.0 equiv.) and (*R*,*S*,*S*)-**L5**, with a reaction time of 3 h. The crude material was purified by column chromatography (petroleum ether / EtOAc (95:5)) to give **14l** as a colourless oil (17 mg, 38 μmol, 83%); $[\alpha]_D^{25}$ –48.9 (*c* = 1.0, $CHCl_3$); $\mathbf{R_f}$ 0.40 (petroleum ether / EtOAc (9:1)); **IR** (thin film, ν_{max} / cm^{-1}) 2926, 2856, 1456, 1354, 1165, 1090; **^{1}H NMR** (500 MHz, C_6D_6) δ_H 7.83 (2H, d, *J* = 8.0 Hz, Ts*H*), 7.07 (2H, t, *J* = 7.5 Hz, Ph*H*), 7.00 (1H, t, *J* = 7.5 Hz, Ph*H*), 6.83 (2H, d, *J* = 7.5 Hz, Ph*H*), 6.79 (2H, d, *J* = 8.0 Hz, Ts*H*), 5.43-5.38 (1H, m, H5), 4.98 (1H, d, *J* = 11.5 Hz, H4), 3.91 (1H, dd, *J* = 12.0 and 7.5 Hz, H2), 3.53 (1H, dd, *J* = 12.0 and 6.0 Hz, H2), 3.16-3.10 (2H, m, H3a and 9), 2.76-2.69 (2H, m, H3 and 9), 2.37-2.31 (1H, m, H7), 2.11-2.03 (2H, m, H6 and 7), 1.88 (3H, s, TsCH_3), 1.84-1.73 (2H, m, H6 and 10), 1.53-1.32 (7H, m, H10 and 11-13), 0.94 (3H, t, *J* = 7.0 Hz, H14); **^{13}C NMR** (125 MHz, C_6D_6) δ_C 143.4, 140.7, 136.7, 136.6, 135.1, 131.6, 129.6, 128.9, 128.5, 128.4, 127.6, 127.0, 54.8, 45.8, 45.5, 37.0, 32.5, 30.8, 30.3, 28.0, 26.8, 23.2, 21.2, 14.5; **HRMS** (ESI+) calc. for $C_{28}H_{35}O_2NNaS$ $[M+Na]^+$ 472.2281, found 472.2268.

(3*R,3a*S**)-3,8-diphenyl-1-tosyl-1,2,3,3a,6,7-hexahydrocyclohepta[*b*]pyrrole, 7m**

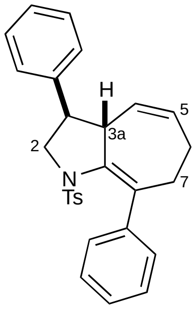

Prepared by General Procedure F using ynamide **1m** (20 mg, 45 µmol, 1.0 equiv.), with a reaction time of 16 h. The crude material was purified by column chromatography (petroleum ether / EtOAc (9:1)) to give **7m** as a colourless oil (16 mg, 36 µmol, 79%); **R*f*** 0.23 (petroleum ether / EtOAc (9:1)); **IR** (thin film, ν_{max} / cm^{-1}) 3027, 2970, 2947, 1739, 1598, 1493, 1360, 1165, 1074; **^{1}H NMR** (500 MHz, C_6D_6) δ_H 7.57 (2H, d, *J* = 8.0 Hz, Ph*H*), 7.50 (2H, d, *J* = 8.0 Hz, wTs*H*), 7.26 (2H, t, *J* = 8.0 Hz, Ph*H*), 7.13 (1H, t, *J* = 8.0 Hz, Ph*H*), 7.09-7.03 (3H, m, Ph*H*), 6.85 (2H, d, *J* = 7.5 Hz, Ph*H*), 6.71 (2H, d, *J* 8.0 Hz, Ts*H*), 5.51-5.47 (1H, m, H5), 5.19 (1H, d, *J* = 11.0 Hz, H4), 4.00 (1H, dd, *J* = 12.0 and 7.5 Hz, H2), 3.67 (1H, br d, *J* = 11.0 Hz, H3a), 3.18 (1H, t, *J* = 12.0, H2), 2.83 (1H, t, *J* = 13.5 Hz, H7), 2.66 (1H, td, *J* = 11.0 and 7.5 Hz, H3), 2.43-2.33 (2H, m, H6 and 7), 2.16-2.11 (1H, m, H6), 1.88 (3H, s, TsC*H*$_3$); **^{13}C NMR** (125 MHz, C_6D_6) δ_C 143.8, 142.9, 139.9, 138.6, 137.7, 132.5, 130.5, 129.3, 129.0, 128.8, 128.4, 128.3, 128.1, 128.0, 127.4, 126.6, 56.0, 51.4, 49.4, 33.4, 27.4, 21.2; **HRMS** (ESI+) calc. for $C_{28}H_{27}NNaO_2S$ $[M+Na]^+$ 464.1655, found 464.1660.

(3*S,3a*S**)-3-(2-((4-methoxybenzyl)oxy)ethyl)-8-phenyl-1-tosyl-1,2,3,3a,6,7-hexahydrocyclohepta[*b*]pyrrole, 7n**

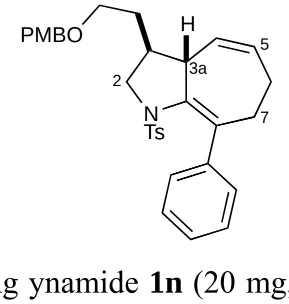

Prepared by General Procedure F using ynamide **1n** (20 mg, 38 µmol, 1.0 equiv.), with a reaction time of 20 h. The crude material was purified by column chromatography (petroleum ether / EtOAc (9:1)) to give **7n** as a colourless oil (14 mg, 26 µmol, 70%); **R*f*** 0.40 (petroleum ether / EtOAc (4:1)); **IR** (thin film, ν_{max} / cm^{-1}) 3057, 2917, 2857, 1612, 1513, 1356, 1248, 1162, 1090, 1033; **^{1}H NMR** (500 MHz, C_6D_6) δ_H 7.55-7.52 (4H, m, Ts*H* and Ph*H*), 7.25-7.21 (4H, m, PMB*H* and Ph*H*), 7.10 (1H, t, *J* = 7.5 Hz, Ph*H*), 6.86 (2H, d, *J* = 8.5 Hz, PMB*H*), 6.69 (2H, d, *J* = 8.0 Hz, Ts*H*), 5.48-5.43 (1H, m, H5), 5.06 (1H, app. dq, *J* = 11.5 and 2.0 Hz, H4), 4.24 (1H, d, *J* = 11.5 Hz, PMBC*H*$_2$), 4.20 (1H, d, *J* = 11.5 Hz, PMBC*H*$_2$), 4.08 (1H, dd, *J* = 12.5 and 7.0 Hz, H2), 3.33 (3H, s, PMBC*H*$_3$),

3.16-3.07 (3H, m, H3a and H9), 2.82 (1H, dd, J = 12.5 and 11.0 Hz, H2) 2.81 (1H, ddd, J = 15.0, 13.5 and 2.0 Hz, H7), 2.41-2.32 (2H, m, H6 and H7), 2.16-2.09 (1H, m, H6), 1.87 (3H, s, TsCH_3), 1.63-1.57 (1H, m, H3), 1.51-1.44 (1H, m, H8), 1.30-1.23 (1H, m, H8); **^{13}C NMR** (125 MHz, C_6D_6) δ_C 158.6, 142.6, 141.5, 138.0, 136.7, 130.4, 129.8, 128.8, 128.3, 128.2, 128.0, 127.4, 127.4, 127.1, 125.3, 113.0, 71.7, 67.3, 54.0, 53.7, 46.3, 42.1, 32.0, 31.6, 26.3, 20.0; **HRMS** (ESI+) calc. for $C_{32}H_{36}O_4NS$ $[M+H]^+$ 530.2360, found 530.2355. 26

(2*S,3a*R**)-2-methyl-8-phenyl-1-tosyl-1,2,3,3a,6,7-hexahydrocyclohepta[*b*]pyrrole, 7o and (2*S**,3a*S**)-2-methyl-8-phenyl-1-tosyl-1,2,3,3a,6,7-hexahydrocyclohepta[*b*]pyrrole, 14o**

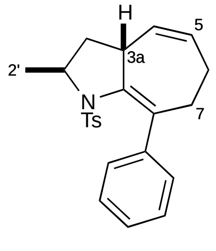

7o

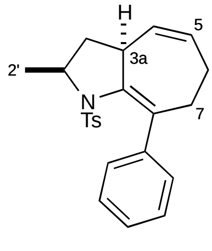

14o

Prepared by General Procedure F using ynamide **1o** (20 mg, 53 µmol, 1.0 equiv.), with a reaction time of 2 h. The crude material was purified by column chromatography (petroleum ether / EtOAc (95:5)) to give **7o** and **14o** as an inseparable mixture in a 2:1 ratio as a colourless oil (18 mg, 48 µmol, 90%).

For **7o**: **R*f*** 0.35 (petroleum ether / EtOAc (9:1)); **IR** (thin film, ν_{max} / cm^{-1}) 3018, 2958, 1651, 1494, 1444, 1355, 1164, 1087, 1035; **^{1}H NMR** (500 MHz, C_6D_6) δ_H 7.59-7.56 (4H, m, Ts*H* and Ph*H*), 7.27 (2H, t, J = 7.5 Hz, Ph*H*), 7.11 (1 H, t, J = 7.5, Ph*H*), 6.7 (2H, d, J = 8.0 Hz, Ts*H*), 5.45 (1H, m, H5), 4.91 (1H, d, J = 11.5 Hz, H4), 4.16 (1H, quin, J = 7.0 Hz, H2), 3.66-3.59 (1H, m, H3a), 2.90-2.84 (1H, m, H7), 2.46-2.38 (2H, m, H6), 2.16-2.09 (1H, m, H6), 1.88 (3H, s, TsCH_3), 1.23-1.19 (1H, m, H3β), 1.00-0.94 (4H, m, H2′ and 3α); **^{13}C NMR** (125 MHz, C_6D_6) δ_C 143.8, 142.6, 138.2, 137.8, 132.5, 130.0, 129.6, 129.1, 128.4, 128.4, 128.3, 126.6, 57.0, 39.5, 37.8, 33.1, 27.7, 21.1, 20.5; **HRMS** (ESI+) calc. for $C_{23}H_{25}NNaO_2S$ $[M+Na]^+$ 402.1498, found 402.1491.

For **14o**: **R*f*** 0.41 (petroleum ether / EtOAc (9:1)); **IR** (thin film, ν_{max} / cm^{-1}) 3019, 2962, 1652, 1492, 1451, 1355, 1165, 1087, 1032; **^{1}H NMR** (500 MHz, C_6D_6) δ_H 7.51 (2H, d, J = 8.0 Hz, Ph*H*), 7.45 (2H, d, J = 8.5 Hz, Ts*H*), 7.25 (2H, t, J = 8.0 Hz, Ph*H*), 7.11 (1H, t, J = 8.0 Hz, Ph*H*), 6.68 (2H, d, J = 8.5 Hz, Ts*H*), 5.47-5.42 (1H, m, H5), 5.21 (1H, d, J = 11.5 Hz, H4), 4.06 (1H, quin d, J = 7.0 and 2.5 Hz, H2), 3.54-3.48 (1H, m, H3a), 2.95-2.89 (1H, m, H7), 2.39-2.34 (1H, m, H7), 2.03-1.98 (2H, m, H6), 1.86 (3H, s, TsCH_3), 1.84-1.78 (1H, m, H3α), 1.09 (3H, d, J = 7.0 Hz, H2′), 0.92-0.88 (1H, m, H3β); **^{13}C NMR** (125 MHz, C_6D_6) δ_C 143.4, 142.7, 138.3, 137.9, 134.8, 132.8, 130.1, 129.6,

129.2, 128.3, 127.9, 126.5, 57.2, 40.5, 38.8, 33.9, 26.0, 21.6, 21.1; **HRMS** (ESI+) calc. for $C_{23}H_{25}NNaO_2S$ $[M+Na]^+$ 402.1498, found 402.1490.

(2*R*,3a*R*)-2-(((4-methoxybenzyl)oxy)methyl)-8-methyl-1-tosyl-1,2,3,3a,6,7-hexahydrocyclohepta[*b*]pyrrole, 7p and (2*R*,3a*S*)-2-(((4-methoxybenzyl)oxy)methyl)-8-methyl-1-tosyl-1,2,3,3a,6,7-hexahydrocyclohepta[*b*]pyrrole, 14p

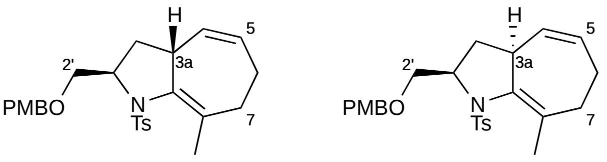

7p **14p**

Prepared by General Procedure F using ynamide **1p** (20 mg, 44 µmol, 1.0 equiv.), with a reaction time of 1 h. The crude material was purified by column chromatography (petroleum ether / EtOAc (9:1)) to give **7p** and **14p** as an inseparable mixture in a 1.8:1 ratio as a colourless oil (18 mg, 40 µmol, 91%); **R*f*** 0.30 (petroleum ether / EtOAc (8:2)); **IR** (thin film, ν_{max} / cm^{-1}) 3007, 2934, 2905, 2853, 1613, 1513, 1351, 1247, 1165, 1091; **^{1}H NMR** (500 MHz, C_6D_6) δ_H 7.77-7.74 (4H, m, Ts*H* of 7p and 14p), 7.19-7.16 (4H, m, PMB*H* of 7p and 14p), 6.80-6.72 (8H, m, Ts*H* and PMB*H* of 7p and 14p), 5.53-5.48 (1H, m, H5 of 14p), 5.30-5.25 (1H, m, H5 of 7p), 5.16 (1H, d, *J* = 11.0 Hz, H4 of 14p), 4.75 (1H, d, *J* = 11.0 Hz, H4 of 7p), 4.39-4.19 (6H, m, PMBCH_2 and H1 of 7p and 14p), 3.86 (1H, dd, *J* = 9.0 and 5.0 Hz, H2′ of 14p), 3.59-3.53 (1H, m, H3a of 7p), 3.49 (1H, dd, *J* = 9.5 and 5.0 Hz, H2′ of 7p), 3.34 (1H, dd, *J* = 9.0 and 9.0 Hz, H2′ of 14p), 3.30 (3H, s, PMBCH_3 of 7p), 3.29 (3H, s, PMBCH_3 of 14p), 3.21 (1H, dd, *J* = 9.5 and 7.5 Hz, H2′ of 7p), 2.82-2.76 (1H, m, H3a of 14p), 2.60-2.54 (1H, m, H7 of 7p), 2.32-2.26 (4H, m, H9 of 7p, and H7 of 14p), 2.21-2.05 (3H, d, *J* = 2.0 Hz, H9 of 14p), 2.13-2.05 (2H, m, H6 of 7p and 14p), 1.97-1.82 (8H, m, H6 and TsCH_3 of 7p, and H6, H7 and TsCH_3 of 14p), 1.79 (1H, dd, *J* = 12.5 and 9.0 Hz, H3 of 7p), 1.76-1.72 (1H, m, H7 of 7p), 1.64-1.58 (1H, m, H3 of 14p), 1.40-1.35 (1H, m, H3 of 14p), 0.65-0.59 (1H, m, H3 of 7p); **^{13}C NMR** (125 MHz, C_6D_6) δ_C 159.4, 159.4, 142.9, 142.7, 142.7, 137.8, 136.8, 136.3, 135.1, 131.9, 131.9, 130.6, 130.6, 130.4, 130.4, 129.5, 129.2, 129.0, 128.9, 128.8, 128.4, 128.0, 113.7, 113.7, 72.8, 72.8, 72.7, 71.2, 60.7, 59.1, 54.4, 54.4, 39.6, 38.6, 34.6, 33.1, 32.9, 32.3, 26.1, 25.5, 23.4, 22.8, 20.8, 20.8; **HRMS** (ESI+) calc. for $C_{26}H_{31}O_4NNaS$ $[M+Na]^+$ 476.1866, found 476.1854.

(2*R*,3a*R*)-2-(((*tert*-butyldimethylsilyl)oxy)methyl)-8-methyl-1-tosyl-1,2,3,3a,6,7-hexahydrocyclohepta[*b*]pyrrole, 7q

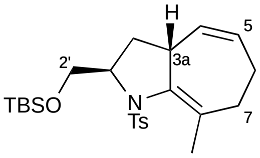

Method A: Prepared by General Procedure F using ynamide **1q** (20 mg, 45 μmol, 1.0 equiv.), with a reaction time of 1 h. The crude material was purified by column chromatography (petroleum ether / EtOAc (98:2)) to give **7q** and **14q** (see data below) in a 1.9:1 ratio as a colourless oil (15 mg, 34 μmol, 76%).

Method B: Prepared by General Procedure G using ynamide **1q** (15 mg, 34 μmol, 1.0 equiv.) and (*S*,*R*,*R*)-**L5**, with a reaction time of 1 h. The crude material was purified by column chromatography (petroleum ether / Et_2O (98:2)) to give **7q** as a colourless oil (14 mg, 32 μmol, 93%).

$[\alpha]_D^{25}$ –78.8 (*c* = 1.0, $CHCl_3$); $\mathbf{R_f}$ 0.37 (petroleum ether / Et_2O (9:1)); **IR** (thin film, ν_{max} / cm^{-1}) 2929, 2903, 2856, 1354, 1166, 1116, 1093; ^{1}**H NMR** (500 MHz, C_6D_6) δ_H 7.76 (2H, d, *J* = 8.0 Hz, Ts*H*), 6.75 (2H, d, *J* = 8.0 Hz, Ts*H*), 5.31-5.27 (1H, m, H5), 4.79 (1H, d, *J* = 11.0 Hz, H4), 4.24 (1H, td, *J* = 7.5 and 5.0 Hz, H2), 3.69-3.63 (2H, m, H2′ and H3a), 3.44 (1H, dd, *J* = 10.0 and 7.5 Hz, H2′), 2.62 (1H, td, *J* = 14.0 and 3.0 Hz, H7), 2.31 (3H, d, *J* = 1.5 Hz, H9), 2.13-2.05 (1H, m, H6), 2.00-1.93 (1H, m, H6), 1.90 (3H, s, TsCH_3), 1.87-1.83 (1H, m, H3β), 1.79-1.74 (1H, m, H7), 0.91 (9H, s, SiC(CH_3)$_3$), 0.69-0.63 (1H, ddd, *J* = 12.5, 10.5 and 8.0, H3α), 0.01 (3H, s, SiCH_3), –0.02 (3H, s, SiCH_3); ^{13}**C NMR** (125 MHz, C_6D_6) δ_C 141.8, 137.3, 136.0, 130.0, 128.2, 128.1, 127.9, 127.6, 64.2, 61.7, 38.0, 31.6, 31.6, 25.3, 24.8, 22.1, 20.0, 17.2, –6.6, –6.6; **HRMS** (ESI+) calc. for $C_{24}H_{37}O_3NNaSSi$ $[M+Na]^+$ 470.2156, found 470.2153.

(2*R*,3a*S*)-2-(((*tert*-butyldimethylsilyl)oxy)methyl)-8-methyl-1-tosyl-1,2,3,3a,6,7-hexahydrocyclohepta[*b*]pyrrole, 14q

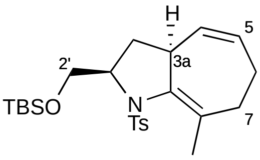

Prepared by General Procedure G using ynamide **1q** (15 mg, 34 μmol, 1.0 equiv.) and (*R*,*S*,*S*)-**L5**, with a reaction time of 7 h. The crude material was purified by column chromatography (petroleum ether / Et_2O (98:2)) to give **14q** as a colourless oil (11 mg, 25 μmol, 76%); $[\alpha]_D^{25}$ –64.9 (*c* = 1.0, $CHCl_3$); $\mathbf{R_f}$ 0.37 (petroleum ether / Et_2O (9:1)); **IR** (thin film, ν_{max} / cm^{-1}) 2953, 2930, 2856, 1598, 1471, 1355, 1255, 1167, 1119, 1092; ^{1}**H NMR** (500 MHz, C_6D_6) δ_H 7.75 (2H, d, *J* = 8.0 Hz, Ts*H*), 6.71 (2H, d, *J* = 8.0 Hz, Ts*H*), 5.56-5.51 (1H, m, H5), 5.22 (1H, d, *J* = 11.0 Hz, H4), 4.12-4.07 (1H,

m, H2), 4.04 (1H, dd, *J* = 10.0 and 5.0 Hz, H2′), 3.55 (1H, dd, *J* = 10.0 and 8.0 Hz, H2′), 2.83-2.77 (1H, m, H3a), 2.33-2.27 (1H, m, H7), 2.24 (3H, d, *J* = 2.0 Hz, H9), 2.16-2.08 (1H, m, H6), 1.94-1.87 (2H, m, H6 and H7), 1.85 (3H, s, TsC*H*3), 1.60 (1H, ddd, *J* = 12.5, 10.0 and 8.0, H3α), 1.41 (1H, ddd, *J* = 12.5, 8.0 and 5.0, H3β), 0.94 (9H, s, SiC(C*H*3)3), 0.06 (3H, s, SiC*H*3), 0.04 (3H, s, SiC*H*3); **^{13}C NMR** (125 MHz, C_6D_6) δ_C 142.1, 135.5, 134.4, 131.0, 130.9, 129.6, 128.3, 127.1, 65.2, 60.1, 38.9, 33.2, 32.3, 24.9, 24.7, 22.7, 20.0, 17.3, –6.4, –6.5; **HRMS** (ESI+) calc. for $C_{24}H_{37}O_3NNaSSi$ [M+Na]$^+$ 470.2156, found 470.2154.

(2*S*,3*R*,3a*S*)-2,8-dimethyl-3-phenyl-1-tosyl-1,2,3,3a,6,7-hexahydrocyclohepta[*b*]pyrrole, 7r

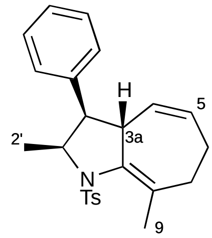

Method A: Prepared by General Procedure F using ynamide **1r** (20 mg, 51 µmol, 1.0 equiv.), with a reaction time of 5 h. The crude material was purified by column chromatography (petroleum ether / EtOAc (95:5)) to give **7r** as a colourless oil (20 mg, 51 µmol, >99%).

Method B: Prepared by General Procedure G using ynamide **1r** (20 mg, 51 µmol, 1.0 equiv.) and (*S*,*R*,*R*)-**L5**, with a reaction time of 1 h. The crude material was purified by column chromatography (petroleum ether / EtOAc (95:5)) to give **7r** as a colourless oil (20 mg, 51 µmol, >99%).

$[\alpha]_D^{25}$ –42.3 (*c* = 1.0, $CHCl_3$); **R*f*** 0.44 (petroleum ether / EtOAc (9:1)); **IR** (thin film, ν_{max} / cm^{-1}) 3062, 2977, 2850, 1598, 1451, 1349, 1165, 1088; **^{1}H NMR** (500 MHz, C_6D_6) δ_H 7.88 (2H, d, *J* = 8.0 Hz, Ts*H*), 7.05-6.97 (3H, m, Ph*H*), 6.82-6.79 (4H, m, Ph*H* and Ts*H*), 5.37-5.33 (1H, m, H5), 4.83 (1H, d, *J* = 11.0 Hz, H4), 4.35 (1H, dq, *J* = 7.0 and 7.0 Hz, H2), 3.90 (1H, d, *J* = 12 Hz, H3a), 2.71 (1H, td, *J* = 14.0 and 2.5 Hz, H7), 2.33 (3H, d, *J* = 1.5 Hz, H9), 2.31 (1H, dd, *J* = 12.0 and 7.0 Hz, H3), 2.21-2.11 (1H, m, H6), 2.03-1.96 (1H, m, H6), 1.87-1.81 (1H, m, H7), 1.86 (3H, s, TsC*H*3), 0.79 (3H, d, *J* = 7.0 Hz, C*H*3); **^{13}C NMR** (125 MHz, C_6D_6) δ_C 143.3, 137.7, 137.7, 136.3, 132.0, 130.2, 129.5, 128.8, 128.7, 128.7, 128.6, 127.1, 60.9, 52.9, 41.8, 32.8, 26.6, 23.2, 21.2, 15.7; **HRMS** (ESI+) calc. for $C_{24}H_{28}O_2NS$ [M+H]$^+$ 394.1835, found 394.1835.

(2*S*,3*R*,3a*R*)-2,8-dimethyl-3-phenyl-1-tosyl-1,2,3,3a,6,7-hexahydrocyclohepta[*b*]pyrrole, 14r

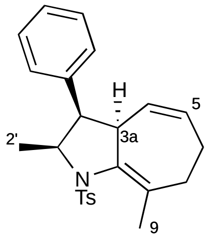

Prepared by General Procedure G using ynamide **1r** (20 mg, 51 μmol, 1.0 equiv.) and (*R*,*S*,*S*)-**L5**, with a reaction time of 3 h. The crude material was purified by column chromatography (petroleum ether / EtOAc (95:5)) to give **7r** and **14r** in a 1.2:1 ratio as a colourless oil (17 mg, 43 μmol, 86%); **R*f*** 0.44 (petroleum ether / EtOAc (9:1)); **IR** (thin film, ν_{max} / cm^{-1}) 3027, 2980, 2931, 1598, 1496, 1453, 1379, 1166, 1123; **^{1}H NMR** (500 MHz, C_6D_6) δ_H 7.88 (2H, d, *J* = 8.5 Hz, Ts*H* of 7r), 7.85 (2H, d, *J* = 8.5 Hz, Ts*H* of 14r), 7.10-7.07 (2H, m, Ph*H* of 14r), 7.05-6.97 (4H, m, Ph*H* of 7r and 14r), 6.91 (2H, d, *J* = 8.0 Hz, Ph*H* of 14r), 6.84-6.79 (6H, m, Ph*H* and Ts*H* of 7r, and Ts*H* of 14r), 5.44-5.39 (1H, m, H5 of 14r), 5.37-5.33 (1H, m, H5 of 7r), 4.91 (1H, d, *J* = 11.0 Hz, H4 of 14r), 4.83 (1H, d, *J* = 11.0 Hz, H4 of 7r), 4.35 (1H, dq, *J* = 7.0 and 7.0 Hz, H2 of 7r), 4.03 (1H, dq, *J* = 7.0 and 7.0 Hz, H2 of 14r), 3.90 (1H, d, *J* = 12.0 Hz, H3a of 7r), 2.75-2.68 (2H, m, H7 of 7r, and H3a of 14r), 2.64 (1H, dd, *J* = 7.5 and 7.0 Hz, H3 of 14r), 2.34 (3H, d, *J* = 2.0 Hz, H9 of 14r), 2.33 (3H, d, *J* = 1.5 Hz, H9 of 7r), 2.32-2.29 (1H, dd, *J* = 12.0 and 7.0 Hz, H3 of 7r), 2.24-2.11 (3H, m, H6 of 7r, and H6 and H7 of 14r), 2.07-1.96 (2H, m, H6 of 7r, and H7 of 14r), 1.88 (3H, s, TsCH_3 of 14r), 1.86 (3H, s, TsCH_3 of 7r), 1.86-1.81 (1H, m, H7 of 7r), 1.32 (3H, d, *J* = 7.0 Hz, H2′ of 14r), 0.79 (3H, d, *J* = 7.0 Hz, H2′ of 7r); **^{13}C NMR** (125 MHz, C_6D_6) δ_C 143.4, 143.3, 138.7, 137.7, 137.6, 136.3, 135.2, 134.6, 132.7, 132.0, 131.3, 130.4, 130.1, 129.5, 129.4, 129.0, 128.8, 128.7, 128.7, 128.6, 128.4, 128.4, 127.1, 127.1, 60.9, 60.1, 52.9, 52.2, 46.9, 41.8, 34.4, 32.8, 26.6, 26.1, 24.6, 23.2, 21.2, 21.2, 20.3, 15.7; **HRMS** (ESI+) calc. for $C_{24}H_{27}O_2NNaS$ [M+Na]$^+$ 416.1655, found 416.1651.

(3a*R,6*S**)-6-(((*tert*-butyldiphenylsilyl)oxy)methyl)-8-hexyl-1-tosyl-1,2,3,3a,6,7-hexahydrocyclohepta[*b*]pyrrole, 7s**

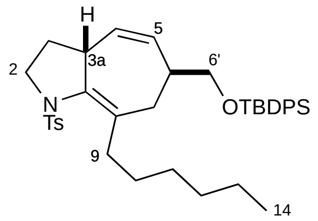

Prepared by General Procedure F using ynamide **1s** (20 mg, 31 μmol, 1.0 equiv.), with a reaction time of 20 h. The crude material was purified by column chromatography (petroleum ether / EtOAc (9:1)) to give **7s** as a colourless oil (14 mg, 22 μmol, 68%); **R*f*** 0.43 (petroleum ether / EtOAc

(9:1)); **IR** (thin film, ν_{max} / cm^{-1}) 3070, 2956, 2929, 2857, 1471, 1354, 1164, 1111; **^{1}H NMR** (500 MHz, C_6D_6) δ_H 7.81-7.79 (4H, m, Ph*H*), 7.76 (2H, d, *J* = 8.0 Hz, Ts*H*), 7.28-7.23 (6H, m, Ph*H*), 6.74 (2H, d, *J* = 8.0 Hz, Ts*H*), 5.30 (1H, d, *J* = 12.0, H5), 4.90 (1H, d, *J* = 12.0 Hz, H4), 3.64 (1H, dd, *J* = 10.0 and 6.0 Hz, H6'), 3.56-3.50 (2H, m, H2 and 6'), 3.21-3.11 (2H, m, H2 and 3a), 3.08-3.02 (1H, m, H9), 2.76-2.69 (1H, m, H9), 2.59-2.52 (1H, m, H6), 2.51-2.46 (1H, m, H7), 2.43-2.37 (1H, m, H7), 1.88 (3H, s, TsCH_3), 1.86-1.77 (2H, m, H10), 1.54-1.47 (2H, m, H11), 1.45-1.34 (4H, m, H12 and 13), 1.22 (9H, s, SiC(CH_3)$_3$), 1.20-1.11 (1H, m, H3), 0.93 (3H, t, *J* = 7.0 Hz, H14), 0.59-0.51 (1H, m, H3); **^{13}C NMR** (125 MHz, C_6D_6) δ_C 143.1, 138.3, 137.8, 136.1, 134.1, 134.1, 133.2, 132.1, 131.0, 130.1, 129.5, 128.5, 68.7, 50.1, 40.5, 40.1, 36.0, 33.2, 32.4, 31.6, 30.3, 28.0, 27.2, 23.2, 21.2, 19.6, 14.5; **HRMS** (ESI+) calc. for $C_{39}H_{51}O_3NNaSSi$ $[M+Na]^+$ 664.3251, found 664.3241.

IV Computational Methods

All quantum chemical calculations were carried out with the Gaussian 09, D01program package.[5] Molecular geometries were fully optimized at the level of density functional theory, using the dispersion-corrected ω-B97XD[6] functional without any symmetry constraints. The effective core potentials (ECPs) of Hay and Wadt with a double-ζ basis set (LanL2DZ)[7] were used for Rh, S and P, and the 6-31G(d) basis set was used for H, C, N and O(BS1). The energies were further estimated using a larger basis set (6-311+G (d, p) basis set for H, C, N, O, S and P) and triple-ζ basis set (LanL2TZ)[8] for Rh (BS2) by single-point calculations, in implicit solvent treated with the SMD universal solvation model[9]. CH_2Cl_2 was used as solvent with a dielectric constant value of 8.93 and using UAHF (United Atom Hartree-Fock) radii for the respective atoms (Rh, H, C, N, O, S and P) in the SMD calculations. Structures of the ynamide substrate and a series of phospohoramidite ligands were computed in full, while the -NTs p-tolyl group was modeled as methyl group in the interests of computational tractability. Additional density functionals such as B3LYP[10], B3LYP-D3[11] and M06[12] were used with selected transition states to evaluate the functional influence. All optimized species were verified as either minima or transition structures by the presence of zero or a single imaginary vibrational frequency. Free energies were evaluated at 298K using harmonic vibrational frequencies. Saddle points were connected to minima in the usual way with intrinsic reaction coordinate (IRC) calculations[13]. The energies given throughout the paper are relative energy values computed with Gaussian 09 at 298 K and P=1 atm. NBO version 6.0[14] were employed to calculate the energy of second order perturbation for the proposed Rh-arene interaction. Computed structures are displayed with CYLVIEW[15] or PyMol[16]. Nonbonding interactions in stereo-determining transition structures were visualized using the NCI (Non-Covalent Interactions) visualization index, based on the density and its derivatives.[17] Promolecular densities were used to analyse the interaction surface between substrate and chiral ligand. NCI analysis has emerged as a means to probe the role of weak interactions in asymmetric catalysis.[18]

V Model for stereoinduction of transition states by chiral ligand

Having identified TS3 as the stereo-determining transition structure based on a full exploration of the potential energy surface with **L1**, the effect of varying the phosphoramidite ligand was studied on this step computationally. We separately optimized transition structure **TS3** with different ligands (**L4-L6**) at the ω-B97XD/6-31G(d)/Lanl2DZ (BS1) level of theory. The single point energy was further evaluated with a large basis set ω-B97XD/6-311+G (d, p)/Lanl2TZ(BS2) and an

implicit solvent treated SMD model. Details of atomic distances and dihedral angles are shown in Figure S1(a), which relate to the Rh-arene and π-π interactions as discussed in the main text. Across all of the ligands studied, the distance from Rh to the closest carbon (C_3) of the proximal arene group is in the region of 2.67-2.82 Å, and for **L5** this distance takes its longest value among all ligands studied. This suggests a weaker interaction between F-Ph with Rh, which corroborates recent collision-induced dissociation measurements of the effect of fluorination upon the relative binding affinities of cationic Rh-η^6-arene complexes.[19] The TS leading to the major enantiomer (alkene *Re* face) benefits from this interaction (ie. 2.69 Å in *Re* and 2.79 Å in *Si* for **L1**). While these interactions are largely electrostatic in origin, the energies (i.e. π donor to vacant *d*-orbital of Rh and Rh back donation to π* of C=C) obtained from second-order perturbation theory of the basis set of natural bonding orbitals (NBOs) indicate the presence of non-classical Rh-arene interactions shown in Figure S1(b), which suggested electron 2π donor from arene to vacant *d*-orbital of Rh majorly contributes to this interaction rather than back donations.

Another important attractive nonbonding interaction is apparent in **TS3** between the ligand biaryl backbone and the terminal alkyne substituent. For the phenyl-substituted alkyne this is a π-π interaction,[20-22] for which the physical origin lies in medium/long range electron correlation (i.e. dispersion effects), and we also expect attractive interactions to also occur when this substituent is aliphatic rather than aromatic. In **TS3** the corresponding two fragments (phenyl and naphthyl) are oriented nearly parallel to each other separated by a perpendicular distances of 3.49-3.93 Å, which should be assigned as a parallel-displaced shape. A closer distance between two centre of phenyl and naphthyl in **L5** suggests a tighter interaction within the substrate and ligand. The importance of this noncovalent interaction is underlined by the effect of partial saturation of the ligand backbone: we compute a significant drop in enantioselectivity to 7% ee as the gap between the two stereoisomers falls to 0.1 kcal mol^{-1}. This was confirmed experimentally, with an observed selectivity of 11% ee.

We present a simplified model in Figure S1(c), where **TS3** can be divided into three parts, which describe the critical interactions that enable chiral relay from the ligand to metal and substrate. The Rh-arene interaction as "left arm" represents an interaction between metal and ligand, the noncovalent π-π interaction as the "right arm" representing an interaction between substrate and ligand, both of which act on the main "body" in **TS3**, the metal-complexed substrate. We have found that the extent of organization in the transition state can be quantified in terms of the dihedral angle made between substrate alkyne atoms and the two points of contact between metal and ligand (P atoms and arene C atom). As shown in Figure S1(a), the largest difference in this dihedral angle

for the *Re* and *Si* TS is computed for the most selective reactions (L1 and L5). It is noticeable that the higher degree of coplanarity corresponds to the lower energy TSs, more closely corresponding to the optimial square-planar coordination of the metal centre. The excellent agreement between the computed selectivities and experimental results gives us confidence in this model to rationalize the selectivity.

VI NCI analysis

Optimized structures for TS show a parallel-displaced arrangment of ligand-backbone and substrate aromatic groups, which upon saturation of the ligand led to a marked erosion in enantioselecitivity. We further probed the existence and nature of noncovalent interactions involving the chiral ligand in TS3 using the Non-Covalent Interaction (NCI) index based on the reduced density gradient of the promolecular electron density, as shown in Figure S2. A large surface is seen between the two aromatic groups of the ligand-backbone and substrate, indicative of a weakly attractive interaction between the two, supporting our earlier interpretation of this attractive interaction between catalyst and substrate. It is difficult to make quantitative arguments based on this analysis, however, the fact that the energy difference between *Re*- and *Si*-face is lessened by 2.6 kcal mol^{-1} when the aromatic portion of the ligand is reduced (i.e. with **L6**) supports the idea that this interaction is more favourable in the *Re*-face TS. Also apparent in the NCI surface is the previously identified Rh-arene interaction, which can be seen as an attractive (blue) region between the metal and the closest carbon atom of the proximal arene group of the ligand. Both of these aspects provide further support for our simplified model in Figure 1(c), and rely only the density rather than distance-based criteria.

VII Functionals Analysis for TS3

Relative energy was further evaluated by other common functionals for selected transition state **TS3** with different coordination mode shown in Table S1. The selected transition states represent the closest energy gap based on our original calculation in main content, and therefore could be a good sample for functional evaluation. From table S1, the relative energy follows the order of B3LYP-D3 < ω-B97XD < M06 << B3LYP suggesting the significant importance of the dispersion within functional, which could be rationalized by a lot of non-covalent interaction. And the energy of our

chosen ω-B97XD functional right located in the middle of B3LYP-D3 and M06 functionals, thus should be fair good to investigate in this reaction.

Supplementary References

1. Alexakis, A., Gille, S., Prian, F., Rosset, S. & Ditrich, K. A practical, solvent free, one-pot synthesis of C2-symmetrical secondary amines. *Tetrahedron Lett.* **45**, 1449-1451, (2004).
2. Maksymowicz, R. M.;, Roth, P. M. C. & Fletcher, S. P. Catalytic asymmetric carbon–carbon bond formation using alkenes as alkylmetal equivalents. *Nature Chem.* **4**, 649-654, (2012).
3. Zhang, Y., Hsung, R. P., Tracey, M. R., Kurtz, K. C. M. & Vera, E. L. Copper Sulfate-Pentahydrate-1,10-Phenanthroline Catalyzed Amidations of Alkynyl Bromides. Synthesis of Heteroaromatic Amine Substituted Ynamides. *Org. Lett.* **6** (7), 1151-1154, (2004).
4. Mansfield, S. J.; Campbell, C. D.; Jones, M. W.; Anderson, E. A. A robust and modular synthesis of ynamides. *Chem. Commun.* **51**, 3316-3319, (2015).
5. Gaussian 09, Revision D.01, Frisch, M. J., Trucks, G. W., Schlegel, H. B., Scuseria, G. E., Robb, M. A., Cheeseman, J. R., Scalmani, G., Barone, V., Mennucci, B., Petersson, G. A., Nakatsuji, H., Caricato, M., Li, X., Hratchian, H. P., Izmaylov, A. F., Bloino, J., Zheng, G., Sonnenberg, J. L., Hada, M., Ehara, M., Toyota, K., Fukuda, R., Hasegawa, J., Ishida, M., Nakajima, T., Honda, Y., Kitao, O., Nakai, H., Vreven, T., Montgomery J. A., Jr., Peralta, J. E., Ogliaro, F., Bearpark, M., Heyd, J. J., Brothers, E., Kudin, K. N., Staroverov, V. N., Kobayashi, R., Normand, J., Raghavachari, K., Rendell, A., Burant, J. C., Iyengar, S. S., Tomasi, J., Cossi, M., Rega, N., Millam, J. M., Klene, M., Knox, J. E., Cross, J. B., Bakken, V., Adamo, C., Jaramillo, J., Gomperts, R., Stratmann, R. E., Yazyev, O., Austin, A. J., Cammi, R., Pomelli, C., Ochterski, J. W., Martin, R. L., Morokuma, K., Zakrzewski, V. G., Voth, G. A., Salvador, P., Dannenberg, J. J., Dapprich, S., Daniels, A. D., Farkas, Ö., Foresman, J. B., Ortiz, J. V., Cioslowski, J. & Fox, D. J., Gaussian, Inc., Wallingford CT, (2009).
6. Chai J.-D. & Head-Gordon, M. Long-range corrected hybrid density functionals with damped atom–atom dispersion corrections. *Phys. Chem. Chem. Phys.* **10**, 6615-6620, (2008).
7. Wadt, W. R. & Hay, P. J. *Ab initio* effective core potentials for molecular calculations. Potentials for main group elements Na to Bi. *J. Chem. Phys.* **82**, 284-298, (1985).
8. (a) Hay, P.J. & Wadt, W.R. *Ab initio* effective core potentials for molecular calculations. Potentials for K to Au including the outermost core orbitals. *J. Chem. Phys.* **82**, 299-310, (1985). (b) Roy, L.E., Hay, P.J. & Martin, R.L. Revised Basis Sets for the LANL Effective Core Potentials. *J. Chem. Theory Comput.* **4**, 1029-1031, (2008).
9. Marenich, A. V., Cramer, C. J., & Truhlar, D. G. Universal Solvation Model Based on Solute Electron Density and on a Continuum Model of the Solvent Defined by the Bulk Dielectric Constant and Atomic Surface Tensions. *J. Phys. Chem. B,* **113**, 6378-6396, (2009).

10. (a) Becke, A.D. Density-functional thermochemistry. III. The role of exact exchange. *J. Chem. Phys.* **98**, 5648-5652, (1993). (b) Lee, C., Yang, W., & Parr, R.G. Development of the Colle-Salvetti correlation-energy formula into a functional of the electron density. *Phys. Rev. B* **37**, 785-789, (1988). (c) Vosko, S.H., Wilk, L. & Nusair, M., Accurate spin-dependent electron liquid correlation energies for local spin density calculations: a critical analysis. *Can. J. Phys.* **58**, 1200-1211, (1980). (d) Stephens, P.J., Devlin, F.J., Chabalowski, C.F. & Frisch, M.J. *Ab Initio* Calculation of Vibrational Absorption and Circular Dichroism Spectra Using Density Functional Force Fields. *J. Phys. Chem.* **98**, 11623-11627, (1994).
11. Grimme, S., Antony, J., Ehrlich, S. & Krieg, H. A consistent and accurate *ab initio* parametrization of density functional dispersion correction (DFT-D) for the 94 elements H-Pu. *J. Chem. Phys*. **132**, 154104, (2010).
12. Zhao, Y. & Truhlar, D. G. The M06 suite of density functionals for main group thermochemistry, thermochemical kinetics, noncovalent interactions, excited states, and transition elements: two new functionals and systematic testing of four M06-class functionals and 12 other functionals. *Theor. Chem. Account.* **120**, 215-241, (2008).
13. (a) Hratchian, H. P. & Schlegel, H. B. Accurate reaction paths using a Hessian based predictor–corrector integrator. *J. Chem. Phys.* **120**, 9918-9924, (2004); (b) Hratchian, H. P. & Schlegel, H. B. Using Hessian Updating To Increase the Efficiency of a Hessian Based Predictor-Corrector Reaction Path Following Method. *J. Chem. Theory Comput.* **1**, 61-69, (2005).
14. NBO 6.0. Glendening, E. D., Badenhoop, J. K., Reed, A. E., Carpenter, J. E., Bohmann, J. A., Morales, C. M., Landis, C. R. & Weinhold, F. Theoretical Chemistry Institute, University of Wisconsin, Madison, WI, (2013); http://nbo6.chem.wisc.edu.
15. CYL view, 1.0b. Legault, C. Y. Université de Sherbrooke, (2009); http://www.cylview.org.
16. The PyMOL Molecular Graphics System, Version 1.5.0.4 Schrödinger, LLC.
17. (a) Johnson, E R., Keinan, S., Mori-Sanchez, P., Contreras-Garcia, J., Cohen, A. J. & Yang, W. Revealing Noncovalent Interactions. *J. Am. Chem. Soc.* **132**, 6498-6506, (2010); (b) Contreras-Garcia, J., Johnson, E. R., Keinan, S., Chaudret, R., Piquemal, J-P., Beratan, D. N. & Yang, W. NCIPLOT: A Program for Plotting Noncovalent Interaction Regions. *J. Chem. Theory Comput.* **7**, 625-632, (2011).
18. (a) Arbour, J. L., Rzepa, H. S., Contreras-García, J., Adrio, L. A., Barreiro, E. M. & Hii, K. K. Silver-Catalysed Enantioselective Addition of O–H and N–H Bonds to Allenes: A New Model for Stereoselectivity Based on Noncovalent Interactions. *Chem. Eur. J.* **18**, 11317–11324, (2012); (b) Paton, R. S. Dissecting non-covalent interactions in oxazaborolidinium catalyzed cycloadditions of maleimides. *Org. Biomol. Chem.* **12**, 1717-1720, (2014).

19. Pike, S. D., Pernik, I., Theron, R., McIndoe, J. S. & Weller, A. S. Relative binding affinities of fluorobenzene ligands in cationic rhodium bisphosphine η^6–fluorobenzene complexes probed using collision-induced dissociation. *J. Organomet. Chem.* **784**, 75–83, (2015).
20. See a theoretical pioneer paper on benzene dimer: Sinnokrot, M.O., Valeev, E.F., Sherrill, C. D. Estimates of the *Ab Initio* Limit for π–π Interactions: The Benzene Dimer. *J. Am. Chem. Soc.* **124** (36), 10887–10893, (2002).
21. Recent perspective paper for π-π interaction: Martinez, C. R. & Iverson, B. L. Rethinking the term "pi-stacking". *Chem. Sci.* **3**, 2191-2201, (2012).
22. Recent review paper for substituent effect of noncovalent interactions: Wheeler, S. E. Understanding Substituent Effects in Noncovalent Interactions Involving Aromatic Rings. *Acc. Chem. Res.* **46**, 1029–1038, (2013)
