# Supplementary material for: Computational ligand design in enantio- and diastereoselective ynamide [5+2] cycloisomerization
Source: Nat Commun. 2016 Jan 5;7:10109. doi: 10.1038/ncomms10109 (PMC4728367; doi:10.1038/ncomms10109)
Supplement: Supplementary Data 1 — xyz coordinates for computational modelling [file ncomms10109-s2.docx]

**Cartesian Coordinates**

| **10, P-*trans*-ene/Down *Re***  E_BS1_= -2546.367682  E_BS2_= -3270.186831  X Y Z  Rh -1.99473800 -0.17476600 0.30578600  P 0.05659000 1.03384300 0.35309600  O 1.23660900 0.45230700 1.40919600  O 0.94696600 1.12716000 -1.09370900  N -0.24131500 2.67666900 0.59607600  C 3.32400800 0.45738800 0.23361700  C 2.55848500 0.84845700 1.30953000  C 3.07638800 1.61077600 2.37709400  H 2.42859300 1.83122600 3.21895400  C 4.37822100 2.02695800 2.33366000  H 4.79575700 2.60416900 3.15368200  C 5.19769500 1.72902800 1.21344300  C 6.53100100 2.20905100 1.13287400  H 6.92385500 2.79587300 1.95870100  C 7.30598300 1.94804100 0.03363000  H 8.32248900 2.32411200 -0.02173900  C 6.77365900 1.19962200 -1.04041400  H 7.38338400 1.01329900 -1.91894200  C 5.49265400 0.71270300 -0.98812200  H 5.09705700 0.15154600 -1.82720700  C 4.66901400 0.94893300 0.14504300  C 2.74064900 -0.42598500 -0.81089200  C 1.59575800 -0.04079500 -1.46977600  C 1.03960300 -0.77439800 -2.53592900  H 0.16301600 -0.37850300 -3.03704600  C 1.61140300 -1.95889900 -2.90524100  H 1.19644800 -2.53385600 -3.72852400  C 2.73064000 -2.46949400 -2.19617100  C 3.27038400 -3.74776900 -2.50062100  H 2.84226700 -4.31058900 -3.32674500  C 4.30931800 -4.26436000 -1.77172000  H 4.71265800 -5.24350100 -2.00919000  C 4.86039300 -3.51762500 -0.70486400  H 5.67704700 -3.93506400 -0.12418000  C 4.37767600 -2.27013200 -0.40103900  H 4.80561100 -1.71031200 0.42305800  C 3.30359200 -1.70348800 -1.13961600  C 0.67655000 3.77124500 0.18750200  H 1.54264300 3.27357100 -0.25598900  C 1.16598200 4.57034400 1.39285100  H 1.73531600 3.91949500 2.06071600  H 0.34611900 5.01538500 1.96602800  H 1.82098000 5.38099500 1.06059400  C 0.00787900 4.59441000 -0.90541300  C -0.65185800 5.79601000 -0.64487100  H -0.64414800 6.22428400 0.35358700  C -1.31477300 6.47808800 -1.66457200  H -1.82066600 7.41328700 -1.44523100  C -1.31891500 5.97007800 -2.95869800  H -1.83008000 6.50442600 -3.75346000  C -0.64869500 4.77883600 -3.23286500  H -0.62958200 4.38478700 -4.24471300  C 0.00813800 4.09928500 -2.21409800  H 0.52976000 3.16978100 -2.42744500  C -1.51865600 3.00544800 1.23033600  H -1.57780100 4.09885300 1.20619800  C -1.54844800 2.55749900 2.69788700  H -0.66271300 2.94344300 3.20910700  H -1.53933900 1.46290000 2.77597100  H -2.43215700 2.92726200 3.22482500  C -2.72749600 2.53269100 0.41315400  C -4.02366400 2.75457800 0.91941300  H -4.15301900 3.10581200 1.93793300  C -5.14152600 2.60215400 0.11571700  H -6.12622900 2.81011700 0.52210000  C -5.00771800 2.19459200 -1.21833000  H -5.88631800 2.09292800 -1.84710200  C -3.74660100 1.93872800 -1.72747300  H -3.62011400 1.65124800 -2.76666300  C -2.60687900 2.10886300 -0.92435300  H -1.63398600 2.08312600 -1.40219500  C -1.16878700 -1.94284100 -0.43338100  C -0.77398900 -1.87141400 0.77175000  C 0.13022000 -2.17143600 1.84843100  C 1.37308600 -2.75299300 1.55595500  C -0.19160800 -1.86028500 3.17383200  C 2.26992100 -3.03150800 2.57707400  H 1.64519300 -2.94510600 0.52302900  C 0.71346500 -2.13443700 4.19111200  H -1.14779100 -1.39446700 3.38942200  C 1.94270500 -2.72149800 3.89632700  H 3.23083200 -3.47638200 2.33844000  H 0.46121400 -1.89257300 5.21878100  H 2.64777300 -2.93372800 4.69378200  N -1.52824800 -2.61059300 -1.55152900  C -2.55931600 -1.98005700 -2.38928800  C -3.89487200 -1.74195500 -1.64535500  H -2.69550800 -2.60958500 -3.27006700  H -2.12457500 -1.02771100 -2.70523500  H -4.37856900 -0.84712100 -2.04924500  H -4.56253800 -2.59188400 -1.83435100  C -3.71790100 -1.63705000 -0.15218200  C -4.18530500 -0.62757200 0.65214800  H -3.42568500 -2.56948000 0.32759800  H -4.66990400 0.22163000 0.17717000  C -4.37176500 -0.72109100 2.11799100  C -5.30021400 -1.79957000 2.66529400  C -3.83500400 -1.87125500 2.93921600  H -4.43147300 0.24396700 2.61353200  H -5.71180200 -2.49764900 1.94260600  H -5.97338900 -1.51994200 3.46902700  H -3.49496500 -1.63504700 3.94280900  H -3.25100000 -2.63879000 2.43880300  S -1.82930800 -4.41864600 -1.27499500  O -2.57434800 -4.61738100 0.04233800  O -2.40751900 -4.90185900 -2.59692200  C -0.09799400 -4.99684400 -1.06874300  H 0.47671700 -4.58051000 -1.89488700  H 0.24023400 -4.63285100 -0.09885300  H -0.13992900 -6.08635400 -1.09853400 | **10, P-*trans*-ene/Down *Si***  E_BS1_= -2546.370610  E_BS2_= -3270.189915  X Y Z  Rh -2.03756400 -0.05410000 0.47139800  P 0.17256200 0.86821900 0.46830000  O 1.30597800 0.19941800 1.53060400  O 1.01721500 0.76056500 -1.00381100  N 0.09271700 2.55020500 0.62295800  C 3.40432700 0.22331900 0.37163400  C 2.62967900 0.60562900 1.44492000  C 3.12961300 1.39109300 2.50375900  H 2.47735800 1.60654100 3.34348200  C 4.41806200 1.84741400 2.44968300  H 4.82148600 2.44451400 3.26254800  C 5.23620000 1.57582600 1.32212000  C 6.54728800 2.11060600 1.22059600  H 6.92630000 2.71902600 2.03721000  C 7.31689000 1.87604300 0.11179400  H 8.31537100 2.29505900 0.03979300  C 6.80294400 1.09807300 -0.95053500  H 7.40827000 0.93211300 -1.83610400  C 5.54541600 0.55661800 -0.87695600  H 5.16242700 -0.03064500 -1.70408900  C 4.72753200 0.76692900 0.26561500  C 2.85611300 -0.71928000 -0.63981800  C 1.68305200 -0.42060400 -1.29363200  C 1.13923700 -1.24277200 -2.30295400  H 0.22324800 -0.93259700 -2.79413200  C 1.78379200 -2.40015600 -2.64280600  H 1.39394900 -3.03061500 -3.43763600  C 2.95972300 -2.80500900 -1.95549400  C 3.58581700 -4.04694600 -2.23984200  H 3.17750100 -4.66813300 -3.03278800  C 4.67919700 -4.46206600 -1.52542800  H 5.14970900 -5.41433000 -1.74808700  C 5.19094100 -3.65069400 -0.48716900  H 6.04636900 -3.99136600 0.08773600  C 4.61617900 -2.43940200 -0.19778900  H 5.01232800 -1.83557300 0.61024700  C 3.49332100 -1.97115800 -0.93105700  C 1.11651300 3.50043000 0.10939200  H 1.93877400 2.87676300 -0.24929100  C 1.66217400 4.38668300 1.22725300  H 2.14801200 3.76937900 1.98570400  H 0.88402900 4.98036800 1.71791900  H 2.40160800 5.08190200 0.81926100  C 0.55637100 4.25983600 -1.08586200  C -0.09459200 5.48754600 -0.95062500  H -0.14768700 5.97943200 0.01668200  C -0.67028600 6.11336200 -2.05450900  H -1.17223600 7.06793300 -1.93025800  C -0.58950600 5.52494000 -3.31209000  H -1.03236000 6.01534400 -4.17335300  C 0.07788400 4.31105400 -3.46269800  H 0.16522000 3.85527600 -4.44448900  C 0.64560300 3.68565900 -2.35805700  H 1.15752600 2.73487200 -2.47587800  C -1.15730000 3.07505600 1.18252300  H -1.05810500 4.16328900 1.13393500  C -1.31110600 2.68914400 2.66008700  H -0.39359900 2.94700300 3.19578700  H -1.48585700 1.61128800 2.77834500  H -2.14297100 3.21362200 3.13760100  C -2.38947500 2.74994100 0.32704400  C -3.66211000 3.14420300 0.77892800  H -3.78034700 3.54325200 1.78106100  C -4.77087800 3.08815100 -0.05315500  H -5.73583300 3.42068900 0.31706100  C -4.64398600 2.63380000 -1.36946800  H -5.50808200 2.61600200 -2.02609300  C -3.40172100 2.23239800 -1.83338100  H -3.27761900 1.91377200 -2.86368000  C -2.28001300 2.28365300 -0.99579700  H -1.30388600 2.09516400 -1.42852100  C -1.59623300 -1.78056200 -0.54824200  C -1.03868400 -1.92880500 0.58462200  C -0.09207000 -2.49499800 1.50495400  C 1.01700600 -3.19044700 1.00329700  C -0.24235200 -2.33923300 2.88757500  C 1.95358300 -3.73151100 1.87329300  H 1.15572100 -3.27227000 -0.06959100  C 0.69803400 -2.87981700 3.75369300  H -1.09343400 -1.78533600 3.27059600  C 1.79579400 -3.57578100 3.24902400  H 2.81658300 -4.25288200 1.47141300  H 0.57741400 -2.75732900 4.82521600  H 2.53120600 -3.99227100 3.93016300  N -2.21644700 -2.26412800 -1.64831500  C -3.26939000 -1.41920400 -2.23334400  C -4.45577800 -1.16046200 -1.29077400  H -3.61805000 -1.91767200 -3.13966400  H -2.77589800 -0.48552600 -2.51562700  H -5.16273900 -0.53109500 -1.84167300  H -4.96706900 -2.11245300 -1.10938300  C -4.15781900 -0.48172500 0.03875800  C -3.84882300 -1.16962700 1.20222400  H -4.54669800 0.53180600 0.13459300  H -3.65397200 -2.23844800 1.11442700  C -4.03295000 -0.69322600 2.59124600  C -4.61613500 0.65001400 2.95579100  C -5.45075100 -0.57676300 3.13509900  H -3.32033100 -1.11610500 3.29431900  H -4.88891200 1.32767000 2.15538400  H -4.21325600 1.13734700 3.83835700  H -5.63048800 -0.95505300 4.13572500  H -6.25982700 -0.74217700 2.43042300  S -2.79032300 -4.02132800 -1.42632800  O -3.32054500 -4.20547400 -0.00778200  O -3.66147300 -4.29259800 -2.64365600  C -1.18000300 -4.89196900 -1.57726100  H -0.71960000 -4.53821800 -2.49828600  H -0.60050800 -4.63959700 -0.68930000  H -1.41512600 -5.95636200 -1.61565700 |
| --- | --- |
| **10, P-*trans*-ene/Up *Re***  E_BS1_= -2546.372734  E_BS2_= -3270.188583  X Y Z  Rh -2.02420600 -0.61033200 -0.42300500  P -0.07336800 0.62851100 0.05960700  O 0.89738900 -0.12304600 1.20749700  O 1.08010300 0.96170100 -1.15326300  N -0.47885700 2.23950400 0.37843300  C 3.17786000 0.34169400 0.65368400  C 2.13013100 0.40660800 1.54457500  C 2.24622500 0.95954800 2.83656900  H 1.38397500 0.91191900 3.49397600  C 3.43520500 1.51664600 3.21675300  H 3.54952600 1.94149500 4.21003800  C 4.52869300 1.57672200 2.31279800  C 5.73979000 2.22171600 2.67578100  H 5.82355000 2.65038700 3.67081000  C 6.77923300 2.31638800 1.78832300  H 7.69950900 2.81629500 2.07301200  C 6.64596700 1.77277300 0.49101900  H 7.46214900 1.86875400 -0.21826000  C 5.49376500 1.13137500 0.11442900  H 5.40714700 0.72861700 -0.88808700  C 4.40380300 0.99833800 1.01634800  C 3.01814800 -0.35391600 -0.65323100  C 2.00746600 0.00538600 -1.51654200  C 1.91183100 -0.49404100 -2.83285100  H 1.11635300 -0.12315500 -3.47129900  C 2.83530200 -1.39680900 -3.27831600  H 2.79290000 -1.77008100 -4.29787500  C 3.83242400 -1.89654800 -2.40092000  C 4.72734700 -2.91670800 -2.81610200  H 4.66760300 -3.27789000 -3.83962300  C 5.63991800 -3.44853800 -1.94266000  H 6.31981000 -4.22949200 -2.26860300  C 5.69302100 -2.98107100 -0.60953400  H 6.40561700 -3.41631100 0.08431700  C 4.85548000 -1.98131500 -0.18529700  H 4.90488900 -1.63439200 0.84079600  C 3.90869400 -1.39949400 -1.06933900  C 0.50497000 3.35586800 0.38018900  H 1.46596100 2.90142400 0.12826000  C 0.63646300 3.98126300 1.76680000  H 0.97006200 3.22632900 2.48194700  H -0.30692700 4.40113100 2.13053600  H 1.37784000 4.78541900 1.74375100  C 0.15559200 4.33983200 -0.73163100  C -0.43992500 5.57647400 -0.47822300  H -0.63024500 5.89580900 0.54210400  C -0.78669800 6.42883600 -1.52569300  H -1.24878800 7.38695700 -1.30846800  C -0.53489900 6.05820900 -2.84142600  H -0.80019900 6.72448300 -3.65641900  C 0.07131300 4.83102600 -3.10551900  H 0.28711300 4.54139200 -4.12976400  C 0.41170700 3.98130100 -2.06011900  H 0.88872300 3.02745500 -2.26569400  C -1.88742000 2.56756800 0.62422800  H -1.92889200 3.66109400 0.56325600  C -2.34771200 2.14017600 2.01927000  H -1.59035600 2.40381300 2.75961900  H -2.48720900 1.05909000 2.05634100  H -3.28789300 2.61503800 2.31100900  C -2.82856400 2.08346200 -0.48444200  C -4.21470100 2.25373700 -0.32335900  H -4.60791600 2.62067100 0.61841800  C -5.09488400 2.01199500 -1.36823100  H -6.15694800 2.18228600 -1.22104200  C -4.62635500 1.56104500 -2.60675800  H -5.32011100 1.38129900 -3.42159100  C -3.26652500 1.36797900 -2.78281800  H -2.87404600 1.05271100 -3.74481200  C -2.36675400 1.62738900 -1.73556000  H -1.30748000 1.65942600 -1.97076300  C -1.85598200 -1.97367100 1.28489900  C -1.01197300 -2.23140200 0.38170300  C 0.13988400 -3.00601200 -0.01605700  C 0.44734300 -3.21490400 -1.36275500  C 0.95294600 -3.56569800 0.98037300  C 1.52137000 -4.02478000 -1.70957300  H -0.15257100 -2.73026900 -2.12578400  C 2.04433800 -4.34511900 0.62471900  H 0.73293600 -3.35949200 2.02282500  C 2.32030300 -4.58936200 -0.71973500  H 1.75300700 -4.19331400 -2.75582000  H 2.68483700 -4.76160400 1.39538700  H 3.17795700 -5.19314300 -0.99735100  N -2.73296800 -2.17803500 2.27266400  C -4.14234000 -2.39076300 1.91000300  C -4.73074500 -1.31287600 1.00186200  H -4.71956400 -2.42466300 2.83752300  H -4.19707400 -3.38338900 1.45097200  H -5.80625400 -1.52060100 0.91268100  H -4.65029700 -0.34436000 1.50740700  C -4.18831400 -1.19396700 -0.40467700  C -3.56812900 -2.16507100 -1.15934400  H -4.62158400 -0.35902100 -0.95316600  H -3.28452000 -3.10663600 -0.68921700  C -3.54222000 -2.17933100 -2.63748200  C -2.63209500 -3.13366400 -3.37592900  C -4.10228900 -3.40209800 -3.34529800  H -3.74967500 -1.22599300 -3.11204700  H -1.99735600 -3.78008600 -2.77728900  H -2.18260000 -2.78951200 -4.30205300  H -4.68619900 -3.22598400 -4.24269400  H -4.45672200 -4.22463200 -2.73110600  S -2.46531600 -1.13524000 3.77775600  O -1.09152200 -0.51090700 3.59520900  O -3.72089400 -0.29946100 4.00716300  C -2.37333800 -2.45099700 5.05628000  H -2.21670400 -1.94370000 6.00898600  H -3.32273100 -2.98544700 5.03892000  H -1.53160100 -3.08678600 4.78531800 | **10, P-*trans*-ene/Up *Si***  E_BS1_= -2546.365124  E_BS2_= -3270.182526  X Y Z  Rh -1.96410500 -0.82805000 -0.43869100  P -0.22006800 0.60401000 0.12745600  O 0.84540500 -0.02523300 1.26426000  O 0.88957200 1.00722700 -1.10669900  N -0.78227500 2.16259300 0.43258100  C 3.07683400 0.53520200 0.58701600  C 2.06380800 0.57966600 1.52023100  C 2.20779300 1.18127600 2.78751300  H 1.37975900 1.11009000 3.48511900  C 3.38081500 1.81048600 3.09701100  H 3.51524800 2.27254800 4.07092400  C 4.42982400 1.89503900 2.14400800  C 5.62083700 2.61104300 2.43234000  H 5.72590700 3.07457500 3.40953200  C 6.61415700 2.72885800 1.49635100  H 7.51984700 3.28166000 1.72454700  C 6.45206200 2.13909200 0.22255800  H 7.23018100 2.25449600 -0.52553600  C 5.31938000 1.42823000 -0.08171700  H 5.20892400 0.99190700 -1.06778400  C 4.27951600 1.26750300 0.87341400  C 2.89334800 -0.21920000 -0.68291500  C 1.81466700 0.05663800 -1.49185800  C 1.62751700 -0.54337900 -2.75401900  H 0.76992400 -0.24528900 -3.34918500  C 2.54121100 -1.45345300 -3.20590200  H 2.42553200 -1.90954000 -4.18514400  C 3.63704700 -1.83813100 -2.39095700  C 4.55326700 -2.83227100 -2.82213800  H 4.42411600 -3.26638900 -3.81020800  C 5.57560600 -3.24644300 -2.00897400  H 6.27333900 -4.00547800 -2.34869300  C 5.71731000 -2.68779900 -0.71831100  H 6.51540400 -3.03282500 -0.06813400  C 4.85562400 -1.71567300 -0.27867700  H 4.97321000 -1.30127500 0.71616200  C 3.80173700 -1.24545900 -1.10662600  C 0.11828600 3.34045900 0.55558100  H 1.12805900 2.95949000 0.37812500  C 0.07630200 3.91375700 1.97003100  H 0.36644900 3.14446800 2.68857300  H -0.92071200 4.27295900 2.24407600  H 0.77531200 4.75092900 2.05624100  C -0.18912500 4.34780300 -0.54771600  C -0.85424300 5.54969100 -0.29923700  H -1.14037900 5.82029100 0.71257600  C -1.14933200 6.42935700 -1.33977700  H -1.66500000 7.36058200 -1.12613100  C -0.77762700 6.12047900 -2.64297000  H -1.00350900 6.80794200 -3.45222100  C -0.10256000 4.92852900 -2.90086200  H 0.20581700 4.68707800 -3.91383500  C 0.18833100 4.05205200 -1.86253500  H 0.71969000 3.12642200 -2.06322000  C -2.23184900 2.38528700 0.51522200  H -2.33763200 3.47020100 0.39536900  C -2.83289300 1.99247100 1.86761700  H -2.12759700 2.20859600 2.67047800  H -3.05904700 0.92456600 1.89963800  H -3.75874700 2.53787500 2.07169400  C -3.00383500 1.79759800 -0.67030500  C -4.41090300 1.78212600 -0.62705100  H -4.92519300 2.03320300 0.29407500  C -5.15976400 1.51197600 -1.76351800  H -6.24361500 1.54541200 -1.70821800  C -4.53105800 1.21158700 -2.97670100  H -5.12161200 1.01442400 -3.86553800  C -3.14826700 1.17161600 -3.02973300  H -2.64067100 0.94491900 -3.96151000  C -2.38348900 1.46363800 -1.89009700  H -1.31278600 1.59217900 -2.01136200  C -1.64308600 -2.04484700 1.37324500  C -0.81127400 -2.30495500 0.45308100  C 0.40155100 -3.03098800 0.13624000  C 0.63658400 -3.55439800 -1.13762500  C 1.33962200 -3.23915300 1.15881800  C 1.76852200 -4.32623500 -1.37044400  H -0.06749700 -3.36259700 -1.93898700  C 2.48024200 -3.98840700 0.90966100  H 1.17180200 -2.78465900 2.12982900  C 2.68825500 -4.54697800 -0.35031200  H 1.94350500 -4.74034100 -2.35808100  H 3.21336100 -4.13399200 1.69646300  H 3.58414800 -5.12657100 -0.54537200  N -2.46311200 -2.23358100 2.40667100  C -3.82536600 -2.72361800 2.15768000  C -4.48827300 -2.11131300 0.92017600  H -4.42634300 -2.50286300 3.04288300  H -3.75504800 -3.81149400 2.04760500  H -5.48537200 -2.56642000 0.84684100  H -4.63275100 -1.03376600 1.05186400  C -3.73935700 -2.42068600 -0.35204500  C -3.81250900 -1.70478500 -1.51029400  H -3.29629200 -3.41528100 -0.39810300  H -4.41109000 -0.79506100 -1.50647900  C -3.44532900 -2.21125900 -2.86653900  C -2.05876300 -2.07624700 -3.45992400  C -2.59662900 -3.43033700 -3.08781400  H -4.25395900 -2.06366600 -3.57948600  H -1.27806600 -1.65695700 -2.82719800  H -1.97903100 -1.81475800 -4.51072700  H -2.88587500 -4.10989200 -3.88260100  H -2.16364900 -3.92076600 -2.22053400  S -2.29290800 -1.04003800 3.80860200  O -1.06592100 -0.19960000 3.49462100  O -3.66776100 -0.43044200 4.05810200  C -1.89850700 -2.20954500 5.16909000  H -1.80686100 -1.61010600 6.07565200  H -2.72679100 -2.91456700 5.23578300  H -0.96091300 -2.69364900 4.89907500 |
| **10, P-*trans*-yne/Down *Re***  E_BS1_= -2546.364375  E_BS2_= -3270.181704  X Y Z  Rh -1.48123300 -0.26907600 -0.07791100  P 0.75551400 0.55268600 -0.00236300  O 1.63958900 -0.25256900 1.20963700  O 1.84829500 0.53518900 -1.30487300  N 0.72550500 2.21956300 0.28572200  C 3.90878000 -0.24966700 0.47957600  C 2.96274300 0.09884200 1.41583000  C 3.28536900 0.77396800 2.61092400  H 2.49773400 0.96822900 3.33052100  C 4.58239200 1.14527200 2.83880100  H 4.85144500 1.65367400 3.76035400  C 5.59016400 0.89596700 1.87051300  C 6.92492800 1.33449100 2.07154300  H 7.16885900 1.85527700 2.99346800  C 7.88639200 1.11405900 1.12073500  H 8.90363800 1.45628000 1.28137900  C 7.55062200 0.44667100 -0.07937300  H 8.31235700 0.29022300 -0.83674400  C 6.27269300 0.00003600 -0.29879000  H 6.02992100 -0.50325300 -1.22821600  C 5.25602300 0.20020900 0.67288600  C 3.51389700 -1.07849900 -0.69211700  C 2.54037300 -0.64111100 -1.56127800  C 2.21800000 -1.32743100 -2.75171900  H 1.50392000 -0.87933500 -3.43572600  C 2.82450800 -2.52255000 -3.02340200  H 2.59407500 -3.05750600 -3.94020300  C 3.74809700 -3.08944900 -2.10679800  C 4.32338600 -4.36581100 -2.33930300  H 4.05820900 -4.89960300 -3.24784500  C 5.19104600 -4.91886300 -1.43440700  H 5.62509000 -5.89630300 -1.61850500  C 5.51708100 -4.21742600 -0.25141000  H 6.19255200 -4.66677000 0.46970600  C 4.98868200 -2.97615100 -0.00470400  H 5.24452700 -2.45374600 0.91008900  C 4.09740600 -2.36838800 -0.92895200  C 1.74935500 3.18736500 -0.18718800  H 2.51977600 2.58316700 -0.66840400  C 2.41738400 3.94931600 0.95447600  H 2.93875500 3.25148700 1.61467600  H 1.70911700 4.52774900 1.55624100  H 3.15121400 4.64898500 0.54344900  C 1.11149900 4.05979600 -1.26025200  C 0.58882300 5.32555700 -0.99573100  H 0.70088900 5.77241200 -0.01193500  C -0.06991000 6.04439000 -1.99336000  H -0.47246700 7.02775900 -1.77084800  C -0.20520800 5.50884900 -3.26906800  H -0.71329700 6.07156200 -4.04603500  C 0.32989700 4.25189600 -3.54889200  H 0.24629100 3.83444700 -4.54805500  C 0.98083900 3.53568500 -2.55165700  H 1.39576600 2.55435900 -2.76785100  C -0.39202000 2.70207200 1.10679300  H -0.38939900 3.78991500 0.97208100  C -0.17512600 2.40350500 2.59386700  H 0.84619400 2.67407800 2.86909600  H -0.32068600 1.33913900 2.80992200  H -0.85445000 2.98057300 3.22715700  C -1.76082800 2.25543100 0.57481700  C -2.87226300 2.18581000 1.43766500  H -2.72797900 2.17873300 2.51179500  C -4.16506600 2.13450000 0.93448400  H -5.00352600 2.07069800 1.61951600  C -4.38764700 2.14968300 -0.44082600  H -5.40442400 2.12176900 -0.81834400  C -3.30791700 2.17858000 -1.31429000  H -3.46578100 2.18109900 -2.38798400  C -2.00330500 2.22647100 -0.81701700  H -1.17297300 2.35994500 -1.50548100  C -3.53969800 -0.90498800 -0.24674000  C -3.30422200 -0.89991900 0.97155700  C -3.33879100 -0.87689300 2.40748300  C -2.16555600 -0.92279900 3.17073200  C -4.58801500 -0.76749600 3.03462100  C -2.24430600 -0.85590000 4.55707100  H -1.20167500 -1.00962600 2.67545300  C -4.65029000 -0.70303800 4.42125000  H -5.48616800 -0.73606300 2.42526800  C -3.48358200 -0.74337400 5.18360200  H -1.33488500 -0.89405600 5.14865600  H -5.61620700 -0.62089400 4.90923000  H -3.54197400 -0.69031600 6.26610300  N -4.17839000 -1.15026900 -1.42347300  C -3.57236800 -2.26859800 -2.17532500  C -2.15014000 -1.91018600 -2.58697100  H -4.16938100 -2.42677600 -3.07722700  H -3.60609100 -3.19477000 -1.58908700  H -1.80863600 -2.69367600 -3.27820600  H -2.17422100 -0.97825400 -3.16424000  C -1.08398200 -1.79269200 -1.51696100  C -1.02505900 -2.42439500 -0.26254800  H -0.11697800 -1.56319900 -1.96052900  H -1.91159600 -2.93845900 0.10861300  C 0.24710500 -2.88986600 0.33137700  C 0.31596900 -3.32439100 1.77223600  C 0.37801800 -4.35888600 0.69182400  H 1.14482800 -2.43391900 -0.07214000  H -0.59728100 -3.26958100 2.35847300  H 1.22187700 -3.07926400 2.31641400  H 1.33091500 -4.82955400 0.47174600  H -0.48773900 -4.99947300 0.55004200  S -5.98619600 -1.42050900 -1.12302900  O -6.27359200 -2.91379600 -1.18142000  O -6.32610300 -0.57744000 0.09914200  C -6.66402300 -0.60232100 -2.62046600  H -6.29489900 0.42182900 -2.60666300  H -6.31184900 -1.16138300 -3.48672700  H -7.74971700 -0.65144500 -2.52887300 | **10, P-*trans*-yne/Down *Si***  E_BS1_= -2546.352676  E_BS2_= -3270.170466  X Y Z  Rh -1.33784000 -0.38511100 0.13118300  P 0.80055300 0.71423300 0.21245700  O 1.85698200 0.08627900 1.37846300  O 1.80964800 0.74295200 -1.16113100  N 0.59956000 2.37476200 0.42756100  C 3.99645000 -0.15815900 0.35248800  C 3.20964700 0.37115400 1.35068100  C 3.73275700 1.14697500 2.40526500  H 3.05818000 1.49317900 3.18123300  C 5.07302700 1.41796100 2.43245800  H 5.49578600 2.00418100 3.24337800  C 5.93091600 0.94953200 1.40281300  C 7.31562200 1.26013300 1.40711300  H 7.71349200 1.85412300 2.22533300  C 8.13545500 0.82627300 0.39883400  H 9.19323800 1.06846900 0.41024500  C 7.59945100 0.06763300 -0.66631700  H 8.25023600 -0.25912400 -1.47130300  C 6.26673600 -0.25516300 -0.69654000  H 5.87189300 -0.82945700 -1.52730300  C 5.39358700 0.16300700 0.34295300  C 3.37529600 -1.00501500 -0.70254900  C 2.36006700 -0.49080900 -1.47832700  C 1.80777300 -1.18110100 -2.57687700  H 1.06184200 -0.67769700 -3.18325200  C 2.21836400 -2.45955500 -2.83734100  H 1.80652800 -3.00801400 -3.67980300  C 3.16169800 -3.10030700 -1.99062400  C 3.50911100 -4.46341800 -2.17973900  H 3.06122700 -5.00580700 -3.00793900  C 4.38433300 -5.08783200 -1.33011800  H 4.63711600 -6.13275900 -1.47800800  C 4.95664700 -4.37178100 -0.25364700  H 5.63890700 -4.87631200 0.42295600  C 4.65717600 -3.04819300 -0.05530900  H 5.09916500 -2.51026000 0.77615000  C 3.75429400 -2.37116600 -0.91836000  C 1.55544700 3.41310300 -0.03513500  H 2.37957900 2.86633400 -0.49800400  C 2.13720500 4.22493200 1.11970300  H 2.70344900 3.56955400 1.78645300  H 1.37059700 4.73659000 1.71010300  H 2.81831400 4.98547700 0.72672300  C 0.87921100 4.23279200 -1.12587200  C 0.27042200 5.46312900 -0.87831800  H 0.33004300 5.91915200 0.10587700  C -0.40819500 6.13628100 -1.89394700  H -0.87618300 7.09332700 -1.68518100  C -0.47917400 5.59001100 -3.17034700  H -1.00267400 6.11765900 -3.96157800  C 0.13979100 4.36833500 -3.43202500  H 0.10564300 3.94323200 -4.43084200  C 0.81174500 3.69777000 -2.41727700  H 1.29294300 2.74404800 -2.61967400  C -0.58485700 2.76875600 1.19632700  H -0.64205200 3.85816400 1.08972700  C -0.42629900 2.43499700 2.68375100  H 0.54317200 2.79675100 3.03514400  H -0.46876700 1.35275400 2.84763800  H -1.20002500 2.90438300 3.29649600  C -1.88438700 2.25268400 0.56944800  C -3.08620000 2.30221300 1.30459900  H -3.06333900 2.46252800 2.37626800  C -4.30805400 2.15567300 0.67520800  H -5.22036900 2.18468300 1.26155700  C -4.37867500 1.96888400 -0.71036900  H -5.34867400 1.86940400 -1.18476000  C -3.21340100 1.89345800 -1.44988500  H -3.24380100 1.74923000 -2.52513700  C -1.96745000 2.01596200 -0.81766300  H -1.07313800 2.09118100 -1.42828400  C -3.29318500 -1.02715700 -0.33253700  C -3.31156700 -0.82761700 0.90010700  C -3.70499000 -0.77743800 2.28324200  C -2.75279600 -0.68218900 3.30438400  C -5.07117800 -0.81564900 2.59682300  C -3.16329000 -0.62715100 4.63131900  H -1.69651200 -0.65629500 3.05069200  C -5.46807900 -0.75040300 3.92702900  H -5.79863900 -0.88811700 1.79449600  C -4.52017600 -0.65596000 4.94485400  H -2.42176400 -0.55939800 5.42123900  H -6.52579100 -0.77802600 4.16948000  H -4.83933300 -0.60721800 5.98114700  N -3.65803400 -1.37562500 -1.60090700  C -2.96901500 -2.55839000 -2.17385600  C -1.46925000 -2.54268400 -1.87505200  H -3.13793700 -2.54821700 -3.25382800  H -3.41671100 -3.47411900 -1.76996000  H -1.04888700 -3.46949300 -2.28784700  H -0.97563700 -1.71133900 -2.38581900  C -1.22539500 -2.50001500 -0.38372500  C -0.02289100 -2.29497900 0.27779300  H -1.97586700 -3.03499300 0.19943300  H 0.87417400 -2.09685100 -0.30244600  C 0.19753000 -2.84397300 1.63396300  C 1.13657300 -4.04584600 1.71962700  C 1.52356400 -2.74436300 2.33914400  H -0.69227500 -2.89929200 2.25647500  H 1.65668400 -4.34000800 0.81122200  H 0.82312700 -4.87009500 2.35196700  H 1.50128300 -2.64936300 3.41994800  H 2.31023900 -2.18050000 1.85315300  S -5.49787200 -1.56379400 -1.73340700  O -5.82294900 -3.04529400 -1.85710700  O -6.09369700 -0.69804600 -0.63200800  C -5.76603500 -0.74167600 -3.35260900  H -5.36545800 0.26616000 -3.25698400  H -5.24188600 -1.32985500 -4.10517100  H -6.84337500 -0.74380400 -3.52268700 |
| **10, P-*trans*-yne/Up *Re***  E_BS1_= -2546.357943  E_BS2_= -3270.173041  X Y Z  Rh -1.47102900 -0.51945900 -0.12053800  P 0.68104800 0.47341300 -0.04923900  O 1.53255900 -0.15527500 1.29022500  O 1.85187100 0.30902800 -1.27229100  N 0.61565700 2.15959300 -0.01668900  C 3.84084900 -0.13487900 0.68507000  C 2.82628600 0.29432500 1.51003400  C 3.03742700 1.17049600 2.59379700  H 2.19406500 1.43758800 3.22184900  C 4.29650400 1.65332400 2.82484400  H 4.48143800 2.32260200 3.66032100  C 5.37219600 1.31620700 1.96207600  C 6.66509900 1.86946200 2.15445100  H 6.82381500 2.54416200 2.99128600  C 7.69093600 1.57116700 1.29684800  H 8.67442200 2.00411200 1.44850100  C 7.46424400 0.70615000 0.20218700  H 8.27496500 0.48674400 -0.48545200  C 6.22970100 0.14616800 -0.00466600  H 6.07186600 -0.50862500 -0.85433400  C 5.14835900 0.42314200 0.87422400  C 3.55072600 -1.12991100 -0.38405700  C 2.59869400 -0.85535600 -1.34115100  C 2.36459900 -1.69504300 -2.44957200  H 1.65227200 -1.37310000 -3.20216700  C 3.04629000 -2.87532700 -2.55313100  H 2.88288400 -3.53069200 -3.40389900  C 3.96350100 -3.27017000 -1.54464700  C 4.62648900 -4.52325800 -1.60905000  H 4.42901300 -5.17428900 -2.45639100  C 5.49349100 -4.90969500 -0.62142400  H 5.99475000 -5.87050500 -0.67852400  C 5.73110100 -4.05661700 0.48028200  H 6.40901700 -4.37332400 1.26662000  C 5.11401100 -2.83500500 0.56678300  H 5.30356500 -2.19330800 1.42000000  C 4.21980600 -2.39775900 -0.44722400  C 1.66287500 3.05803600 -0.57962400  H 2.48605600 2.40336200 -0.86669900  C 2.20348200 4.03965200 0.45730800  H 2.66105700 3.49360500 1.28578900  H 1.43233400 4.70022200 0.86565100  H 2.96805000 4.66960800 -0.00675700  C 1.11896000 3.70379500 -1.84799600  C 0.54418600 4.97546600 -1.85703400  H 0.53655100 5.58194400 -0.95581000  C -0.01395900 5.49621400 -3.02364400  H -0.45965100 6.48599800 -3.01045200  C 0.00637300 4.75560800 -4.20002200  H -0.42339700 5.16430200 -5.10918800  C 0.59519400 3.49214400 -4.20758500  H 0.63433300 2.91622100 -5.12756100  C 1.14467300 2.97252200 -3.04124600  H 1.60359500 1.98763100 -3.04876000  C -0.59428900 2.76689900 0.55180800  H -0.57187600 3.80450100 0.19898800  C -0.55545700 2.78866900 2.08172200  H 0.43068200 3.12307800 2.41174000  H -0.74765900 1.79855600 2.50284500  H -1.29965000 3.47096500 2.49933400  C -1.88489300 2.20367600 -0.04478600  C -3.10863400 2.39839800 0.61342000  H -3.12865500 2.73568500 1.64344700  C -4.31053400 2.16815200 -0.04109700  H -5.24093700 2.31493100 0.49582700  C -4.32893400 1.74233600 -1.37014000  H -5.27310200 1.57054100 -1.87596500  C -3.13260900 1.50214100 -2.02582100  H -3.12987800 1.16342200 -3.05672700  C -1.91071700 1.71219600 -1.36967200  H -0.99155200 1.68462500 -1.95014400  C -3.11995800 -0.91733900 1.18114100  C -3.59908500 -1.00879100 0.02947000  C -4.61909400 -1.31789100 -0.93950200  C -5.95825500 -1.33035100 -0.52446200  C -4.30293700 -1.58208600 -2.27632800  C -6.96201400 -1.60471800 -1.44553100  H -6.19442100 -1.09736000 0.50910400  C -5.31173700 -1.86772800 -3.18750700  H -3.26400200 -1.55080500 -2.59018900  C -6.64316600 -1.87633200 -2.77512200  H -7.99894800 -1.60486100 -1.12441100  H -5.06239900 -2.07496600 -4.22359900  H -7.43111200 -2.09128500 -3.48991400  N -2.95509900 -1.06385300 2.53191500  C -1.55369500 -1.09649300 3.00804900  C -0.76677700 -2.23664500 2.34126000  H -1.57323200 -1.23565200 4.09188700  H -1.06237900 -0.14303900 2.79676700  H 0.29659700 -2.00159300 2.43700200  H -0.96019400 -3.17739700 2.86974000  C -1.14651200 -2.43919100 0.89396400  C -0.30492800 -2.40997400 -0.21425700  H -2.07360200 -2.99026100 0.76500300  H 0.75234900 -2.21034100 -0.04814600  C -0.60569000 -3.08911800 -1.50036200  C -0.57244200 -4.60869700 -1.52532300  C -1.86047600 -3.88099900 -1.75941500  H -0.15264500 -2.61021800 -2.36211500  H -0.44302000 -5.11276400 -0.57213400  H -0.07641100 -5.08711700 -2.36345400  H -2.25733200 -3.85389600 -2.76897100  H -2.63031800 -3.92767100 -0.99553700  S -3.96014400 0.19120700 3.45300000  O -3.04226800 1.31944100 3.91640900  O -5.17641000 0.46231800 2.58052500  C -4.46816800 -0.81932800 4.90051600  H -5.11668900 -0.17941700 5.50018500  H -4.99794700 -1.68297100 4.50144600  H -3.56626900 -1.09395700 5.44610000 | **10, P-*trans*-yne/Up *Si***  E_BS1_= -2546.372669  E_BS2_= -3270.184481  X Y Z  Rh -1.62252600 0.54486200 -0.23889900  P 0.62254700 -0.30214600 -0.24734800  O 1.59954200 0.09585100 -1.57673200  O 1.68129700 0.03729500 1.03813800  N 0.52551500 -1.98031700 -0.00910900  C 3.85624100 -0.12927500 -0.79952700  C 2.84713400 -0.50999300 -1.65677900  C 3.01080600 -1.50769800 -2.63891600  H 2.18203600 -1.72414500 -3.30452800  C 4.20406600 -2.16988600 -2.72806500  H 4.35196700 -2.93255800 -3.48740100  C 5.24803700 -1.90508500 -1.80390100  C 6.45489600 -2.65182200 -1.83032700  H 6.57777500 -3.41578900 -2.59320300  C 7.44015800 -2.42578000 -0.90587900  H 8.35766400 -3.00497600 -0.93000300  C 7.25644900 -1.44153900 0.09187000  H 8.03054800 -1.28097700 0.83571300  C 6.10912700 -0.69163700 0.13379100  H 5.98255500 0.05496800 0.90971600  C 5.07459600 -0.88889100 -0.82013100  C 3.66630900 1.02675600 0.11996500  C 2.60475700 1.06150900 0.99616900  C 2.43241400 2.08835800 1.94849700  H 1.60074400 2.01653400 2.64206400  C 3.31775200 3.12807700 1.98580800  H 3.20698700 3.91813200 2.72306500  C 4.38316800 3.20207900 1.04993400  C 5.26589900 4.31291500 1.03710300  H 5.13127000 5.09351700 1.78096400  C 6.26243400 4.40661400 0.10195300  H 6.93223400 5.26052900 0.09761800  C 6.40974300 3.39235700 -0.87071600  H 7.18443000 3.48122800 -1.62581400  C 5.58175200 2.29885200 -0.87520600  H 5.70556400 1.53559400 -1.63479900  C 4.55391800 2.15739600 0.09582600  C 1.58030800 -2.81129700 0.63477100  H 2.45949800 -2.16902500 0.70480600  C 1.95460500 -4.00354600 -0.24392900  H 2.33776200 -3.65001900 -1.20340200  H 1.10912000 -4.67110600 -0.43896500  H 2.73466300 -4.59086000 0.24923600  C 1.16536900 -3.18247700 2.05436000  C 0.44507500 -4.34417700 2.34251800  H 0.22618400 -5.06602500 1.56061000  C 0.01177100 -4.61287000 3.63914700  H -0.54741700 -5.52133800 3.84097300  C 0.30565500 -3.72963100 4.67259200  H -0.02802600 -3.94095700 5.68376200  C 1.04780800 -2.58208700 4.40330000  H 1.30384700 -1.89730700 5.20640100  C 1.47318800 -2.31383300 3.10680100  H 2.04547200 -1.41463800 2.90228600  C -0.79396300 -2.57555000 -0.24653700  H -0.72508700 -3.59946800 0.13098600  C -1.08817900 -2.65014300 -1.74780400  H -0.29215900 -3.21898600 -2.23693600  H -1.10290100 -1.64386000 -2.18285900  H -2.04776900 -3.11979500 -1.97178400  C -1.90204700 -1.92467800 0.59227000  C -3.23570100 -2.32780200 0.41532800  H -3.50062400 -2.98372500 -0.40671700  C -4.22852200 -1.92285400 1.29666300  H -5.24705100 -2.25109400 1.12814700  C -3.92997100 -1.08879300 2.37491400  H -4.71287700 -0.77057900 3.05509500  C -2.62523300 -0.66799900 2.56677400  H -2.36881200 -0.03266200 3.40812300  C -1.61065000 -1.08223300 1.68987000  H -0.58181800 -0.90030200 1.98430400  C -3.78935600 0.72916000 -0.99771400  C -3.61242900 1.25450400 0.11964700  C -4.08323900 2.04066700 1.23603200  C -5.46369100 2.12784200 1.46230600  C -3.19418000 2.69573400 2.09537700  C -5.94209300 2.86562000 2.53769100  H -6.14392600 1.59621800 0.80421400  C -3.68147400 3.44478700 3.15921000  H -2.12510300 2.61078800 1.92288700  C -5.05426500 3.52824300 3.38373400  H -7.01107100 2.92702700 2.71484400  H -2.99025900 3.96039600 3.81833700  H -5.43260400 4.10715700 4.22020800  N -4.14811200 0.36155700 -2.23041100  C -3.16110800 0.51322100 -3.31577600  C -2.36808500 1.79403000 -3.07775300  H -3.71083500 0.57989800 -4.25861900  H -2.50696400 -0.36566300 -3.35149800  H -1.89514800 2.07155700 -4.02711400  H -3.06813700 2.59827600 -2.82803500  C -1.26262300 1.69801200 -2.04060900  C -1.20277900 2.54234700 -0.91901600  H -0.32212700 1.32215100 -2.43980700  H -2.09712200 3.13054700 -0.70446600  C 0.04981100 3.12913700 -0.35836700  C 1.28877600 3.28973200 -1.19890700  C 0.45121500 4.49239900 -0.88524700  H 0.19787100 2.98794500 0.71005100  H 1.27403400 2.90569800 -2.21342300  H 2.24961100 3.20259800 -0.70843200  H 0.83028500 5.21647600 -0.17132800  H -0.15131000 4.91644700 -1.68413200  S -5.16496900 -1.17656100 -2.34311600  O -4.27662800 -2.33844500 -2.77699400  O -5.97509500 -1.20936900 -1.05884800  C -6.27869600 -0.72361400 -3.73409500  H -7.02535000 -1.51773400 -3.77815600  H -6.72416700 0.23712800 -3.47928500  H -5.68293600 -0.69116000 -4.64517900 |
| **TS2, P-*trans*-ene/Down *Re***  E_BS1_= -2546.338316  E_BS2_= -3270.163575  Frequency=-318.3 cm^-1^  X Y Z  Rh -1.9993305 0.5814702 -1.2513341  P 0.0135855 -0.4568698 -0.7223021  O 1.2934195 -0.2160418 -1.8100391  O 0.6760215 0.0417752 0.7587979  N -0.1450295 -2.1059238 -0.3232911  C 3.2519435 -0.4524208 -0.4551971  C 2.4923145 -0.8613558 -1.5275931  C 2.8719435 -1.9167998 -2.3814841  H 2.2523375 -2.1391958 -3.2435831  C 4.0164505 -2.6160668 -2.1114241  H 4.3352175 -3.4196428 -2.7690611  C 4.7782885 -2.3315848 -0.9478191  C 5.9119845 -3.1135228 -0.6041251  H 6.2042765 -3.9258878 -1.2638781  C 6.6187715 -2.8574098 0.5413079  H 7.4813015 -3.4636328 0.7990829  C 6.2176695 -1.8063908 1.3971929  H 6.7686905 -1.6209288 2.3137549  C 5.1377145 -1.0215778 1.0838619  H 4.8402595 -0.2210898 1.7518949  C 4.3912275 -1.2521428 -0.1024061  C 2.9167125 0.7938402 0.2900999  C 1.6863065 0.9846762 0.8774669  C 1.3884285 2.1027002 1.6851029  H 0.4030225 2.2030402 2.1254419  C 2.3322345 3.0713182 1.8644459  H 2.1074285 3.9398992 2.4757789  C 3.5830345 2.9925872 1.2002649  C 4.5251925 4.0486792 1.2958819  H 4.2880155 4.8995682 1.9287639  C 5.6995365 4.0083682 0.5914119  H 6.4132095 4.8227362 0.6652459  C 5.9778775 2.9038972 -0.2449251  H 6.8997525 2.8833102 -0.8178281  C 5.0948405 1.8584442 -0.3425281  H 5.3259355 1.0257592 -0.9968931  C 3.8747335 1.8623392 0.3864709  C 0.6553965 -2.7671408 0.7536789  H 1.4077575 -2.0376578 1.0477519  C 1.4082285 -3.9929168 0.2409909  H 2.0603955 -3.7112428 -0.5879591  H 0.7524425 -4.8010328 -0.0974281  H 2.0269125 -4.3924838 1.0497269  C -0.2299205 -3.0288048 1.9640279  C -1.0199115 -4.1744008 2.0724339  H -0.9456065 -4.9666168 1.3330549  C -1.9179735 -4.3251108 3.1260479  H -2.5297135 -5.2197918 3.1874669  C -2.0255085 -3.3380958 4.1003919  H -2.7224175 -3.4572798 4.9241199  C -1.2144255 -2.2074568 4.0243869  H -1.2639625 -1.4509308 4.8026809  C -0.3252935 -2.0552338 2.9646159  H 0.2994405 -1.1685448 2.9034289  C -1.1781005 -2.8913688 -1.0100981  H -1.0271305 -3.9146538 -0.6563621  C -0.9277215 -2.9181198 -2.5236931  H 0.0998975 -3.2450938 -2.7028661  H -1.0608135 -1.9282508 -2.9716471  H -1.5948865 -3.6147828 -3.0372471  C -2.6127745 -2.5451118 -0.6002201  C -3.6895915 -3.1253458 -1.2809161  H -3.5123045 -3.7588548 -2.1444301  C -4.9999585 -2.9295488 -0.8576441  H -5.8147965 -3.3975668 -1.4013991  C -5.2675415 -2.1635178 0.2767359  H -6.2869295 -2.0371868 0.6272659  C -4.2078195 -1.5892528 0.9670279  H -4.4063735 -1.0385618 1.8818889  C -2.8934175 -1.7657868 0.5284319  H -2.0755655 -1.3815088 1.1280529  C -2.7711815 2.4538282 -0.2443401  C -1.4827805 2.4282312 -0.5064701  C -0.3798725 3.3555522 -0.6408611  C -0.5049625 4.6449642 -0.0935351  C 0.7891075 3.0217062 -1.3390161  C 0.5390475 5.5507112 -0.2032131  H -1.4017035 4.9096532 0.4544819  C 1.8222885 3.9412312 -1.4611801  H 0.8945835 2.0438942 -1.7915051  C 1.7050275 5.2025002 -0.8849641  H 0.4403445 6.5349152 0.2434429  H 2.7284775 3.6636662 -1.9897941  H 2.5212145 5.9134842 -0.9656051  N -3.5942315 2.7785232 0.7897649  C -4.9764065 2.3115172 0.6484149  C -5.2146625 2.3978522 -0.8485011  H -5.6444935 2.9516782 1.2228279  H -5.0773365 1.2772982 1.0092339  H -6.1268445 1.8622572 -1.1261491  H -5.3348365 3.4475042 -1.1296141  C -4.0075575 1.8219662 -1.6036391  C -4.1275895 0.4083312 -2.0009801  H -3.6709525 2.4701622 -2.4111701  H -4.7698775 -0.2227638 -1.3910141  C -3.4130965 -0.1735478 -3.0224741  C -2.5449265 0.6156682 -3.9881121  C -1.4193445 1.1932512 -3.1411081  H -3.5764705 -1.2302608 -3.2073531  H -3.1282725 1.3918352 -4.4977831  H -2.1523315 -0.0523998 -4.7585721  H -0.4405805 0.7618212 -3.3477331  H -1.3593855 2.2834022 -3.1239191  S -2.9025545 2.6721752 2.4781819  O -1.7268615 3.6320322 2.4994029  O -4.1157925 2.8122932 3.3783819  C -2.2091995 0.9735552 2.6702339  H -3.0423605 0.2762792 2.6561449  H -1.4861785 0.8021992 1.8729569  H -1.7227765 0.9863602 3.6462869 | **TS2, P-*trans*-ene/Down *Si***  E_BS1_= -2546.345464  E_BS2_= -3270.160071  Frequency=-166.1 cm^-1^  X Y Z  Rh 1.9700485 0.4510895 -0.2269910  P -0.3684775 0.7709335 -0.4174430  O -1.0789355 -0.2531035 -1.5716470  O -1.2722575 0.3843865 0.9640170  N -0.9111095 2.3695215 -0.5809570  C -3.1558675 -0.9900555 -0.6297250  C -2.4604165 -0.3560935 -1.6363570  C -3.0855765 0.1841795 -2.7786600  H -2.4640605 0.6239095 -3.5517380  C -4.4474135 0.1274025 -2.8870000  H -4.9441025 0.5269235 -3.7665280  C -5.2327245 -0.4223675 -1.8406310  C -6.6502595 -0.4170065 -1.9121770  H -7.1249405 -0.0026475 -2.7973680  C -7.4066415 -0.9121525 -0.8831270  H -8.4900835 -0.8999075 -0.9451320  C -6.7731825 -1.4298065 0.2693650  H -7.3766355 -1.8017785 1.0914230  C -5.4053765 -1.4620055 0.3628750  H -4.9365355 -1.8568555 1.2570680  C -4.5906175 -0.9740435 -0.6942880  C -2.4289065 -1.6531205 0.4880940  C -1.5313005 -0.9441775 1.2537050  C -0.8915945 -1.4832035 2.3872060  H -0.2259815 -0.8474855 2.9617710  C -1.1361075 -2.7813125 2.7362870  H -0.6630945 -3.2125165 3.6137550  C -1.9792495 -3.5966545 1.9389590  C -2.1733475 -4.9675385 2.2478260  H -1.6967635 -5.3730585 3.1363190  C -2.9316175 -5.7707515 1.4372300  H -3.0726945 -6.8193675 1.6796260  C -3.5249705 -5.2331015 0.2724080  H -4.1094505 -5.8761095 -0.3783020  C -3.3752795 -3.9061295 -0.0400940  H -3.8359355 -3.5090255 -0.9376010  C -2.6126785 -3.0423165 0.7907080  C -2.2614225 2.8288965 -0.1385930  H -2.7880125 1.9295365 0.1831520  C -3.0601005 3.4381505 -1.2892280  H -3.2189325 2.6910055 -2.0698790  H -2.5704405 4.3072035 -1.7399400  H -4.0367095 3.7638385 -0.9191820  C -2.1161645 3.7373935 1.0764970  C -2.0335545 5.1269115 0.9658240  H -2.1536525 5.6109865 0.0007000  C -1.8167635 5.9201595 2.0911690  H -1.7513735 6.9984455 1.9833900  C -1.6938205 5.3346255 3.3462860  H -1.5295435 5.9526305 4.2234980  C -1.8004995 3.9509245 3.4726830  H -1.7275795 3.4861975 4.4514660  C -2.0095695 3.1614355 2.3473010  H -2.0858135 2.0831435 2.4484430  C 0.0076055 3.3697945 -1.1373610  H -0.4903295 4.3283685 -0.9585400  C 0.1596485 3.2148745 -2.6536050  H -0.8317545 3.1131275 -3.1016320  H 0.7416155 2.3259005 -2.9130470  H 0.6413835 4.0849715 -3.1081960  C 1.3209015 3.4784305 -0.3583440  C 2.4541555 4.0652255 -0.9373800  H 2.4531445 4.3356285 -1.9879200  C 3.5888535 4.3363435 -0.1788050  H 4.4516035 4.7953135 -0.6510480  C 3.6154555 4.0327665 1.1823450  H 4.4960235 4.2601345 1.7749120  C 2.5010785 3.4470525 1.7733830  H 2.4972335 3.2232065 2.8357860  C 1.3662365 3.1673005 1.0116350  H 0.4800065 2.7738795 1.5005200  C 3.3240815 -1.0726235 0.6081820  C 2.1423805 -1.4529015 0.2088450  C 1.4078435 -2.7029145 0.1211740  C 1.6862745 -3.7145035 1.0546300  C 0.5195125 -2.9556925 -0.9253430  C 1.1066035 -4.9677485 0.9171050  H 2.3522885 -3.5014285 1.8870060  C -0.0552905 -4.2160915 -1.0584900  H 0.2858585 -2.1704375 -1.6347900  C 0.2413845 -5.2236485 -0.1472090  H 1.3173885 -5.7441495 1.6464370  H -0.7430065 -4.4083515 -1.8751160  H -0.2177595 -6.2008755 -0.2511790  N 4.5003505 -1.5935295 1.0065280  C 5.4619665 -0.6494875 1.5852260  C 4.9631015 0.7771785 1.3217200  H 6.4394025 -0.8317615 1.1273710  H 5.5487855 -0.8499865 2.6562770  H 4.3104815 1.1182455 2.1304000  H 5.8198105 1.4600865 1.2795460  C 4.2203265 0.8418415 0.0039980  C 5.0337485 0.4998515 -1.1792450  H 3.7455615 1.8260975 -0.0974310  H 6.0395115 0.1258325 -0.9997270  C 4.6175735 0.6128325 -2.4459990  C 3.2201085 0.9986615 -2.8374310  C 2.1764875 0.0655755 -2.2381460  H 5.3124925 0.3562965 -3.2404890  H 3.1164835 0.9520475 -3.9295280  H 3.0157915 2.0422355 -2.5620570  H 1.1987365 0.1767765 -2.7116100  H 2.4987825 -0.9764745 -2.3110080  S 5.2644415 -2.7142265 -0.3553730  O 4.3017685 -2.7146705 -1.5340170  O 6.7285715 -2.3012235 -0.4936110  C 5.1826695 -4.3455965 0.4815660  H 5.6880675 -4.2340765 1.4400790  H 4.1232455 -4.5800855 0.5844760  H 5.6993975 -5.0495265 -0.1719350 |
| **TS3, P-*trans*-ene/Down *Re***  E_BS1_= -2546.346525  E_BS2_= -3270.159042  Frequency=-266.5 cm^-1^  X Y Z  Rh -1.9518074 0.5587721 -0.0760579  P 0.3770836 0.7822371 0.1985301  O 1.0205096 -0.2349019 1.4046451  O 1.3150446 0.3124731 -1.1371929  N 0.9650186 2.3732741 0.3063361  C 3.1460096 -0.9791219 0.5785441  C 2.3988126 -0.3231979 1.5336691  C 2.9689466 0.2573911 2.6852521  H 2.3099476 0.7146011 3.4159861  C 4.3251026 0.2185761 2.8556641  H 4.7788476 0.6483171 3.7441601  C 5.1617656 -0.3492189 1.8598251  C 6.5747246 -0.3219149 1.9906431  H 7.0065286 0.1222501 2.8833311  C 7.3805496 -0.8334129 1.0081541  H 8.4601246 -0.8034349 1.1149861  C 6.8031926 -1.3900049 -0.1556819  H 7.4461336 -1.7740179 -0.9414829  C 5.4411516 -1.4447609 -0.3052109  H 5.0153586 -1.8683739 -1.2077819  C 4.5759756 -0.9404009 0.7030471  C 2.4695476 -1.6937339 -0.5394299  C 1.5787366 -1.0282089 -1.3508539  C 0.9543496 -1.6281729 -2.4631279  H 0.2788006 -1.0296459 -3.0653869  C 1.2313886 -2.9316189 -2.7630549  H 0.7708036 -3.4066949 -3.6240709  C 2.0901896 -3.6954559 -1.9318179  C 2.3169506 -5.0719269 -2.1904139  H 1.8642236 -5.5155099 -3.0731549  C 3.0690476 -5.8339539 -1.3360849  H 3.2321306 -6.8878079 -1.5383749  C 3.6287196 -5.2455009 -0.1784049  H 4.2096766 -5.8556519 0.5061191  C 3.4493676 -3.9113489 0.0848041  H 3.8865426 -3.4762619 0.9764321  C 2.6880176 -3.0896379 -0.7904909  C 2.2931146 2.8099821 -0.2122179  H 2.7767646 1.9046501 -0.5796279  C 3.1817146 3.3874381 0.8869541  H 3.3729126 2.6286961 1.6488671  H 2.7433036 4.2628401 1.3769611  H 4.1395766 3.6936261 0.4562881  C 2.0762286 3.7349651 -1.4043979  C 2.1491986 5.1246361 -1.3001969  H 2.4324066 5.5917721 -0.3612459  C 1.8779066 5.9368101 -2.4008449  H 1.9409516 7.0159211 -2.2997389  C 1.5403676 5.3680451 -3.6233519  H 1.3345606 6.0002591 -4.4815069  C 1.4820826 3.9803511 -3.7438629  H 1.2405846 3.5266011 -4.7005269  C 1.7479636 3.1733631 -2.6438099  H 1.7037466 2.0918741 -2.7398759  C 0.1053326 3.3961041 0.9125801  H 0.5958426 4.3460981 0.6729161  C 0.0698366 3.2656551 2.4402681  H 1.0875346 3.1260521 2.8121061  H -0.5270204 2.4035771 2.7534961  H -0.3404784 4.1576441 2.9211821  C -1.2773694 3.4988391 0.2629021  C -2.3140814 4.1889051 0.9081761  H -2.1669204 4.5866251 1.9065061  C -3.5310474 4.4124191 0.2744451  H -4.3064774 4.9711431 0.7898931  C -3.7601694 3.9275091 -1.0137179  H -4.7115314 4.1049821 -1.5053369  C -2.7503004 3.2325131 -1.6645719  H -2.8944324 2.8729181 -2.6789309  C -1.5105024 3.0314391 -1.0408729  H -0.6874904 2.6252111 -1.6212899  C -3.5953234 -0.6790059 -0.4739179  C -2.4276344 -1.3185219 -0.4020049  C -1.8247384 -2.6209909 -0.5576389  C -2.3124424 -3.5021289 -1.5329619  C -0.7820674 -3.0235199 0.2849401  C -1.7841914 -4.7820389 -1.6391889  H -3.0748194 -3.1607769 -2.2282169  C -0.2795054 -4.3149819 0.1902951  H -0.3807774 -2.3231769 1.0105051  C -0.7822744 -5.1965979 -0.7634209  H -2.1547594 -5.4588179 -2.4030459  H 0.5281806 -4.6277719 0.8441151  H -0.3704304 -6.1972819 -0.8419699  N -4.8875504 -0.7655879 -0.9334759  C -5.5560544 0.5275111 -0.6857119  C -5.0919904 1.0937821 0.6829961  H -6.6358104 0.3887721 -0.7427449  H -5.2405894 1.1851401 -1.5002379  H -4.8258284 2.1485641 0.5959881  H -5.8930724 0.9973601 1.4228091  C -3.9153364 0.2633561 1.1408401  C -2.7383664 0.7818681 1.8287411  H -4.2150004 -0.7050179 1.5485191  H -2.7310744 1.8634611 1.9848311  C -2.0834864 0.0009711 2.9095721  C -2.9223564 -0.5771339 4.0401551  C -2.2415364 -1.4890729 3.0680141  H -1.1168884 0.3974401 3.2052931  H -4.0015684 -0.4759259 3.9630671  H -2.5313744 -0.4882869 5.0484331  H -1.3628334 -2.0304109 3.4035561  H -2.8577934 -2.0402009 2.3626751  S -5.8467674 -2.1599799 -0.0568239  O -4.9261694 -2.7383809 1.0200331  O -7.2127234 -1.5774329 0.2943941  C -6.0414514 -3.3908169 -1.4040659  H -6.4143974 -2.8517599 -2.2739899  H -5.0591764 -3.8325759 -1.5627339  H -6.7616434 -4.1234999 -1.0372879 | **TS3, P-*trans*-ene/Down *Si***  E_BS1_= -2546.334338  E_BS2_= -3270.143845  Frequency=-306.1 cm^-1^  X Y Z  Rh -2.1319406 0.5921054 -0.1584609  P 0.2000984 0.6379884 0.0494551  O 0.7587014 -0.2967896 1.3550261  O 1.1407354 0.0489084 -1.2325259  N 0.8451624 2.2160154 0.0390541  C 3.0044334 -0.8757956 0.7735941  C 2.1182474 -0.2603026 1.6277231  C 2.5192264 0.4137994 2.7993621  H 1.7548514 0.8261974 3.4490941  C 3.8517864 0.5161114 3.0885691  H 4.1806794 1.0163904 3.9949251  C 4.8258334 0.0084064 2.1888941  C 6.2115944 0.1928704 2.4337621  H 6.5160284 0.7096694 3.3398211  C 7.1479624 -0.2595026 1.5420611  H 8.2053144 -0.1085896 1.7342711  C 6.7346894 -0.9131756 0.3592291  H 7.4788504 -1.2505206 -0.3553029  C 5.4039144 -1.1216946 0.1017061  H 5.1054524 -1.6181796 -0.8143909  C 4.4076924 -0.6801236 1.0131771  C 2.5132324 -1.7233436 -0.3492489  C 1.6424924 -1.2356576 -1.2977709  C 1.2890794 -1.9605266 -2.4562189  H 0.6271204 -1.4929756 -3.1766259  C 1.7937604 -3.2132416 -2.6454919  H 1.5450864 -3.7740586 -3.5415219  C 2.5960174 -3.8252386 -1.6471609  C 3.0197884 -5.1724836 -1.7765889  H 2.7661334 -5.7120156 -2.6854749  C 3.7187504 -5.7895676 -0.7718869  H 4.0317304 -6.8235716 -0.8765519  C 4.0230434 -5.0774086 0.4097931  H 4.5569324 -5.5739996 1.2140351  C 3.6478174 -3.7652956 0.5529401  H 3.8853534 -3.2371396 1.4695021  C 2.9325764 -3.0933936 -0.4744559  C 2.1627644 2.5712454 -0.5646439  H 2.5988704 1.6290194 -0.8927439  C 3.1301004 3.1826524 0.4453871  H 3.3184174 2.4757554 1.2562271  H 2.7624974 4.1164614 0.8828461  H 4.0806874 3.3995934 -0.0506989  C 1.9179954 3.4187724 -1.8079809  C 2.0683954 4.8058044 -1.8167989  H 2.4307874 5.3240924 -0.9337399  C 1.7755374 5.5473314 -2.9612839  H 1.9003344 6.6257804 -2.9485769  C 1.3367924 4.9090174 -4.1152599  H 1.1147394 5.4851534 -5.0081309  C 1.1983924 3.5214344 -4.1232879  H 0.8756834 3.0123714 -5.0267629  C 1.4869614 2.7856784 -2.9801589  H 1.3806884 1.7040634 -2.9881999  C 0.0646604 3.3085534 0.6340571  H 0.5357704 4.2212494 0.2504431  C 0.1941714 3.3161134 2.1587461  H 1.2478504 3.2238764 2.4292151  H -0.3420946 2.4700404 2.5938191  H -0.1834646 4.2419954 2.6020981  C -1.3773536 3.3854704 0.1183971  C -2.3802906 4.0239454 0.8636081  H -2.1761426 4.3614564 1.8736711  C -3.6362396 4.2717194 0.3194071  H -4.3847056 4.7837674 0.9163161  C -3.9303776 3.8855134 -0.9890229  H -4.9061876 4.0928334 -1.4164119  C -2.9534306 3.2502034 -1.7455079  H -3.1513536 2.9660104 -2.7741969  C -1.6870546 3.0025994 -1.2002939  H -0.8981906 2.6158204 -1.8410319  C -3.7895406 -0.7897306 -0.6686519  C -2.5514916 -1.2372426 -0.6722969  C -1.8378256 -2.4684276 -0.9683329  C -2.0899016 -3.1592606 -2.1606529  C -0.9635016 -3.0116336 -0.0196209  C -1.5020166 -4.4021766 -2.3785059  H -2.7510636 -2.7226746 -2.9047539  C -0.3870216 -4.2548516 -0.2416659  H -0.7558716 -2.4614406 0.8924261  C -0.6629416 -4.9555746 -1.4143429  H -1.6976056 -4.9352716 -3.3040949  H 0.2924354 -4.6729576 0.4939791  H -0.2006666 -5.9227466 -1.5846319  N -5.0471986 -1.2953796 -0.8229669  C -6.0643416 -0.3332266 -0.4065789  C -5.5700306 0.2313884 0.9267051  H -7.0312936 -0.8291336 -0.3147549  H -6.1231466 0.4350264 -1.1837099  H -6.1729376 1.0885404 1.2418531  H -5.6600626 -0.5360866 1.7006501  C -4.1299166 0.6912404 0.7756961  C -3.0549996 0.2933724 1.6929161  H -4.1249786 1.7541694 0.5106931  H -3.0682486 -0.7735526 1.9268361  C -2.6231746 1.1126224 2.8396411  C -3.0196436 0.5746154 4.2127521  C -1.5909966 0.5387574 3.7854301  H -2.6573156 2.1893674 2.7137061  H -3.5726826 -0.3591576 4.2472211  H -3.3209646 1.3078224 4.9536571  H -0.9034606 1.2321074 4.2597561  H -1.1492736 -0.4144366 3.5112101  S -5.2725406 -2.8758486 0.2949781  O -4.0805676 -2.9431496 1.2485301  O -6.7040496 -2.8168876 0.8227761  C -5.1613926 -4.2140196 -0.9564559  H -5.8895946 -3.9691266 -1.7285879  H -4.1361976 -4.2191316 -1.3253289  H -5.4174196 -5.1351736 -0.4313049 |
| **TS2, P-*trans*-ene/Up *Re***  E_BS1_= -2546.355527  E_BS2_= -3270.156482  Frequency=-194.0 cm^-1^  X Y Z  Rh -2.1974719 -0.5128507 -0.0257764  P -0.0516559 0.5170553 0.2730106  O 0.8623851 -0.3260857 1.4497986  O 1.0778171 0.6034503 -0.9899854  N -0.1239689 2.1762953 0.6046356  C 3.1561031 0.0351873 0.8629726  C 2.1219361 0.1590983 1.7656296  C 2.2848941 0.7459273 3.0376096  H 1.4375201 0.7623033 3.7147306  C 3.4997221 1.2706863 3.3837476  H 3.6453571 1.7166803 4.3635226  C 4.5727951 1.2824773 2.4547646  C 5.8078131 1.9072393 2.7697236  H 5.9276061 2.3608103 3.7498266  C 6.8253271 1.9518933 1.8535786  H 7.7638401 2.4377123 2.1003476  C 6.6482921 1.3738623 0.5759166  H 7.4496311 1.4292813 -0.1541144  C 5.4728621 0.7492153 0.2464236  H 5.3498971 0.3169663 -0.7403284  C 4.4027711 0.6729583 1.1780146  C 2.9632161 -0.7478937 -0.3888524  C 1.9379791 -0.4461367 -1.2564604  C 1.7543741 -1.1111847 -2.4866934  H 0.9551001 -0.7825207 -3.1413084  C 2.5967271 -2.1282407 -2.8329684  H 2.4736641 -2.6434917 -3.7809354  C 3.6205681 -2.5486427 -1.9458004  C 4.4334681 -3.6697097 -2.2540454  H 4.2920881 -4.1666017 -3.2100914  C 5.3630751 -4.1316697 -1.3597234  H 5.9752001 -4.9949557 -1.6009984  C 5.5206211 -3.4829027 -0.1135854  H 6.2472111 -3.8590067 0.5999236  C 4.7659321 -2.3813607 0.2030236  H 4.8977651 -1.9001227 1.1652696  C 3.7983761 -1.8724787 -0.7057994  C 0.9543461 3.1497933 0.2671696  H 1.7789401 2.5510143 -0.1203884  C 1.4759971 3.9025923 1.4887076  H 1.9108671 3.2014163 2.2036206  H 0.7009231 4.4788943 2.0035196  H 2.2547441 4.6031863 1.1732996  C 0.4603731 4.0416863 -0.8639354  C -0.1257649 5.2875793 -0.6373434  H -0.1593359 5.7069183 0.3641286  C -0.6729399 6.0188753 -1.6912334  H -1.1308119 6.9835593 -1.4956264  C -0.6283529 5.5188663 -2.9878454  H -1.0515839 6.0897733 -3.8084414  C -0.0215909 4.2867993 -3.2292814  H 0.0376741 3.8972213 -4.2413534  C 0.5166561 3.5566243 -2.1757764  H 0.9760971 2.5904903 -2.3628064  C -1.3182219 2.6634493 1.3149486  H -1.2393789 3.7542333 1.2618476  C -1.2934939 2.2687113 2.7927686  H -0.3199849 2.5310563 3.2140116  H -1.4516409 1.1934383 2.9258316  H -2.0606569 2.8034833 3.3608826  C -2.6296339 2.3532733 0.5843676  C -3.7885509 1.9798843 1.2805176  H -3.7278269 1.6928893 2.3237896  C -5.0350049 2.0058833 0.6548786  H -5.9234439 1.7432893 1.2211446  C -5.1441449 2.3803403 -0.6801384  H -6.1168609 2.4175923 -1.1604194  C -3.9941519 2.7192003 -1.3948084  H -4.0672449 3.0209423 -2.4352534  C -2.7530959 2.7024573 -0.7730154  H -1.8697449 3.0039793 -1.3271514  C -2.9397979 -2.5575027 0.3624586  C -1.7206339 -2.4658367 -0.0466334  C -0.6296579 -3.3355777 -0.4442994  C 0.6103061 -3.2996707 0.2051036  C -0.8685099 -4.2799767 -1.4524524  C 1.5849931 -4.2324017 -0.1265144  H 0.7995161 -2.5483927 0.9644686  C 0.1278601 -5.1836127 -1.8011714  H -1.8322189 -4.2905367 -1.9524284  C 1.3486051 -5.1684317 -1.1306974  H 2.5423851 -4.2164567 0.3840356  H -0.0557239 -5.9107437 -2.5858164  H 2.1238511 -5.8804537 -1.3948454  N -3.7331619 -3.0218387 1.3529586  C -5.1669419 -2.7942507 1.1430306  C -5.3443279 -1.3959357 0.5538896  H -5.6862819 -2.9214097 2.0951266  H -5.4975209 -3.5845597 0.4633406  H -6.3548869 -1.3000137 0.1399326  H -5.2475289 -0.6476527 1.3467426  C -4.3296969 -1.1239827 -0.5382524  C -4.4852569 -1.9014227 -1.7855814  H -4.3045119 -0.0428817 -0.7414244  H -5.0976839 -2.8007697 -1.7537044  C -3.8820539 -1.5477347 -2.9249234  C -1.8009899 -0.3681047 -2.0537764  C -3.0053229 -0.3336087 -2.9955004  H -4.0193469 -2.1490867 -3.8193634  H -1.1426099 -1.2062617 -2.3019954  H -1.2383289 0.5636403 -2.1763364  H -3.5969529 0.5673693 -2.7911644  H -2.6124139 -0.2138007 -4.0133204  S -3.1746689 -2.0999547 2.9135076  O -2.5542059 -0.7598597 2.4374466  O -4.3376869 -2.0859397 3.8938786  C -1.8106549 -3.1945237 3.4691166  H -1.3444229 -2.6973487 4.3202056  H -2.2722099 -4.1429057 3.7415436  H -1.1233039 -3.2902047 2.6277986 | **TS2, P-*trans*-ene/Up *Si***  E_BS1_= -2546.331488  E_BS2_= -3270.141763  Frequency=-224.8cm^-1^  X Y Z  Rh -1.6212600 -0.6017165 0.1987007  P 0.7665780 -0.7394195 -0.4150463  O 0.6046070 0.5921255 -1.5060583  O 1.7376790 0.0173055 0.7744527  N 1.8933290 -1.8537465 -0.9913933  C 2.1607960 2.2786325 -0.8072433  C 1.6713740 1.4285115 -1.7769413  C 2.2036390 1.3845065 -3.0832293  H 1.7256990 0.7417795 -3.8149363  C 3.2913120 2.1518875 -3.3941723  H 3.7094540 2.1293715 -4.3965203  C 3.9084810 2.9660225 -2.4086063  C 5.0836990 3.7062045 -2.7007873  H 5.4970340 3.6527895 -3.7043393  C 5.6956350 4.4620145 -1.7357243  H 6.5981150 5.0189155 -1.9665783  C 5.1577630 4.5042005 -0.4293943  H 5.6577580 5.0869075 0.3380347  C 4.0170060 3.8092065 -0.1187503  H 3.6248370 3.8434595 0.8916367  C 3.3479780 3.0295625 -1.1006473  C 1.4672970 2.3716885 0.5051697  C 1.2755750 1.2303015 1.2528667  C 0.6611770 1.2461885 2.5239227  H 0.5693370 0.3138985 3.0696037  C 0.2259770 2.4327365 3.0455937  H -0.2334300 2.4633805 4.0292917  C 0.3576040 3.6380815 2.3093827  C -0.1209010 4.8683315 2.8309827  H -0.5664310 4.8737085 3.8219937  C -0.0362470 6.0238035 2.0994887  H -0.4013360 6.9602405 2.5095797  C 0.5203320 5.9928245 0.7996487  H 0.5702120 6.9064295 0.2152617  C 1.0009990 4.8208745 0.2714447  H 1.4248190 4.8147665 -0.7263443  C 0.9565050 3.6106625 1.0172637  C 3.3342530 -1.5187505 -1.1880673  H 3.4222570 -0.4521055 -0.9697493  C 3.7596490 -1.7223045 -2.6414463  H 3.1581630 -1.0915195 -3.2992183  H 3.6558470 -2.7587195 -2.9769633  H 4.8102080 -1.4403815 -2.7562403  C 4.1952770 -2.2565745 -0.1727823  C 4.7226130 -3.5262605 -0.4157063  H 4.5919900 -4.0036905 -1.3828073  C 5.4383280 -4.1991215 0.5722137  H 5.8384200 -5.1874405 0.3679307  C 5.6480140 -3.6047785 1.8121327  H 6.2078600 -4.1291145 2.5804057  C 5.1469760 -2.3281335 2.0566047  H 5.3199610 -1.8489325 3.0155537  C 4.4282590 -1.6613285 1.0708727  H 4.0241450 -0.6729955 1.2674187  C 1.4304540 -3.2170345 -1.3032493  H 2.3377460 -3.7487015 -1.6046193  C 0.4911400 -3.2113735 -2.5148163  H 0.9488640 -2.6296765 -3.3192873  H -0.4766490 -2.7548765 -2.2754503  H 0.3090140 -4.2198215 -2.8952303  C 0.9111880 -3.9710125 -0.0779783  C 0.0489910 -5.0632905 -0.2201293  H -0.3251060 -5.3458235 -1.1989953  C -0.3388910 -5.8183035 0.8842867  H -1.0065800 -6.6635325 0.7469737  C 0.1331190 -5.4986055 2.1542747  H -0.1601450 -6.0932555 3.0139257  C 0.9904520 -4.4125255 2.3079237  H 1.3784440 -4.1569505 3.2893577  C 1.3752720 -3.6570615 1.2036727  H 2.0686120 -2.8339885 1.3410107  C -4.5919510 -0.3795655 0.1442047  C -3.4279300 0.1462345 0.5192017  C -3.4534340 1.5625405 0.9516867  C -4.3789100 2.0421135 1.8862997  C -2.5223510 2.4477555 0.3845537  C -4.3751550 3.3862955 2.2447777  H -5.0879990 1.3552655 2.3390607  C -2.5359240 3.7920765 0.7374647  H -1.8002350 2.0962185 -0.3515053  C -3.4585880 4.2629225 1.6689347  H -5.0903230 3.7481045 2.9770447  H -1.8113410 4.4694535 0.2970377  H -3.4516110 5.3107255 1.9514437  N -5.6432680 -0.1331105 -0.5688873  C -6.7522720 -1.0803065 -0.5760903  C -6.1534290 -2.4909445 -0.8091683  H -7.4597330 -0.8088805 -1.3610993  H -7.2460020 -1.0040145 0.3963117  H -6.8956250 -3.2421095 -0.5252993  H -5.9428670 -2.6059125 -1.8773073  C -4.9005350 -2.6212195 -0.0005113  C -3.6617540 -2.4974515 -0.5482413  H -5.0091730 -2.8464105 1.0572847  H -3.5968170 -2.3467435 -1.6264803  C -2.3721390 -2.5125415 0.1655327  C -1.4974740 -1.4579065 2.0398727  C -2.3026040 -2.7047285 1.6804707  H -1.6311040 -3.1044815 -0.3719613  H -1.9449780 -0.7999495 2.7870107  H -0.4466350 -1.6579545 2.2566037  H -1.8056750 -3.6386205 1.9555717  H -3.2877240 -2.6804975 2.1534847  S -5.2068350 0.6183515 -2.3106263  O -3.6987960 0.4536545 -2.4465563  O -6.1970600 -0.0026485 -3.2865843  C -5.5978610 2.3892835 -1.9959103  H -5.3893680 2.9210795 -2.9253003  H -6.6534790 2.4320405 -1.7295093  H -4.9421880 2.7034345 -1.1822423 |
| **TS3, P-*trans*-ene/Up *Re***  E_BS1_= -2546.355092  E_BS2_= -3270.163856  Frequency=-248.9 cm^-1^  X Y Z  Rh 2.0609405 -0.4048841 -0.2119492  P -0.0579165 0.6628089 -0.4769922  O -0.9852625 -0.2502441 -1.5959732  O -1.1571145 0.7110899 0.8193598  N -0.0660235 2.3043979 -0.8961322  C -3.2224645 -0.3585341 -0.7425522  C -2.3394065 -0.0267891 -1.7492572  C -2.7667795 0.4926269 -2.9898692  H -2.0231335 0.6697659 -3.7600422  C -4.0952175 0.7435349 -3.1941892  H -4.4392355 1.1333969 -4.1480782  C -5.0395685 0.5302819 -2.1565342  C -6.4089775 0.8581769 -2.3332652  H -6.7284945 1.2630269 -3.2897332  C -7.3104315 0.6797499 -1.3173052  H -8.3553945 0.9350119 -1.4608762  C -6.8750205 0.1702979 -0.0725752  H -7.5894115 0.0499159 0.7358848  C -5.5608255 -0.1675621 0.1253628  H -5.2411935 -0.5508381 1.0882898  C -4.6040835 -0.0132031 -0.9138192  C -2.7180195 -1.0640761 0.4663198  C -1.6929855 -0.5123031 1.2003348  C -1.1699765 -1.1191291 2.3601768  H -0.3989385 -0.6037581 2.9215298  C -1.6642955 -2.3256441 2.7686518  H -1.2797745 -2.8016321 3.6662388  C -2.6849535 -2.9745541 2.0286838  C -3.1794905 -4.2420021 2.4306988  H -2.7707295 -4.6984101 3.3282138  C -4.1513345 -4.8798921 1.7062948  H -4.5299355 -5.8456181 2.0263778  C -4.6570575 -4.2795241 0.5312018  H -5.4142185 -4.7947501 -0.0519502  C -4.2000565 -3.0540541 0.1178908  H -4.5932375 -2.6146811 -0.7913922  C -3.2154715 -2.3506991 0.8635488  C -1.2262625 3.2139549 -0.6974292  H -1.9927345 2.6033009 -0.2156152  C -1.8158255 3.7396719 -2.0057452  H -2.1850485 2.9132819 -2.6155062  H -1.0989315 4.3137439 -2.6007772  H -2.6575385 4.4002999 -1.7778302  C -0.8385505 4.3129909 0.2823178  C -0.3107435 5.5380329 -0.1330102  H -0.2200505 5.7687869 -1.1906462  C 0.0980065 6.4898399 0.7997128  H 0.5092115 7.4349689 0.4583438  C -0.0333705 6.2354689 2.1614788  H 0.2770105 6.9797469 2.8881438  C -0.5853845 5.0281829 2.5856468  H -0.7121305 4.8293049 3.6456948  C -0.9818925 4.0770959 1.6514548  H -1.4051725 3.1330109 1.9840548  C 1.0618855 2.7536969 -1.7405242  H 0.9251405 3.8340579 -1.8319802  C 0.9986385 2.1560389 -3.1494452  H 0.0417265 2.4067229 -3.6134572  H 1.0794525 1.0633059 -3.1352742  H 1.8032535 2.5503909 -3.7776092  C 2.4168325 2.5779069 -1.0563392  C 3.3464925 1.6038489 -1.4532022  H 3.1412615 0.9377199 -2.2878962  C 4.6317885 1.5728899 -0.8906882  H 5.3419185 0.8239429 -1.2297582  C 4.9965305 2.5121179 0.0633738  H 5.9988295 2.5062369 0.4804158  C 4.0717745 3.4800549 0.4682918  H 4.3549915 4.2250019 1.2059828  C 2.7938535 3.5020509 -0.0723542  H 2.0821135 4.2605699 0.2433568  C 2.9839425 -2.3036851 -0.1776382  C 1.6831135 -2.3618361 -0.2052612  C 0.6154625 -3.3374391 -0.2429982  C 0.6509145 -4.4098371 0.6621498  C -0.4120465 -3.2615761 -1.1895622  C -0.3300975 -5.3902431 0.6163678  H 1.4490275 -4.4640311 1.3968948  C -1.3781825 -4.2582191 -1.2405022  H -0.4411025 -2.4277891 -1.8802212  C -1.3416335 -5.3188001 -0.3394892  H -0.3059535 -6.2126351 1.3242008  H -2.1692445 -4.2011761 -1.9805862  H -2.1091245 -6.0845391 -0.3733512  N 4.1403075 -3.0146551 -0.3736242  C 4.9652995 -3.0391231 0.8503018  C 4.2055965 -2.2336771 1.9107808  H 5.9402945 -2.5861531 0.6408718  H 5.1121605 -4.0770921 1.1567688  H 3.4151825 -2.8377741 2.3662018  H 4.8927275 -1.9244941 2.7066368  C 3.6251575 -0.9786141 1.2835388  C 2.4430415 -0.3354601 1.8272218  H 4.4087515 -0.2951871 0.9506888  H 1.7512705 -0.9913351 2.3590888  C 2.5550365 1.0367379 2.3634768  C 1.3429365 1.8530889 2.7387578  C 2.1958605 1.2817359 3.8221758  H 3.4001955 1.5985049 1.9830788  H 0.3636125 1.4239979 2.5614718  H 1.4011495 2.9172429 2.5371878  H 2.8430065 1.9431709 4.3890208  H 1.8083315 0.4386349 4.3878948  S 5.0396835 -2.2628121 -1.8384072  O 3.9925365 -1.4759811 -2.6251972  O 6.2998985 -1.5673561 -1.3188162  C 5.4882865 -3.7805221 -2.7628722  H 6.0108645 -3.4541371 -3.6626172  H 6.1316855 -4.3701541 -2.1101472  H 4.5504655 -4.2863131 -2.9892442 | **TS3, P-*trans*-ene/Up *Si***  E_BS1_= -2546.352095  E_BS2_= -3270.153612  Frequency=-222.5 cm^-1^  X Y Z  Rh 2.0809541 -0.4606280 -0.3148346  P -0.0760779 0.5485950 -0.5646586  O -1.0373069 -0.3241490 -1.6962336  O -1.1029179 0.4847570 0.7897884  N -0.1875609 2.2159840 -0.8664036  C -3.2755359 -0.2381600 -0.8516796  C -2.3649169 0.0330780 -1.8511866  C -2.7373459 0.6402450 -3.0690476  H -1.9834219 0.7657800 -3.8384876  C -4.0322459 1.0417830 -3.2524256  H -4.3360019 1.4999970 -4.1894086  C -4.9877909 0.9024710 -2.2120586  C -6.3106529 1.3957150 -2.3577526  H -6.5878569 1.8749100 -3.2927726  C -7.2184979 1.2809050 -1.3381516  H -8.2258839 1.6669590 -1.4567036  C -6.8388609 0.6672260 -0.1222416  H -7.5582479 0.5932030 0.6873304  C -5.5721379 0.1695760 0.0453384  H -5.2928639 -0.2942240 0.9852864  C -4.6100409 0.2639950 -0.9959736  C -2.8511569 -1.0712970 0.3053374  C -1.7571389 -0.7079070 1.0572924  C -1.2808699 -1.4817720 2.1335714  H -0.4048509 -1.1348280 2.6657674  C -1.9255139 -2.6393930 2.4683594  H -1.5728389 -3.2433760 3.2994184  C -3.0362699 -3.0910300 1.7118054  C -3.6616439 -4.3311600 2.0041274  H -3.3118329 -4.8990870 2.8621064  C -4.6681459 -4.8155330 1.2112394  H -5.1370899 -5.7676540 1.4388334  C -5.0914529 -4.0746670 0.0837014  H -5.8756909 -4.4692290 -0.5548976  C -4.5228869 -2.8620230 -0.2111066  H -4.8590309 -2.3117370 -1.0821106  C -3.4879089 -2.3224070 0.6013954  C -1.2884999 3.0915530 -0.3822696  H -1.9948639 2.4243920 0.1130534  C -2.0425589 3.7898290 -1.5120616  H -2.5099659 3.0504360 -2.1646376  H -1.4033019 4.4317690 -2.1265596  H -2.8288669 4.4192170 -1.0847496  C -0.7294229 4.0344590 0.6758244  C -0.3246289 5.3388900 0.3898294  H -0.4732329 5.7559810 -0.6020346  C 0.2680091 6.1320630 1.3731004  H 0.5764351 7.1451400 1.1333394  C 0.4527341 5.6324030 2.6579274  H 0.9094281 6.2516300 3.4237424  C 0.0237231 4.3409210 2.9616024  H 0.1354231 3.9531980 3.9703844  C -0.5647759 3.5536170 1.9789524  H -0.9105369 2.5504960 2.2159144  C 0.8282471 2.7906150 -1.7716136  H 0.6683391 3.8705270 -1.7171296  C 0.6067451 2.3603430 -3.2236976  H -0.3980329 2.6503770 -3.5409196  H 0.6903491 1.2746690 -3.3436286  H 1.3342581 2.8369820 -3.8878676  C 2.2602771 2.5789640 -1.2837476  C 3.1213081 1.6245440 -1.8498556  H 2.7882271 0.9803310 -2.6579866  C 4.4725301 1.5760300 -1.4804476  H 5.1278711 0.8515130 -1.9552916  C 4.9685931 2.4640880 -0.5340466  H 6.0204721 2.4437150 -0.2656946  C 4.1071431 3.3869790 0.0643114  H 4.4857431 4.0822600 0.8073684  C 2.7666541 3.4391930 -0.3024306  H 2.1081331 4.1716860 0.1566634  C 3.1264411 -2.2308030 0.0350994  C 1.8349931 -2.4354310 0.0016204  C 0.8476011 -3.4802840 0.0481144  C 1.1041571 -4.6120680 0.8407214  C -0.3388669 -3.4027310 -0.6949876  C 0.1824981 -5.6483280 0.8887144  H 2.0246201 -4.6638320 1.4148724  C -1.2449219 -4.4532330 -0.6556846  H -0.5423999 -2.5212990 -1.2938716  C -0.9896179 -5.5703580 0.1376244  H 0.3788281 -6.5189830 1.5063754  H -2.1684129 -4.3910760 -1.2216976  H -1.7151479 -6.3763670 0.1782224  N 4.3634991 -2.7407070 -0.2374166  C 5.3880871 -2.2466690 0.6956844  C 5.0312381 -0.8246180 1.1444194  H 6.3694651 -2.2902720 0.2206094  H 5.3817781 -2.9415030 1.5422154  H 5.5909941 -0.5835520 2.0557404  H 5.3005371 -0.0939100 0.3788904  C 3.5574311 -0.7687760 1.4434164  C 2.7728991 0.4379460 1.4253144  H 3.2404601 -1.4878030 2.1990774  H 3.2672281 1.2999690 0.9726954  C 1.8676791 0.8610000 2.5219774  C 1.5150761 0.0270780 3.7224714  C 2.5017571 1.1468530 3.8711314  H 1.1311811 1.5908000 2.2003904  H 1.8797111 -0.9936410 3.7973424  H 0.5272231 0.1721600 4.1484534  H 2.1956331 2.0502830 4.3883094  H 3.5488901 0.8861680 3.9995504  S 4.8038471 -2.2598620 -2.0412276  O 3.5747801 -1.5788520 -2.6520166  O 6.1567531 -1.5453120 -2.0188126  C 4.9746311 -3.9371440 -2.7655426  H 5.1965361 -3.7977210 -3.8240446  H 5.7914691 -4.4250950 -2.2349296  H 4.0190201 -4.4357530 -2.6067256 |
| **TS2, P-*trans*-yne/Down *Re***  E_BS1_= -2546.312986  E_BS2_= -3270.136515  Frequency=-365.2 cm^-1^  X Y Z  Rh -1.5921687 -0.2275994 0.4248758  P 0.7476393 0.5923326 0.4304608  O 1.6788463 -0.2933934 1.5576318  O 1.7061573 0.4020846 -0.9725912  N 0.9550393 2.2615586 0.6234428  C 3.7731373 -0.7802164 0.5247728  C 3.0577333 -0.2033354 1.5499988  C 3.6750293 0.4522726 2.6352888  H 3.0492593 0.8424286 3.4311258  C 5.0382513 0.5610506 2.6636788  H 5.5306973 1.0515096 3.4986068  C 5.8287073 0.0562736 1.5981058  C 7.2387123 0.2179396 1.5924448  H 7.7086453 0.7198606 2.4338528  C 7.9938213 -0.2407684 0.5454218  H 9.0710303 -0.1086874 0.5480378  C 7.3659993 -0.8780714 -0.5488812  H 7.9664723 -1.2229194 -1.3848882  C 6.0067953 -1.0598344 -0.5684032  H 5.5403563 -1.5419814 -1.4203752  C 5.1968723 -0.6114624 0.5093318  C 3.0531723 -1.5291484 -0.5412012  C 2.0809243 -0.8957014 -1.2821792  C 1.4469523 -1.5046974 -2.3858652  H 0.7594073 -0.9113574 -2.9805072  C 1.7310953 -2.8070414 -2.6916432  H 1.2564503 -3.2885454 -3.5419722  C 2.6395113 -3.5526264 -1.8959102  C 2.8884483 -4.9247474 -2.1590252  H 2.3808233 -5.3956004 -2.9965422  C 3.7468443 -5.6451634 -1.3711252  H 3.9289233 -6.6950364 -1.5769712  C 4.3915713 -5.0214414 -0.2784832  H 5.0566733 -5.6018754 0.3530658  C 4.1819863 -3.6939684 -0.0057972  H 4.6778063 -3.2312684 0.8400858  C 3.3093133 -2.9145544 -0.8115402  C 2.1099073 3.0347956 0.0931558  H 2.7621963 2.2985336 -0.3790312  C 2.9196693 3.7181966 1.1929828  H 3.3343273 2.9685606 1.8714048  H 2.3305633 4.4273056 1.7828358  H 3.7501103 4.2694146 0.7420248  C 1.6050453 3.9685746 -0.9994772  C 1.3248683 5.3156546 -0.7703062  H 1.5278913 5.7628246 0.1985048  C 0.7956183 6.1143486 -1.7837182  H 0.5850683 7.1612836 -1.5883772  C 0.5492183 5.5762836 -3.0415122  H 0.1448543 6.2009226 -3.8319912  C 0.8389053 4.2344086 -3.2854602  H 0.6684983 3.8109976 -4.2710632  C 1.3613063 3.4393506 -2.2721372  H 1.5852093 2.3925316 -2.4601462  C -0.0431727 2.9601046 1.4522968  H 0.1516503 4.0252966 1.2843218  C 0.1687633 2.6620856 2.9405568  H 1.2254163 2.7807586 3.1893338  H -0.1220747 1.6319686 3.1807058  H -0.4032387 3.3420996 3.5780428  C -1.4770777 2.7262646 0.9705158  C -2.5532217 2.7065846 1.8616788  H -2.3823197 2.7901206 2.9293808  C -3.8649717 2.5895916 1.3991098  H -4.6842567 2.5643586 2.1103578  C -4.1241677 2.4886906 0.0397898  H -5.1448857 2.3775906 -0.3065662  C -3.0599617 2.5031836 -0.8607692  H -3.2453577 2.4248596 -1.9275482  C -1.7491037 2.6195836 -0.4050432  H -0.9308807 2.6789796 -1.1179212  C -3.3753147 -0.8347194 -0.5791632  C -3.5287037 -0.8242374 0.7070628  C -4.1959917 -1.2700504 1.8874498  C -3.9532917 -0.6423034 3.1202008  C -5.0813767 -2.3611774 1.8217338  C -4.5805357 -1.1015504 4.2694928  H -3.2765117 0.2058936 3.1555878  C -5.7134397 -2.8045064 2.9730148  H -5.2731637 -2.8344934 0.8637738  C -5.4608257 -2.1802814 4.1955808  H -4.3933377 -0.6164744 5.2219858  H -6.4060157 -3.6381584 2.9213698  H -5.9562077 -2.5347584 5.0940788  N -4.0892077 -0.4560844 -1.7083002  C -3.6349807 -1.0945714 -2.9515682  C -2.1322757 -1.1650884 -2.7856642  H -3.9179897 -0.4728914 -3.8045942  H -4.0799127 -2.0906514 -3.0682342  H -1.6874727 -1.8347234 -3.5299282  H -1.6937897 -0.1698724 -2.9151022  C -1.8004567 -1.6857154 -1.3974882  C -2.0933497 -3.1054104 -1.0962412  H -0.7366467 -1.5023204 -1.2320302  H -2.8804077 -3.6009434 -1.6602032  C -1.4232557 -3.7535784 -0.1390122  C -1.0281377 -1.8928164 1.5012798  C -0.4119547 -3.0602504 0.7356228  H -1.6485557 -4.7982064 0.0569638  H -1.8988477 -2.2330684 2.0642428  H -0.2983767 -1.4458344 2.1864268  H 0.4585603 -2.7299264 0.1546418  H -0.0210517 -3.7673044 1.4777108  S -5.8882697 -0.6349694 -1.4558332  O -6.2354437 -2.1021854 -1.6805112  O -6.2248577 0.0672946 -0.1527162  C -6.5232387 0.3751286 -2.8546852  H -6.0173817 1.3387666 -2.8031122  H -6.3244387 -0.1637914 -3.7798452  H -7.5948747 0.4734526 -2.6762432 | **TS2, P-*trans*-yne/Down *Si***  E_BS1_= -2546.338057  E_BS2_= -3270.151447  Frequency=-396.9 cm^-1^  X Y Z  Rh -1.5949935 0.3225509 0.8144306  P 0.7596645 0.8753299 0.5703416  O 1.6858135 0.0654689 1.7454636  O 1.5571475 0.3142439 -0.8446374  N 1.2146315 2.5052489 0.5956726  C 3.2770585 -1.3269961 0.5736466  C 2.9790115 -0.3851561 1.5364646  C 3.9557085 0.1391889 2.4116266  H 3.6411015 0.8486559 3.1697016  C 5.2568475 -0.2580641 2.2891396  H 6.0136315 0.1316769 2.9638146  C 5.6437895 -1.1634301 1.2672796  C 7.0032985 -1.5297081 1.0922766  H 7.7434455 -1.1250401 1.7771796  C 7.3796825 -2.3675981 0.0759086  H 8.4226635 -2.6375101 -0.0542974  C 6.4061105 -2.8683401 -0.8170964  H 6.7100495 -3.5150551 -1.6343064  C 5.0822895 -2.5418501 -0.6665494  H 4.3530845 -2.9267331 -1.3704684  C 4.6550695 -1.6922501 0.3893506  C 2.2091585 -1.8989681 -0.2917014  C 1.4185895 -1.0434891 -1.0258864  C 0.4391905 -1.4929951 -1.9379104  H -0.1087385 -0.7500571 -2.5107014  C 0.2293925 -2.8375001 -2.0802354  H -0.4918885 -3.2096991 -2.8043694  C 0.9427145 -3.7720531 -1.2820554  C 0.6714235 -5.1628801 -1.3634554  H -0.0694735 -5.5096801 -2.0795104  C 1.3296345 -6.0547911 -0.5580034  H 1.1171525 -7.1167221 -0.6285904  C 2.2880435 -5.5899161 0.3712356  H 2.7949175 -6.2984041 1.0186376  C 2.5851845 -4.2540571 0.4623046  H 3.3213365 -3.9113161 1.1807196  C 1.9343485 -3.3048491 -0.3699874  C 2.6350495 2.9404429 0.6100936  H 3.2183985 2.0416349 0.8225706  C 2.8767995 3.9145079 1.7614276  H 2.5930545 3.4451559 2.7078826  H 2.2974405 4.8371769 1.6560266  H 3.9349605 4.1888309 1.8035566  C 3.0916165 3.4478249 -0.7510864  C 3.0046945 4.7938459 -1.1151434  H 2.6655215 5.5372749 -0.3991224  C 3.3637355 5.2100419 -2.3957174  H 3.2842975 6.2595819 -2.6615704  C 3.8302735 4.2857899 -3.3245234  H 4.1129865 4.6095309 -4.3214654  C 3.9479045 2.9449609 -2.9625034  H 4.3296055 2.2198439 -3.6748934  C 3.5830625 2.5318109 -1.6859214  H 3.6640785 1.4838159 -1.4134134  C 0.1515905 3.4729419 0.2303256  H 0.6737425 4.3367289 -0.1910434  C -0.6299055 3.9310189 1.4600476  H 0.0513335 4.3889439 2.1807236  H -1.1149525 3.0852999 1.9600296  H -1.3965785 4.6649419 1.1912526  C -0.7023385 2.8860069 -0.8922794  C -2.0327505 2.4898719 -0.6906204  H -2.5567575 2.7697059 0.2192576  C -2.7457655 1.8354659 -1.7057194  H -3.7823545 1.5580059 -1.5461934  C -2.1363615 1.5838559 -2.9302694  H -2.6983645 1.0960159 -3.7211324  C -0.8255975 2.0104079 -3.1479264  H -0.3512105 1.8446339 -4.1105414  C -0.1143315 2.6498179 -2.1387934  H 0.9173575 2.9428369 -2.3087574  C -4.0937885 -1.2278271 0.5765566  C -3.5995335 -0.0265651 0.8918196  C -4.5428025 1.1066189 1.0995796  C -4.1802755 2.1041809 2.0216686  C -5.7783105 1.2319309 0.4446196  C -5.0158095 3.1843679 2.2791596  H -3.2354855 2.0293469 2.5480216  C -6.6097005 2.3153059 0.7002716  H -6.0753655 0.5039039 -0.2997264  C -6.2352175 3.2942139 1.6177906  H -4.7134435 3.9371589 3.0003186  H -7.5543915 2.3975449 0.1723996  H -6.8898175 4.1374299 1.8140836  N -4.9872085 -1.8887561 -0.1114724  C -5.4257295 -3.1834341 0.3976146  C -4.1465955 -3.9192491 0.8561606  H -5.9774985 -3.7143591 -0.3804444  H -6.0993725 -2.9877141 1.2355746  H -4.4082945 -4.7011011 1.5737846  H -3.6740805 -4.4061091 -0.0044554  C -3.2168255 -2.9091951 1.4645046  C -2.0560495 -2.5219391 0.8487866  H -3.3875155 -2.6377971 2.5028296  H -1.8513315 -2.9141481 -0.1463294  C -1.0475305 -1.6176511 1.3804416  C -1.4176915 0.1168909 2.8626766  C -0.9458805 -1.3300191 2.8757506  H -0.0870755 -1.8039641 0.9066756  H -2.3928575 0.2652879 3.3243086  H -0.6925135 0.8496069 3.2234846  H 0.0894425 -1.4245561 3.2083796  H -1.5675605 -1.9842481 3.4958096  S -4.9727545 -1.7084281 -2.0033544  O -5.8573025 -2.8365761 -2.4987204  O -5.2482635 -0.2434191 -2.2854234  C -3.2201965 -2.0528821 -2.4559244  H -2.5932135 -1.3565601 -1.8988284  H -3.0290925 -3.0957801 -2.2073164  H -3.1593985 -1.8773371 -3.5310674 |
| **TS3, P-*trans*-yne/Down *Re***  E_BS1_= -2546.323489  E_BS2_= -3270.143737  Frequency=-350.9 cm^-1^  X Y Z  Rh -1.6866401 0.1858909 0.7081573  P 0.6764069 0.8359059 0.5792453  O 1.8237709 0.2058109 1.6785483  O 1.4291049 0.3481859 -0.8819867  N 0.8402109 2.5058669 0.4164833  C 3.6075109 -0.7852671 0.4108623  C 3.1693799 0.1003589 1.3727413  C 4.0528239 0.8925649 2.1358563  H 3.6409409 1.5293739 2.9112163  C 5.3968389 0.8202629 1.8990403  H 6.0884949 1.4159589 2.4877753  C 5.9072979 -0.0167211 0.8730453  C 7.2968409 -0.0549781 0.5871003  H 7.9677589 0.5530919 1.1877413  C 7.7835089 -0.8330821 -0.4296987  H 8.8475399 -0.8547931 -0.6421567  C 6.8931899 -1.6002841 -1.2140927  H 7.2792719 -2.1980511 -2.0338527  C 5.5461669 -1.5930031 -0.9556827  H 4.8798139 -2.1815071 -1.5760657  C 5.0107969 -0.8163771 0.1072493  C 2.6278979 -1.6308931 -0.3237467  C 1.5901519 -1.0186401 -0.9918627  C 0.6751059 -1.7243251 -1.8009287  H -0.1063871 -1.1657351 -2.3055117  C 0.7985599 -3.0815991 -1.9169517  H 0.1277029 -3.6422801 -2.5631627  C 1.7910509 -3.7864691 -1.1856397  C 1.8772909 -5.2025171 -1.2399967  H 1.1882549 -5.7441301 -1.8833497  C 2.8082039 -5.8785931 -0.4961207  H 2.8671079 -6.9612041 -0.5451017  C 3.6931829 -5.1638901 0.3437213  H 4.4164479 -5.7060231 0.9445743  C 3.6457289 -3.7950581 0.4069333  H 4.3291429 -3.2581621 1.0554063  C 2.7049559 -3.0620331 -0.3653937  C 2.0708199 3.1762719 -0.0721767  H 2.7930639 2.3731679 -0.2458777  C 2.6585799 4.1028129 0.9904103  H 2.9034239 3.5306769 1.8889323  H 1.9675479 4.9014659 1.2777963  H 3.5735389 4.5692069 0.6141853  C 1.8077889 3.8440409 -1.4161117  C 1.5029749 5.2012929 -1.5321597  H 1.4971889 5.8423219 -0.6550897  C 1.2209879 5.7637279 -2.7759267  H 0.9888429 6.8218169 -2.8476397  C 1.2488459 4.9763279 -3.9215367  H 1.0364349 5.4163509 -4.8908207  C 1.5652709 3.6229619 -3.8185497  H 1.6047069 3.0043829 -4.7103157  C 1.8421399 3.0634499 -2.5766757  H 2.0829619 2.0072129 -2.4958227  C -0.3517501 3.3054679 0.7363553  H -0.1457121 4.2987009 0.3215613  C -0.5428291 3.4462999 2.2500773  H 0.4087459 3.7120259 2.7156213  H -0.8825601 2.5010639 2.6926333  H -1.2685111 4.2246469 2.5018923  C -1.5998631 2.8087359 -0.0078007  C -2.8892281 3.0801119 0.4792553  H -3.0180951 3.5315549 1.4572713  C -4.0177171 2.8333489 -0.3041327  H -5.0010931 3.0650219 0.0911763  C -3.8899171 2.2642929 -1.5635747  H -4.7678601 2.0367759 -2.1576547  C -2.6199461 1.9644749 -2.0532237  H -2.5127491 1.5104259 -3.0314987  C -1.4869191 2.2442019 -1.2963247  H -0.5032111 2.0718239 -1.7211897  C -2.7584941 -1.3548521 -0.2539887  C -3.5516211 -0.4855031 0.3186413  C -4.9066511 -0.2414301 0.7368993  C -5.1646161 0.6980999 1.7477313  C -5.9708451 -0.9881111 0.2015053  C -6.4577011 0.8920599 2.2135623  H -4.3376281 1.2660699 2.1619953  C -7.2649251 -0.7675921 0.6528703  H -5.7869631 -1.7285011 -0.5714447  C -7.5109291 0.1677079 1.6577313  H -6.6478111 1.6129439 3.0024463  H -8.0853271 -1.3332621 0.2233523  H -8.5245471 0.3284679 2.0114003  N -2.6206931 -2.3220091 -1.2342757  C -2.4930711 -3.6806001 -0.6455777  C -2.3451411 -3.4366931 0.8541243  H -1.6062471 -4.1636341 -1.0647527  H -3.3832971 -4.2759261 -0.8690277  H -3.3242341 -3.3657111 1.3360993  H -1.7758801 -4.2400841 1.3342863  C -1.5758031 -2.1250481 1.0281023  C -1.6970641 -1.2757091 2.1951553  H -0.5581601 -2.2278831 0.6462683  H -2.6822191 -1.2807271 2.6710853  C -0.5770131 -1.0805491 3.1380513  C -0.6795931 0.0081909 4.1882283  C -0.7794111 -1.4241591 4.6029283  H 0.4145299 -1.2601701 2.7364953  H -1.5903731 0.6015879 4.2110303  H 0.2290779 0.5570719 4.4128463  H 0.0624799 -1.8761081 5.1171263  H -1.7510211 -1.8016051 4.9078063  S -3.7592021 -2.1927141 -2.6232097  O -4.9537841 -3.1040211 -2.3548267  O -3.9424771 -0.7087051 -2.8916657  C -2.7286991 -2.9405951 -3.9448837  H -1.8250441 -2.3367081 -4.0181457  H -2.5258111 -3.9719321 -3.6563317  H -3.3251221 -2.8957751 -4.8570937 | **TS3, P-*trans*-yne/Down *Si***  E_BS1_= -2546.323489  E_BS2_= -3270.149003  Frequency=-373.0cm^-1^  X Y Z  Rh -1.5266309 -0.1364365 -0.2276688  P 0.6599341 0.7685915 0.2546282  O 1.5809591 -0.0784725 1.4100782  O 1.7096081 0.6908305 -1.1018258  N 0.7376831 2.4411305 0.5046982  C 3.6391551 -0.7064655 0.3671322  C 2.9612031 -0.0819525 1.3914862  C 3.6195651 0.5093755 2.4902552  H 3.0184971 0.9412525 3.2835742  C 4.9856881 0.4993225 2.5387312  H 5.5061091 0.9403375 3.3840712  C 5.7449051 -0.0721735 1.4842492  C 7.1635861 -0.0445365 1.5080142  H 7.6606111 0.4078255 2.3618252  C 7.8936291 -0.5687485 0.4741152  H 8.9782411 -0.5405535 0.4998582  C 7.2303781 -1.1375385 -0.6363448  H 7.8117171 -1.5346715 -1.4626068  C 5.8604911 -1.1871285 -0.6854158  H 5.3711301 -1.6181305 -1.5516128  C 5.0738441 -0.6704785 0.3789832  C 2.8874741 -1.3519615 -0.7447318  C 2.0110251 -0.6004005 -1.4949308  C 1.3801301 -1.0952185 -2.6568548  H 0.7585561 -0.4199575 -3.2371308  C 1.5659861 -2.4027605 -3.0157318  H 1.0960631 -2.8001125 -3.9112918  C 2.3457251 -3.2692955 -2.2037268  C 2.4518181 -4.6544915 -2.4966858  H 1.9491411 -5.0422015 -3.3787578  C 3.1686521 -5.4906365 -1.6809688  H 3.2401061 -6.5492715 -1.9089508  C 3.8156841 -4.9749145 -0.5345818  H 4.3713031 -5.6442365 0.1143202  C 3.7490841 -3.6385625 -0.2352018  H 4.2444691 -3.2548855 0.6497842  C 3.0166941 -2.7448195 -1.0608448  C 1.9179091 3.2712925 0.1445582  H 2.6280201 2.5830555 -0.3178228  C 2.6093331 3.8859245 1.3594342  H 2.9647501 3.0957725 2.0260652  H 1.9568011 4.5525055 1.9316962  H 3.4742101 4.4693355 1.0297942  C 1.4956941 4.2736275 -0.9224878  C 1.2382801 5.6145405 -0.6386138  H 1.3893861 6.0033865 0.3641832  C 0.7987381 6.4816565 -1.6386518  H 0.6045751 7.5229215 -1.4006648  C 0.6184821 6.0179715 -2.9363928  H 0.2817711 6.6949305 -3.7153098  C 0.8848191 4.6818855 -3.2335888  H 0.7625371 4.3154685 -4.2487088  C 1.3189821 3.8193595 -2.2345318  H 1.5288841 2.7776945 -2.4642258  C -0.3764119 3.0727945 1.2304072  H -0.2216239 4.1491415 1.0881332  C -0.2998369 2.7727925 2.7319532  H 0.7081111 2.9805205 3.0964242  H -0.5110999 1.7166985 2.9328252  H -0.9950529 3.3871425 3.3105282  C -1.7399229 2.7701465 0.5999292  C -2.9125729 2.7804755 1.3602562  H -2.8692189 2.9195225 2.4345062  C -4.1570089 2.6044955 0.7608162  H -5.0514399 2.5888155 1.3747802  C -4.2606929 2.4420925 -0.6167538  H -5.2348869 2.3037015 -1.0717008  C -3.1066219 2.4427485 -1.3922708  H -3.1688029 2.3227345 -2.4694978  C -1.8555369 2.5941005 -0.7918728  H -0.9653379 2.6593515 -1.4120708  C -3.4273359 -0.7928735 -0.5768108  C -3.3297019 -0.5298605 0.7028032  C -3.6991739 -0.8307255 2.0457322  C -3.0389799 -0.2093895 3.1186202  C -4.6903209 -1.7989315 2.3026622  C -3.3650469 -0.5410165 4.4243912  H -2.2708899 0.5227585 2.9009002  C -5.0198189 -2.1134655 3.6124482  H -5.1949439 -2.2889375 1.4757862  C -4.3592809 -1.4882965 4.6707982  H -2.8513369 -0.0630255 5.2521742  H -5.7891409 -2.8522105 3.8113642  H -4.6185289 -1.7426465 5.6938802  N -4.2635339 -0.8116765 -1.6795788  C -3.8212829 -1.7463275 -2.7474258  C -2.3694149 -2.1492195 -2.4730158  H -3.9344399 -1.2591505 -3.7185658  H -4.4525189 -2.6431115 -2.7171998  H -2.1619759 -3.1183295 -2.9406088  H -1.6614119 -1.4209115 -2.8781308  C -2.2209709 -2.2613635 -0.9760828  C -0.9710899 -2.1677235 -0.2677038  H -2.9541279 -2.9315315 -0.5189168  H -0.0790889 -2.1706645 -0.8944058  C -0.8267419 -2.9118625 1.0111212  C -0.0284179 -4.2084115 0.9896422  C 0.5009931 -3.0086865 1.7093762  H -1.7100189 -2.9066005 1.6459702  H 0.4566261 -4.4846625 0.0562802  H -0.4155069 -5.0435855 1.5646152  H 0.4921231 -3.0013835 2.7943902  H 1.3391311 -2.4868005 1.2642842  S -5.9957089 -1.1022745 -1.1777608  O -6.1669649 -2.6025195 -0.9418288  O -6.2809519 -0.0991925 -0.0731168  C -6.8808749 -0.6106975 -2.7092908  H -6.5888599 0.4167905 -2.9229858  H -6.5943209 -1.3059635 -3.4976288  H -7.9435829 -0.6917815 -2.4772508 |
| **TS2, P-*trans*-yne/Up *Re***  E_BS1_= -2546.334741  E_BS2_= -3270.136242  Frequency=-264.1 cm^-1^  X Y Z  Rh -1.8849635 -1.3465957 -0.9600639  P -0.1196365 0.4614973 -0.4923949  O 0.5894245 -0.0237097 0.9711921  O 1.3117045 0.6660573 -1.4512289  N -0.6063845 2.0966483 -0.3203519  C 2.9688425 -0.2101697 0.6241081  C 1.8943205 0.2473843 1.3556621  C 2.0416565 0.9416763 2.5762761  H 1.1480905 1.2219113 3.1211391  C 3.2943285 1.2169713 3.0438771  H 3.4217875 1.7366093 3.9890111  C 4.4448915 0.8489813 2.2985241  C 5.7467645 1.1854633 2.7510121  H 5.8490905 1.7099973 3.6970661  C 6.8532565 0.8652023 2.0091941  H 7.8451695 1.1286193 2.3622611  C 6.6995285 0.2034713 0.7707181  H 7.5760665 -0.0267587 0.1730161  C 5.4546435 -0.1458137 0.3122811  H 5.3576945 -0.6408047 -0.6471849  C 4.2886215 0.1480683 1.0692511  C 2.7741685 -1.0121877 -0.6146969  C 2.0147935 -0.5007377 -1.6452419  C 1.9094585 -1.1367787 -2.9017949  H 1.3275625 -0.6521797 -3.6788189  C 2.5276185 -2.3407767 -3.1003019  H 2.4665965 -2.8385457 -4.0642359  C 3.2248255 -2.9787217 -2.0386159  C 3.7822225 -4.2742267 -2.2003549  H 3.6964115 -4.7612657 -3.1679769  C 4.4083135 -4.9045837 -1.1573259  H 4.8306095 -5.8954317 -1.2907009  C 4.5023785 -4.2638307 0.0995531  H 4.9888145 -4.7720877 0.9263471  C 3.9891705 -3.0053547 0.2837281  H 4.0724235 -2.5221707 1.2510611  C 3.3446085 -2.3188627 -0.7809199  C 0.2258425 3.0389003 0.4912921  H 1.1038565 2.4744813 0.8116861  C -0.5249265 3.4382833 1.7591511  H -0.7890885 2.5365263 2.3154891  H -1.4500025 3.9806033 1.5433441  H 0.1079565 4.0729263 2.3883341  C 0.7740575 4.1935193 -0.3373469  C 0.3400525 5.5084913 -0.1721899  H -0.4036405 5.7494063 0.5813651  C 0.8639775 6.5344233 -0.9576309  H 0.5157235 7.5526043 -0.8131419  C 1.8304995 6.2565273 -1.9173139  H 2.2387025 7.0554553 -2.5286079  C 2.2793175 4.9468723 -2.0827539  H 3.0439125 4.7224303 -2.8204689  C 1.7578985 3.9265373 -1.2965459  H 2.1067635 2.9052633 -1.4263699  C -1.3784895 2.6564723 -1.4599479  H -0.6947695 3.2824433 -2.0437679  C -2.5404845 3.5422883 -1.0102119  H -2.1649365 4.4403363 -0.5162589  H -3.2111925 3.0216543 -0.3225699  H -3.1144365 3.8619263 -1.8859699  C -1.8257855 1.5397113 -2.3812979  C -2.9048785 0.6883823 -2.0215839  H -3.5429435 0.9480083 -1.1838079  C -3.2688075 -0.3625697 -2.8701909  H -4.1951775 -0.8898227 -2.6869579  C -2.5699015 -0.5798897 -4.0792319  H -2.8849915 -1.3770087 -4.7446929  C -1.5301335 0.2540903 -4.4220239  H -1.0023025 0.1128503 -5.3601149  C -1.1602495 1.3118363 -3.5695359  H -0.3307705 1.9573903 -3.8438899  C -2.0318925 -1.9594057 1.3253131  C -2.8742435 -1.1280817 0.7784471  C -4.1229515 -0.4557397 1.0956681  C -4.1402805 0.9331443 1.2872411  C -5.3152175 -1.1844967 1.1939731  C -5.3360935 1.5796973 1.5720011  H -3.2025365 1.4774433 1.2434781  C -6.5108775 -0.5274257 1.4683861  H -5.2987435 -2.2605767 1.0485631  C -6.5236185 0.8529253 1.6553511  H -5.3422915 2.6535603 1.7319441  H -7.4336695 -1.0944997 1.5392301  H -7.4579795 1.3617053 1.8701801  N -1.5679925 -2.2125487 2.5798421  C -0.5161035 -3.2280867 2.6678031  C 0.1702765 -3.2277977 1.3127241  H -0.9904065 -4.1918657 2.8821121  H 0.1617135 -2.9770807 3.4862621  H 0.8610895 -2.3849577 1.2308121  H 0.7334475 -4.1518037 1.1462121  C -0.9216105 -3.1184987 0.2614441  C -0.3621315 -2.9675777 -1.1181369  H -1.6254345 -3.9508207 0.3110281  H 0.6732885 -2.6543197 -1.1727929  C -1.0368305 -3.2267797 -2.2778439  C -2.4676575 -3.7327487 -2.3183599  C -3.2457165 -2.8989667 -1.3022589  H -0.4851875 -3.1179657 -3.2079589  H -2.5100115 -4.8133277 -2.1242689  H -2.8637405 -3.5802227 -3.3259739  S -1.3146315 -0.7324357 3.6807031  O 0.0024265 -0.9419467 4.4197481  O -1.5962995 0.5387423 2.8925331  C -2.7221125 -1.0016907 4.8257301  H -2.6868255 -0.1943277 5.5579781  H -3.6227395 -0.9596377 4.2123861  H -2.5752625 -1.9776607 5.2867951  H -4.2056405 -2.5376217 -1.6687929  H -3.4310365 -3.4146137 -0.3568149 | **TS2, P-*trans*-yne/Up *Si***  E_BS1_= -2546.320291  E_BS2_= -3270.120513  Frequency=-342.5 cm^-1^  X Y Z  Rh 1.9643229 -0.4122646 -0.0489605  P -0.3160071 0.5690544 -0.2751175  O -1.1813101 -0.5048996 -1.3019815  O -1.3835811 0.5935194 1.0726195  N -0.5224171 2.1756994 -0.7642475  C -3.4348611 -0.5598496 -0.5471795  C -2.5217901 -0.2729726 -1.5350175  C -2.8981301 0.2209254 -2.8015215  H -2.1274411 0.3855614 -3.5466055  C -4.2195261 0.4689204 -3.0549565  H -4.5303981 0.8407654 -4.0273645  C -5.2014231 0.2752554 -2.0477335  C -6.5650701 0.5895744 -2.2844015  H -6.8501281 0.9689584 -3.2619875  C -7.5038081 0.4287434 -1.2996585  H -8.5440621 0.6731164 -1.4890215  C -7.1145711 -0.0479846 -0.0270655  H -7.8592011 -0.1548556 0.7555115  C -5.8068371 -0.3707656 0.2303575  H -5.5238571 -0.7270216 1.2147465  C -4.8121181 -0.2328246 -0.7744345  C -2.9629331 -1.1895936 0.7156755  C -2.0012481 -0.5741226 1.4885295  C -1.6437561 -1.0602036 2.7667905  H -0.9556851 -0.4832546 3.3730915  C -2.1874681 -2.2243336 3.2316215  H -1.9227821 -2.5963556 4.2171175  C -3.0860541 -2.9717716 2.4266735  C -3.5927261 -4.2241806 2.8611075  H -3.2950801 -4.5968086 3.8375525  C -4.4357651 -4.9537376 2.0648055  H -4.8193171 -5.9104266 2.4046855  C -4.8039941 -4.4595066 0.7928385  H -5.4601101 -5.0478446 0.1590005  C -4.3404001 -3.2466286 0.3502065  H -4.6304391 -2.8836526 -0.6293555  C -3.4758651 -2.4576056 1.1566725  C -1.6181791 3.0720604 -0.3162715  H -2.2787901 2.4497894 0.2887945  C -2.4387361 3.6099284 -1.4851155  H -2.9039371 2.7801324 -2.0217645  H -1.8376341 4.1846354 -2.1974515  H -3.2296671 4.2665404 -1.1100915  C -1.0130171 4.1324534 0.5977205  C -0.8519611 5.4624344 0.2130015  H -1.2368701 5.8084584 -0.7416555  C -0.1952171 6.3694344 1.0473305  H -0.0743821 7.4008284 0.7305115  C 0.2997639 5.9555224 2.2785475  H 0.8094159 6.6610464 2.9273925  C 0.1245309 4.6307504 2.6810715  H 0.4940549 4.3027904 3.6483435  C -0.5248631 3.7311334 1.8468745  H -0.6540881 2.6977264 2.1578725  C 0.5103139 2.7311184 -1.6700245  H 0.3003539 3.8034834 -1.7049495  C 0.3621079 2.1707334 -3.0857515  H -0.6383351 2.4003324 -3.4628545  H 0.4868699 1.0824174 -3.1079505  H 1.1001219 2.6159124 -3.7606395  C 1.9210779 2.6077574 -1.1004575  C 2.8011019 1.5801114 -1.5004165  H 2.5283329 0.9051074 -2.3079635  C 4.1127859 1.5403974 -1.0051035  H 4.7923169 0.7731254 -1.3575515  C 4.5489909 2.4911004 -0.0894195  H 5.5635529 2.4525514 0.2934435  C 3.6744469 3.4941554 0.3189975  H 4.0053519 4.2493204 1.0255145  C 2.3738559 3.5553534 -0.1852155  H 1.7132019 4.3571044 0.1314265  C 2.5022849 -2.4593106 -0.1428995  C 3.5678459 -1.7085056 -0.1199055  C 5.0118929 -1.6119996 -0.0243825  C 5.8149299 -1.5731416 -1.1713655  C 5.5999459 -1.5103756 1.2432985  C 7.1916919 -1.4272856 -1.0416855  H 5.3516959 -1.6760006 -2.1465765  C 6.9773419 -1.3590696 1.3590685  H 4.9741109 -1.5627136 2.1291675  C 7.7749209 -1.3119706 0.2182705  H 7.8117729 -1.4068756 -1.9324765  H 7.4286559 -1.2832126 2.3433735  H 8.8496939 -1.1937066 0.3119455  N 1.9144199 -3.3792966 -1.0055765  C 0.4828699 -3.5700226 -0.6736845  C 0.4412999 -3.4733816 0.8344305  H 0.1496249 -4.5433496 -1.0385605  H -0.1317841 -2.7682536 -1.1070245  H -0.5934521 -3.4225096 1.1862365  H 0.9108209 -4.3613676 1.2698075  C 1.2072289 -2.2195816 1.2795165  C 2.0003319 -2.3778076 2.5229625  H 0.4687009 -1.4279416 1.4223095  H 2.3366279 -3.3844196 2.7619985  C 2.3021809 -1.3585156 3.3303535  C 1.9554029 0.0624794 2.9857675  C 2.5750449 0.5005824 1.6713935  H 2.8661779 -1.5487786 4.2396355  H 2.3256959 0.7394744 3.7663895  H 0.8709879 0.2166504 2.9429765  S 2.0797279 -2.6209046 -2.6907895  O 1.4387079 -1.2193956 -2.5796385  O 3.5329159 -2.7405276 -3.1126605  C 1.0237419 -3.6916636 -3.7420655  H 1.2203099 -3.3769386 -4.7676615  H 1.3481439 -4.7181736 -3.5703545  H -0.0128911 -3.5155606 -3.4591885  H 2.3069599 1.5368734 1.4367645  H 3.6623919 0.3941244 1.6916375 |
| **TS3, P-*trans*-yne/Up *Re***  E_BS1_= -2546.334131  E_BS2_= -3270.148855  Frequency=-420.4 cm^-1^  X Y Z  Rh -1.5427634 -0.2615195 -0.2060699  P 0.6286916 0.6917705 0.0812361  O 1.4463846 -0.0853565 1.3747111  O 1.8319116 0.5734815 -1.1230959  N 0.6050276 2.3688015 0.2471161  C 3.7033096 -0.4422025 0.6802791  C 2.8030216 0.1179205 1.5596451  C 3.2009496 0.8726995 2.6823521  H 2.4367036 1.2551865 3.3510911  C 4.5335186 1.0873095 2.9051651  H 4.8591576 1.6566605 3.7711511  C 5.5095216 0.5888885 2.0030721  C 6.8905166 0.8489745 2.2026661  H 7.1919406 1.4215255 3.0756911  C 7.8261426 0.3961925 1.3103641  H 8.8797416 0.6012905 1.4703561  C 7.4172786 -0.3317105 0.1697221  H 8.1605096 -0.6705575 -0.5451419  C 6.0916846 -0.6082225 -0.0467259  H 5.7943276 -1.1596195 -0.9317869  C 5.0981776 -0.1713605 0.8702061  C 3.2011676 -1.2645495 -0.4541759  C 2.3120586 -0.7067945 -1.3464169  C 1.8581896 -1.3800845 -2.4990039  H 1.2002376 -0.8533385 -3.1830349  C 2.2581976 -2.6688205 -2.7217299  H 1.9228736 -3.2022785 -3.6067259  C 3.0965276 -3.3363095 -1.7909049  C 3.4575516 -4.6965085 -1.9761079  H 3.0964746 -5.2152705 -2.8601119  C 4.2405566 -5.3457115 -1.0585009  H 4.5088426 -6.3866995 -1.2075899  C 4.6951906 -4.6596675 0.0910721  H 5.3050356 -5.1831035 0.8207731  C 4.3738676 -3.3420365 0.2936191  H 4.7279706 -2.8286355 1.1808041  C 3.5735926 -2.6358875 -0.6446359  C 1.7376226 3.2654095 -0.1055409  H 2.5467166 2.6026975 -0.4180429  C 2.2426676 4.0683885 1.0909261  H 2.6097346 3.3898885 1.8654481  H 1.4730316 4.7098085 1.5311651  H 3.0701666 4.7105935 0.7750121  C 1.3356136 4.1014045 -1.3139529  C 0.8473256 5.4035385 -1.1999879  H 0.8060846 5.8933445 -0.2313429  C 0.4180716 6.1031975 -2.3272759  H 0.0377396 7.1143245 -2.2191369  C 0.4821006 5.5119805 -3.5837759  H 0.1528266 6.0587935 -4.4618569  C 0.9844706 4.2177035 -3.7111739  H 1.0573516 3.7558545 -4.6913929  C 1.4056066 3.5199545 -2.5850899  H 1.7978156 2.5111645 -2.6847299  C -0.6266834 2.9558225 0.8017121  H -0.5486754 4.0251065 0.5731961  C -0.6902864 2.7911005 2.3222931  H 0.2765616 3.0515265 2.7583711  H -0.9306414 1.7596785 2.5960131  H -1.4492724 3.4357785 2.7723301  C -1.8907674 2.4888935 0.0707391  C -3.1364834 2.4985695 0.7072551  H -3.2052814 2.6695645 1.7753551  C -4.3117144 2.2865505 -0.0125089  H -5.2593074 2.2871935 0.5139511  C -4.2694224 2.0603075 -1.3832679  H -5.1873464 1.9023395 -1.9394089  C -3.0379304 2.0003785 -2.0286509  H -2.9848154 1.8235425 -3.0987249  C -1.8553314 2.2046235 -1.3116659  H -0.9099694 2.2661915 -1.8458379  C -2.9157704 -1.1200145 1.1157941  C -3.5194514 -0.9175715 -0.0175039  C -4.6708404 -1.1927665 -0.8265459  C -5.8602914 -1.6498645 -0.2346129  C -4.6024654 -1.0615305 -2.2223319  C -6.9582354 -1.9532145 -1.0278329  H -5.9193934 -1.7257135 0.8454651  C -5.6969594 -1.3867085 -3.0108659  H -3.6788434 -0.7127505 -2.6721609  C -6.8778064 -1.8274575 -2.4144639  H -7.8799534 -2.2913315 -0.5654439  H -5.6341624 -1.2909225 -4.0902429  H -7.7372684 -2.0726505 -3.0306319  N -2.9937654 -1.2563555 2.4921701  C -1.6516474 -1.3365235 3.1029691  C -0.8430284 -2.3226265 2.2441141  H -1.7483414 -1.6710195 4.1380031  H -1.1716864 -0.3523695 3.0947991  H 0.1819506 -1.9589825 2.1456221  H -0.8264714 -3.3149475 2.7055361  C -1.4931954 -2.4517765 0.8814261  C -0.7807804 -2.2188245 -0.3368619  H -2.2771744 -3.2074525 0.8460651  H 0.2967556 -2.1010515 -0.2177869  C -1.1746864 -2.8601735 -1.6202349  C -1.1765524 -4.3760305 -1.7275849  C -2.4621254 -3.6144555 -1.8278899  H -0.7759284 -2.3477995 -2.4913469  H -0.9873904 -4.9344835 -0.8146829  H -0.7515094 -4.8192135 -2.6221469  H -2.9366434 -3.5175305 -2.7990549  H -3.1752344 -3.6882835 -1.0122239  S -4.0521424 0.0061095 3.2919141  O -3.1823344 1.1478865 3.8121981  O -5.1968184 0.2576565 2.3207241  C -4.6779164 -0.9680465 4.7170341  H -5.3865364 -0.3186865 5.2326291  H -5.1596284 -1.8524635 4.3029211  H -3.8273984 -1.2097275 5.3533301 | **TS3, P-*trans*-yne/Up *Si***  E_BS1_= -2546.327176  E_BS2_= -3270.137005  Frequency=-276.7 cm^-1^  X Y Z  Rh -1.8584439 -0.4592112 -0.5750993  P 0.4511701 0.1975028 -0.0694303  O 1.2717611 -0.9116162 0.9284767  O 1.6627961 0.5627828 -1.2160003  N 0.3786351 1.7529368 0.6134467  C 3.5854241 -0.3501242 0.7210607  C 2.4626541 -0.4986112 1.5040307  C 2.4559551 -0.2551662 2.8927977  H 1.5414021 -0.4386842 3.4469367  C 3.5969641 0.1984728 3.4953667  H 3.6170971 0.3781998 4.5665467  C 4.7552291 0.4874258 2.7265417  C 5.9122661 1.0484098 3.3273057  H 5.9054101 1.2324938 4.3982437  C 7.0121481 1.3638798 2.5738397  H 7.8900621 1.7980898 3.0412517  C 6.9996661 1.1340848 1.1791067  H 7.8662921 1.4036678 0.5835037  C 5.9033901 0.5785258 0.5708937  H 5.9064871 0.4130858 -0.5008943  C 4.7519671 0.2273748 1.3254337  C 3.5741391 -0.8034502 -0.6988263  C 2.6398861 -0.3319142 -1.5951563  C 2.6582621 -0.6750662 -2.9650643  H 1.9256351 -0.2165922 -3.6214923  C 3.5954441 -1.5525762 -3.4314733  H 3.6290401 -1.8116442 -4.4859243  C 4.5262731 -2.1519432 -2.5425033  C 5.4593391 -3.1159252 -3.0051163  H 5.4688901 -3.3647282 -4.0629153  C 6.3260641 -3.7261682 -2.1375333  H 7.0360651 -4.4631482 -2.4988293  C 6.2845301 -3.4037372 -0.7625163  H 6.9549981 -3.9073682 -0.0732473  C 5.4038071 -2.4657232 -0.2876063  H 5.3821831 -2.2408672 0.7723077  C 4.5089421 -1.7932242 -1.1635163  C 1.4669961 2.7656848 0.5732887  H 2.3229241 2.2622088 0.1230317  C 1.8806031 3.2075328 1.9750297  H 2.2599651 2.3518098 2.5361547  H 1.0536161 3.6456178 2.5435437  H 2.6725671 3.9590728 1.9054987  C 1.0604291 3.9033028 -0.3567733  C 0.4985781 5.0922268 0.1114067  H 0.3962261 5.2693958 1.1782787  C 0.0729891 6.0784538 -0.7772203  H -0.3630109 6.9954578 -0.3927133  C 0.2149091 5.8936988 -2.1478863  H -0.1116919 6.6635758 -2.8400343  C 0.7929811 4.7192958 -2.6265683  H 0.9273921 4.5745118 -3.6944693  C 1.2113211 3.7356828 -1.7378613  H 1.6609261 2.8205358 -2.1131023  C -0.9133579 2.1514688 1.1814307  H -0.8153089 3.2202208 1.3975137  C -1.1746289 1.4210738 2.5017837  H -0.3372619 1.6086758 3.1797307  H -1.2518359 0.3400178 2.3493227  H -2.0948349 1.7469578 2.9908277  C -2.0604769 2.0675798 0.1672947  C -3.3774929 2.3010968 0.5787677  H -3.6065159 2.4029948 1.6328777  C -4.4030789 2.4451778 -0.3516333  H -5.4087559 2.6566708 -0.0042813  C -4.1612339 2.2957138 -1.7139033  H -4.9737869 2.3867298 -2.4259513  C -2.8749039 2.0011618 -2.1458333  H -2.6579679 1.8959028 -3.2039833  C -1.8214529 1.9108098 -1.2207323  H -0.8034349 1.9316998 -1.5994413  C -3.3731749 -1.7680072 0.4533117  C -3.8209569 -0.9683982 -0.4677643  C -5.1249099 -0.6129322 -1.0019643  C -6.1807709 -0.2933192 -0.1405303  C -5.3327659 -0.6115692 -2.3852373  C -7.4279419 0.0277588 -0.6663663  H -5.9996539 -0.2613302 0.9291247  C -6.5862019 -0.3080732 -2.9027473  H -4.5052439 -0.8436242 -3.0498253  C -7.6345009 0.0191068 -2.0441733  H -8.2414089 0.2897138 0.0033827  H -6.7446879 -0.3192302 -3.9766443  H -8.6103559 0.2670458 -2.4496453  N -3.8179369 -2.4567532 1.5622907  C -3.1467999 -3.7598812 1.7063317  C -2.4540739 -3.9624272 0.3672767  H -3.8895389 -4.5355822 1.9068817  H -2.4146979 -3.7263922 2.5226627  H -1.7002169 -4.7550102 0.4262087  H -3.1755279 -4.2243812 -0.4114913  C -1.7355399 -2.6495992 0.0219677  C -1.4275019 -2.3303752 -1.3623013  H -0.9038009 -2.5016012 0.7139367  H -2.2383039 -2.5389832 -2.0672813  C -0.0808849 -2.5629912 -1.9487703  C 1.0263661 -3.2422852 -1.1877493  C 0.2812101 -4.0142852 -2.2316373  H 0.2059181 -1.8598542 -2.7270573  H 0.8618311 -3.4900522 -0.1443713  H 2.0443431 -2.9483112 -1.4088983  H 0.7764541 -4.2277412 -3.1731333  H -0.4190919 -4.7796242 -1.9073103  S -3.7308029 -1.4278672 3.0856977  O -2.3513649 -1.5987532 3.7211287  O -4.2471849 -0.0521902 2.6821547  C -4.9900459 -2.2776502 4.1154437  H -5.0592819 -1.7097422 5.0439167  H -5.9205929 -2.2546862 3.5496287  H -4.6335789 -3.2915842 4.2962167 |
| **(*R*)-7a-Rh-L1**  EBS1= -2546.478155  EBS2= -3270.269861  X Y Z  Rh 2.0737796 -0.3767376 -0.5823638  P -0.1098354 0.7886684 -0.4599728  O -1.1719374 0.0945354 -1.6160038  O -0.9515144 0.4319714 0.9794182  N -0.2948884 2.4759984 -0.3513848  C -3.2937874 -0.1136296 -0.5236828  C -2.5214274 0.3946914 -1.5459698  C -3.0501354 1.2115394 -2.5675218  H -2.3898484 1.5361014 -3.3649058  C -4.3709174 1.5639044 -2.5315308  H -4.7954954 2.1812934 -3.3181328  C -5.1953634 1.1646714 -1.4472868  C -6.5440294 1.5979384 -1.3565748  H -6.9435284 2.2249414 -2.1490918  C -7.3238554 1.2465154 -0.2865178  H -8.3521444 1.5876134 -0.2219298  C -6.7814094 0.4484724 0.7459882  H -7.3952304 0.1905334 1.6034312  C -5.4865304 0.0016074 0.6801082  H -5.0855774 -0.6004246 1.4874912  C -4.6557424 0.3323034 -0.4244568  C -2.7178404 -1.0879026 0.4448652  C -1.5823514 -0.7803026 1.1604502  C -1.0684114 -1.6148526 2.1763562  H -0.1814044 -1.2862676 2.7045142  C -1.7016694 -2.7895486 2.4648822  H -1.3324074 -3.4353686 3.2567022  C -2.8242314 -3.2095946 1.7053752  C -3.4205834 -4.4787826 1.9227022  H -3.0424444 -5.0997046 2.7307262  C -4.4348944 -4.9267536 1.1170222  H -4.8788844 -5.9029076 1.2845412  C -4.8992344 -4.1161506 0.0558202  H -5.6881234 -4.4817256 -0.5940068  C -4.3625144 -2.8721866 -0.1604158  H -4.7286544 -2.2630286 -0.9792208  C -3.3167664 -2.3743146 0.6629012  C -1.3723584 3.1525484 0.4275132  H -2.0010474 2.3513624 0.8149832  C -2.2546814 4.0371254 -0.4504788  H -2.7402934 3.4333524 -1.2199378  H -1.6999694 4.8414264 -0.9447318  H -3.0311524 4.5005394 0.1654862  C -0.7463724 3.8668204 1.6197802  C -0.5100364 5.2418924 1.6326162  H -0.8265794 5.8615724 0.7985452  C 0.1195646 5.8472574 2.7194302  H 0.2951556 6.9186824 2.7100462  C 0.5128056 5.0846244 3.8128072  H 0.9986266 5.5561614 4.6613982  C 0.2678276 3.7122394 3.8176092  H 0.5580736 3.1111904 4.6744092  C -0.3557874 3.1109894 2.7311452  H -0.5438104 2.0407074 2.7347492  C 0.6802546 3.3351554 -1.0349678  H 0.4788516 4.3407114 -0.6490918  C 0.4092456 3.3656444 -2.5445818  H -0.6435054 3.6070144 -2.7095898  H 0.5961066 2.3915244 -3.0076488  H 1.0089636 4.1173014 -3.0641218  C 2.1330696 3.0589244 -0.6351718  C 3.1866656 3.5398374 -1.4173908  H 2.9877916 4.0432094 -2.3574438  C 4.5110696 3.4124864 -0.9983388  H 5.3072026 3.8143864 -1.6178518  C 4.8116066 2.7791514 0.2044742  H 5.8421536 2.6845974 0.5330882  C 3.7746706 2.2811494 0.9894082  H 3.9870816 1.7875484 1.9323642  C 2.4490836 2.4326714 0.5803212  H 1.6576686 2.1178384 1.2529622  C 3.4968166 -1.8676536 -0.7588998  C 2.2908116 -2.3804946 -1.3272798  C 1.4311906 -3.4013606 -0.6633328  C 1.9801646 -4.4978636 0.0087462  C 0.0462226 -3.3533476 -0.8721748  C 1.1534806 -5.5168086 0.4814662  H 3.0556576 -4.5702246 0.1341802  C -0.7703464 -4.3802256 -0.4198418  H -0.3893794 -2.5064156 -1.3972728  C -0.2200664 -5.4596386 0.2686972  H 1.5916836 -6.3715446 0.9886312  H -1.8379844 -4.3420276 -0.6064748  H -0.8643814 -6.2580086 0.6233822  N 4.1154756 -2.3222706 0.4679582  C 5.5601126 -2.0013676 0.3523982  C 5.6525226 -0.8367206 -0.6384258  H 6.0374696 -2.9058306 -0.0365158  H 5.9872876 -1.7743406 1.3309492  H 5.4735106 0.1047254 -0.1091998  H 6.6286876 -0.7810156 -1.1240608  C 4.5072626 -1.1162396 -1.6226248  C 3.6567916 0.0971214 -1.9534368  H 4.8529156 -1.6819966 -2.4963508  H 4.0527816 1.0641774 -1.6516088  C 2.4539936 0.0941864 -2.6721888  C 1.8879576 -1.0397926 -3.4850288  C 2.1860006 -2.4044626 -2.8622518  H 2.0878356 1.0818124 -2.9376268  H 2.2643636 -0.9903146 -4.5150088  H 0.8041266 -0.8938126 -3.5360098  H 1.4093366 -3.1146476 -3.1490848  H 3.1271356 -2.8129156 -3.2511198  S 3.3590456 -1.6097826 1.9478182  O 4.3290486 -0.6943756 2.6888912  O 1.9699736 -1.0281786 1.4823372  C 2.9529376 -3.0622696 2.9796512  H 2.2749386 -3.6748406 2.3842422  H 3.8934936 -3.5663576 3.2019892  H 2.4830106 -2.6656396 3.8803712 | **(*S*)-7a-Rh-L1**  EBS1= -2546.481458  EBS2= -3270.273417  X Y Z  Rh 1.9388605 -0.4180592 0.0107901  P -0.1830765 0.8183168 -0.2130829  O -1.0940135 -0.0085782 -1.4132249  O -1.2693625 0.7764498 1.1122391  N -0.2449945 2.4914248 -0.4965009  C -3.3442495 -0.0485062 -0.6469939  C -2.4228635 0.3190948 -1.5990589  C -2.7914905 0.9819848 -2.7889649  H -2.0313655 1.1876528 -3.5327099  C -4.1004105 1.3273978 -2.9859299  H -4.4008305 1.8276368 -3.9023189  C -5.0819415 1.0573308 -1.9964489  C -6.4306915 1.4639348 -2.1682309  H -6.7057095 1.9836228 -3.0820859  C -7.3676425 1.2137188 -1.2004729  H -8.3964205 1.5301948 -1.3394259  C -6.9917685 0.5467618 -0.0120659  H -7.7349605 0.3647998 0.7580191  C -5.6985945 0.1323148 0.1799681  H -5.4245505 -0.3708332 1.1009161  C -4.7062745 0.3655698 -0.8094059  C -2.9040645 -0.8735332 0.5081171  C -1.9313225 -0.4128982 1.3676221  C -1.6064465 -1.0922242 2.5602071  H -0.9214845 -0.6184932 3.2529371  C -2.1756405 -2.3059252 2.8293651  H -1.9341075 -2.8327332 3.7482991  C -3.0665325 -2.9053872 1.9024361  C -3.5761115 -4.2131512 2.1098001  H -3.2853265 -4.7492792 3.0090251  C -4.4084315 -4.7950302 1.1899011  H -4.7908455 -5.7974492 1.3539381  C -4.7698175 -4.0878522 0.0201991  H -5.4228455 -4.5561892 -0.7096799  C -4.3060695 -2.8157102 -0.2007769  H -4.5879605 -2.2876552 -1.1047609  C -3.4428435 -2.1836502 0.7348021  C -1.2024235 3.4226978 0.1516771  H -1.8387725 2.8013058 0.7812941  C -2.1130035 4.1296908 -0.8483539  H -2.6941235 3.3907458 -1.4065559  H -1.5631765 4.7460448 -1.5670519  H -2.8072155 4.7836358 -0.3117419  C -0.3978945 4.3374768 1.0673601  C -0.1259375 5.6701488 0.7654771  H -0.5638025 6.1325528 -0.1141699  C 0.7097655 6.4297118 1.5869491  H 0.9121905 7.4665038 1.3363761  C 1.2756755 5.8639548 2.7232811  H 1.9228545 6.4557488 3.3630781  C 0.9906785 4.5361368 3.0469221  H 1.4108055 4.0938228 3.9456571  C 0.1610275 3.7845728 2.2258691  H -0.0654245 2.7503958 2.4776521  C 0.6988025 3.0571178 -1.4825639  H 0.6256715 4.1381928 -1.3347679  C 0.2731555 2.7602628 -2.9232579  H -0.7352985 3.1432558 -3.0952669  H 0.2595245 1.6851048 -3.1281609  H 0.9556685 3.2388828 -3.6322339  C 2.1684715 2.7242048 -1.2256919  C 2.8501675 1.7444908 -1.9605949  H 2.3190025 1.1172528 -2.6696809  C 4.2382545 1.6165608 -1.8668769  H 4.7399655 0.8687598 -2.4739859  C 4.9595225 2.4576018 -1.0257209  H 6.0418195 2.3839568 -0.9737409  C 4.2823745 3.3959478 -0.2440269  H 4.8373425 4.0511058 0.4205421  C 2.8995215 3.5228588 -0.3383199  H 2.3855555 4.2824218 0.2460471  C 3.3045625 -1.8896952 0.5439401  C 2.0389435 -2.2354132 1.0997281  C 1.2818505 -3.4030892 0.5438751  C 1.8554265 -4.6765952 0.6278371  C -0.0125925 -3.2691792 0.0376551  C 1.1436875 -5.7971372 0.2066051  H 2.8612605 -4.7884892 1.0228441  C -0.7227895 -4.3887362 -0.3844569  H -0.4673195 -2.2877642 -0.0432769  C -0.1461005 -5.6540532 -0.3018709  H 1.5956565 -6.7814092 0.2822711  H -1.7321915 -4.2671382 -0.7661439  H -0.7027525 -6.5280622 -0.6254469  N 3.9325545 -2.6180052 -0.5275389  C 5.3881375 -2.3544992 -0.4475899  C 5.5417445 -1.0460302 0.3399501  H 5.8266255 -2.3064492 -1.4466139  H 5.8239295 -3.2053352 0.0841721  H 6.4916805 -0.9999232 0.8760471  H 5.4967225 -0.1995252 -0.3499919  C 4.3256245 -1.0260052 1.2792311  C 3.5311135 0.2648448 1.2722451  H 4.5682675 -1.3992232 2.2823571  H 3.9564485 1.1243808 0.7621301  C 2.3370995 0.4193028 1.9861741  C 1.7384155 -1.9914422 2.5775031  C 1.9501835 -0.5516512 3.0805121  H 1.9506145 1.4336768 2.0680561  H 2.3445825 -2.7068192 3.1509181  H 0.6982025 -2.2823462 2.7341041  H 1.0369595 -0.1915532 3.5579211  H 2.7252945 -0.5212112 3.8568621  S 3.2105705 -2.2698652 -2.1457779  O 1.8748955 -1.4752962 -1.8780949  O 4.2469745 -1.6544782 -3.0821549  C 2.6922505 -3.9132362 -2.7547959  H 2.2038335 -3.7354282 -3.7134049  H 3.5998485 -4.5070572 -2.8617259  H 2.0112455 -4.3182572 -2.0059159 |
| **10, P-*trans*-ene/Up *Re***  by **L5**  E_BS1_= -2744.785556  E_BS2_= -3468.671203  X Y Z  Rh -2.0748605 -0.7719679 -0.2890603  P -0.1805155 0.5145011 0.2719037  O 0.9124295 -0.3250549 1.2302397  O 0.8656695 1.1044891 -0.9424653  N -0.6638885 2.0291871 0.8507197  C 3.1202885 0.3510341 0.5963877  C 2.1421235 0.2192191 1.5565777  C 2.3337445 0.5688191 2.9091347  H 1.5289975 0.3687351 3.6092467  C 3.5238915 1.1223841 3.2906887  H 3.6976025 1.3859391 4.3301177  C 4.5403695 1.3834111 2.3343107  C 5.7486635 2.0252051 2.7120357  H 5.8930325 2.2888211 3.7562737  C 6.7103965 2.3165521 1.7810687  H 7.6291935 2.8117051 2.0784797  C 6.4971035 1.9851611 0.4242267  H 7.2495845 2.2409401 -0.3150483  C 5.3472795 1.3500801 0.0298947  H 5.1975195 1.1138641 -1.0172833  C 4.3393665 1.0128821 0.9729617  C 2.8978165 -0.1499959 -0.7878043  C 1.8064375 0.2703471 -1.5138783  C 1.6327645 -0.0297759 -2.8813053  H 0.7717815 0.3830781 -3.3972463  C 2.5645755 -0.7946969 -3.5236703  H 2.4628925 -1.0097069 -4.5838133  C 3.6522065 -1.3560719 -2.8055833  C 4.5627935 -2.2433279 -3.4358463  H 4.4416885 -2.4457419 -4.4969003  C 5.5660495 -2.8450449 -2.7222383  H 6.2555855 -3.5250329 -3.2126023  C 5.6994405 -2.5828909 -1.3395433  H 6.4849015 -3.0744119 -0.7738073  C 4.8489435 -1.7122199 -0.7073793  H 4.9618595 -1.5234469 0.3543017  C 3.8068385 -1.0628369 -1.4208623  C 0.2699055 3.1756241 1.0220427  H 1.2427665 2.8171051 0.6763627  C 0.4110185 3.5592961 2.4932117  H 0.7827295 2.7045951 3.0622077  H -0.5382415 3.8757611 2.9374507  H 1.1263725 4.3803531 2.5988987  C -0.1507455 4.3182531 0.1043107  C -0.7502785 5.4855741 0.5808407  H -0.8965475 5.6347811 1.6458897  C -1.1650335 6.4914241 -0.2893273  H -1.6290865 7.4005081 0.0764327  C -0.9675045 6.3192141 -1.6489593  C -0.3623465 5.1779871 -2.1592353  H -0.2072465 5.0855031 -3.2285823  C 0.0425535 4.1864351 -1.2762733  H 0.5238815 3.2920841 -1.6597503  C -2.0815105 2.2508111 1.1607517  H -2.1707915 3.3395771 1.2502777  C -2.5038185 1.6212511 2.4912127  H -1.7219745 1.7612051 3.2393187  H -2.6622485 0.5481651 2.3728607  H -3.4271145 2.0602041 2.8793807  C -3.0201705 1.8683471 0.0106937  C -4.4106165 1.9211261 0.2141247  H -4.8059385 2.1364011 1.2005627  C -5.3069215 1.7361951 -0.8282823  H -6.3777815 1.7982301 -0.6677853  C -4.8175285 1.4658451 -2.1035723  C -3.4617725 1.4029051 -2.3536753  H -3.1016025 1.2299031 -3.3616993  C -2.5643885 1.6157241 -1.2983183  H -1.5167605 1.7448321 -1.5491853  C -1.7066895 -2.3255469 1.2003307  C -0.9230395 -2.4161289 0.2131847  C 0.2361995 -3.0548849 -0.3632803  C 0.4651555 -3.0467549 -1.7415523  C 1.1411385 -3.7026839 0.4906817  C 1.5566485 -3.7274179 -2.2659633  H -0.2087555 -2.4957499 -2.3888683  C 2.2462325 -4.3531459 -0.0387713  H 0.9783465 -3.6648509 1.5629367  C 2.4470575 -4.3795619 -1.4180623  H 1.7293415 -3.7265219 -3.3368263  H 2.9551315 -4.8394319 0.6234947  H 3.3161125 -4.8805919 -1.8312723  N -2.4887735 -2.7128229 2.2128977  C -3.9037385 -2.9833419 1.9164977  C -4.6254735 -1.8425339 1.2025087  H -4.4078715 -3.1816639 2.8655417  H -3.9240205 -3.9086879 1.3312817  H -5.6893435 -2.1143019 1.1555377  H -4.5697925 -0.9451919 1.8280027  C -4.1990285 -1.4993709 -0.2070413  C -3.5947385 -2.3121849 -1.1398033  H -4.7204635 -0.6268899 -0.5982353  H -3.2094515 -3.2849939 -0.8343813  C -3.7133825 -2.1183429 -2.5998813  C -2.7891195 -2.8407389 -3.5532753  C -4.2082815 -3.2918479 -3.4302463  H -4.0805245 -1.1455209 -2.9104583  H -2.0270435 -3.4822369 -3.1212573  H -2.4835345 -2.3167009 -4.4533603  H -4.9042175 -3.0643439 -4.2308153  H -4.4005745 -4.2299129 -2.9179873  S -2.1855765 -1.8276979 3.8123867  O -0.9008135 -1.0412529 3.6063717  O -3.4916225 -1.1640599 4.2383677  C -1.8509215 -3.2636359 4.9079917  H -1.6707545 -2.8539809 5.9026387  H -2.7365525 -3.8982789 4.8897357  H -0.9716335 -3.7620799 4.5024387  F -5.6775455 1.2492901 -3.1021133  F -1.3639085 7.2772441 -2.4937893 | **TS3, P-*trans*-ene/Up *Re*** by **L5**  E_BS1_= -2744.768305  E_BS2_= -3468.647169  Frequency=-250.9 cm^-1^  X Y Z  Rh 2.0064983 -0.6194340 -0.1887295  P 0.0061233 0.6374750 -0.5350355  O -1.0211877 -0.2536150 -1.5786245  O -1.0671647 0.8922850 0.7600285  N 0.1563763 2.2373900 -1.0737615  C -3.2450537 -0.0865050 -0.6969905  C -2.3504267 0.0913070 -1.7321015  C -2.7436697 0.5737130 -2.9985815  H -1.9981857 0.6272770 -3.7852325  C -4.0434717 0.9447760 -3.2049085  H -4.3620427 1.3083760 -4.1778265  C -4.9872407 0.8914700 -2.1463425  C -6.3184447 1.3489130 -2.3282955  H -6.6107237 1.7258030 -3.3046975  C -7.2154067 1.3283170 -1.2931315  H -8.2296997 1.6852150 -1.4401255  C -6.8133857 0.8538170 -0.0236805  H -7.5219637 0.8585510 0.7986405  C -5.5378177 0.3938390 0.1808545  H -5.2432787 0.0418870 1.1634525  C -4.5882167 0.3843100 -0.8762055  C -2.7912067 -0.7498030 0.5553265  C -1.7098307 -0.2427280 1.2388355  C -1.2312897 -0.8040400 2.4398185  H -0.4101107 -0.3202120 2.9564305  C -1.8299807 -1.9247140 2.9423405  H -1.4804947 -2.3647620 3.8720415  C -2.9155297 -2.5301410 2.2598035  C -3.5207827 -3.7110130 2.7613755  H -3.1422767 -4.1364810 3.6869495  C -4.5609687 -4.3049020 2.0968995  H -5.0229627 -5.2037670 2.4929335  C -5.0289807 -3.7470340 0.8857865  H -5.8440587 -4.2274400 0.3531835  C -4.4636647 -2.6064050 0.3750665  H -4.8299937 -2.1985740 -0.5598195  C -3.4024207 -1.9489490 1.0541485  C -0.9028787 3.2719900 -0.9307035  H -1.7258657 2.7736290 -0.4137895  C -1.4439347 3.7738720 -2.2691405  H -1.9030397 2.9533100 -2.8232595  H -0.6743137 4.2283360 -2.9007465  H -2.2098407 4.5328190 -2.0843735  C -0.3993327 4.3822100 -0.0199285  C 0.2332583 5.5293180 -0.5059505  H 0.3273813 5.6981250 -1.5745775  C 0.7486413 6.4900490 0.3611715  H 1.2394743 7.3831770 -0.0092125  C 0.6085623 6.2987370 1.7271265  C -0.0389227 5.1853320 2.2453685  H -0.1499737 5.0776440 3.3187485  C -0.5377987 4.2352060 1.3627435  H -1.0399597 3.3550940 1.7540445  C 1.3162083 2.5126520 -1.9498145  H 1.2903573 3.5936080 -2.1106165  C 1.1750423 1.8352770 -3.3160685  H 0.2437113 2.1555490 -3.7892435  H 1.1389143 0.7435060 -3.2291805  H 2.0083223 2.1012310 -3.9736545  C 2.6506913 2.2393790 -1.2594415  C 3.4752393 1.1560640 -1.6002895  H 3.2014843 0.4672530 -2.3955565  C 4.7445583 1.0051110 -1.0255045  H 5.3878513 0.1744840 -1.3001495  C 5.1761293 1.9524610 -0.1164615  C 4.3859723 3.0431830 0.2381455  H 4.7661093 3.7722390 0.9459345  C 3.1254983 3.1707850 -0.3248425  H 2.5015363 4.0186960 -0.0541795  C 2.7177893 -2.6062490 -0.0352275  C 1.4194483 -2.5202720 -0.0576435  C 0.2518013 -3.3747160 -0.0413975  C 0.1648233 -4.3779650 0.9362915  C -0.7501107 -3.2608650 -1.0114265  C -0.9115137 -5.2540990 0.9380145  H 0.9441653 -4.4617860 1.6881915  C -1.8136657 -4.1539550 -1.0124395  H -0.6830557 -2.4811080 -1.7596905  C -1.8975227 -5.1474840 -0.0407165  H -0.9807397 -6.0239390 1.6997725  H -2.5849477 -4.0691750 -1.7706775  H -2.7380687 -5.8333020 -0.0376215  N 3.7912293 -3.4406130 -0.2013465  C 4.6396913 -3.4677560 1.0063485  C 3.9851453 -2.5273960 2.0249205  H 5.6482753 -3.1250310 0.7503285  H 4.6946653 -4.4931540 1.3783115  H 3.1479293 -3.0204370 2.5282245  H 4.7154253 -2.2402350 2.7898185  C 3.5202593 -1.2607960 1.3284085  C 2.4172613 -0.4697570 1.8419675  H 4.3663113 -0.6831560 0.9508455  H 1.6652003 -1.0224170 2.4076865  C 2.6811323 0.9058600 2.3141925  C 1.5742403 1.8919120 2.5957435  C 2.3025803 1.2728940 3.7416535  H 3.6053243 1.3365170 1.9466785  H 0.5545933 1.5844250 2.3943525  H 1.7800293 2.9278540 2.3489215  H 3.0085183 1.8750340 4.3042795  H 1.7869983 0.5179150 4.3293835  S 4.7227543 -2.8781880 -1.7322835  O 3.7318393 -2.0521800 -2.5518775  O 6.0515253 -2.2651340 -1.2857175  C 5.0088403 -4.4917540 -2.5524925  H 5.5215693 -4.2782420 -3.4909485  H 5.6271223 -5.0833350 -1.8775545  H 4.0250343 -4.9324950 -2.7090725  F 1.1018493 7.2144490 2.5676665  F 6.3801033 1.8156060 0.4497095 |
| **10, P-*trans*-ene/Up *Si*** by **L5**  E_BS1_= -2744.777993  E_BS2_= -3468.665500  X Y Z  Rh -2.0019936 -0.8701455 -0.2224825  P -0.2182246 0.5348375 0.3111295  O 0.9470414 -0.2042935 1.2688935  O 0.7801774 1.1220985 -0.9455885  N -0.7797556 2.0288885 0.8556225  C 3.1076894 0.4778715 0.4922135  C 2.1727534 0.3949575 1.5003415  C 2.4081834 0.8525725 2.8131555  H 1.6368004 0.6886595 3.5584885  C 3.5964874 1.4629815 3.1012605  H 3.8015594 1.8157805 4.1080635  C 4.5703554 1.6670195 2.0884255  C 5.7772904 2.3593565 2.3678615  H 5.9523044 2.7111895 3.3809825  C 6.6990544 2.5916465 1.3815205  H 7.6179894 3.1245275 1.6039465  C 6.4445614 2.1477665 0.0643895  H 7.1661234 2.3546555 -0.7197705  C 5.2935414 1.4647675 -0.2352195  H 5.1118994 1.1435725 -1.2543575  C 4.3272844 1.1860305 0.7688895  C 2.8238394 -0.1115825 -0.8446645  C 1.6864954 0.2562925 -1.5276615  C 1.4204714 -0.1483805 -2.8518925  H 0.5200474 0.2167955 -3.3355105  C 2.3139814 -0.9555625 -3.4973775  H 2.1422664 -1.2525875 -4.5282235  C 3.4593344 -1.4472375 -2.8196825  C 4.3480034 -2.3542555 -3.4526935  H 4.1616894 -2.6279225 -4.4879905  C 5.4143954 -2.8835115 -2.7742285  H 6.0888074 -3.5763365 -3.2677205  C 5.6314934 -2.5318535 -1.4225185  H 6.4652474 -2.9691885 -0.8820055  C 4.8023454 -1.6409805 -0.7898965  H 4.9794874 -1.3835495 0.2483275  C 3.7022724 -1.0564915 -1.4719705  C 0.0993894 3.2130845 1.0487575  H 1.1006764 2.8892845 0.7507765  C 0.1583044 3.6146055 2.5206175  H 0.5198454 2.7738085 3.1163475  H -0.8208486 3.9078105 2.9126095  H 0.8449724 4.4558015 2.6540585  C -0.3204686 4.3306355 0.1007705  C -0.9992846 5.4711615 0.5341585  H -1.2093016 5.6193545 1.5887045  C -1.4117186 6.4515835 -0.3655225  H -1.9367686 7.3402295 -0.0336245  C -1.1316186 6.2811815 -1.7109435  C -0.4450276 5.1677735 -2.1775105  H -0.2275046 5.0765785 -3.2360285  C -0.0432296 4.2014855 -1.2655225  H 0.4992194 3.3281935 -1.6146265  C -2.2218836 2.1896235 1.0851385  H -2.3637856 3.2764605 1.1162945  C -2.6985376 1.6084045 2.4193235  H -1.9369156 1.7479775 3.1868305  H -2.8881126 0.5361685 2.3268845  H -3.6199516 2.0872045 2.7630245  C -3.0757346 1.7212325 -0.0993735  C -4.4735166 1.6331565 0.0538885  H -4.9175686 1.7386915 1.0373365  C -5.3143496 1.4628135 -1.0350015  H -6.3913526 1.4200785 -0.9144475  C -4.7605076 1.3543535 -2.3094795  C -3.3954856 1.4090535 -2.5070055  H -2.9891876 1.3464755 -3.5104655  C -2.5559016 1.5950875 -1.4022725  H -1.5049376 1.7846035 -1.5911415  C -1.4294776 -2.2745485 1.4467915  C -0.7566356 -2.3997855 0.3788155  C 0.3638194 -3.0684655 -0.2435075  C 0.3993774 -3.2677795 -1.6266955  C 1.4116774 -3.5422435 0.5588165  C 1.4459244 -3.9832975 -2.1943425  H -0.3972366 -2.8605395 -2.2410945  C 2.4677134 -4.2308815 -0.0205555  H 1.3952454 -3.3441775 1.6257425  C 2.4775684 -4.4665635 -1.3944205  H 1.4707354 -4.1446225 -3.2672685  H 3.2872374 -4.5830315 0.5972655  H 3.3068524 -5.0005995 -1.8457135  N -2.1501326 -2.5698355 2.5178925  C -3.5157836 -3.0916095 2.3539655  C -4.3202846 -2.3965795 1.2495575  H -4.0310656 -2.9767125 3.3103185  H -3.4203826 -4.1609855 2.1359375  H -5.2956326 -2.9008845 1.2181055  H -4.5072756 -1.3489595 1.5082865  C -3.6677296 -2.5218165 -0.1045925  C -3.8915016 -1.7109635 -1.1816085  H -3.1630996 -3.4706925 -0.2822415  H -4.5454976 -0.8503985 -1.0428555  C -3.5928226 -2.0600915 -2.5938695  C -2.9564016 -3.3623255 -3.0096845  C -4.4156956 -3.1475695 -3.2657125  H -3.3712246 -1.2020345 -3.2209025  H -2.6677616 -4.0801935 -2.2480425  H -2.2777326 -3.3276045 -3.8563225  H -4.7518256 -2.9714925 -4.2820945  H -5.1195176 -3.6864845 -2.6385375  S -1.8654936 -1.5231775 4.0225565  O -0.7108666 -0.5981165 3.6768575  O -3.2278556 -1.0111505 4.4743315  C -1.2804556 -2.8088375 5.1976975  H -1.1036146 -2.3016775 6.1469835  H -2.0731636 -3.5519355 5.2808835  H -0.3650406 -3.2205605 4.7746595  F -5.5693926 1.1825155 -3.3559805  F -1.5257636 7.2143655 -2.5840355 | **TS3, P-*trans*-ene/Up *Si*** by **L5**  E_BS1_= -2744.765879  E_BS2_= -3468.636011  Frequency=-204.1 cm^-1^  X Y Z  Rh 1.9997065 -0.7468561 -0.2508719  P 0.0102245 0.5307539 -0.6510789  O -1.0767655 -0.2875851 -1.7028069  O -0.9960635 0.7244229 0.7069051  N 0.1185255 2.1705069 -1.0877449  C -3.2678665 0.0653589 -0.8034019  C -2.3670465 0.1940459 -1.8397919  C -2.7144865 0.7744369 -3.0778609  H -1.9734685 0.7909289 -3.8695659  C -3.9712565 1.2860109 -3.2521549  H -4.2564795 1.7251939 -4.2039609  C -4.9095505 1.2802149 -2.1872829  C -6.1899625 1.8750969 -2.3338669  H -6.4478915 2.3271879 -3.2876869  C -7.0806315 1.8888559 -1.2931309  H -8.0559565 2.3492799 -1.4135799  C -6.7240185 1.3106449 -0.0532429  H -7.4277945 1.3384459 0.7727651  C -5.4987145 0.7191519 0.1169811  H -5.2376605 0.2838689 1.0754041  C -4.5576525 0.6744929 -0.9467559  C -2.8804585 -0.7113921 0.4047651  C -1.7448805 -0.3732941 1.1053721  C -1.3146215 -1.0743621 2.2479971  H -0.4053385 -0.7499981 2.7373331  C -2.0449295 -2.1387341 2.6975261  H -1.7314135 -2.6850561 3.5825061  C -3.1999075 -2.5688391 1.9971501  C -3.9246085 -3.7136411 2.4192211  H -3.6068735 -4.2243701 3.3242811  C -4.9889975 -4.1790911 1.6936181  H -5.5351345 -5.0576641 2.0225441  C -5.3703725 -3.5171441 0.5039271  H -6.2016015 -3.8998081 -0.0801769  C -4.7046635 -2.3946981 0.0827011  H -5.0104335 -1.9023661 -0.8332909  C -3.6099485 -1.8722981 0.8244151  C -0.8602295 3.2100649 -0.6687289  H -1.6485095 2.6709339 -0.1422769  C -1.5208795 3.9295059 -1.8433529  H -2.0690375 3.2157149 -2.4614079  H -0.8102255 4.4639279 -2.4818419  H -2.2321865 4.6654269 -1.4566419  C -0.1947845 4.1407199 0.3358111  C 0.4512445 5.3204059 -0.0400629  H 0.4278575 5.6553209 -1.0730479  C 1.1261345 6.1037589 0.8941831  H 1.6277555 7.0218529 0.6087861  C 1.1341005 5.7004239 2.2203541  C 0.4740215 4.5520589 2.6368631  H 0.4699075 4.2883689 3.6892371  C -0.1866105 3.7813709 1.6877931  H -0.7086845 2.8801249 1.9983341  C 1.1719975 2.5358049 -2.0568119  H 1.1668875 3.6284329 -2.0743049  C 0.8440595 2.0473779 -3.4709569  H -0.1080525 2.4737099 -3.7961199  H 0.7460295 0.9570409 -3.5111379  H 1.6208915 2.3516979 -4.1790529  C 2.5752725 2.1504299 -1.5970049  C 3.2403475 1.0086389 -2.0735909  H 2.7712325 0.3492219 -2.7972189  C 4.5684335 0.7472609 -1.7206909  H 5.0926225 -0.1210881 -2.1064999  C 5.2176765 1.6361549 -0.8812629  C 4.5835405 2.7638679 -0.3733329  H 5.1261385 3.4349359 0.2835041  C 3.2630965 3.0104089 -0.7343249  H 2.7613645 3.8921879 -0.3449069  C 2.7560165 -2.6047341 0.3524441  C 1.4546895 -2.6185101 0.2820011  C 0.3144265 -3.4966301 0.3135911  C 0.2931915 -4.5648101 1.2262141  C -0.7386065 -3.3353131 -0.5966939  C -0.7676685 -5.4589921 1.2187541  H 1.1084755 -4.6795771 1.9343921  C -1.7857025 -4.2468621 -0.6085779  H -0.7274115 -2.5029491 -1.2919739  C -1.8035785 -5.3044421 0.2974401  H -0.7865965 -6.2798381 1.9285641  H -2.5997345 -4.1219041 -1.3142669  H -2.6327445 -6.0041641 0.2939781  N 3.9116455 -3.2925131 0.1381961  C 5.0215755 -2.8162671 0.9782911  C 4.8682205 -1.3134561 1.2476011  H 5.9724055 -3.0452931 0.4939421  H 4.9586565 -3.3911701 1.9081081  H 5.4760835 -1.0468241 2.1203411  H 5.2301595 -0.7267901 0.4010921  C 3.4241975 -1.0101481 1.5439051  C 2.8160195 0.2792719 1.3590291  H 3.0203755 -1.5732111 2.3854751  H 3.4188585 0.9903319 0.7893851  C 1.9902225 0.9800769 2.3724311  C 1.5562845 0.3970669 3.6887181  C 2.6762645 1.3942059 3.6612161  H 1.3427885 1.7360139 1.9381861  H 1.7946325 -0.6361451 3.9256631  H 0.5985165 0.7269399 4.0795551  H 2.4939455 2.3998499 4.0259231  H 3.6840095 1.0260009 3.8334131  S 4.3349385 -3.1039061 -1.7268729  O 3.1808325 -2.3324851 -2.3811239  O 5.7700535 -2.5853691 -1.8342649  C 4.2353515 -4.8632411 -2.2364259  H 4.4366095 -4.8915911 -3.3078119  H 4.9911325 -5.3972831 -1.6615009  H 3.2238375 -5.1904961 -1.9973719  F 6.4828775 1.3851579 -0.5234549  F 1.7823245 6.4422399 3.1250431 |
| **10, P-*trans*-ene/Up *Re***  by **L6**  E_BS1_= -2775.352101  E_BS2_= -3499.235575  X Y Z  Rh -1.5678677 -1.3566080 -0.0949618  P 0.1340343 0.1841410 0.4488782  O 1.4131403 -0.5495300 1.2537862  O 0.9871543 1.0325900 -0.7646118  N -0.5325327 1.5447730 1.1958222  C 3.4394533 0.5246740 0.5763442  C 2.5582863 0.1579540 1.5685672  C 2.7781773 0.4221640 2.9360572  H 2.0579043 0.0420120 3.6533092  C 3.8903843 1.1250090 3.3060172  H 4.0853843 1.3296080 4.3549702  C 4.7934913 1.6197020 2.3283312  C 5.9124793 2.4082210 2.7028332  H 6.0788613 2.6055580 3.7584182  C 6.7610363 2.9199050 1.7569382  H 7.6119293 3.5249620 2.0534742  C 6.5169833 2.6707970 0.3877932  H 7.1776553 3.0962050 -0.3612098  C 5.4520883 1.9012580 -0.0056178  H 5.2756643 1.7296460 -1.0611438  C 4.5658953 1.3357410 0.9504172  C 3.2007373 0.1191900 -0.8362508  C 2.0111783 0.4233210 -1.4601338  C 1.8014413 0.2304250 -2.8423368  H 0.8561343 0.5468240 -3.2715938  C 2.7974803 -0.3095940 -3.6051948  H 2.6629223 -0.4367870 -4.6757848  C 4.0004253 -0.7594320 -3.0017288  C 4.9927233 -1.4313370 -3.7616048  H 4.8380773 -1.5505830 -4.8308968  C 6.1155703 -1.9372750 -3.1605868  H 6.8666973 -2.4545850 -3.7493928  C 6.2922463 -1.7907890 -1.7657458  H 7.1737193 -2.2093260 -1.2899468  C 5.3624983 -1.1245060 -1.0091008  H 5.5093863 -1.0226650 0.0602092  C 4.1942163 -0.5777350 -1.6032208  C 0.2046943 2.8262640 1.3837842  H 1.1886693 2.6669360 0.9359492  C 0.4112533 3.1309430 2.8656382  H 0.9667873 2.3175380 3.3374292  H -0.5357667 3.2527150 3.4013202  H 0.9854493 4.0551140 2.9801642  C -0.4847767 3.9340530 0.5989732  C -1.2171007 4.9504470 1.2045142  H -1.2883097 5.0074140 2.2866532  C -1.8698527 5.9320690 0.4556872  H -2.4241737 6.7071310 0.9720282  C -1.7896797 5.9047600 -0.9356758  C -1.0468627 4.8929840 -1.5601958  H -0.9771837 4.8969110 -2.6430828  C -0.4072187 3.9309480 -0.8023708  H 0.1756363 3.1599350 -1.2976378  C -1.9350047 1.4936740 1.6290902  H -2.1973497 2.5432440 1.8050982  C -2.1143447 0.7201490 2.9367012  H -1.3327027 0.9946270 3.6470292  H -2.0374537 -0.3529530 2.7562362  H -3.0824757 0.9188370 3.4045872  C -2.8897687 1.0192890 0.5285392  C -4.2403467 0.7970070 0.8301502  H -4.5800977 0.8541370 1.8587102  C -5.1862627 0.5363660 -0.1549178  H -6.2197857 0.3888370 0.1366762  C -4.7986907 0.4598980 -1.4996088  C -3.4624567 0.6839810 -1.8280528  H -3.1696007 0.6681410 -2.8724788  C -2.5263697 0.9720250 -0.8353978  H -1.5454517 1.3124280 -1.1517038  C -0.8134827 -2.9349660 1.1884162  C -0.1146677 -2.8120070 0.1419812  C 1.0736873 -3.2059540 -0.5758408  C 1.1975453 -3.0049780 -1.9528038  C 2.1173963 -3.8162300 0.1357272  C 2.3276213 -3.4601060 -2.6202528  H 0.4102483 -2.4820610 -2.4854008  C 3.2570783 -4.2382590 -0.5331168  H 2.0281513 -3.9329300 1.2109282  C 3.3575503 -4.0743580 -1.9139578  H 2.4190273 -3.3106480 -3.6905888  H 4.0711293 -4.6950480 0.0203552  H 4.2523793 -4.3972350 -2.4355878  N -1.4204687 -3.5604140 2.2064992  C -2.7809447 -4.0662080 1.9719942  C -3.7586747 -3.0244680 1.4320282  H -3.1619407 -4.4493060 2.9223242  H -2.6776317 -4.9173500 1.2907392  H -4.7520557 -3.4947050 1.4208622  H -3.8225327 -2.1984400 2.1481152  C -3.5198237 -2.4709440 0.0452622  C -2.8603597 -3.0616930 -1.0089168  H -4.2211177 -1.6788130 -0.2128048  H -2.2836247 -3.9710200 -0.8393318  C -3.1366997 -2.7543780 -2.4278718  C -2.1685147 -3.1753390 -3.5092068  C -3.4557467 -3.9182470 -3.3522998  H -3.7088647 -1.8495790 -2.6052358  H -1.2619897 -3.6849980 -3.1978778  H -2.0477437 -2.5131120 -4.3606808  H -4.2438347 -3.7632790 -4.0817958  H -3.4157137 -4.9207680 -2.9363618  S -1.1613767 -2.7841160 3.8645092  O -0.0420387 -1.7733720 3.6742862  O -2.5248477 -2.3949950 4.4288232  C -0.5208287 -4.2355190 4.7904152  H -0.3412487 -3.8940850 5.8105582  H -1.2897327 -5.0066650 4.7555842  H 0.3977223 -4.5396490 4.2906402  O -5.6201027 0.1647070 -2.5275018  O -2.3773947 6.7974560 -1.7629078  C -6.9936147 -0.0466460 -2.2543978  H -7.4605097 -0.2525250 -3.2168448  H -7.1381577 -0.9058400 -1.5877498  H -7.4523957 0.8451330 -1.8121658  C -3.1047337 7.8625600 -1.1881368  H -3.4718887 8.4599180 -2.0228168  H -3.9572457 7.4960930 -0.6022368  H -2.4654447 8.4856140 -0.5502948 | **TS3, P-*trans*-ene/Up *Re*** by **L6**  E_BS1_= -2775.332988  E_BS2_= -3499.208616  Frequency=-242.6 cm^-1^  X Y Z  Rh 1.8819697 -0.8657336 -0.3390657  P -0.1894063 0.1950144 -0.8522227  O -1.1668073 -0.8652626 -1.7894267  O -1.2632493 0.4879014 0.4356593  N -0.1998143 1.7417864 -1.5440357  C -3.3995893 -0.6662566 -0.9481527  C -2.4991653 -0.5550066 -1.9875947  C -2.8829203 -0.1547616 -3.2846887  H -2.1298323 -0.1383826 -4.0653107  C -4.1848313 0.1875474 -3.5254087  H -4.4975253 0.4862864 -4.5220807  C -5.1396693 0.1859604 -2.4755397  C -6.4764593 0.6064024 -2.7011247  H -6.7639753 0.9098754 -3.7042117  C -7.3850473 0.6384154 -1.6765937  H -8.4043583 0.9638114 -1.8580267  C -6.9897913 0.2575834 -0.3737957  H -7.7081603 0.3036024 0.4387483  C -5.7090913 -0.1651376 -0.1259227  H -5.4202433 -0.4465106 0.8808253  C -4.7475263 -0.2302536 -1.1703817  C -2.9399993 -1.2083796 0.3594093  C -1.8774613 -0.6158446 1.0035943  C -1.4044913 -1.0540136 2.2571243  H -0.5991293 -0.5051866 2.7329553  C -1.9867213 -2.1372516 2.8539943  H -1.6383063 -2.4848376 3.8224473  C -3.0462703 -2.8301806 2.2150773  C -3.6270533 -3.9811966 2.8073933  H -3.2560233 -4.3120816 3.7737753  C -4.6280223 -4.6689866 2.1734403  H -5.0681713 -5.5470076 2.6356943  C -5.0824823 -4.2385046 0.9061353  H -5.8641143 -4.7952156 0.3982843  C -4.5468873 -3.1245136 0.3119133  H -4.9029283 -2.8128236 -0.6634887  C -3.5256053 -2.3736436 0.9542063  C -1.3573213 2.6733464 -1.4031947  H -2.1697093 2.0682504 -0.9974937  C -1.8556453 3.2314624 -2.7369957  H -2.1638783 2.4229074 -3.4037447  H -1.1162083 3.8520164 -3.2526907  H -2.7276573 3.8619634 -2.5414387  C -1.0579973 3.7547114 -0.3795917  C -0.2199863 4.8431154 -0.6550737  H 0.1946967 4.9824064 -1.6504057  C 0.0886887 5.7780754 0.3195443  H 0.7360817 6.6223064 0.1058023  C -0.4609743 5.6652674 1.6034033  C -1.3315833 4.6111794 1.8835713  H -1.7904733 4.5052034 2.8596753  C -1.6138843 3.6715294 0.8932233  H -2.2742383 2.8424494 1.1316193  C 0.9019867 2.0560724 -2.4837127  H 0.7994587 3.1237254 -2.6835577  C 0.7409087 1.3173584 -3.8163567  H -0.2135213 1.5842044 -4.2758627  H 0.7464117 0.2304604 -3.6784147  H 1.5457567 1.5822274 -4.5088747  C 2.2883277 1.8868014 -1.8704877  C 3.0852667 0.7463294 -2.0885417  H 2.7580487 -0.0459866 -2.7581997  C 4.3779807 0.6615524 -1.5743217  H 4.9933497 -0.2122966 -1.7627297  C 4.9148567 1.7235004 -0.8406827  C 4.1469257 2.8760614 -0.6454187  H 4.5416397 3.7267144 -0.1015427  C 2.8491077 2.9371454 -1.1458977  H 2.2622027 3.8331194 -0.9647677  C 2.7230007 -2.7264266 0.2151113  C 1.4222847 -2.7396416 0.1704943  C 0.3388977 -3.6860356 0.3297843  C 0.3960697 -4.5968716 1.3970313  C -0.7122203 -3.7607136 -0.5904007  C -0.5837463 -5.5695626 1.5344243  H 1.2130067 -4.5349676 2.1102043  C -1.6768693 -4.7521846 -0.4573867  H -0.7602803 -3.0489586 -1.4060707  C -1.6176213 -5.6526106 0.6025563  H -0.5394133 -6.2697016 2.3627423  H -2.4844463 -4.8142116 -1.1786847  H -2.3831193 -6.4141036 0.7084173  N 3.8506007 -3.5012446 0.2173393  C 4.7166377 -3.1935076 1.3722113  C 3.9848507 -2.1273056 2.1942863  H 5.6814187 -2.8186726 1.0120703  H 4.8816857 -4.1079696 1.9461933  H 3.1881317 -2.5776146 2.7941193  H 4.6881877 -1.6402276 2.8795123  C 3.4255677 -1.0636196 1.2671473  C 2.2581277 -0.2760306 1.6172023  H 4.2273267 -0.5116906 0.7721293  H 1.5517697 -0.7585116 2.2949293  C 2.3843517 1.1877194 1.7728383  C 1.1800607 2.0875154 1.9163713  C 2.0337467 1.8227894 3.1114943  H 3.2336667 1.6205654 1.2574763  H 0.1971627 1.6328114 1.8692383  H 1.2401667 3.0580844 1.4332843  H 2.6937187 2.6047224 3.4735683  H 1.6420047 1.1696814 3.8867233  S 4.7218077 -3.2625126 -1.4345627  O 3.6994097 -2.6192426 -2.3719067  O 6.0640227 -2.5811106 -1.1679007  C 4.9799807 -5.0109886 -1.9206027  H 5.4651397 -4.9942506 -2.8970267  H 5.6167647 -5.4579466 -1.1575947  H 3.9915467 -5.4672176 -1.9575427  O -0.0955963 6.6227074 2.4850113  O 6.1629667 1.5363784 -0.3600007  C 6.7871477 2.6069044 0.3214923  H 6.2452727 2.8665574 1.2401153  H 7.7857157 2.2548394 0.5788073  H 6.8685067 3.4936034 -0.3183497  C -0.6578693 6.5863244 3.7799133  H -0.2406083 7.4416574 4.3113183  H -1.7503573 6.6795124 3.7438483  H -0.3880383 5.6628834 4.3081773 |
| **10, P-*trans*-ene/Up *Si***  by **L6**  E_BS1_= -2775.344409  E_BS2_= -3499.22936  X Y Z  Rh -1.5838002 -1.3655847 0.0892084  P 0.0649848 0.1684653 0.6810534  O 1.4217148 -0.5343387 1.3846324  O 0.8287118 1.0598913 -0.5623466  N -0.6019142 1.4665863 1.5174514  C 3.3682218 0.5696883 0.5400794  C 2.5710458 0.2031873 1.6018614  C 2.8844068 0.5012023 2.9438634  H 2.2264058 0.1218963 3.7188194  C 4.0043498 1.2352753 3.2173734  H 4.2688048 1.4689403 4.2448204  C 4.8258658 1.7217673 2.1663474  C 5.9547608 2.5365103 2.4423124  H 6.1907888 2.7627013 3.4786454  C 6.7256158 3.0371983 1.4266674  H 7.5848208 3.6623173 1.6480154  C 6.3914488 2.7492213 0.0841954  H 6.9913578 3.1653223 -0.7192156  C 5.3137138 1.9554263 -0.2151526  H 5.0675768 1.7539463 -1.2514836  C 4.5060288 1.4020183 0.8146924  C 3.0141628 0.1343693 -0.8381946  C 1.7655138 0.4153893 -1.3462206  C 1.3984888 0.1291023 -2.6772626  H 0.4054518 0.4109923 -3.0129396  C 2.3010158 -0.4721587 -3.5083066  H 2.0441038 -0.6799447 -4.5432666  C 3.5730718 -0.8700907 -3.0222866  C 4.4849908 -1.5732877 -3.8508226  H 4.2106458 -1.7609457 -4.8856946  C 5.6822228 -2.0220327 -3.3571426  H 6.3713408 -2.5629437 -3.9982196  C 6.0151668 -1.7893297 -2.0034316  H 6.9545798 -2.1646577 -1.6091226  C 5.1640768 -1.0928117 -1.1837866  H 5.4312808 -0.9234127 -0.1467276  C 3.9252918 -0.5963507 -1.6701876  C 0.1205328 2.7450893 1.7644314  H 1.1103508 2.6127143 1.3193484  C 0.3089358 2.9807423 3.2614734  H 0.8759738 2.1553353 3.6974254  H -0.6450772 3.0520363 3.7934024  H 0.8597018 3.9111923 3.4277584  C -0.5647782 3.8873803 1.0277494  C -1.4156172 4.7973513 1.6663654  H -1.5779872 4.7379533 2.7386294  C -2.0586162 5.8011693 0.9594874  H -2.7149322 6.5069043 1.4574484  C -1.8581542 5.9314963 -0.4181076  C -1.0025972 5.0403383 -1.0717346  H -0.8116442 5.1261093 -2.1353756  C -0.3701532 4.0350373 -0.3456656  H 0.2942498 3.3481313 -0.8619326  C -2.0121872 1.3736103 1.9176614  H -2.2990142 2.4146743 2.1059284  C -2.2327822 0.5795883 3.2080044  H -1.3974452 0.7295943 3.8926534  H -2.3095142 -0.4906637 3.0025644  H -3.1494052 0.8913983 3.7175564  C -2.9333382 0.9253403 0.7782004  C -4.2739042 0.5886163 1.0745294  H -4.5759982 0.4409763 2.1055434  C -5.2293422 0.4873033 0.0858424  H -6.2612322 0.2521103 0.3250734  C -4.8894802 0.6955373 -1.2649326  C -3.5729352 1.0022453 -1.5899266  H -3.2736642 1.1892563 -2.6145046  C -2.6100432 1.1178863 -0.5762706  H -1.6361352 1.5113373 -0.8505146  C -0.6338152 -2.9327407 1.3701564  C -0.0900782 -2.7904137 0.2344134  C 0.9942738 -3.2049437 -0.6279656  C 0.8029748 -3.3055957 -2.0093916  C 2.2285398 -3.5566757 -0.0648466  C 1.8174258 -3.8123797 -2.8113996  H -0.1453112 -2.9944377 -2.4362716  C 3.2470888 -4.0340457 -0.8782396  H 2.3800728 -3.4302557 1.0021824  C 3.0364928 -4.1808257 -2.2480666  H 1.6636668 -3.9057387 -3.8819086  H 4.2082068 -4.2902367 -0.4452576  H 3.8329748 -4.5586217 -2.8800636  N -1.1656932 -3.5269277 2.4334734  C -2.4588512 -4.2145637 2.2972774  C -3.4835682 -3.4623257 1.4403334  H -2.8633862 -4.3660847 3.3004924  H -2.2482782 -5.1953067 1.8565854  H -4.3824472 -4.0932497 1.4107774  H -3.7665722 -2.5185467 1.9182904  C -2.9972782 -3.2442887 0.0293204  C -3.4473352 -2.2935797 -0.8414496  H -2.3940872 -4.0553347 -0.3770946  H -4.1874942 -1.5799177 -0.4832386  C -3.2606482 -2.3304577 -2.3166496  C -2.7162042 -3.5232347 -3.0578856  C -4.1811692 -3.2112197 -3.1409106  H -3.0418432 -1.3608997 -2.7555246  H -2.4142942 -4.4054667 -2.5021606  H -2.1025562 -3.3237247 -3.9309806  H -4.5813922 -2.8076667 -4.0656236  H -4.8626582 -3.8567827 -2.5951776  S -0.8699422 -2.7072597 4.0660434  O 0.1111848 -1.5784467 3.7987634  O -2.2328042 -2.4799807 4.7103144  C -0.0054712 -4.0659427 4.9512174  H 0.1952378 -3.6971527 5.9578754  H -0.6807532 -4.9211267 4.9642424  H 0.9103208 -4.2644557 4.3960014  O -5.8963962 0.5592933 -2.1463226  O -2.5245402 6.9425573 -1.0193286  C -5.6177602 0.7494373 -3.5198996  H -6.5634072 0.5983653 -4.0392326  H -5.2524602 1.7645153 -3.7151576  H -4.8832622 0.0169123 -3.8783096  C -2.3109132 7.1576553 -2.3976096  H -2.9163662 8.0242253 -2.6639986  H -1.2562092 7.3725393 -2.6102596  H -2.6332962 6.2940353 -2.9938796 | **TS3, P-*trans*-ene/Up *Si*** by **L6**  E_BS1_= -2775.331874  E_BS2_= -3499.200761  Frequency=-209.3 cm^-1^  X Y Z  Rh 1.3829820 -1.4082999 -0.3472638  P -0.3223190 0.2295881 -0.7455808  O -1.5813620 -0.4272979 -1.7217198  O -1.2364590 0.6946171 0.6118502  N 0.0878280 1.7804251 -1.2952038  C -3.6339910 0.4272641 -0.8320838  C -2.7483420 0.2969441 -1.8817138  C -2.9955010 0.8526621 -3.1547688  H -2.2829940 0.6630351 -3.9502098  C -4.1215830 1.6028651 -3.3543738  H -4.3291550 2.0299181 -4.3314958  C -5.0141790 1.8676251 -2.2830288  C -6.1396920 2.7143461 -2.4581228  H -6.3175660 3.1481871 -3.4383998  C -6.9791660 2.9914561 -1.4115828  H -7.8340190 3.6449291 -1.5534978  C -6.7251400 2.4330971 -0.1378648  H -7.3839500 2.6698671 0.6918432  C -5.6528330 1.6017371 0.0609772  H -5.4665290 1.1861081 1.0452422  C -4.7698870 1.2845141 -1.0063808  C -3.3862410 -0.3339359 0.4221072  C -2.1852160 -0.2000999 1.0813892  C -1.8750280 -0.9130649 2.2553002  H -0.9053530 -0.7565909 2.7091412  C -2.7939830 -1.7768909 2.7813722  H -2.5725010 -2.3273839 3.6912902  C -4.0349450 -1.9927699 2.1310772  C -4.9666010 -2.9373099 2.6343902  H -4.7329070 -3.4537599 3.5615552  C -6.1270410 -3.2093089 1.9592972  H -6.8327530 -3.9359589 2.3497382  C -6.4016620 -2.5463769 0.7409882  H -7.3130840 -2.7771979 0.1980712  C -5.5313870 -1.6118379 0.2405262  H -5.7576630 -1.1173499 -0.6970728  C -4.3283420 -1.2904739 0.9272212  C -0.6328450 3.0277341 -0.9199058  H -1.4880760 2.7013581 -0.3266738  C -1.1840800 3.7830581 -2.1276308  H -1.8982730 3.1584031 -2.6672848  H -0.4068370 4.0986981 -2.8312078  H -1.7024400 4.6826161 -1.7826268  C 0.2683140 3.8559901 -0.0173558  C 1.0619980 4.9004811 -0.4790988  H 0.9938710 5.2252761 -1.5135648  C 1.9602740 5.5663381 0.3599152  H 2.5572150 6.3766811 -0.0423648  C 2.0645960 5.1875301 1.6982202  C 1.2462940 4.1579551 2.1852162  H 1.3120890 3.9015611 3.2379472  C 0.3649110 3.5120261 1.3386652  H -0.2742820 2.7232601 1.7275982  C 1.1882020 1.8699751 -2.2772778  H 1.3857300 2.9408331 -2.3719778  C 0.7571470 1.3532601 -3.6525608  H -0.1164360 1.9134511 -3.9955908  H 0.4767690 0.2946001 -3.6183198  H 1.5604240 1.4731761 -4.3861508  C 2.4943540 1.2621501 -1.7782988  C 2.9622290 0.0054261 -2.2044708  H 2.3931310 -0.5922549 -2.9101308  C 4.2161900 -0.4635479 -1.8193868  H 4.5841070 -1.4213569 -2.1753198  C 5.0293680 0.3101701 -0.9868298  C 4.5668730 1.5519661 -0.5356878  H 5.1722510 2.1728791 0.1146332  C 3.3129060 2.0111441 -0.9330498  H 2.9713040 2.9805061 -0.5789018  C 1.7702700 -3.3752059 0.2507222  C 0.4905000 -3.1198829 0.2415612  C -0.7948870 -3.7516539 0.3898802  C -0.9472090 -4.7609559 1.3555832  C -1.8711410 -3.4147019 -0.4420918  C -2.1612460 -5.4206979 1.4827212  H -0.1103660 -5.0149699 1.9995502  C -3.0744790 -4.0967559 -0.3219288  H -1.7563790 -2.6258429 -1.1776248  C -3.2234070 -5.0926799 0.6406402  H -2.2794310 -6.1946569 2.2345172  H -3.9074970 -3.8350619 -0.9652778  H -4.1742260 -5.6053899 0.7425122  N 2.7470510 -4.2968999 0.0168852  C 3.9562450 -4.0557899 0.8195482  C 4.1301220 -2.5509849 1.0620702  H 4.8224150 -4.4892399 0.3168322  H 3.7987890 -4.5906459 1.7622052  H 4.8056190 -2.4057169 1.9134662  H 4.5810240 -2.0641749 0.1953242  C 2.7865980 -1.9599839 1.3948982  C 2.4362780 -0.5735139 1.2367312  H 2.3097770 -2.4323509 2.2542172  H 3.1411350 0.0195721 0.6507822  C 1.8097410 0.2518161 2.2996012  C 1.3236760 -0.2697429 3.6238072  C 2.6223120 0.4782911 3.5618092  H 1.3157590 1.1366121 1.9109162  H 1.3526280 -1.3367409 3.8262082  H 0.4753020 0.2376531 4.0724382  H 2.6664700 1.4871931 3.9588962  H 3.5401400 -0.0926289 3.6749652  S 3.1538260 -4.2279139 -1.8594938  O 2.1861920 -3.2285509 -2.5051168  O 4.6658310 -4.0529439 -2.0052108  C 2.6515970 -5.9284329 -2.3309458  H 2.8274570 -6.0224819 -3.4029968  H 3.2755020 -6.6071859 -1.7506448  H 1.5957780 -6.0160529 -2.0764218  O 6.2190270 -0.2370699 -0.6505948  O 2.9019000 5.7485511 2.5991012  C 3.7037100 6.8333831 2.1799522  H 4.2730030 7.1421971 3.0567572  H 4.3974980 6.5347571 1.3836852  H 3.0900050 7.6733011 1.8320672  C 7.0920910 0.5114181 0.1723662  H 6.6460420 0.7019151 1.1570892  H 7.9874110 -0.0981639 0.2915532  H 7.3628380 1.4643851 -0.2970528 |
